# Co-dependence between trypanosome nuclear lamina components in nuclear stability and control of gene expression

**DOI:** 10.1093/nar/gkw751

**Published:** 2016-09-12

**Authors:** Luke Maishman, Samson O. Obado, Sam Alsford, Jean-Mathieu Bart, Wei-Ming Chen, Alexander V. Ratushny, Miguel Navarro, David Horn, John D. Aitchison, Brian T. Chait, Michael P. Rout, Mark C. Field

**Affiliations:** 1 School of Life Sciences, University of Dundee, Dundee, Scotland, DD1 5EH, UK; 2 The Rockefeller University, 1230 York Avenue, New York, NY 10065, USA; 3 London School of Hygiene and Tropical Medicine, Keppel Street, London, WC1E 7HT, UK; 4 Instituto de Parasitología y Biomedicina López-Neyra, Consejo Superior de Investigaciones Cientificas, 18100 Grenada, Spain; 5 Center for Infectious Disease Research (formerly Seattle Biomedical Research Institute), Seattle, WA 98109, USA; 6 Institute for Systems Biology, Seattle, WA 98109, USA

## Abstract

The nuclear lamina is a filamentous structure subtending the nuclear envelope and required for chromatin organization, transcriptional regulation and maintaining nuclear structure. The trypanosomatid coiled-coil NUP-1 protein is a lamina component functionally analogous to lamins, the major lamina proteins of metazoa. There is little evidence for shared ancestry, suggesting the presence of a distinct lamina system in trypanosomes. To find additional trypanosomatid lamina components we identified NUP-1 interacting proteins by affinity capture and mass-spectrometry. Multiple components of the nuclear pore complex (NPC) and a second coiled-coil protein, which we termed NUP-2, were found. NUP-2 has a punctate distribution at the nuclear periphery throughout the cell cycle and is in close proximity to NUP-1, the NPCs and telomeric chromosomal regions. RNAi-mediated silencing of NUP-2 leads to severe proliferation defects, gross alterations to nuclear structure, chromosomal organization and nuclear envelope architecture. Further, transcription is altered at telomere-proximal variant surface glycoprotein (VSG) expression sites (ESs), suggesting a role in controlling ES expression, although NUP-2 silencing does not increase VSG switching. Transcriptome analysis suggests specific alterations to Pol I-dependent transcription. NUP-1 is mislocalized in NUP-2 knockdown cells and *vice versa*, implying that NUP-1 and NUP-2 form a co-dependent network and identifying NUP-2 as a second trypanosomatid nuclear lamina component.

## INTRODUCTION

In metazoan cells the structural organization of the nucleus is maintained, at least in part, by the nuclear lamina, a stable protein meshwork at the inner face of the nuclear envelope (NE), comprised of a small family of coiled-coil intermediate filament lamins (1). Mutation or alterations in lamin expression causes abnormalities to nuclear architecture, including irregular protrusions into the cytoplasm, termed nuclear blebs (1,2). Lamins are required for nuclear pore complex (NPC) positioning, with defects leading to NPC clustering (3,4) and/or the absence of NPCs from nuclear blebs (2,5–6). The lamina also interacts with the cytoskeleton through the linker of nucleoskeleton and cytoskeleton complex (LINC) (7,8) and is essential for mechano-signal transduction (9). Lamins are also required for organization of the genome into transcriptionally active euchromatin and repressed heterochromatin (10), and influence transcriptional activity (1,2), interacting with a huge range of transcription factors (11). DNA replication is abolished at the initiation and elongation phases when the lamin network is disrupted (12,13), while lamin mutations lead to increased DNA damage (14). For these and other reasons, hereditary mutations in lamin genes that cause laminopathies (1,5) are of significant clinical interest.

Lamin orthologs are widely distributed across *Metazoa* and social amoeba, and recently have been described as having broad presence, as well as, being absent from several major lineages (1,15–16). Yeast, which are evolutionarily closely related to animals, lack lamins and no lamina structure has been observed by electron microscopy (EM) (16,17). Instead, several proteins appear to have assumed nucleoskeletal functions, e.g. Mlp 1 and 2, large (∼200 kDa) coiled-coil nuclear basket proteins orthologous to the mammalian nuclear basket protein Tpr. Mlp1 and 2 maintain nuclear architecture and NPC organization and interact with Esc1 (18), which itself has roles in telomeric silencing (19), chromatin tethering (20) and organizing the NPC basket (21). For example, over-expressing Esc1 in *Saccharomyces cerevisiae* leads to nuclear blebbing, suggesting a structural system is present in yeasts (22).

In plants a nucleoskeletal structure is also present, but the molecular identity is incompletely defined (23). Nuclear intermediate filament proteins are immunologically identified candidates that form 6–12 nm, lamin-like filaments *in vitro* (24). Another group of candidates are the nuclear matrix constituent proteins at the nuclear periphery. These disassemble and reassemble during mitosis similarly to lamins, affect nuclear size and shape and play a role in heterochromatin organization (23). These examples from yeast and plants suggest that alternative, non-lamin, molecular systems can construct a nuclear lamina.

A functional lamin analog, NUP-1, has been identified in the highly divergent trypanosomatids, which reside within the Excavata supergroup. NUP-1 is a large coiled-coil protein that forms a stable, fenestrated lattice at the edge of the nucleoplasm and expression of NUP-1 is essential for correct nuclear architecture, NPC arrangement, heterochromatin organization and the epigenetic regulation of gene expression (25). A high molecular weight and extended conformation within a relatively small nucleus means that NUP-1 may have roles entirely distinct from lamins, including chromosomal segregation (26). As trypanosomes branched early during eukaryotic evolution (27,28), they are especially valuable for comparative studies.

Many features are conserved between metazoan and trypanosome nuclei, including the NPC transport system (29–33) and peripheral heterochromatin as a transcriptionally repressed portion of the genome (34). The trypanosome nuclear genome is physically segregated into eleven pairs of conventional megabase chromosomes (MBCs) that harbor the majority of protein coding genes, up to five intermediate sized chromosomes (ICs) plus about 100 repetitive lower molecular weight minichromosomes (MCs). MBCs and MCs segregate during mitosis with differential kinetics, locations and possibly mechanisms (35). Transcription of housekeeping genes is polycistronic, with directional gene clusters consisting of functionally unrelated genes (36), while mRNA levels are chiefly regulated post-transcriptionally.

A sophisticated mechanism for immune evasion operates in mammalian infective trypanosomes, involving expression of the variant surface glycoprotein (VSG). VSG expression is monoallelic and exclusively *via* RNA Pol I transcription from telomere-proximal expression sites (ESs), present at both MBC and IC telomeric regions (34). The surface coat is also developmentally regulated and, in early insect stages VSG is replaced by procyclin, another superabundant surface protein. Several proteins mediate repression of inactive VSG genes, including RAP1 (37), DAC3 (38) and NUP-1 (25), while the single active VSG gene is transcribed exclusively at the expression site body, an RNA polymerase I-rich nuclear subdomain distinct from the nucleolus (39).

To further characterize the trypanosome lamina, we investigated the NUP-1 interactome. Amongst many putative interactions, we identified the protein product of Tb927.9.6460, a large, substantially coiled-coil protein essential in the bloodstream form (BSF) (40). We designated Tb927.9.6460 as NUP-2 for nuclear peripheral protein-2 and found that it is widely distributed amongst kinetoplastids but restricted to this lineage. NUP-2 has functions analogous to metazoan lamins, including maintenance of nuclear architecture, chromatin organization and regulation of gene expression and exhibits functional interactions with NUP-1. We therefore define NUP-2 as a second component of the trypanosomatid lamina.

## MATERIALS AND METHODS

### Bioinformatics

BLAST searches were used to identify candidate orthologs in the following predicted proteomes: *T. congolense, T. vivax, T. brucei gambiense, T.grayii, T. cruzi, L. major, L. braziliensis, L. mexicana, L. infantum* and *Bodo saltans* were from geneDB (www.genedb.org/Homepage) and TriTrypDB (http://tritrypdb.org/tritrypdb/) while *Phytomonas serpens* data were from (41). Access to pre-publication predicted proteome data for *T. borreli, T. carassii, T. theileri* and *Euglena gracilis* was kindly provided by Steve Kelly (Oxford) or was from in house sequence data. The NCBI non-redundant protein database was used for searches in other eukaryotes.

JPred (http://www.compbio.dundee.ac.uk/jpred/index.html), COILS (42), InterProScan (https://www.ebi.ac.uk/interpro/search/sequence-search), MotifScan (http://myhits.isb-sib.ch/cgi-bin/motif_scan), Phyre 2 (43), cNLS Mapper (44) were used to predict domains and secondary structure and phosphorylation sites were predicted using Predict Protein (45). Peptide sequences were analyzed for identity and similarity using SIAS (http://imed.med.ucm.es/Tools/sias.html). Phylogenetic trees were created using both PhyML (46) and MrBayes v3.2.1 (47) using multiple sequence alignments generated with Merge Align (48). ProtTest version 2.4 (49) was used to determine the lset rates parameters for MrBayes analyses and the substitution model and gamma parameter for PhyML analyses.

### Trypanosome cell culture

BSF stage and procyclic culture form (PCF) *Trypanosoma brucei brucei* Lister 427 strain were cultured as previously described (50). Single-marker BSF (SMB) and 2T1 BSF cells were used for expression of tetracycline-inducible RNA interference (RNAi) constructs as described (51,52). Reporter 2T1 cell lines (38) were used to investigate telomeric transcriptional regulation.

### *In situ* tagging

The pMOTag vector system (53) was used to introduce C-terminal *in situ* epitope tags, while the pN-PTP derivative vectors (54) were used to introduce N-terminal tags. The primer sequences used were, for C-terminal tagging: NUP-2F CGAAGAGGTCGCACTTCCGGTGGGGCAGGTG GTCCCACTCCCG TTTCCATTACTGGCTCGCTTGGATTGAAGCCATCGGGT ACCGGGCCCCCCCTCGAG, NUP-2R AACTATTCAGTAACGCTTCCATATAATA GATAATATATATATATATATGTTTGGGTGTGTGCTCGTCGTCACGATGGCGGCCGCTCTAGAACTAGTGGAT, *Tb*Nup98F TGGGAATGCTTCAGCAAGTGGT GAAAAGAACAATGCTCCACGGAATCCCTTCTCATTTGGTGCCTCTTCTGGGAATGCTGGTACCGGGCCCCCCCTCGAG and *Tb*Nup98R ACTAAAGAAGG GTAGAAAACAAAGAAAACACCAAATAAGGTACCTGACGCAGC GGCAACACCACGTCGACTTGCTGGCGGCCGCTCTAGAACTAGTGGAT, and for N-terminal tagging; NUP-2F CTTAAGCTTC TATGATCGCTGCGGGCAATGAAAGC and NUP-2R CAGTAAGAATTCC AGCGGCTGAGAGCTGAGAA. All sequences are given in the 5′–3′ orientation. Linear polymerase chain reaction (PCR) products were purified and sterilized by ethanol precipitation. Electroporation was performed with 10–25 μg of DNA using an Amaxa Nucleofector II for BSF trypanosomes and a Bio-Rad Gene Pulser II (1.5 kV and 25 μF) for PCF trypanosomes. Positive clones were assayed for correct insertion and expression by Western blot.

### Western blotting

Normally 1 × 10^7^ cells per lane were resolved on a 4–12% SDS–PAGE gel (Invitrogen) or a home-made 8% SDS-PAGE gel. Proteins were transferred to a nitrocellulose membrane (Whatman). The following primary antibodies were used: HA mouse monoclonal (F7), at 1:10 000 (from Santa Cruz, sc-7392), β-Tubulin mouse monoclonal (KMX-1) at 1:5000 (from Millipore, MAB3408), Detection was by chemiluminescence with luminol using rabbit anti-mouse peroxidase conjugate at 1:10 000 (Sigma, A9044). Images of developed films were analyzed using ImageJ (National Institutes of Health).

### Isolation of protein complexes

Protein–protein interactions were analyzed by cryomilling of cell pellets and then immunoprecipitation, approximately as outlined in (55). In brief, ∼10^10^ PCF trypanosomes harboring an endogenous GPF tag on the affinity handle protein were harvested, frozen and then lysed by mechanical milling in a Retsch Planetary Ball Mill PM100 using liquid nitrogen cooling (Retsch, UK). Aliquots of frozen powder were thawed in buffer (see figure legends) containing protease inhibitors (Roche) and clarified by centrifugation. Tagged proteins were affinity isolated using polyclonal llama anti-GFP antibodies coupled to magnetic beads (Dynabeads^®^). Protein complexes were then fractionated using 1D SDS–PAGE and gel bands excised, trypsin digested and identified by matrix-assisted laser desorption/ionization – time of flight (MALDI-TOF) mass spectrometry (29,56).

### RNA interference

The online RNAit tool (57) was used to design primers for NUP-2 RNAi, to avoid potential off-target effects. The sequences used were, 5′ to 3′; NUP-2F TGAACAGCAAGGGCTCTTTT and NUP-2R GCCTCATGGCTTCTTAGCAC. For some experiments PCR products were cloned into the p2T7^TABlue^ plasmid (51) and transfected into single marker T7 RNAP/TetR BSF (SMB) cells (58). Alternatively, the pRPaiSL plasmid was used to generate targeted stem-loop constructs that were transfected into 2T1 BSF cells (52,59).

### Immunofluorescence microscopy

Samples were prepared for microscopy as described in (60). The following primary antibodies were used: HA mouse monoclonal (F7) at 1:500 – 1:1000, (from Santa Cruz, sc-7392), β-Tubulin mouse monoclonal (KMX-1) at 1:1000 (from Millipore, MAB3408), GFP lapine polyclonal at 1:3000 (in house) and NUP-1 lapine polyclonal at 1:750–1:1500 (in house). Secondary antibodies for IFA were used at 1:1000, and were goat anti-rabbit Oregon green (Invitrogen, O-6381), goat anti-rabbit A568 (Invitrogen, A-11011), goat anti-mouse A568 (Invitrogen, A-11001 and A-11004). Wide-field microscopy was carried out using a Nikon Eclipse E600 epifluorescence microscope with a camera and images captured using Metamorph software (Universal Imaging Corporation). Confocal microscopy was carried out on an SP2-visible inverted confocal microscope (Leica Microsystems) or an LSM 700 confocal microscope (Zeiss) and images deconvolved using Huygens Professional program (Scientific Volume Imaging). Image quantification was carried out on raw images (wide-field microscopy) or raw deconvolved images (confocal microscopy) using ImageJ. Image processing for presentation was carried out using Adobe Photoshop 7.0 (Adobe Systems). When measuring fluorescence intensity for NUP-2 or NUP-1, for each cell quantified a separately focused image set was captured. The DAPI signal was used as a guide for the nucleus region of interest, from which the target mean fluorescence intensity (MFI) was quantified using ImageJ. For each nucleus, local background MFI (outside the cell) was deducted from the nuclear measurement.

### Fluorescence *in situ* hybridization

Telomere Fluorescence *in situ* hybridization (FISH) was performed as described (25) except that for co-stain with the 177 base pair fragment that binds to mini-chromosomes, adjusted BMEB (20% v/v FCS in 100 mM maleic acid, 150 mM NaCl, pH 7.5) was used in place of BMEB.

### Cell-sorting

Mid-log phase cells were harvested by centrifugation and washed extremely gently in Voorheis's-modified phosphate-buffered saline (vPBS) (60). The cells were fixed in 70% methanol in phosphate buffered saline (PBS) at 4°C for 1 h, washed in vPBS and stained in 30 μg/ml propridium iodide (PI) with 10 μg/ml RNAse A in PBS for 45 min at 37°C. The samples were analyzed using a CyAn ADP MLE flow cytometer (Beckman Coulter).

### Transmission electron microscopy

Trypanosome cells were fixed approximately as described in (61) and then post-fixed and stained by the University of Cambridge multi-imaging center as outlined below: Samples were fixed in 4% glutaraldehyde in 0.1 M HEPES buffer at pH 7.4 for 12 h at 4°C, rinsed five times in 0.1 M HEPES buffer, then treated with 1% osmium ferricyanide for 2 h at RT. They were rinsed five times in deionized water and treated with 2% uranyl acetate in 0.05 M maleate buffer at pH 5.5 for 2 h at RT. They were again rinsed in deionized water and dehydrated in an ascending series of ethanol solutions from 70% to 100%. This was followed by treatment with two changes of dry acetonitrile and infiltration with Quetol epoxy resin. Sections of 50–70 nm were used for imaging; images were taken in an FEI Tecnai G2 operated at 120 Kv using an AMT XR60B digital camera running Deben software.

### qRT-PCR

Reverse transcription and qRT-PCR were carried out using 1 μg rather RNA and Superscript III Reverse Transcriptase (Invitrogen). The primer sequences, 5′ to 3′, were NUP-2F GATGCGATTCGCTCACTG, NUP-2R CCGCCTCCTTCTTTTTCA, PFRF GAAGTTGAAGGTGTTGTGAGTCC, PFRR CCTCCAGCGTGATATCTGTTACC, VSG2F CCAAGTTAACGACTATACTTGCCTATT, VSG2R CAAGTAGCAAGGAAAATTTTAAAAGG, VSG16F AGTCGTAGCACTTTTGATTCAGG, VSG16R TTATGCTAAAAACAAAACCGCA, VSG21F CGGATGCTCAAATCTATTACACAG, VSG21R GTCAGAATTCTTAGAATGCAGCC, NPTF TCTGGATTCATCGACTGTGG, NPTR GCGATACCGTAAAGCACGAG, GFP:NPTF CTGCTGCCCGACAACCA and GFP:NPTR TGTGATCGCGCTTCTCGTT.

### Active expression site positional analysis

RNAi was induced for 24 h in a cell line with a GFP-tagged active *VSG* ES promoter. Cells were differentiated and analyzed as described (62).

### Transcriptome analysis

Total RNA was extracted from cells induced for RNAi against NUP-2 for 0, 12, 24 and 48 h using the RNeasy system (Qiagen); three separate cultures were used for each time point and the samples processed individually. RNA libraries were constructed using a Truseq mRNA stranded kit and polyA selected for enrichment and quantified by KAPA library quantification kit. The library size and distribution were assayed on a bioanalyzer using the Agilent DNA 1000 Kit and normalized to 10 nM. Sequencing was performed on a NextSeq500 High output 1 × 75 bp v2 flowcell. RNA-Seq single-end reads of 75bp were mapped to the *T. brucei* 427 strain reference genome sequence using BWA (Burrows–Wheeler Aligner). The mapped reads were filtered by SAMtools based on the MAPping Quality (MAPQ) values with a minimum MAPQ = 20. After filtering, the reads were aligned to annotated transcripts by BEDtools. The transcript abundances were calculated based on RPKM (Reads Per Kilobase of transcript per Million mapped reads) with an upper bound quantile normalization. Differentially expressed genes with positive false discovery rates (pFDR) < 0.05 and absolute fold-change > 2 at least in one time point versus the control were hierarchically clustered using the *clustergram* MATLAB function. pFDR values were calculated using the *mafdr* MATLAB function.

## RESULTS

### Identification of NUP-1 interactions

**Figure 1.**
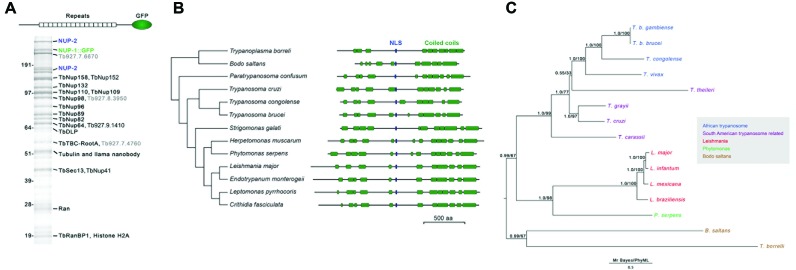
The trypanosome lamin analog NUP-1 interacts with the NPC and a novel high molecular weight protein. (**A**) NUP-1 was co-immunoprecipitated *via* a C-terminal *in situ* GFP tag in 20 mM HEPES, pH 7.4, 250 mM NaCl, 0.5% Triton, 0.5% deoxy-BigCHAP using cryomilling and affinity isolation with anti-GFP antibody. The resulting complexes were fractionated by 1D SDS-PAGE and visualized by staining with Coomassie blue. The identity of protein bands was determined by mass spectrometry. The marker positions of a co-electrophoresed protein ladder are indicated to the left, values are in kilodaltons. Grey accession numbers indicate proteins with no published or predicted function. (**B**) Predicted architecture of NUP-2 orthologs and the major predicted coiled-coil domains and nuclear localization signal (NLS) motifs. Sequence ribbons are aligned to the major C-terminal coiled-coil domain, which is the most conserved region. The coiled-coil domains and total length of each sequence are approximately to scale on the horizontal axis. Species names and total amino acid residues are indicated at left and right respectively. (**C**) A pseudo-rooted phylogenetic tree for NUP-2 orthologs identified by protein BLAST searches, *T. borrelli* was treated as an out-group. Both MrBayes and PhyML algorithms were used to model the relationships between sequences, and the PhyML topology is shown. MrBayes and PhyML posterior probability and bootstrap values respectively are shown at each node. The key indicates a color code for species groups.

To identify candidate protein interactions between NUP-1 and additional nuclear envelope proteins we affinity captured NUP-1::GFP using anti-GFP antibodies. Cells were snap frozen and lysed under cryogenic conditions, to help preserve protein–protein interactions (Figure 1A) (33). Analysis of these NUP-1 isolates by SDS-PAGE indicated a rather complex pattern, with multiple individual protein bands present to near apparent stoichiometric levels with NUP-1::GFP. Repeat isolations were highly consistent (data not shown).

Analysis of the NUP-1 isolates using MALDI-TOF MS and ESI-MS^2^ identified multiple components of the NPC (35,55), indicating an association between NUP-1 and the NPC, most likely mediated primarily by nuclear basket nucleoporins, although these interactions may be indirect. Identification of these nucleoporins likely reports indirect connections *via* mutual NPC association. Detection of the histone H2A may suggest a direct interaction with chromatin, but as H2A is highly abundant the significance is unclear. Tubulin and the dynamin-like protein (63) are frequently found in NPC pullouts (33) and probably are non-specific, as neither is known to associate specifically with NUP-1 based on localization (63).

Significantly, one protein with slow mobility, the product of Tb927.9.6460, and which appears, based on localization (below), not to be a nucleoporin, was identified and designated NUP-2 for nuclear periphery protein-2. NUP-2 migrated as two distinct molecular weights, at the predicted molecular weight of 170 kDa and a slower migrating form; this latter may correspond to a post-translational or otherwise modified form and was not investigated further. We chose NUP-2 for detailed study due to a high molecular weight (170 kDa), which suggested a possible role as a structural protein, the specificity of the interaction with NUP-1 and apparent lineage-specific distribution.

### NUP-2 possesses predicted coiled-coil domains

NUP-2 is predicted to be a large, moderately acidic protein (pI 5.0, MW 170 kDa) that is predominately α-helical with a central monopartite nuclear localization signal (NLS) and four major coiled-coil regions (Figure 1B). No *trans-*membrane domains or β-sheet secondary structure was predicted, but NUP-2 is phosphorylated at several sites (64).

A single NUP-2 coding sequence was identified in all kinetoplastids, but searches of the *Naegleria gruberi* genome and more phylogenetically distant taxa failed to identify NUP-2 orthologs. Trypanosomatid NUP-2 orthologs are syntenic, including *Crithidia* (65). Phylogenetic reconstruction of relationships between NUP-2 orthologs (Figure 1C) closely reflects the taxonomic relationships, suggesting an origin at the base of the trypanosomatids and vertical evolution. Sequence similarity/identity of orthologs with *T. brucei* decreases gradually with taxonomic distance (Supplementary Figure S1). Despite possessing comparable arrangements of coiled-coil domains, NUP-2 orthologs vary quite considerably in length between species (Figure 1B), such that *Leishmania major* NUP-2 is 25% larger than *T. brucei* NUP-2, likely due to an N-terminal extension and several indels within the central portion of the protein (data not shown). The presence of an ortholog in *B. saltans* indicates that NUP-2 is not associated with parasitism, but is an ancient feature of the lineage, in common with NUP-1 (66). The central predicted NLS is conserved across all orthologs, and in several species an additional NLS is also predicted toward the C-terminus (Supplementary Figure S2).

### NUP-2 localizes to the nuclear periphery

**Figure 2.**
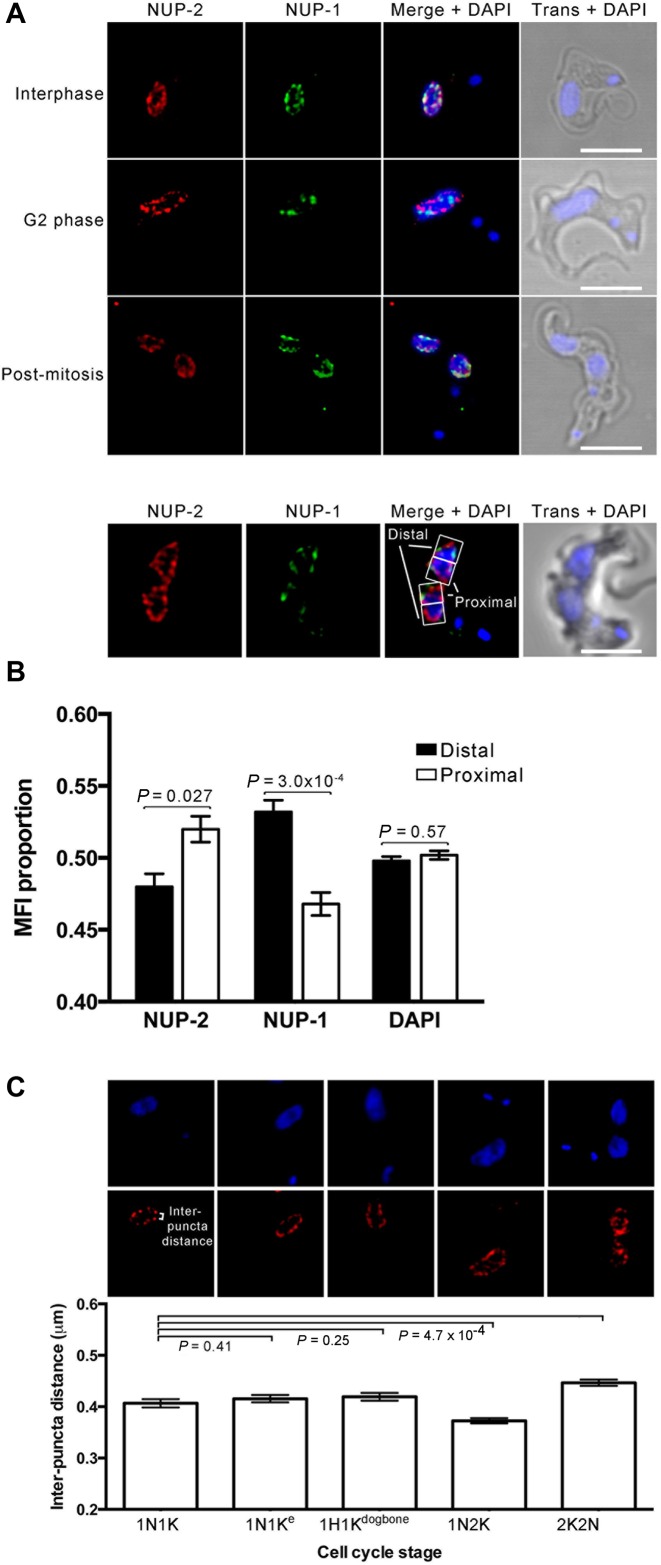
NUP-2 is located at the nuclear periphery in a similar arrangement to NUP-1. All images are slices from deconvolved confocal z-stacks. NUP-2 was visualized by a C-terminal *in situ* 3xHA epitope tag; NUP-1 was visualized using a polyclonal antibody against the repeat region. (**A**) Localization of NUP-2 (red) and NUP-1 (green) at several cell cycle stages. Scale bar represents 5 μm. Trans = transmitted light, phase contrast. (**B**, top) Localization of NUP-2 compared to that of NUP-1 in daughter nuclei immediately following mitosis. Representative proximal and distal regions of interest for quantitation of fluorescence are shown. (**B**, bottom) Mean proportion of NUP-2, NUP-1 and DAPI fluorescence in proximal and distal regions of interest. Error bars represent SEM for 40 nuclei from 20 post-mitotic cells. Statistical significance was determined using a paired Student's *t*-test; *P*-values are indicated in the top part of the chart. (**C**, top) Localization of NUP-2 in the equatorial plane of the nucleus throughout the cell cycle, showing changes in distance between NUP-2 puncta. (**C**, bottom) Mean distance between NUP-2 puncta at the nuclear equator in the five stages of the cell cycle represented by the images above. Error bars are SEM for between 188 and 346 measurements from 14 or more cells at each cell cycle stage. Statistical significance was determined using Student's *t*-test with unequal variance; *P*-values are indicated in the top part of the chart. 1N1K cells represent cells in interphase, and 1N1K^e^ are cells that have an elongated kinetoplast, and which indicates a point in the cell cycle at the initiation of nuclear S-phase. 1N1K^dogbone^ is a later stage where kinetoplast DNA replication is complete and the two daughters are beginning to segregate.

We established the location of NUP-2 by fusion of GFP to the C-terminus of one allele, followed by confocal microscopy. The NUP-2::GFP fusion was observed at the nuclear periphery throughout the cell cycle in an arrangement similar to, but distinguishable from NUP-1 (Figure 2A). NUP-1 and NUP-2 localisations appeared most similar at interphase. NUP-2 was also expressed at a similar level in both major life-cycle forms and interphase localization was indistinguishable between insect or mammalian stage cells (Supplementary Figure S3).

Quantitative analysis of mitotic cells indicated that NUP-2 did not polarize toward the distal halves of daughter nuclei as observed for NUP-1 (25); rather NUP-2 was slightly biased toward the proximal region (Figure 2B). We also observed changes in NUP-2 puncta density at the nuclear periphery during the cell cycle. During interphase and the very earliest stages of mitosis (1N1K and 1NK1e, where ‘e’ designates kinetoplast elongation), NUP-2 remains widely dispersed about the nuclear circumference and is most dispersed in post-mitotic cells (2N2K). However, this is reversed during from S-phase to early mitosis (1N2K cells), suggesting that NUP-2 distribution becomes more compacted just prior to nuclear scission (Figure 2C). Therefore, NUP-2 distribution is subject to cell cycle dependent alterations, but which are distinct from NUP-1.

**Figure 3.**
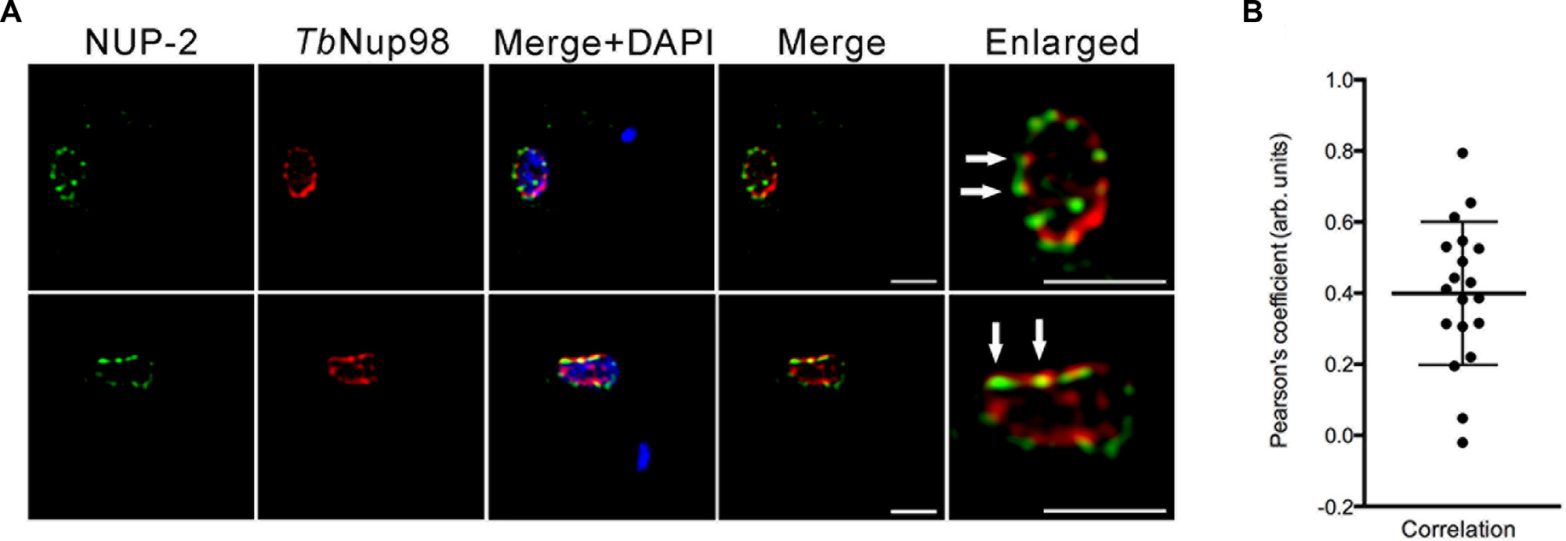
NUP-2 is not part of the nuclear pore complex. (**A**) Localization of NUP-2 (green) and TbNup98 (red) in interphase cells. Images are slices from deconvolved confocal z-stacks. NUP-2 was visualized by a C-terminal *in situ* 13 x myc epitope tag; TbNup98 was visualized using a C-terminal *in situ* 3 x HA epitope. White arrows indicate some example juxtaposed NUP-2 and *Tb*Nup98 puncta in the enlarged images. (**B**) Pearson's correlation coefficient of NUP-2::13 x myc with TbNup98::3 x HA for 10 interphase nuclei in separate deconvolved confocal images.

To examine the relationship between NUP-2 and the NPC we tagged the nucleoporin TbNup98 at the C-terminus with three HA epitopes (33). TbNup98 is repetitive at the C-terminus, and to mitigate against creation of a truncation we assessed both the size of the tagged protein and amplified a region from the 3′ end of the gene to ensure that integration was close to the annotated termination codon (Supplementary Figure S4). NUP-2 did not completely co-localize with TbNup98::3HA, although both were juxtaposed frequently, suggesting that while NUP-2 contacts the nuclear face of the NPC, an intimate association is unlikely (white arrows, Figure 3).

### NUP-2 is required to maintain multiple aspects of nuclear architecture

**Figure 4.**
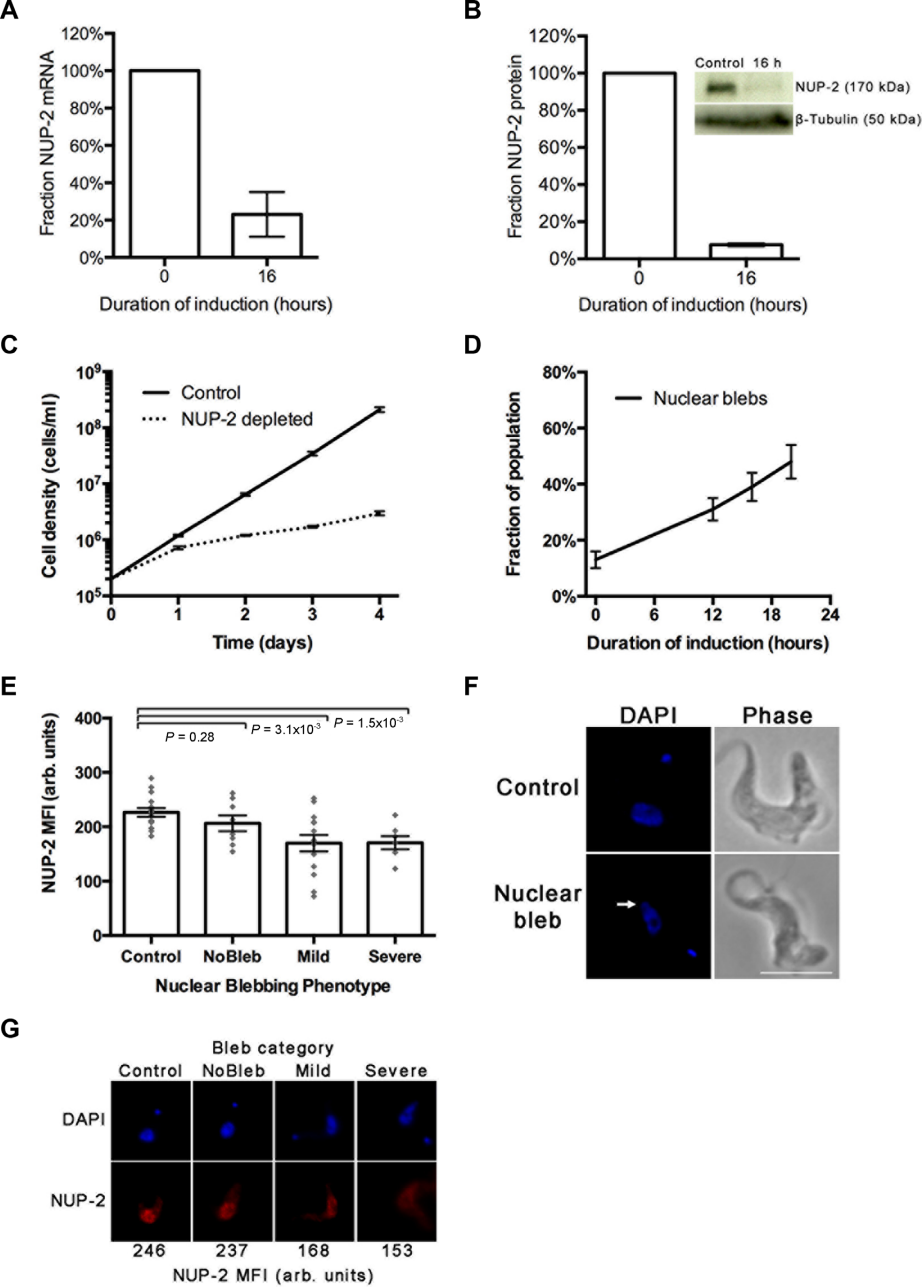
NUP-2 is required for proliferation and to maintain normal nuclear morphology. (**A**) The fraction of NUP-2 mRNA remaining after 16 h RNAi, as determined by qRT-PCR. Data were normalized to PFR in each sample as an internal control and induced and control samples for each replicate were analyzed on the same qRT-PCR plate. Error bars denote SEM for three biological replicates. (**B**) The fraction of NUP-2 protein remaining after 16 h induction of RNAi, determined by semi-quantitative Western blot using a NUP-2 C-terminal *in situ* 13x myc epitope tag. The intensity of protein bands was measured using ImageJ. Data were normalized to ß-tubulin as a loading control. Error bars denote SEM for three biological replicates. (**C**) Cumulative growth curve for NUP-2 RNAi induced (dotted line) or uninduced (solid line). Cell density was monitored every 24 h and maintained in mid-logarithmic phase. (**D**) The proportion of cells that have abnormal nuclear extrusions or ‘blebs’ at various time-points during NUP-2 RNAi induction. A low background level of nuclear blebs was consistently observed and may result from slight leakage of the RNAi construct in non-induced cultures. Error bars denote SEM for two biological replicates, each comprising 100 cells at every time-point. (**E**) The intensity of the NUP-2 signal in the nucleus of interphase cells across NUP-2 depleted and control populations, as determined from wide field images. Control denotes cells from an uninduced culture. Other categories represent cells from the same induced culture. Bleb size was measured as a proportion of the longest nuclear diameter (ND), not including the bleb. None; 0% ND, Mild; single bleb at 20 - 100% ND, Severe; single bleb greater than 100% ND or two or more blebs greater than 50% ND. Error bars denote SEM for between 7 and 15 measurements. Statistical significance was tested using the nonparametric Mann Whitney U test. (**F**) An example image of a bleb nucleus from NUP-2 depleted culture, as compared to a control cell. Images are at the same magnification, scale bar = 5 μm. (**G**) Representative images from each category in panel E. The mean fluorescence index recorded for NUP-2 fluorescence within the nucleus for each of these images is indicated at the bottom.

NUP-2 function was investigated by RNAi. After 16 h induction, NUP-2 mRNA and protein were reduced by more than 80% (Figure 4A and B), which led to substantial proliferation defects (Figure 4C) and perturbed cell cycle progression (Supplementary Figure S5A). There was increased frequency of cells with abnormal DNA content in NUP-2 depleted culture, including ‘monsters’ (>2 kinetoplasts and/or >2 nuclei) (Supplementary Figure S5A) and cells detected by FACS as having DNA content outside the normal cell cycle range (Supplementary Figure S5C). The presence of cells with abnormal DNA content and especially those with more than two nuclei, but the continued division of nuclei, suggests a failure to complete cytokinesis, which is likely a secondary effect resulting from perturbations in mRNA and protein expression, and not a direct impact on DNA replication.

**Figure 5.**
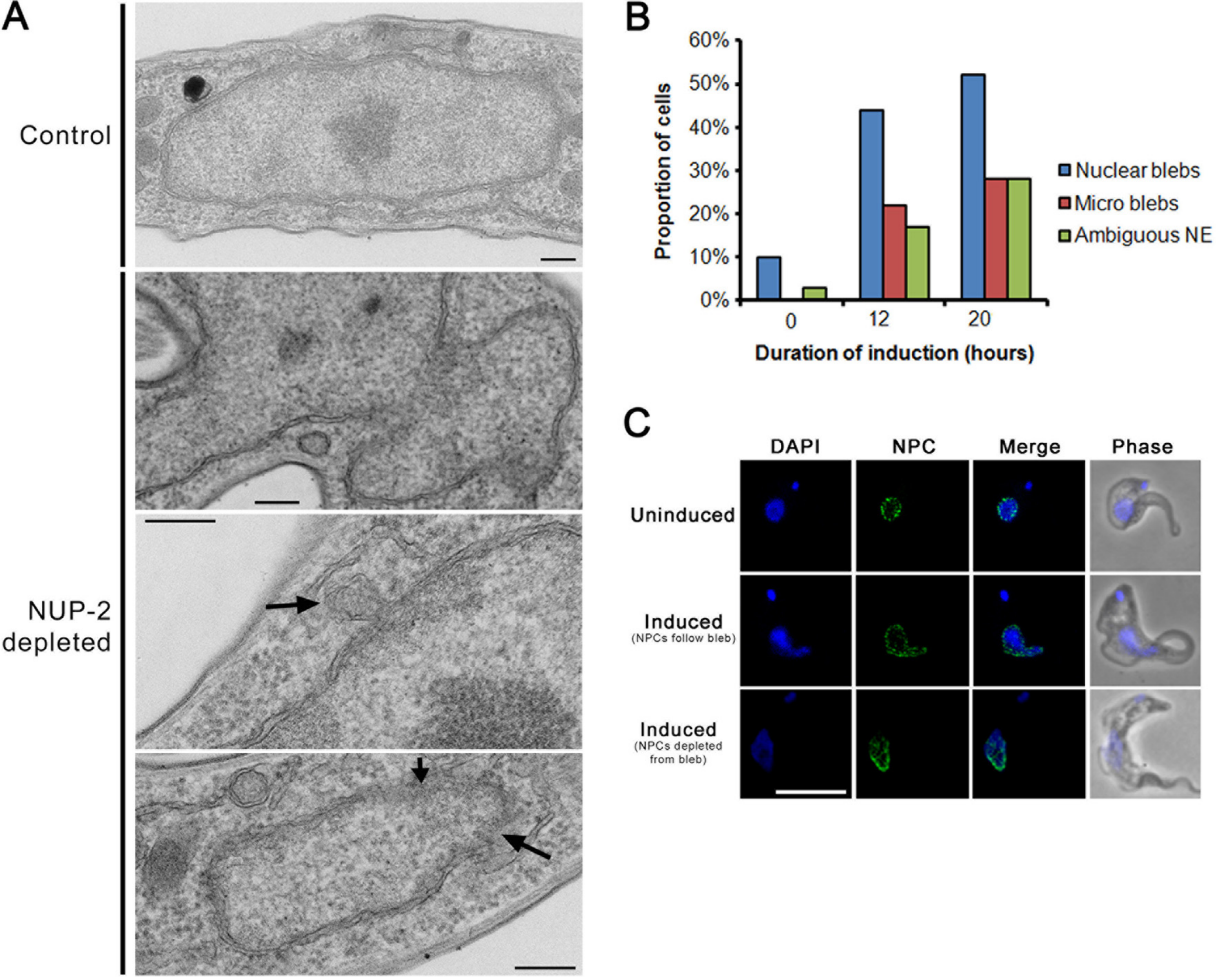
Loss of NUP-2 affects nuclear architecture and NPC organization. (**A**) Example of transmission electron microscopy (TEM) images of nuclei ultrastructure from uninduced (control) and NUP-2 depleted samples. The three major phenotypes observed are represented. The nuclear blebs already recorded by light microscopy are clearly visible in the top NUP-2 depleted image. Dramatic undulations in the nuclear envelope less than 100 nm in diameter termed ‘micro-blebs’ are represented in the second NUP-2 depleted image (indicated by an arrow). Poorly defined or ‘ambiguous’ regions of the nuclear envelope are identified by arrows in the lower image. Scale bars indicate 200 nm. (**B**) The proportion of cells exhibiting morphological defects following NUP-2 depletion as observed by TEM. Examples of nuclear blebs, micro blebs and indistinct nuclear envelope are represented in (A). Data represent observations from 15–25 cells per time point. (**C**) Example images showing the arrangement of NPCs in uninduced cells (top panel) and the two major phenotypes observed in blebbing nuclei from NUP-2 depleted populations. Note that the NPCs either trace the outline of the bleb (middle panel) or appear to be specifically depleted from the nuclear envelope that is incorporated into the bleb (bottom panel). Images are deconvolved slices from a confocal z-stack. All the images are at the same magnification; the scale bar represents 5 μm.

Several lamin mutations lead to nuclear protrusions or blebs (1), indicating a nuclear envelope structural deformity (15). Similar nuclear abnormalities were apparent in NUP-2 depleted cells; the proportion of cells with nuclear blebs increased through time following induction (Figure 4D and F), and the intensity of NUP-2 fluorescence inversely correlated with nuclear bleb frequency (Figure 4E). Example images for nuclear defects and NUP-2 mean fluorescence intensity (MFI) are shown in Figure 4G. Surprisingly, considering the substantial effect of NUP-2 depletion on gross nuclear morphology, we observed no alteration in the frequency of mitotic spindles (Supplementary Figure S6), excluding a major role in spindle formation. Nuclear blebs were also observed in knockdown cells by transmission electron microscopy; invaginations <100 nm in diameter and ill-defined portions of the NE, possibly due to NE disorganization or crenellation were observed (Figure 5A and B).

NUP-1 depletion leads to NPC clustering (25), suggesting that the trypanosomal lamina plays a role in NPC positioning. We monitored TbNup98::3HA during NUP-2 silencing. Depletion of NPCs from nuclear blebs was observed in a significant subset of blebbing nuclei (*P* = 0.006, Fisher's exact test, n = 24 uninduced, n = 14 bleb). NPCs were absent from the bleb membrane itself and depleted from the intersection between the bleb and the nuclear rump (Figure 5C, lower induced image panel), and unlike NUP-1, NPC clustering was not evident suggesting that NPCs do not depend on NUP-2 for positioning, despite juxtaposition and proteomic evidence supporting an interaction. Overall, these data suggest that NUP-2 expression is important for maintaining nuclear envelope morphology, but does not play a major role in NPC positioning or spindle formation.

### NUP-2 interacts with the NPC and NUP-1

**Figure 6.**
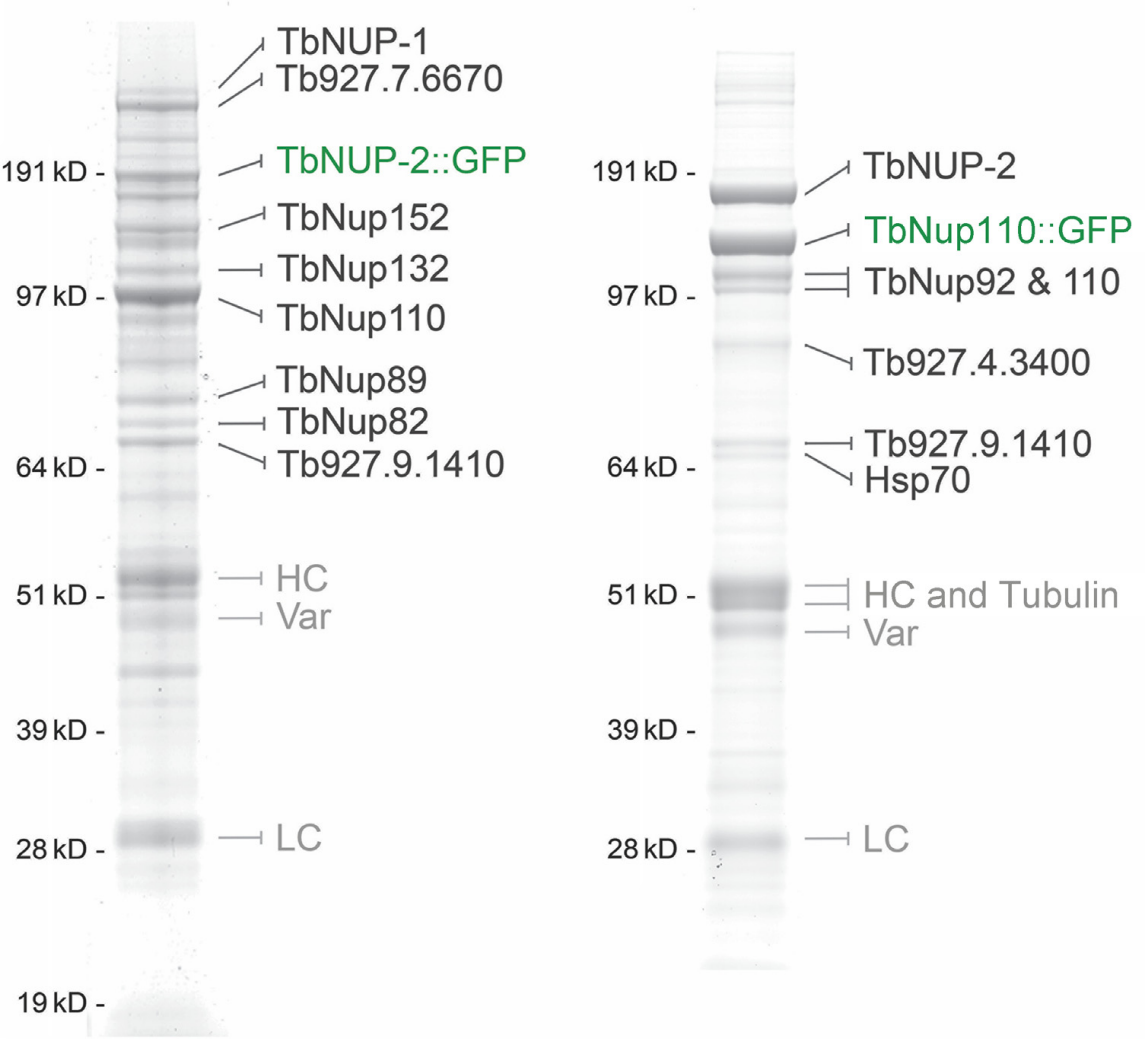
Affinity isolation of NUP-2 containing complexes. Proteins from each affinity isolation preparation were identified by mass spectrometry in the shown or parallel affinity capture preparations. NUP-2 is shown to interact with components of the NPC, and specifically, putative constituents of the NPC core scaffold, as well as the NPC nuclear basket protein TbNup110 and the nuclear lamina component NUP-1. Conditions for the isolation in left panels are 20 mM HEPES, pH 7.4, 250 mM NaCl, 0.5% Triton and for right panel 40 mM Tris, pH 7.4, 500 mM NaCl, 1% CHAPS.

To understand the relationships between NUP-2, NUP-1 and the NPC further we affinity isolated NUP-2::GFP complexes using the same strategy as for NUP-1. In addition to confirming an interaction with NUP-1, we found that NUP-2 forms interactions with nucleoporins including the predicted scaffold-forming nucleoporins (TbNup82, 89, 132) and significantly the nuclear basket nucleoporin TbNup110 (29,55) (Figure 6). Additionally, TbNUP-2 also interacts with several proteins of unknown function, which also localize to the nuclear envelope (SO, MCF and MPR, unpublished).

The number of interactions recovered between NUP-2 and the NPC are not as extensive as for NUP-1. We suggest that interactions with the NPC may be mediated via the nuclear basket protein TbNup110, whose abundance in isolated NUP-2 complexes is significantly higher than other co-purified proteins. TbNup110 is known to form an extensive interaction with the entire trypanosome NPC (33,55). The strength of the NUP-2/TbNup110 interaction is supported by the recovery of stoichiometric amounts of NUP-2 in a reverse affinity capture experiment using TbNup110::GFP as bait in a more stringent buffer containing 0.5 M NaCl (Figure 6). We also recovered, at lower abundance, a second characterized nuclear basket protein TbNup92 (55,67) and several non-NPC proteins. This suggests that we can specifically isolate the nuclear basket and associated proteins, and together with the localization and knockdown evidence, this suggests that TbNUP-2 binds to the NPC, and most probably this is *via* the nuclear basket.

### NUP-2 is required for chromosomal organization

**Figure 7.**
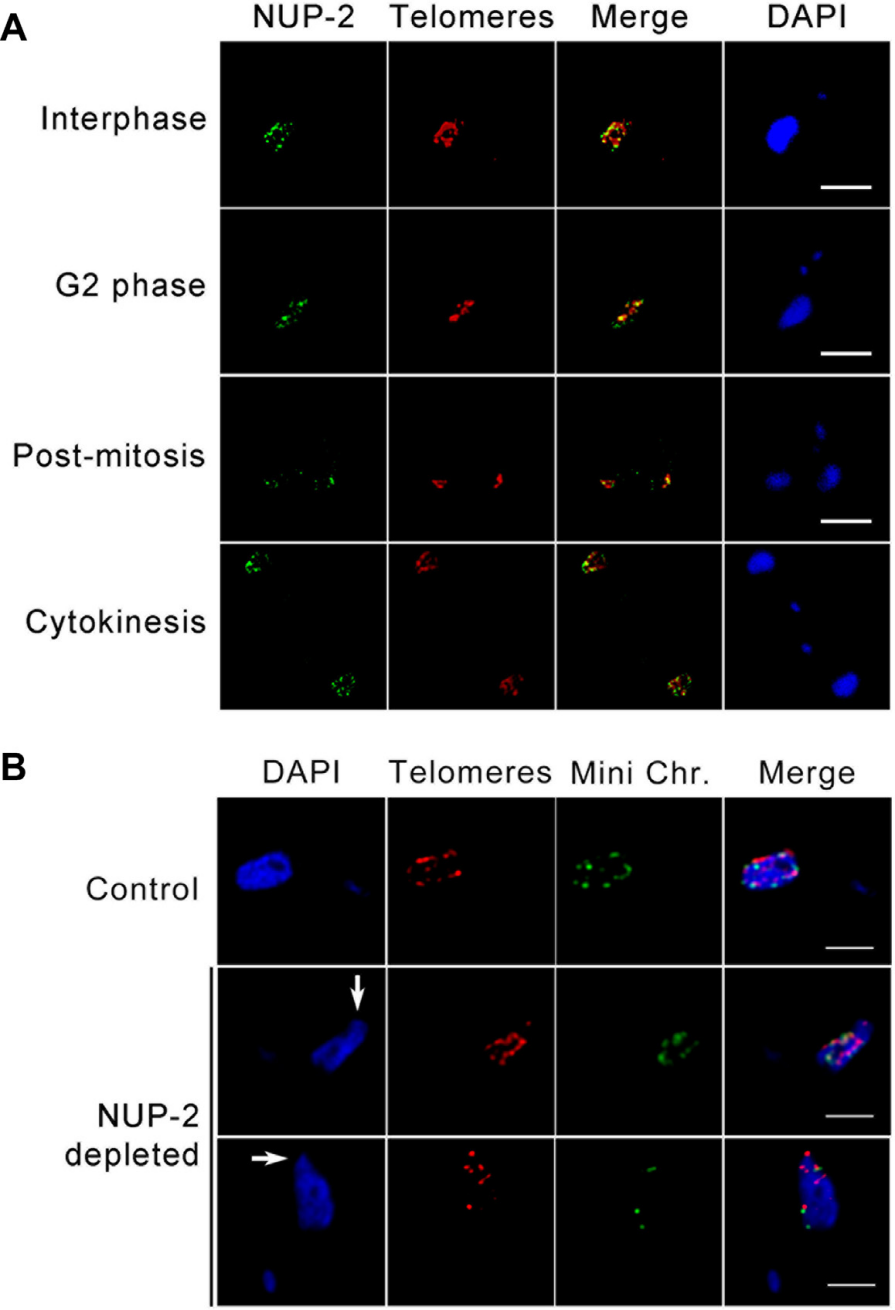
Chromosomal organization is dependent on NUP-2. (**A**) Localization of NUP-2 (green) and telomere repeats (red) throughout the bloodstream form (BSF) cell cycle. Images are slices from deconvolved confocal z-stacks. NUP-2 was visualized using a C-terminal *in situ* 3xHA epitope; telomeres were visualized using telomere-FISH. Images are at the same magnification, scale bar indicates 5 μm. (**B**) Representative images showing the location of all telomeres (red) and mini-chromosomes (green) in interphase cells from uninduced (control) and blebbing nuclei from NUP-2 depleted culture. Nuclear blebs are indicated by white arrows. The telomeres of all chromosomes were visualized by telomere-FISH, while mini-chromosomes were stained using a digoxigenin labelled DNA probe for the 177 bp repeat region. Images are from deconvolved confocal z-stacks. Scale bars denote 2 μm.

Given the apparent broad similarities between NUP-1 and NUP-2 silencing phenotypes in maintaining nuclear morphology, we analyzed the role of NUP-2 in chromatin organization. FISH using a telomeric probe and immunofluorescence for NUP-2::3HA demonstrated that NUP-2 and telomeres are in close proximity through most of the cell cycle (Figure 7A), except during mitosis when telomeres migrate into the nuclear interior and NUP-2 remains at the nuclear periphery. Furthermore, telomeres became disorganized in NUP-2 RNAi cells, a phenotype similar to NUP-1 (25). Telomeres are frequently mis-arranged in interphase blebbed nuclei, locate within blebs and there are frequently large circumferential gaps in telomere distribution, suggesting their absence from their normal positions at the nuclear periphery (Figure 7B).

To determine if telomeric mislocalization was global, or specific to a class of chromosomes, we used the telomeric probe and a mini-chromosome specific 177 base-pair repeat probe (177bpRP) to visualize either all telomeres or the mini-chromosomes specifically. The telomeric FISH alone, i.e. stained with the telomere repeat, indicates megabase chromosomes and/or intermediate chromosomes. Whilst telomeres were slightly more frequent in nuclear blebs than mini-chromosome repeats (76% and 60% respectively, n > 50 cells), it appears that both classes of element are affected by the NUP-2 knockdown, suggesting a general impact on chromosome and telomeric localization (Figure 7B).

### NUP-1 and NUP-2 localizations are codependent

In metazoan cells the location of one lamin sub-type is frequently affected by mutation or depletion of other subtypes, suggesting their interdependence in constructing and/or maintaining the mammalian lamina (2–3,5). However, while evolutionary reconstructions indicate that metazoan lamins are paralogous, we find no evidence for a common origin for NUP-1 and NUP-2. To directly seek evidence for codependence between NUP-1 and NUP-2, the localization and fluorescence intensity at the nuclear periphery of each protein was assessed, under conditions where the mRNA of the other was knocked down.

**Figure 8.**
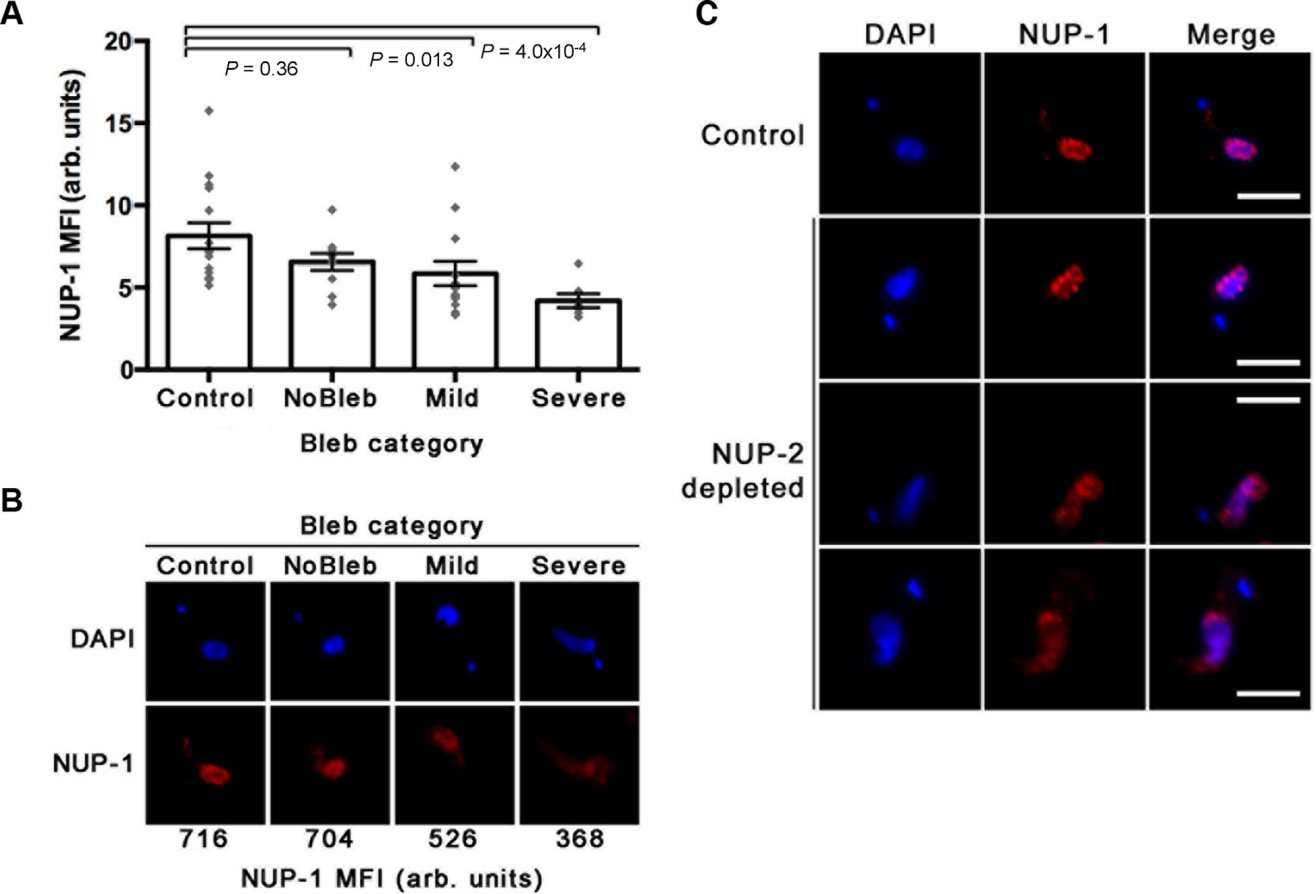
NUP-2 is required for the stability of the NUP-1 network. The fluorescence intensity and localization of NUP-1 are affected in blebbing nuclei of NUP-2 depleted cells. (**A**) The intensity of NUP-1 fluorescence in the nucleus of interphase cells across NUP-2 depleted and control populations, as determined from wide field images. Control denotes cells from an uninduced culture. The other categories represent cells from the same induced culture. Bleb size categorized as in Figure 4 Panel E. Error bars denote SEM for between 7 and 15 measurements. Statistical significance was tested using the non-parametric Mann–Whitney U-test. (**B**) Representative images from each category in panel A. The MFI recorded for NUP-1 signal within the nucleus for each of these images is indicated at the bottom. (**C**) Example images showing abnormal NUP-1 localization in blebbing NUP-2 depleted nuclei. Images are wide-field microscopy using an antibody against the repeat region to visualize NUP-1. Scale bar denotes 5 μm.

**Figure 9.**
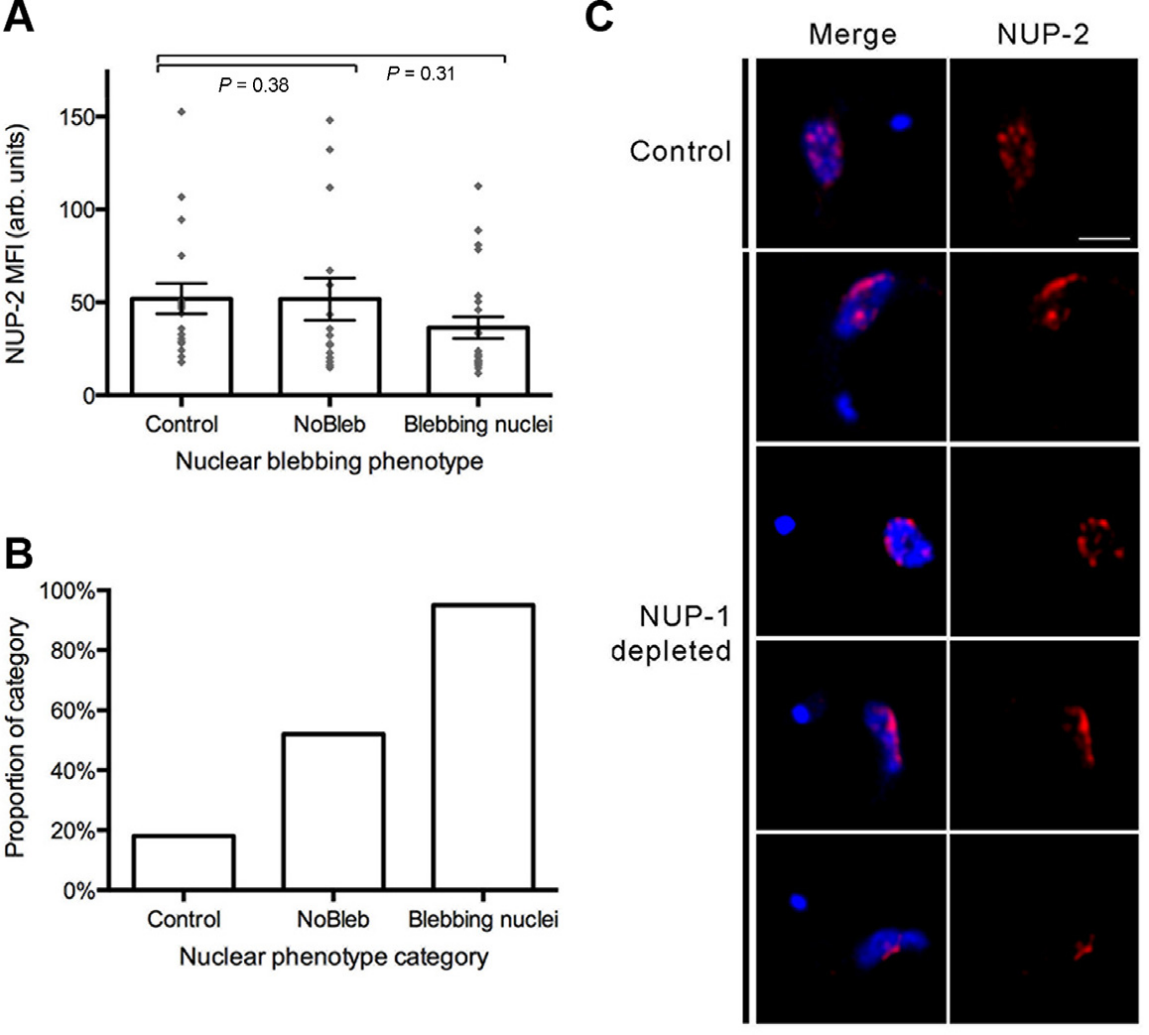
NUP-1 has a reciprocal role in supporting NUP-2. NUP-2 was examined using antibodies against a C-terminal epitope tag, after 24 h NUP-1 RNAi induction. (**A**) The intensity of NUP-2 signal in the nucleus of interphase cells across NUP-1 depleted and control populations, as determined from wide field images. Control denotes cells from and uninduced culture. The other categories represent cells from the same induced culture. Nuclear protrusions greater than 20% of the longest diameter of that nucleus (not including the protrusion) were counted as blebs. Error bars denote SEM for between 15 and 23 measurements. Statistical significance was tested using the non-parametric Mann–Whitney U-test. (**B**) The proportion of cells where NUP-2 is misarranged in blebbing and non blebbing (NoBleb) nuclei across a NUP-1 depleted population, and in an uninduced (control) population. In each category ∼30 cells were scored for NUP-2 arrangement by confocal microscopy. The presence of one or more gaps greater than 0.7 μm between NUP-2 puncta was used as a cut-off for NUP-2 misarrangement. (**C**) Representative images showing abnormal NUP-2 localization in the nuclei of NUP-1 depleted cells. Images are from deconvolved confocal z-stacks, and all are at the same magnification, scale bar denotes 2 μm.

In NUP-2 silenced cells, the intensity of NUP-1 fluorescence was diminished and inversely correlated with the severity of nuclear blebbing (Figure 8). Furthermore, NUP-1 was mislocalized in blebbed nuclei in NUP-2 knockdown cells. RNAi against NUP-1 also led to a significant reduction in NUP-2 fluorescence intensity in blebbing nuclei (Figure 9), and NUP-2 was likewise mislocalized in NUP-1 depleted cells. Notably, this impact includes cells lacking observable nuclear blebs, though the phenotype was, as expected, more prominent within blebbed nuclei. Together, these data indicate codependence between NUP-1 and NUP-2 for nuclear peripheral localisation, and together with evidence for a physical interaction (*albeit* perhaps indirect) suggests a level of contact and functionality.

### Increased DNA damage is associated with NUP-2-depletion

In mammals, unprocessed or truncated lamin A increases the frequency of double-stranded breaks (DSBs), as monitored by the marker γ-H2AX and increased sensitivity to DNA damaging agents (14). DNA rearrangements are of particular importance in trypanosomes for VSG switching and therefore antigenic variation (34).

**Figure 10.**
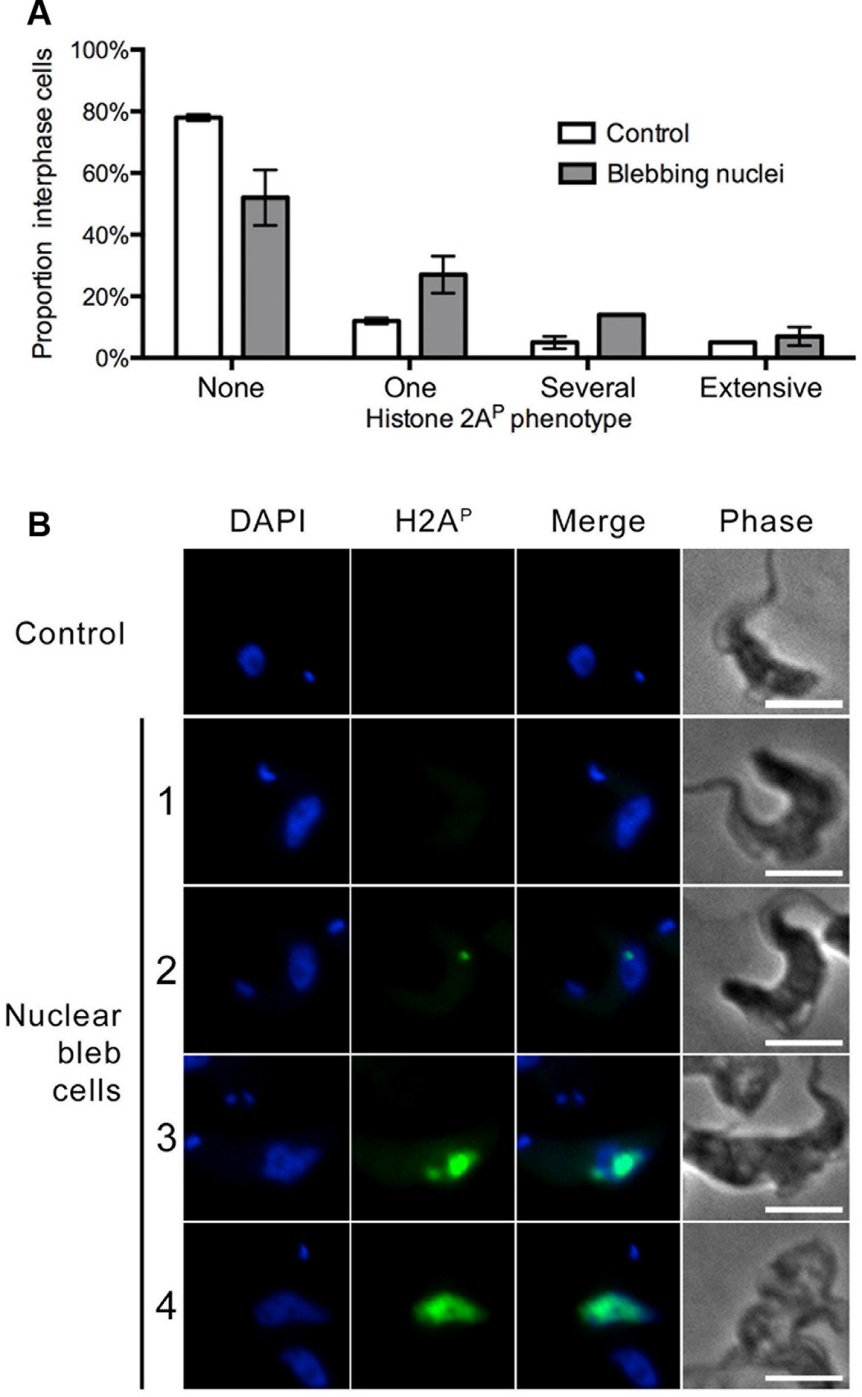
Double strand DNA breaks are increased in bleb nuclei of NUP-2 knockdown cells. (**A**) The proportion of cells scored for four Histone 2A^P^ (H2A^P^) phenotypes in uninduced (control) and NUP-2 depleted cells. H2A^P^ phenotypes were categorized according to the extent and number of puncta of H2A^P^ stain: No discernible stain was categorized as ‘no response’. A single H2A^P^ punctum visible in the nucleus was categorized as ‘one’. Cells that had more than one punctum of H2A^P^ stain in their nucleus were categorized as ‘several’. Finally, where the H2A^P^ stain extended across more than 75% of the DAPI stained nucleus, it was scored as ‘extensive’. Error bars denote SEM for three biological replicates of more than 50 cells each. (**B**) Wide-field images of representative cells from each scored category of H2A^P^ stain. Images are of interphase cells from uninduced culture (control) and of cells that have nuclear blebs from NUP-2 depleted culture. Scale bar denotes 5 μm.

A γH2A-like marker (H2A^P^) reports on DSBs in trypanosomes (67); the antibody revealed increased H2A^P^ reactivity in blebbed interphase nuclei from NUP-2 depleted cells compared to interphase nuclei from uninduced cells (Figure 10). This change was highly significant (*P* < 0.0001, Pearson's chi-squared test for pooled replicates, n > 150 cells in total for each condition). However, when all cells from NUP-2 depleted cultures were considered, no signiifcant difference was apparent (Supplementary Figure S7). Thus, an increase in DSBs is restricted to blebbed nuclei, i.e. cells with the most severe suppression of NUP-2, and which may therefore suggest that DSBs arise as a consequence of disrupted nuclear structure and not directly from silencing of NUP-2 (Figure 4E).

### NUP-2 suppression misregulates the entire VSG ES

NUP-2 likely interacts with chromatin at some level as it locates at the nuclear periphery and knockdown leads to telomere positional effects. While this interaction may be indirect and mediated *via* connections through additional factors, we asked if NUP-2 is important to the control of telomere-proximal gene transcription, as previously reported for NUP-1 (25).

**Figure 11.**
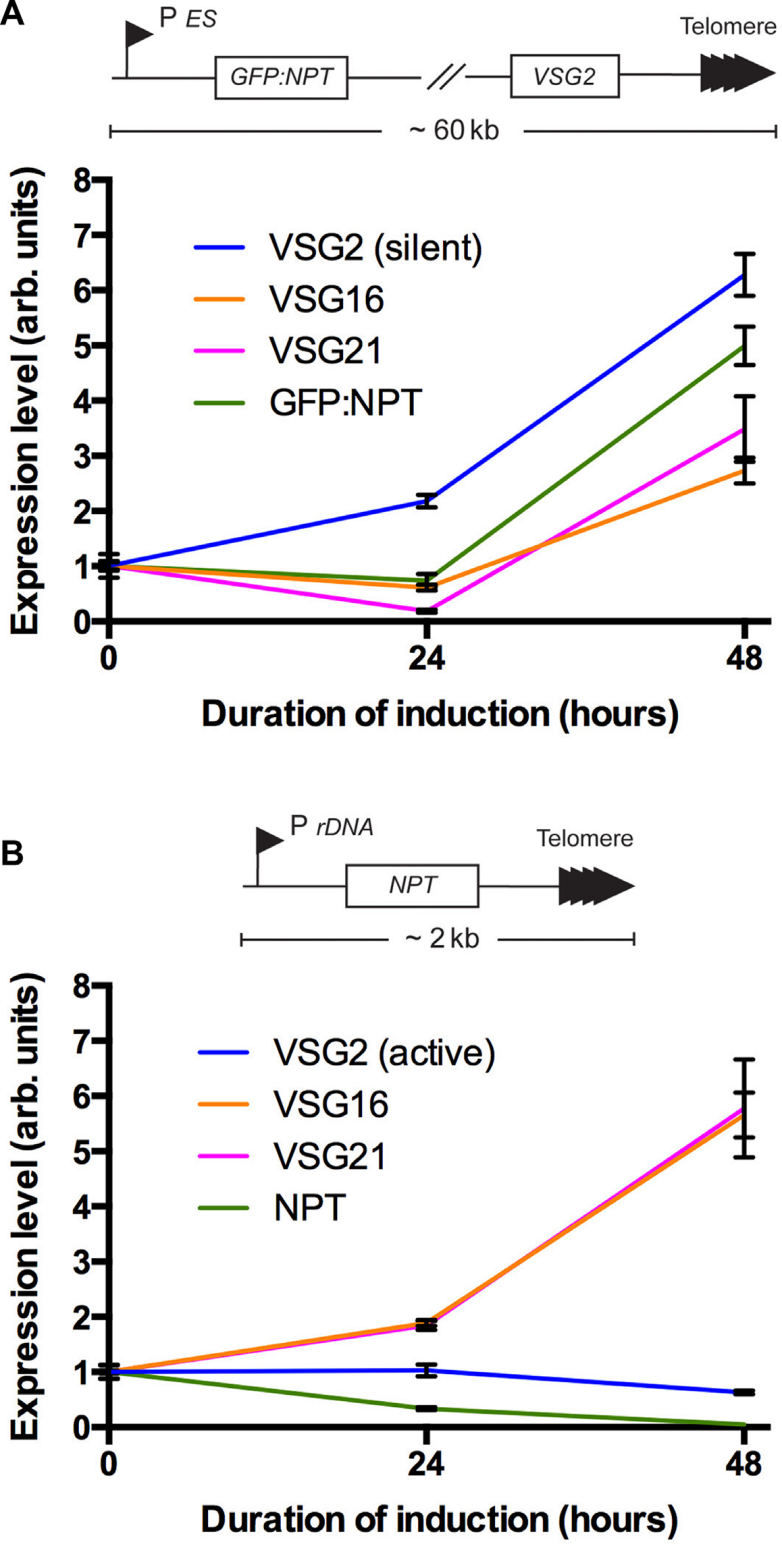
Depletion of NUP-2 specifically de-represses silent VSG expression sites, but not all telomeric genes. (**A**, top) NUP-2 RNAi was induced in cells with a *GFP:NPT* reporter downstream of the repressed *VSG2* ES promoter. (**A**, bottom) Expression of *VSG2*, several other inactive *VSGs*, and the reporter increased, as determined by qRT-PCR normalized to *PFR*. The data shown are from a single clone, representative of three independent clones. Error bars represent SEM for three technical replicates. (**B**, top) NUP-2 RNAi was induced in a cell line with a *NPT* reporter ∼2 kbp upstream of a *de novo* telomere. (**B**, bottom) Expression of inactive *VSGs* increased, while in the majority of clones the reporter and the active *VSG2* did not, as determined by qRT-PCR normalized to PFR. The data shown is from a single clone, representative of at least three independent clones. Error bars represent SEM for three technical replicates.

The *VSG* expression sites (ESs) comprise an RNA polymerase I promoter driving a polycistronic transcription unit, at the distal telomere-proximal end of which is the *VSG* gene. We asked whether *VSG* expression is affected by loss of NUP-2, whether this impact is specific to ESs, if it is part of a more generic telomeric effect, and if any effects are limited to *VSG* genes or encompass the entire ES. We used previously validated qRT-PCR profiling at telomeric sites (25), and specifically neomycin phosphotransferase (*NPT*) at a *de novo* telomeric site and *GFP-NPT* close to the *VSG2* ES promoter (Figure 11) (38).

NUP-2 RNAi (validated by Western blot: Supplementary Figure S8) led to a clear, specific increase in silent *VSG* mRNA levels, including *VSG16, VSG21* and *VSG2*, when this latter *VSG* was not the active *VSG* (Figure 11). However, as expected, when *VSG2* was active there was no further increase to *VSG2* mRNA levels. An *NPT* reporter at a *de novo* telomeric site was also not de-repressed, indicating that the effect is specific to *bona fide* silent ESs and not a generic telomere phenomenon, similar to that observed for NUP-1 (25). Further, the promoter-proximal *GFP-NPT* reporter was up-regulated in the vast majority of clones analysed (Figure 11A), suggesting that expression across the entire ES is affected by NUP-2, and not the result of a more local effect. Overall, this implies that, similar to NUP-1, NUP-2 has a specific role in the regulation of the normally silenced ES genes, including the *VSG* genes that are an essential element of the antigenic variation mechanism.

### The genome-wide transcriptional impact of NUP-2 is focused on Pol I transcriptional units

**Figure 12.**
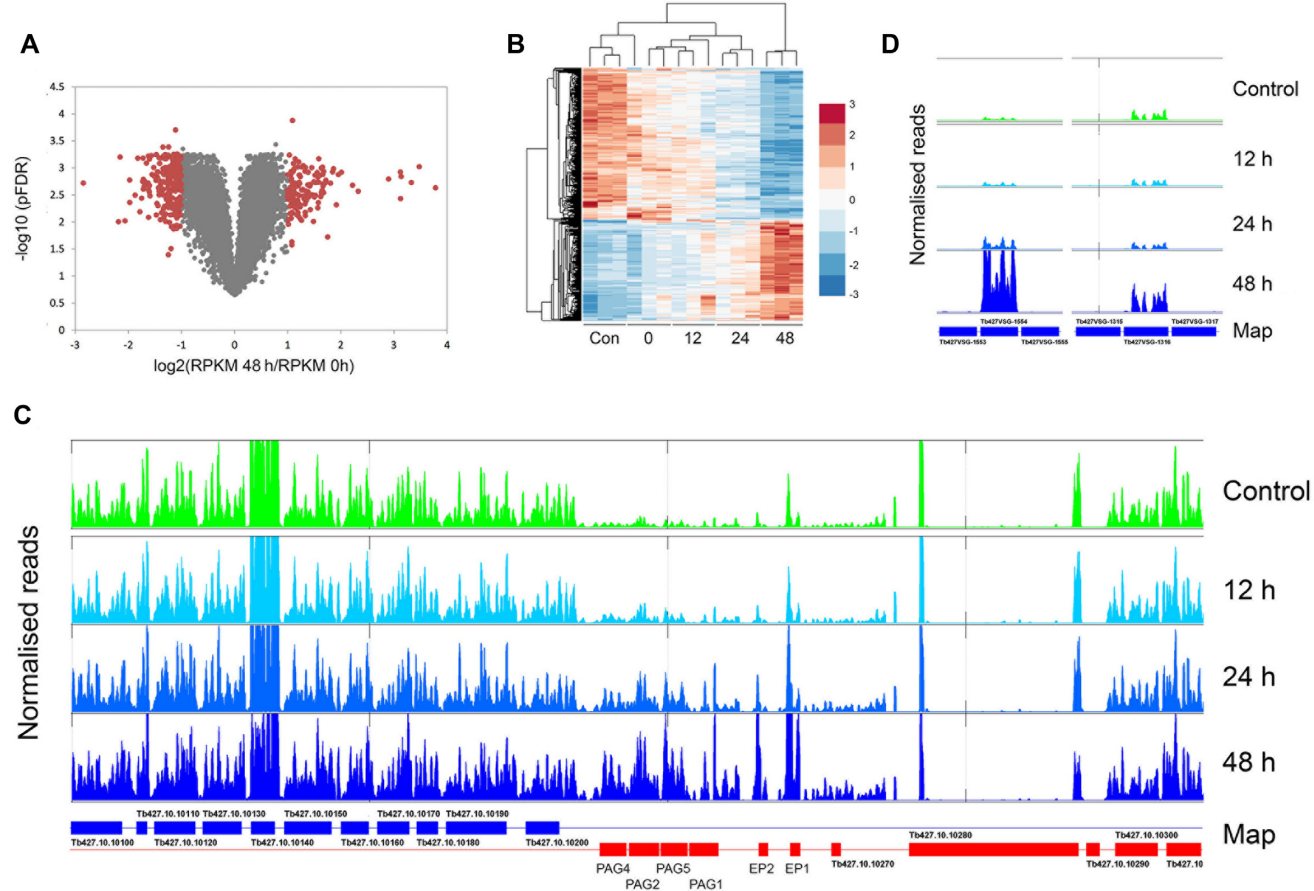
NUP-2 is required for repression of procyclin and VSG genes. (**A**) Volcano plot of RPKM values (averaged across three biological replicates) of all mapped coding sequences for 48 h induced versus non induced NUP-2 RNAi cells. Data are plotted with genes considered unaltered in grey and differentially expressed genes with positive false discovery rates (pFDR) <0.05 and absolute fold-change >2 outlined in red (see Materials and Methods for details). (**B**) Heat map that represents hierarchical clustering results of the standardized RPKM values (including all three biological replicates for each time point) of RNA profiles of the differentially expressed genes (with pFDR < 0.05 and absolute fold-change >2 at least in one time point versus the control). RPKM values are standardized so that the mean is 0 and the standard deviation is 1. (**C**) EP procyclin region from chromosome 10. Note the increased read density for the region encompassing PAG4 to Tb427.10.10270 (a probable procyclin pseudogene) and which corresponds to a Pol I transcription unit. (**D**) Example of two VSG genes that are derepressed by NUP-2 RNAi assembled as part of a pseudocontig, numbered according to Cross *et al.* (2014) (65). The flanking VSGs are not transcribed. The full data set, together with accession numbers of the significant affected VSG genes are provided in the Supplementary Data archive.

Knockdown of NUP-1 led to a significant loss of repression of Pol I transcriptional units, and specifically VSG and procyclin (25), consistent with a loss of heterochromatinization. We chose to carry out RNAseq following NUP-2 knockdown, sampling at 12, 24 and 48 h post-induction of RNAi, with three replicates at each time point, in order that we could identify with confidence differentially regulated genes by mapping reads to the Lister 427 genome (Supplementary Table S1). A relatively small number of transcripts were altered, with only 142 upregulated 2-fold or more at 48 h and over 200 downregulated at this timepoint (Figure 12, Supplementary Table S2).

Significantly, the procyclin loci on both chromosomes 6 and 10 were upregulated most strongly, with GPEET2 upregulated 14-fold and EP1 over 10-fold at 48 h post-induction. The extent of this alteration was revealed when the RNAseq reads were mapped and plotted onto the relevant region of the genome and were restricted to the procyclin polycistron only; significantly the number of mapped reads increased with duration of NUP-2 knockdown (Figure 12, EP locus). Increased RNA abundance for example did not extend into Tb427.10.10280, a predicted microtubule-binding protein and the coding sequence adjacent to the Pol I-transcribed procyclin locus. At 24 h post-induction, up-regulation was restricted to genes within the procyclin locus as well as a Zn^2+^-finger protein ZC3H45 and a few hypothetical proteins. Whilst Zn finger proteins are important for control of RNA levels and potentially in differentiation, no connection between ZC3H45 and procyclin expression has been previously reported. The more extensive downregulated cohort contains a large number of ribosomal proteins, and significantly two nuclear proteins: Tb427.08.760 (Nopp44/46), and Tb427.08.730, a predicted nucleolar RNA-binding protein upregulated at 24 h. This suggests a possible impact in the nucleolus and/or translational machinery, and which may reflect a rather more extensive loss of nuclear integrity.

We also analyzed the predicted VSG repertoire by creating a concatenated VSG pseudocontig and mapping unique reads to this sequence subset (64, Supp data archive 1). Of over 2500 predicted VSGs in the 427 genome, very few were altered significantly (using a cutoff read depth of 0.2). TbVSG11 was extremely highly expressed, and represents the active VSG. Most of the affected VSGs also exhibit evidence for low-level transcription in control and parental cells, suggesting they are associated with an expression site, and consistent with a model where NUP-2 knockdown leads to derepression at telomeric sites, allowing increased VSG transcription; moreover, the number of reads mapped to these VSGs also increased with longer silencing times. Significantly, VSGs 397, 531, 653 and 1954 are present at metacyclic expression sites and account for four of the five known mVSGs in 427. Given the low level expression of most of these VSGs in the parental cells, these data suggest that NUP-2 knockdown has relieved the normal silencing associated with all but one of the expression-competent VSG genes and is also consistent with the impact on the VSG reporters detected by qRT-PCR (Figure 11).

### NUP-2 loss does not lead to a detectable increase in VSG switch rate

**Figure 13.**
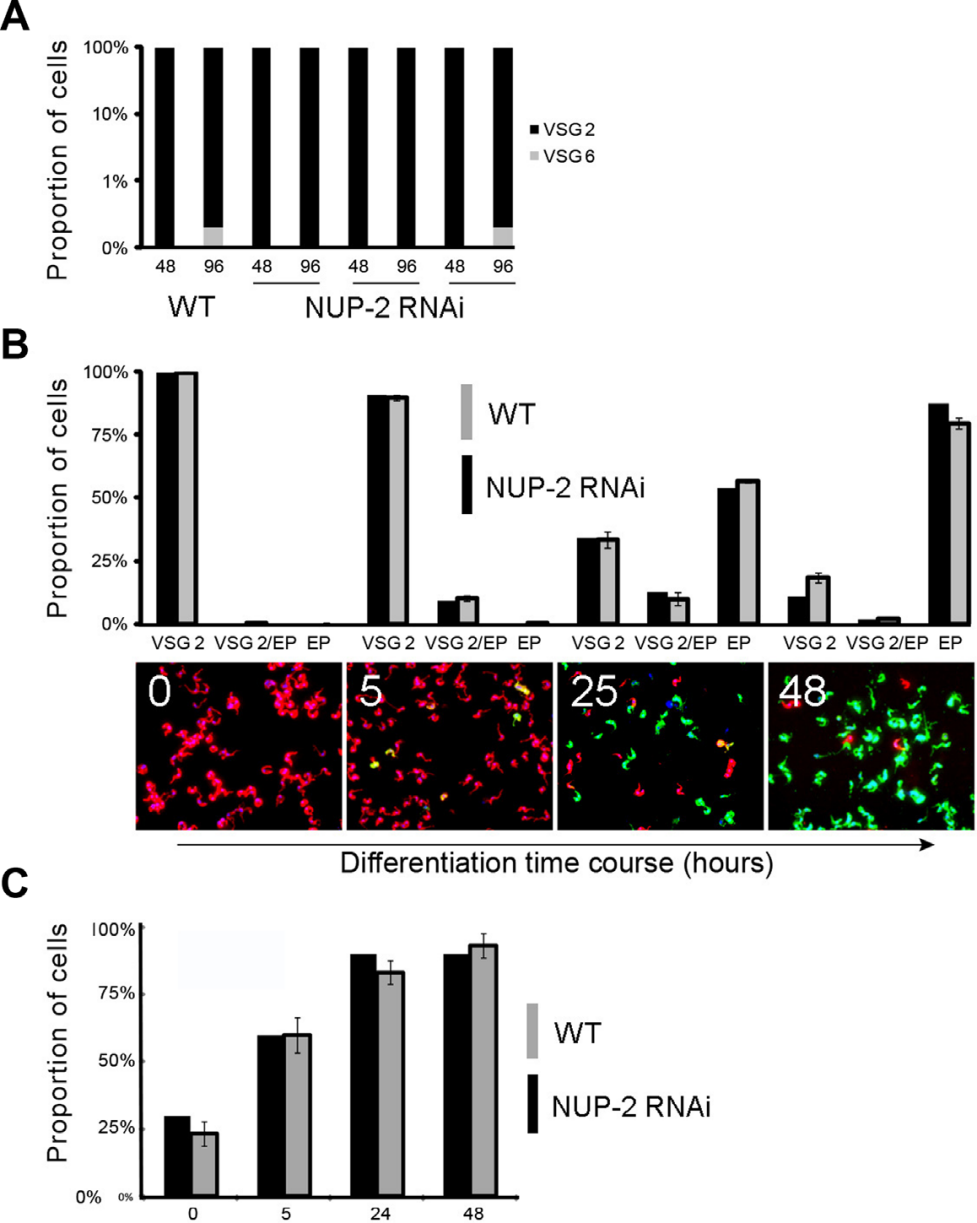
NUP-2 suppression has no detectable impact on VSG switching, differentiation or active expression site repositioning. (**A**) The proportion of cells expressing VSG2 protein at the cell surface as opposed to VSG6, in wild-type cells and in three separate NUP-2 RNAi clones at two RNAi induction time-points (48 and 96 h). VSG type was assayed by immunofluorescence microscopy. Data represents 500 cells for each analysis. X-axis numbers represent duration of induction in hours. (**B**) The proportion of cells expressing markers for BSF (VSG2) and PCF (EP procyclin, abbreviated to EP in the figure), at several time-points during differentiation. Wide field microscopy images at the differentiation time-points are shown below the histograms as representative images, where white numbers represent hours post RNAi induction. Nuclei and kinetoplasts are visualized by DAPI and colored blue. VSG2 and EP procyclin were detected by antibodies and colored red and green respectively. (**C**) Proportion of cells in which the active ES is located at the nuclear periphery at several time points during differentiation from BSF to PCF. X-axis numbers represent duration of induction in hours. Error bars denote SEM for three NUP-2 RNAi clones, 10 cells were scored for each condition and clone.

The role of NUP-2 in silent *VSG* mRNA repression and impact on NUP-1 location suggested that loss of NUP-2 may also increase the *VSG* switching rate (25). We assayed for the presence of VSG6 protein at the cell surface in a cell line where VSG2 was initially active. However, ablation of NUP-2 did not lead to increased frequency of VSG6 expression above the level in control populations (Figure 13A), distinct from NUP-1 where a similar procedure resulted in increased VSG6 expression and suggested increased switching (25). During differentiation from BSF to the insect-infective PCF, the active ES moves to the nuclear periphery (62). We observed no overall effect of NUP-2 RNAi on BSF to PCF differentiation (Figure 13B). Furthermore, in contrast to loss of NUP-1, ablation of NUP-2 did not appear to impair active ES repositioning to the nuclear periphery (Figure 13C). Hence, these data suggest a less profound role for NUP-2 in possible VSG switching mechanisms than for NUP-1.

## DISCUSSION

A nuclear lamina is a major feature of most eukaryotic cells, and in metazoan cells is intimately involved in regulating multiple nuclear events. Lamins are broadly represented across the Eukaryotes but with alternate systems probably present in fungi, plants and trypanosomes (18,23–25). We recently described NUP-1, a very large, repetitive coiled-coil protein in trypanosomes, and suggested that NUP-1 acts as a lamin analog, representing the first component of a lamina in this lineage (25).

By affinity isolation we identified at least one new lamina component, NUP-2, through its association with NUP-1. Reciprocal affinity isolation using NUP-2 confirmed this interaction together with connections to the NPC. These data also suggest that the interaction is mediated *via* TbNup110, a component of the nuclear basket, and which provides both a plausible anchoring mechanism for the lamina as well as a possible explanation for NPC clustering in NUP-1-depleted cells. NUP-2 is restricted to trypanosomatids and related taxa, with searches failing to identify an ortholog in *N. gruberi*, a member of the heterolobosid sister taxon within the *Excavata* supergroup. The coevolutionary distribution of NUP-1 and NUP-2 suggests that the trypanosome lamina configuration is complex and likely encompasses many proteins, but has origins distinct from lamina systems in non-kinetoplastid organisms. It is however, also an ancient system, being present at the root of the kinetoplastids, and hence is not associated with parasitism, antigenic variation or other specific features of kinetoplastid pathogenesis *per se* (68).

Significantly, NUP-2 shares both a high molecular weight and coiled-coil architecture with NUP-1, though there is no evidence for sequence similarity. Unlike NUP-1, NUP-2 has no obvious repeat organization. If fully extended as a helix, the 1520 residue NUP-2 protein would be ∼230 nm long, more than 10% the diameter of the trypanosome nucleus. As NUP-1 may be ∼400 nm long (25), NUP-1 and NUP-2 can likely access much of the trypanosome nuclear volume, indicating the potential for long range influence on nuclear functions. The positioning of NUP-2 at the nuclear periphery throughout the cell cycle is reminiscent of the NUP-1 arrangement, but as these are both very large proteins our data do not presently inform on the location of the majority of the entire polypeptide, and hence the precise associations between NUP-1 and NUP-2 and the NPC remains to be characterized.

Silencing of NUP-2 affected nuclear morphology, faithful arrangement of chromatin at the nuclear periphery and epigenetic regulation. While these are all in common with NUP-1, the details of the phenotypes are quite distinct. Specifically, NUP-2 does not relocate within the nucleus to the same degree as NUP-1 at mitosis, NUP-2 depletion does not elicit a VSG switching event, nor does it result in NPC clustering, indicating that NUP-1 and NUP-2 have distinct roles, presumably the basis for their independent origins and retention throughout the kinetoplastids. The absence of a full VSG switch may, however, reflect a threshold effect, as NUP-2 knockdown does relieve the silencing at silent VSG ESs, and thus switching may be increased but remained below our detection limit. If this is the case, VSG switching is at least an order of magnitude less frequent than for NUP-1 knockdown.

NUP-1 and NUP-2 are clearly spatially distinct, with disparate localizations during mitosis, reminiscent of the A and B-type lamin networks in vertebrates (2,64). Significantly, the impact of depletion of NUP-1 or NUP-2 on the localization of the other does not correspond to nuclear deformity in precisely the same way: loss of NUP-1 led to NUP-2 disorganization in some non-blebbing as well as blebbing nuclei, whilst the reverse was not the case. This implies that NUP-1 could be the primary organizing component of the trypanosome nucleoskeleton, and the detection of substantially more NPC proteins in NUP-1 versus NUP-2 pullouts is consistent with this view. However, NUP-2 mis-arrangement was more common in blebbing nuclei in NUP-1 depleted cells, so that in both cases disorganization correlates to some extent with nuclear deformity.

The data here also further supports multiple levels of *VSG* regulation: NUP-2 is required for an apparently redundant level of regulation, i.e. suppressing RNA production from silent *VSG* ESs. However, loss of NUP-2 does not affect VSG protein switching overall, nor does it result in a transcriptional level for the *VSG* ES that approaches a fully active *VSG*, indicating the presence of additional regulatory mechanisms independent of NUP-2. Transcription of a small cohort of VSGs that are already transcribed at a very low level in uninduced cells suggests that NUP-2 impacts ES-associated VSGs and which is also supported by the presence of metacyclic ES-associated VSGs in this cohort. Significantly, the procyclin locus is also misregulated, with increased RNA levels associated exclusively with the procyclin and procyclin-associated genes. Finally, a role for NUP-2 in preventing DSBs may be linked to its function in *VSG* regulation (34), but this may also be a consequence of the loss of nuclear structural organization and mechanical stability of the envelope.

The identification of a second trypanosomal nucleoskeletal protein provides further evidence that a structure similar to the nuclear lamina is a common or even near-universal feature of eukaryotic cells, but the absence of detectable sequence similarity of either of the NUPs with lamins suggests that a nucleoskeleton subtending similar functions has arisen by convergent evolution in the highly divergent kinetoplastids and many other eukaryotic lineages (16). Resolving the evolutionary history of the lamina is a clearly important aspect of understanding the origins of the nucleus and gene regulation mechanisms and adds further evolutionary divergence to events at the trypanosome nuclear periphery.

## Supplementary Material

SUPPLEMENTARY DATA
