# Supplementary material for: Co-dependence between trypanosome nuclear lamina components in nuclear stability and control of gene expression
Source: Nucleic Acids Res. 2016 Sep 12;44(22):10554–70. doi: 10.1093/nar/gkw751 (PMC5159534; doi:10.1093/nar/gkw751)
Supplement: SUPPLEMENTARY DATA [file supp_44_22_10554__index.html]

Co-dependence between trypanosome nuclear lamina components in nuclear stability and control of gene expression — Co-dependence between trypanosome nuclear lamina components in nuclear stability and control of gene expression — SUPPLEMENTARY DATA 

# Co-dependence between trypanosome nuclear lamina components in nuclear stability and control of gene expression

## SUPPLEMENTARY DATA

- SUPPLEMENTARY DATA
- SUPPLEMENTARY DATA
- SUPPLEMENTARY DATA
- SUPPLEMENTARY DATA
- SUPPLEMENTARY DATA
- SUPPLEMENTARY DATA
- SUPPLEMENTARY DATA
- SUPPLEMENTARY DATA
- SUPPLEMENTARY DATA
- SUPPLEMENTARY DATA
- SUPPLEMENTARY DATA
- SUPPLEMENTARY DATA
