# Supplementary material for: Co-dependence between trypanosome nuclear lamina components in nuclear stability and control of gene expression
Source: Nucleic Acids Res. 2016 Sep 12;44(22):10554–70. doi: 10.1093/nar/gkw751 (PMC5159534; doi:10.1093/nar/gkw751)
Supplement: SUPPLEMENTARY DATA [file supp_gkw751_nar-01100-x-2016-File015.docx]

**Supplementary data for:**

**Co-dependence between trypanosome nuclear lamina components in nuclear stability and control of gene expression**

Luke Maishman^1^, Samson Obado^2^, Sam Alsford^3^, Jean Bart^4^, Wei-Ming Chen^5^, Alexander Ratushney^5^, Miguel Navarro^4^, David Horn^1^, John Aitcheson^5^, Brian T. Chait^2^, Michael P. Rout^2^ and Mark C. Field^1*^

^1^School of Life Sciences, University of Dundee, Dundee, Scotland, DD1 5EH, UK, ^2^The Rockefeller University, 1230 York Avenue, New York, NY 10021, USA, ^3^London School of Hygiene and Tropical Medicine, Keppel Street, London WC1E 7HT, UK, ^4^Instituto de Parasitología y Biomedicina López-Neyra, Consejo Superior de Investigaciones Cientificas, 18100 Granada, España and ^5^Seattle Biomedical Research Institute, Seattle, Washington, USA.

**Supplementary figure legends**

**Figure S1: Identity and similarity between NUP-2 orthologs.** Scores for identity and similarity between NUP-2 orthologs across trypanosomatids were calculated by SIAS, the sequence identity and similarity tool (<http://imed.med.ucm.es/Tools/sias.html>) using standard settings. Species are ordered by similarity to *T. brucei* NUP-2. Panel A: Graphical representation of the percent similarity and identity between TbNUP-2 and orthologs in indicated genomes. Panel B: Matrix showing the percent similarity and identity between all NUP-2 ortholog predicted protein sequences.

**Figure S2: NLS is conserved across NUP-2 orthologs.** NLSs predicted by the NLS mapper tool are highlighted in purple in a Jalview representation of NUP-2 predicted proteins aligned at this region. Dark purple indicates a score greater than 5, light purple a score of 5 or less. Panel A: A monopartite NLS is located at the central region of all NUP-2 orthologs identified and the position is highly conserved between orthologs. Panel B: A second monopartite NLS pattern was predicted in *Leishmania* orthologs, as well as in *T. cruzi*, *T. carassi* and *P. serpens* at similar positions.

**Figure S3: NUP-2 has similar localization in procyclic and bloodstream forms.** Localization of NUP-2 to a punctate distribution at the nuclear periphery in interphase cells in both BSF and PCF cells. Images are wide-field IFA microscopy. NUP-2 was visualized using a C-terminal *in situ* three times HA epitope tag in BSF cells and a C-terminal *in situ* GFP epitope tag in PCF cells.

**Figure S4: Validation of the TbNup98 NPC marker.** Panel A: Western blot to verify correct insertion of the TbNup98 3xHA C-terminal epitope tag. Numbers at left indicate molecular weights of coelectrophoresed markers in kilodaltons (kDa). Panel B: The TbNup98 3 x HA C-terminal epitope tag was further investigated by PCR, using primers against part of the non-repetitive N-terminus and against the 3 x HA tag. The expected length of the PCR product for full-length TbNup98, including the 3 x HA tag, is 3052 base pairs. The numbers at left indicate DNA sequence length of coelectrophoresed markers in base pairs (bp).

**Figure S5: Depletion of NUP-2 affects cell cycle progression.** Panel A: The proportion of cells in major stages in the cell cycle at various induction times. Fixed cells stained with DAPI were categorized as 1K1N, 2K1N, 2K2N, monsters (having more than 2K and/or 2N) and others that deviate from these categories. Error bars denote SEM for two biological replicates of 100 cells each at every time-point. Panel B: Example FACSgrams of NUP-2 RNAi cells, both uninduced and 24 hours post induction. Each plot represents one biological replicate of 2 x 10^5^ cells. The data were gated by DNA content as measured by propidium iodide fluorescence: R2 denotes half of the cells in G_1_ phase, R3 denotes half of the cells in G_2_M phase, R6 denotes all cells of normal DNA content (2C-4C), R4 denotes cells with an abnormally small amount of DNA (less than 2C), R5 denotes cells with an abnormally large amount of DNA (greater than 4C). Panel C (left): The proportion of cells in G_1_ and G_2_M phase at either 0 hours or 24 hours post induction, as determined by FACS analyses such as those in panel B. Error bars denote SEM for three biological replicates of 200 000 cells each at both time points. Panel C (Right): The proportion of cells having normal and abnormal amounts of DNA in uninduced and 24 hours induced culture, as determined by FACS analyses such as those in panel B. Error bars denote SEM for three biological replicates of 200 000 cells each at both time points.

**Figure S6: Loss of NUP-2 does not affect mitotic spindle formation.** Example wide-field images showing the mitotic spindle in mitotic cells stained with the KMX-1 anti-ß-tubulin antibody, in uninduced (control) and NUP-2 depleted populations. There was no obvious difference in the frequency of cells with a mitotic spindle between the two populations.

**Figure S7: H2A^P^ phenotypes across the NUP-2 depleted population.** The proportion of interphase cells scored for four H2A^P^ phenotypes in uninduced interphase cell nuclei (control) and blebbing interphase nuclei from NUP-2 depleted culture. H2A^P^ phenotypes were categorized according to the extent and number of puncta of H2A^P^ stain: No discernible stain was categorized as “none”. A single H2A^P^ punctum visible in the nucleus was categorized as “one”. Cells that had more than one punctum of H2A^P^ stain in their nucleus were categorized as “several”. Finally, where the H2A^P^ stain extended across more than 75% of the DAPI stained nucleus, location was scored as “extensive”. Error bars denote SEM for three biological replicates of more than 50 cells each.

**Figure S8: NUP-2 protein depletion by stem loop RNAi.** Panel A: The fraction of NUP-2 protein remaining after 24 hours induction of a NUP-2 2^T1^ stem loop RNAi construct, as determined by semi-quantitative western blotting. A NUP-2 C-terminal *in situ* 3 x HA epitope tag was used as a target for immunoblotting. The intensity of protein bands was measured using ImageJ. Data were normalized to ß-tubulin as a loading control. Error bars denote SEM for three biological replicates. Panel B: Representative western blot images. Images of both ß-tubulin and Ponceau S stained membranes are shown as loading controls. Migration positions of co-electrophoresed markers are shown at left in kDa.
