# Supplementary figures and images for: Co-dependence between trypanosome nuclear lamina components in nuclear stability and control of gene expression (part 1 of 2)
Source: Nucleic Acids Res. 2016 Sep 12;44(22):10554–70. doi: 10.1093/nar/gkw751 (PMC5159534; doi:10.1093/nar/gkw751)

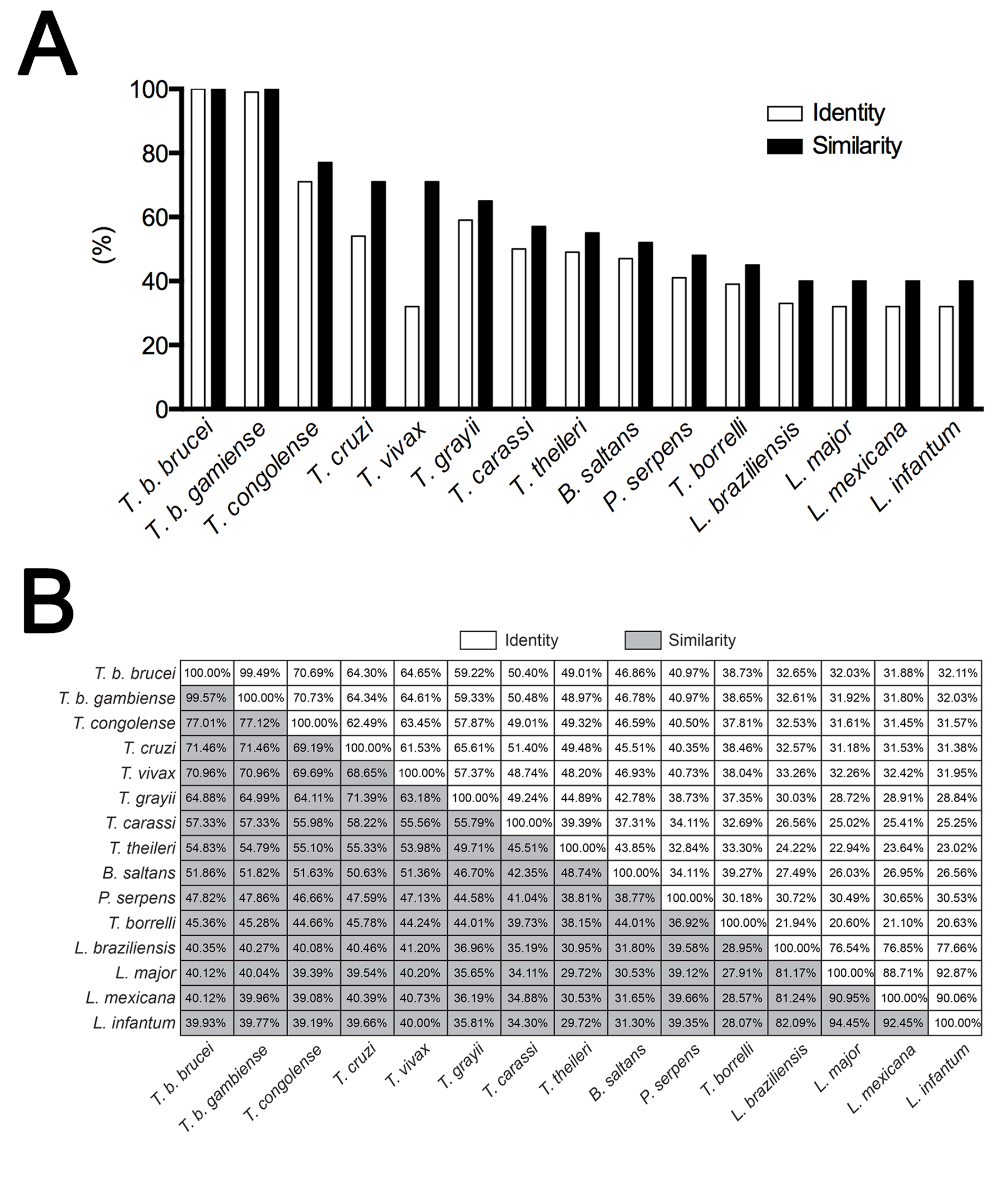

Supplement: SUPPLEMENTARY DATA [file supp_gkw751_nar-01100-x-2016-File016.png]

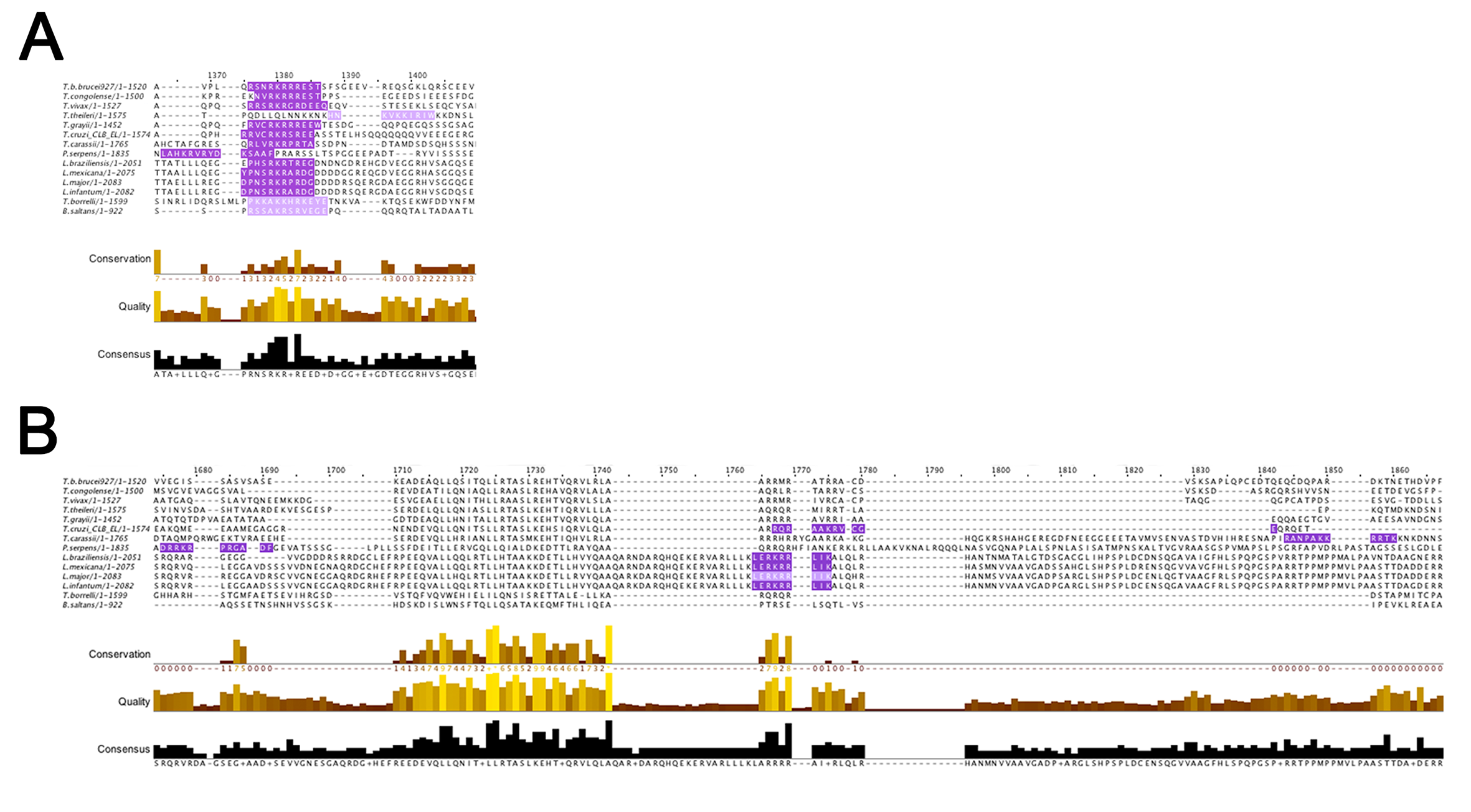

Supplement: SUPPLEMENTARY DATA [file supp_gkw751_nar-01100-x-2016-File017.png]

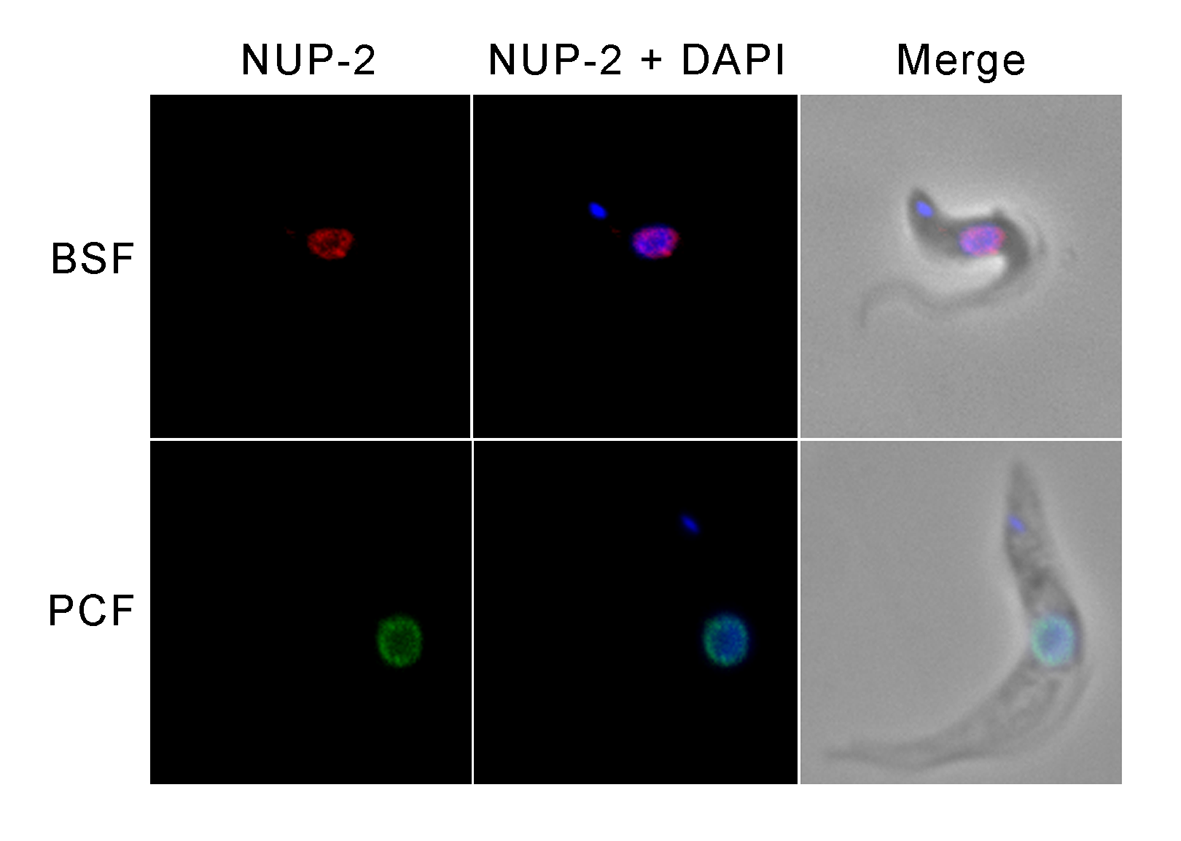

Supplement: SUPPLEMENTARY DATA [file supp_gkw751_nar-01100-x-2016-File018.png]

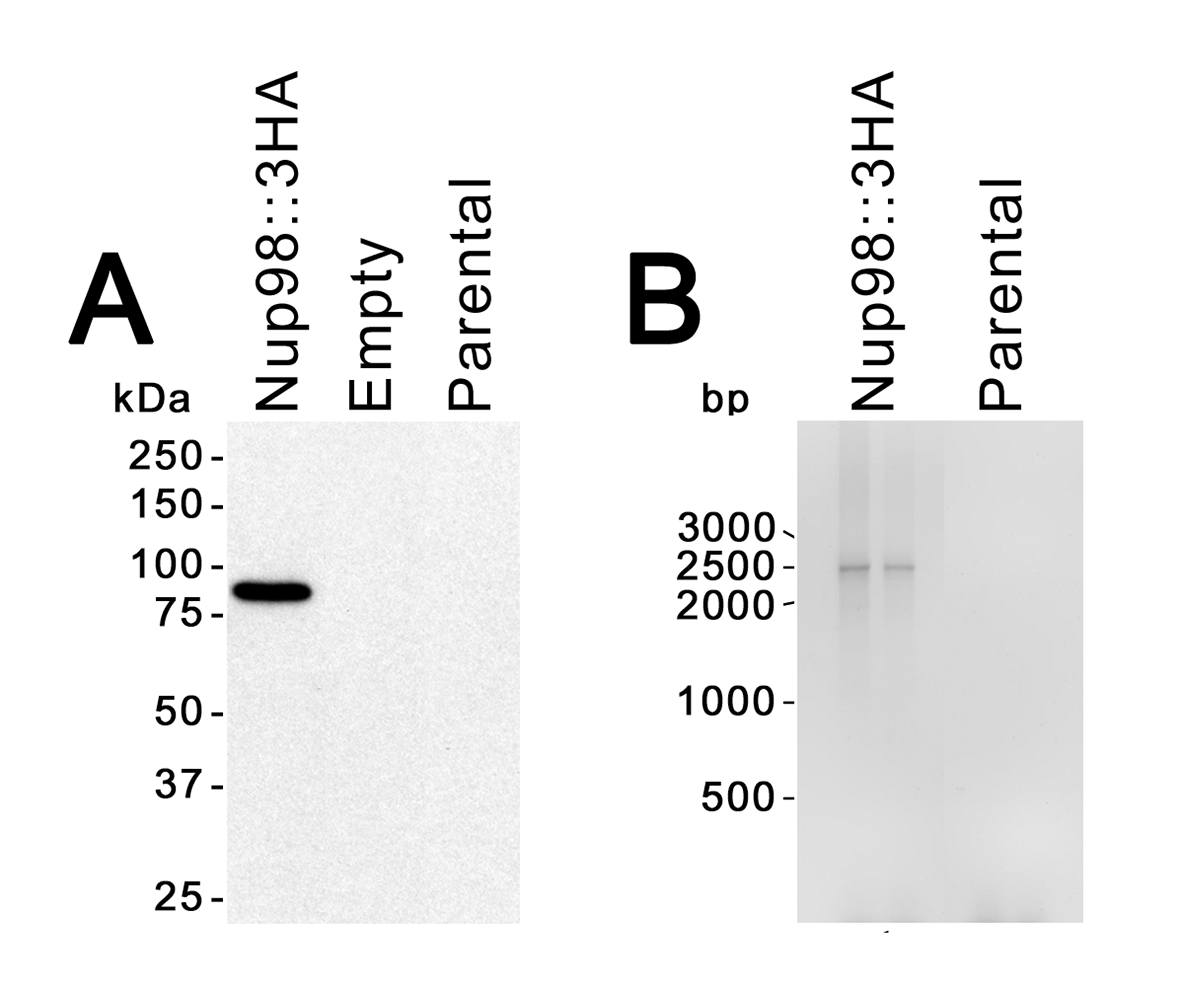

Supplement: SUPPLEMENTARY DATA [file supp_gkw751_nar-01100-x-2016-File019.png]

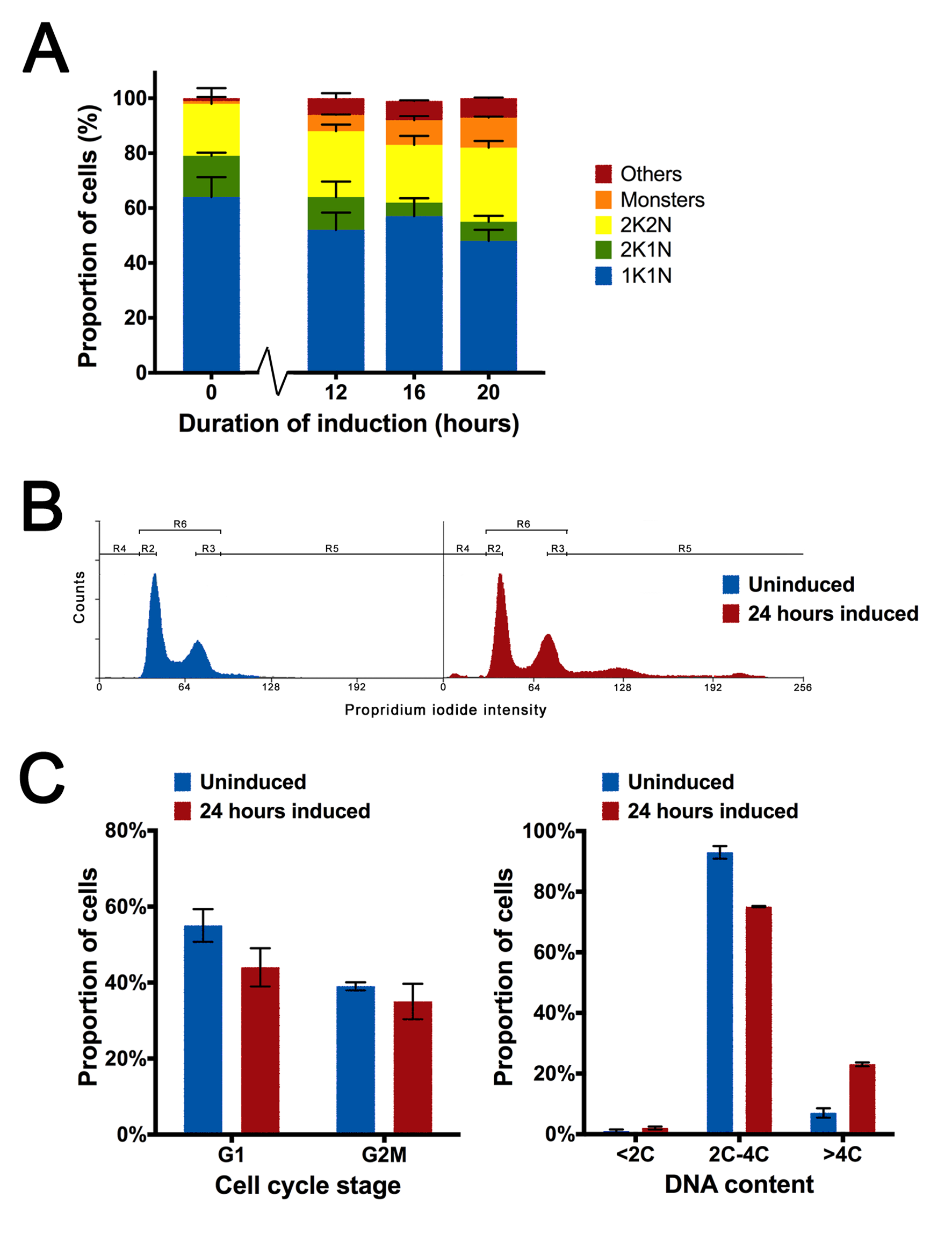

Supplement: SUPPLEMENTARY DATA [file supp_gkw751_nar-01100-x-2016-File020.png]

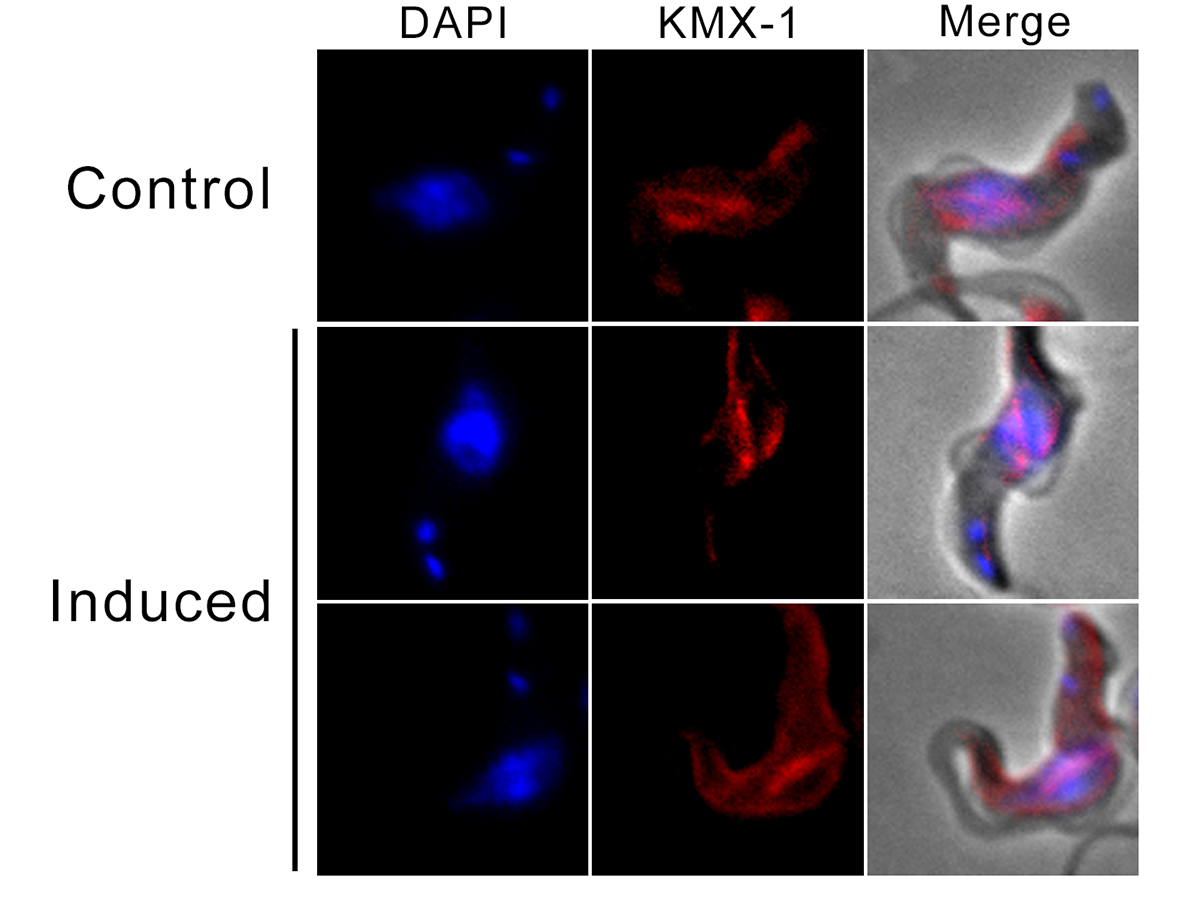

Supplement: SUPPLEMENTARY DATA [file supp_gkw751_nar-01100-x-2016-File021.png]

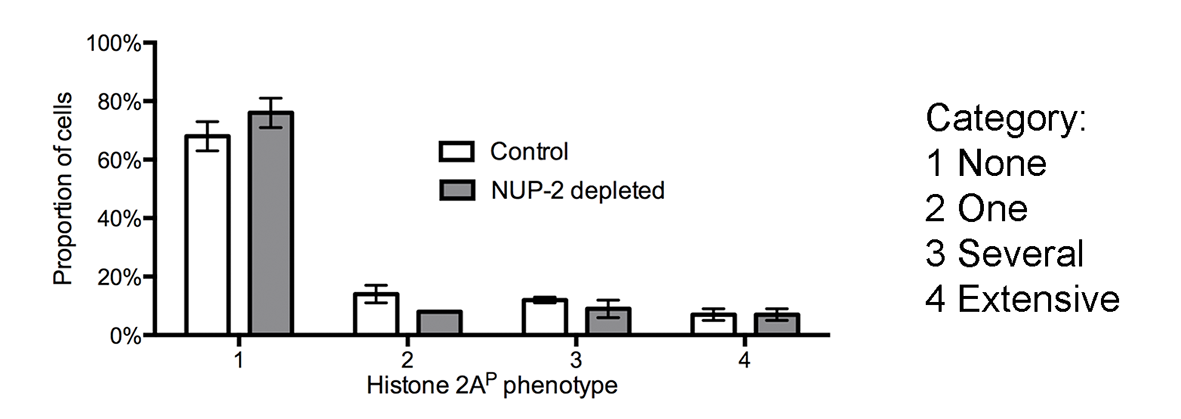

Supplement: SUPPLEMENTARY DATA [file supp_gkw751_nar-01100-x-2016-File022.png]

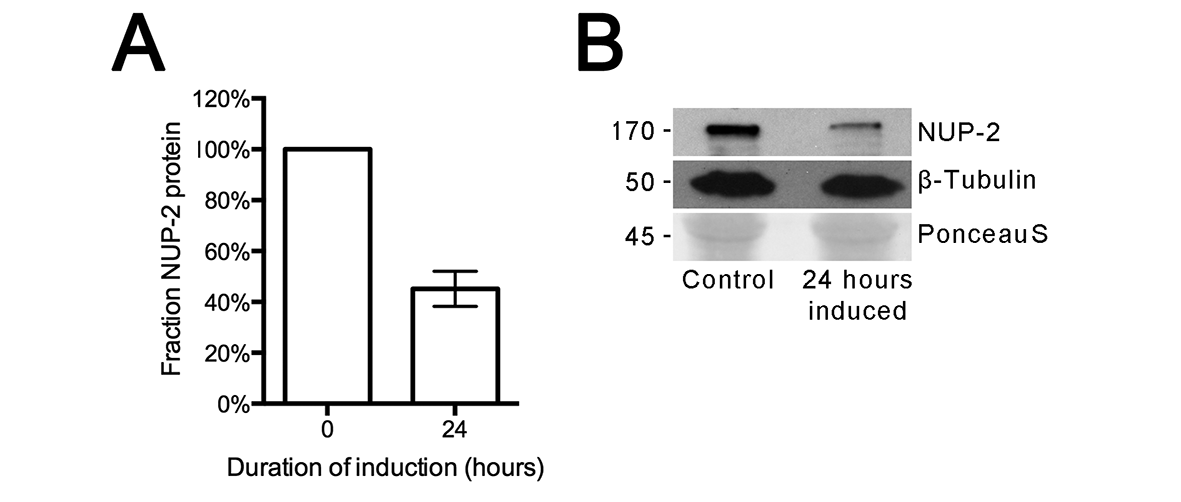

Supplement: SUPPLEMENTARY DATA [file supp_gkw751_nar-01100-x-2016-File023.png]

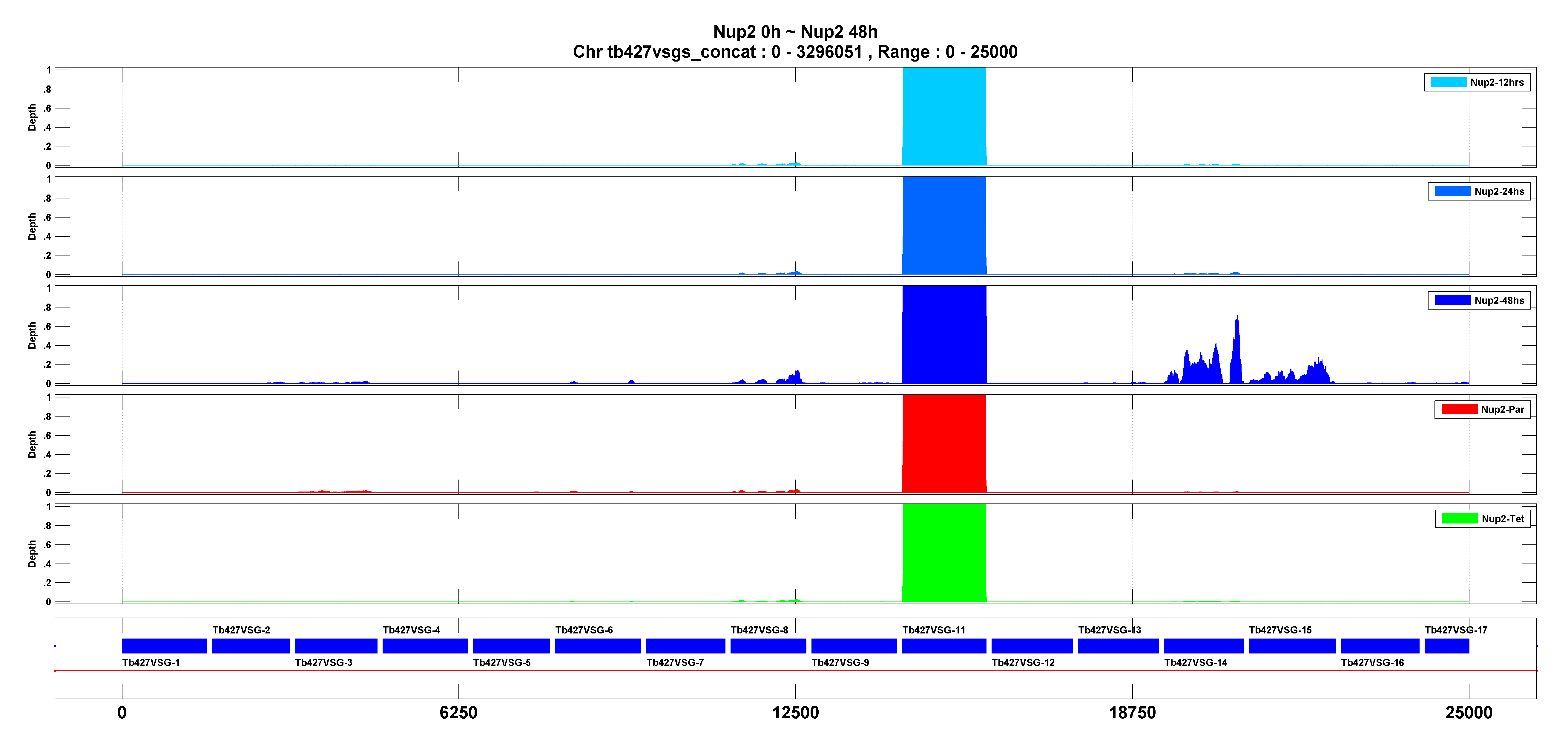

Supplement: SUPPLEMENTARY DATA [file supp_gkw751_nar-01100-x-2016-File026.zip › VSG transcriptome map/fig_tb427vsgs_concat_whole-seq_1.png]

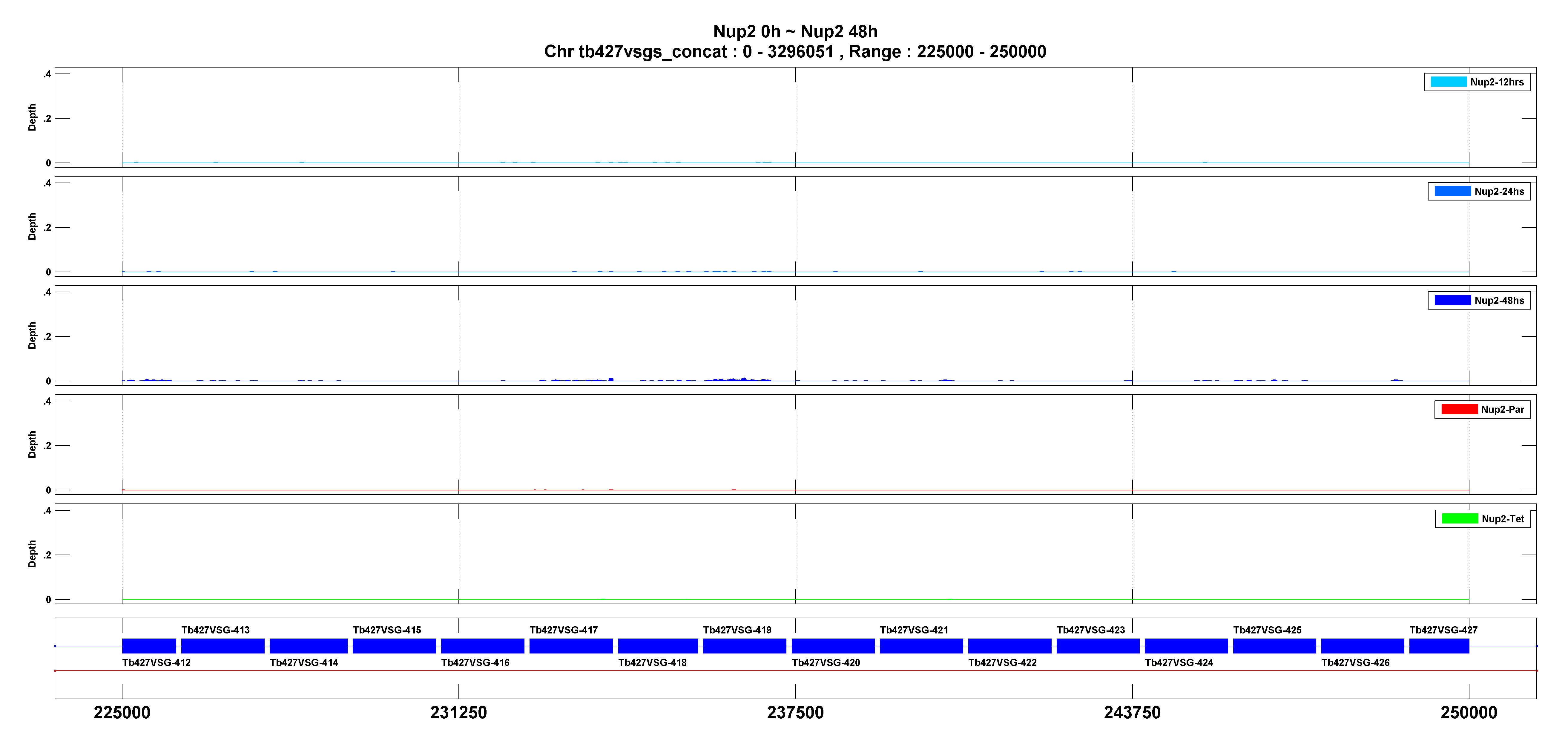

Supplement: SUPPLEMENTARY DATA [file supp_gkw751_nar-01100-x-2016-File026.zip › VSG transcriptome map/fig_tb427vsgs_concat_whole-seq_10.png]

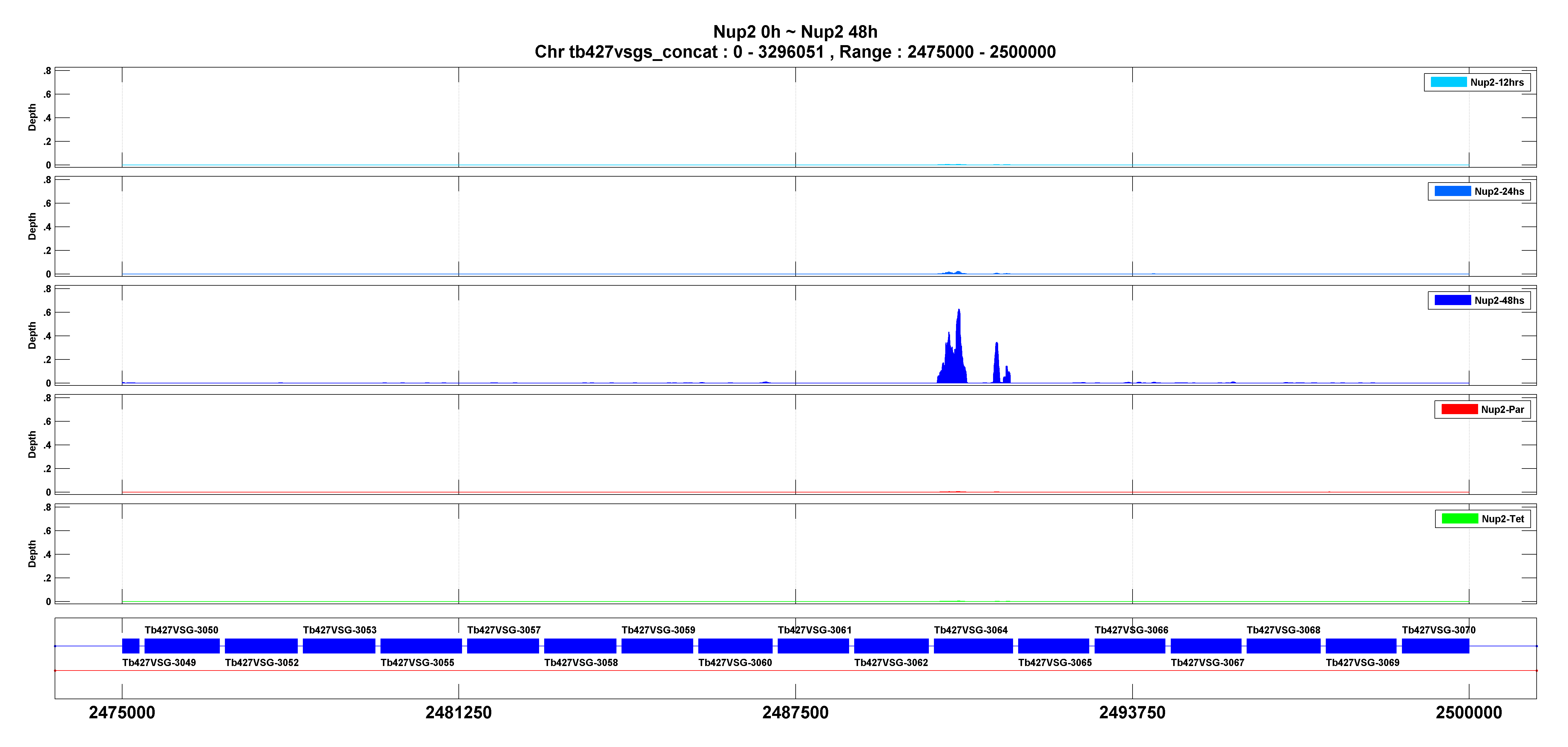

Supplement: SUPPLEMENTARY DATA [file supp_gkw751_nar-01100-x-2016-File026.zip › VSG transcriptome map/fig_tb427vsgs_concat_whole-seq_100.png]

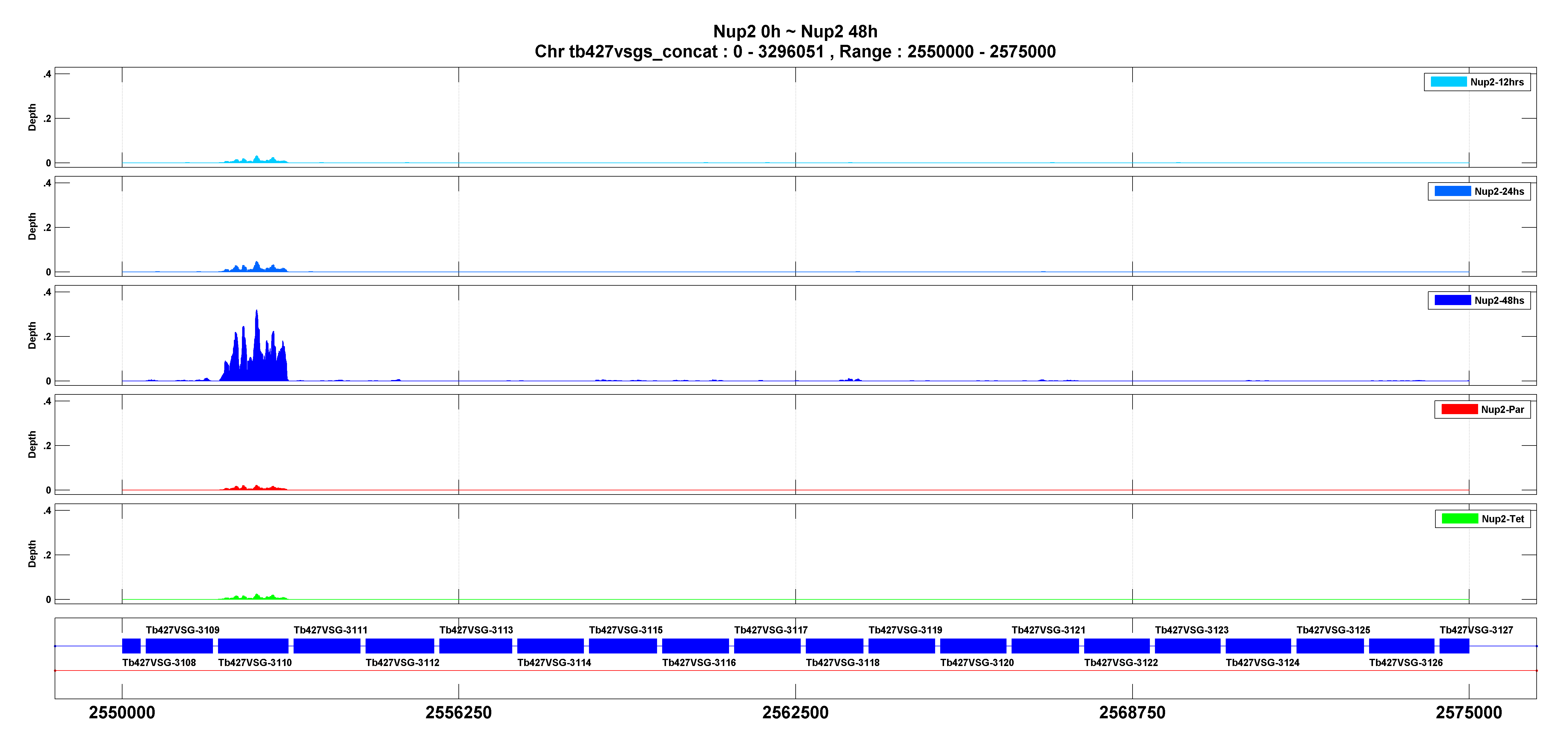

Supplement: SUPPLEMENTARY DATA [file supp_gkw751_nar-01100-x-2016-File026.zip › VSG transcriptome map/fig_tb427vsgs_concat_whole-seq_103.png]

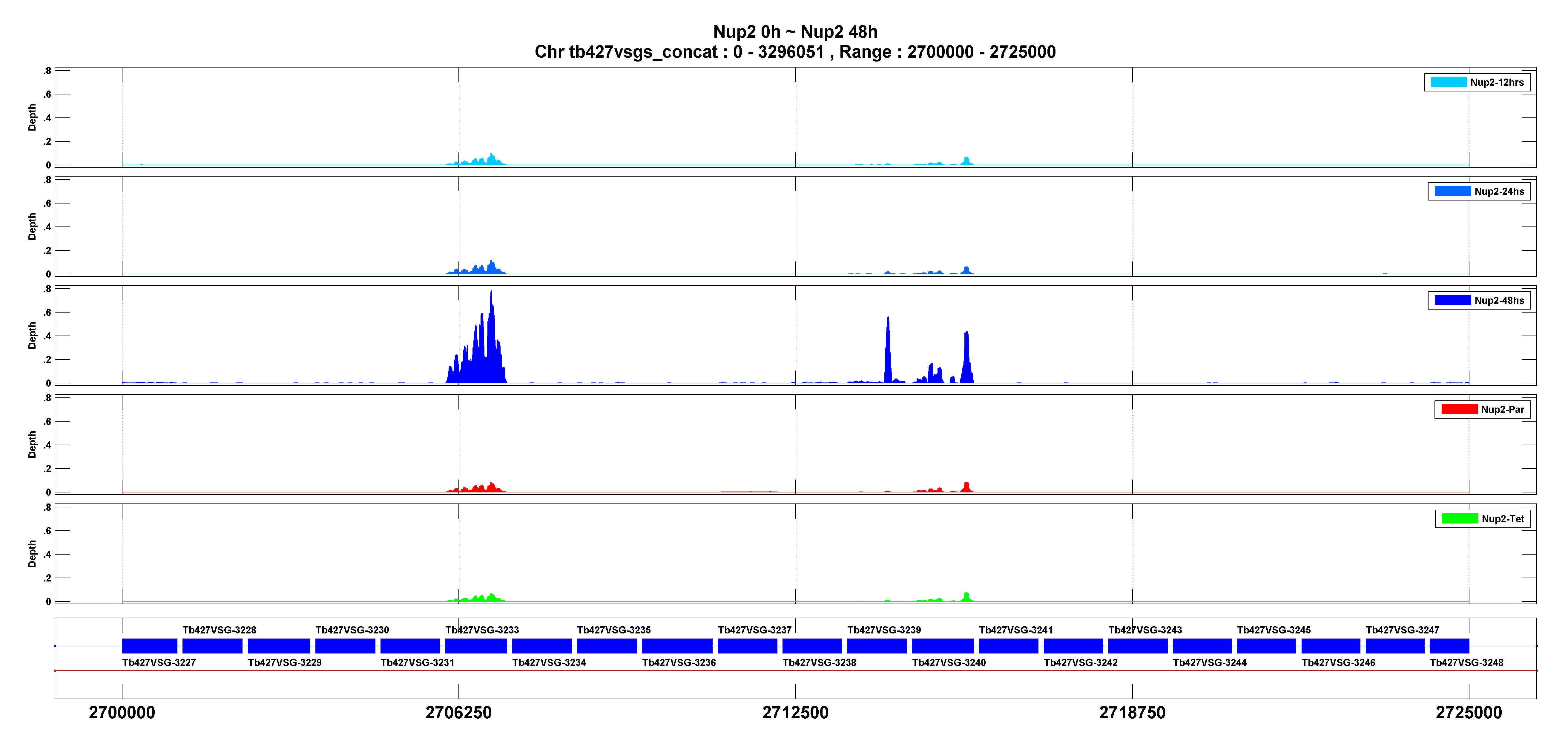

Supplement: SUPPLEMENTARY DATA [file supp_gkw751_nar-01100-x-2016-File026.zip › VSG transcriptome map/fig_tb427vsgs_concat_whole-seq_109.png]

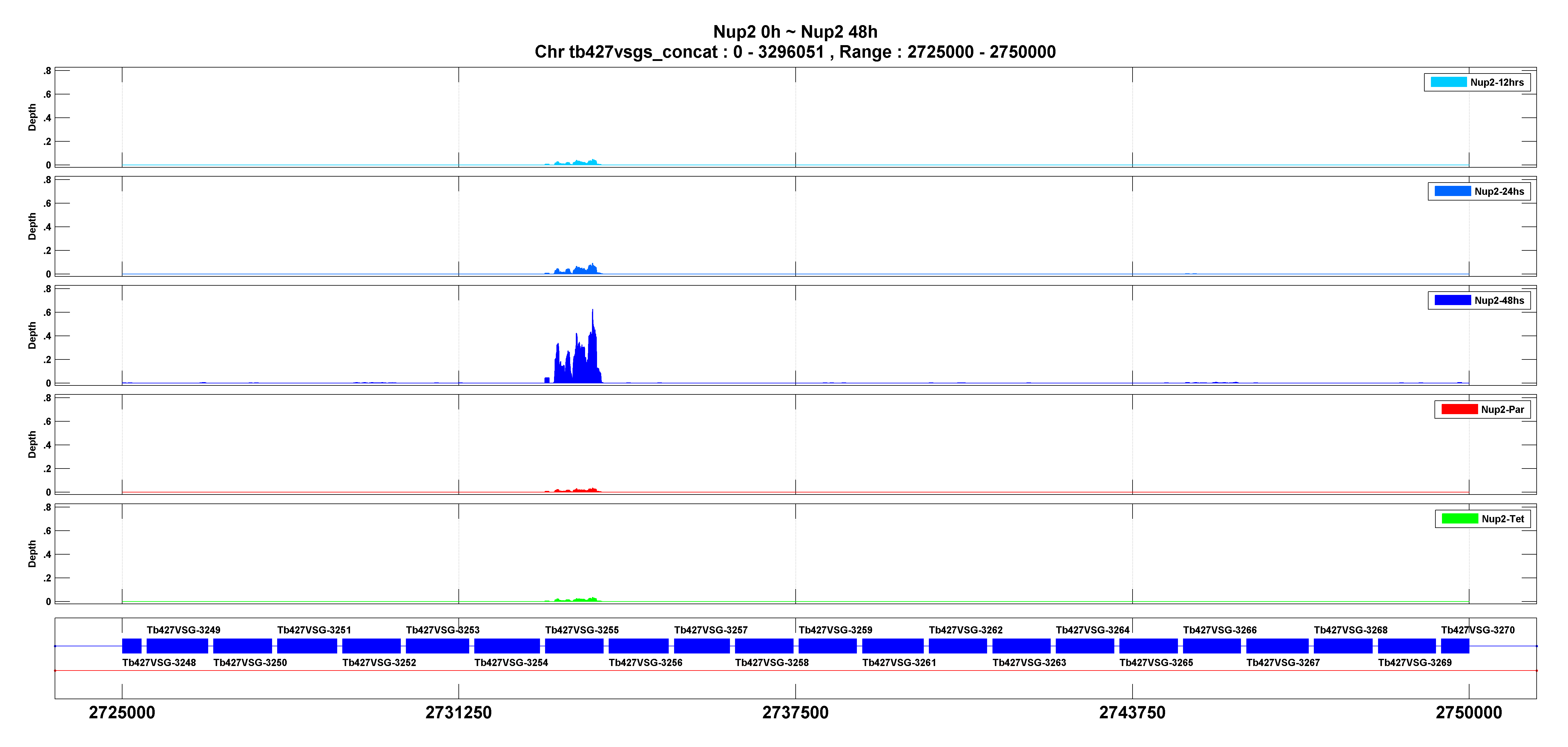

Supplement: SUPPLEMENTARY DATA [file supp_gkw751_nar-01100-x-2016-File026.zip › VSG transcriptome map/fig_tb427vsgs_concat_whole-seq_110.png]

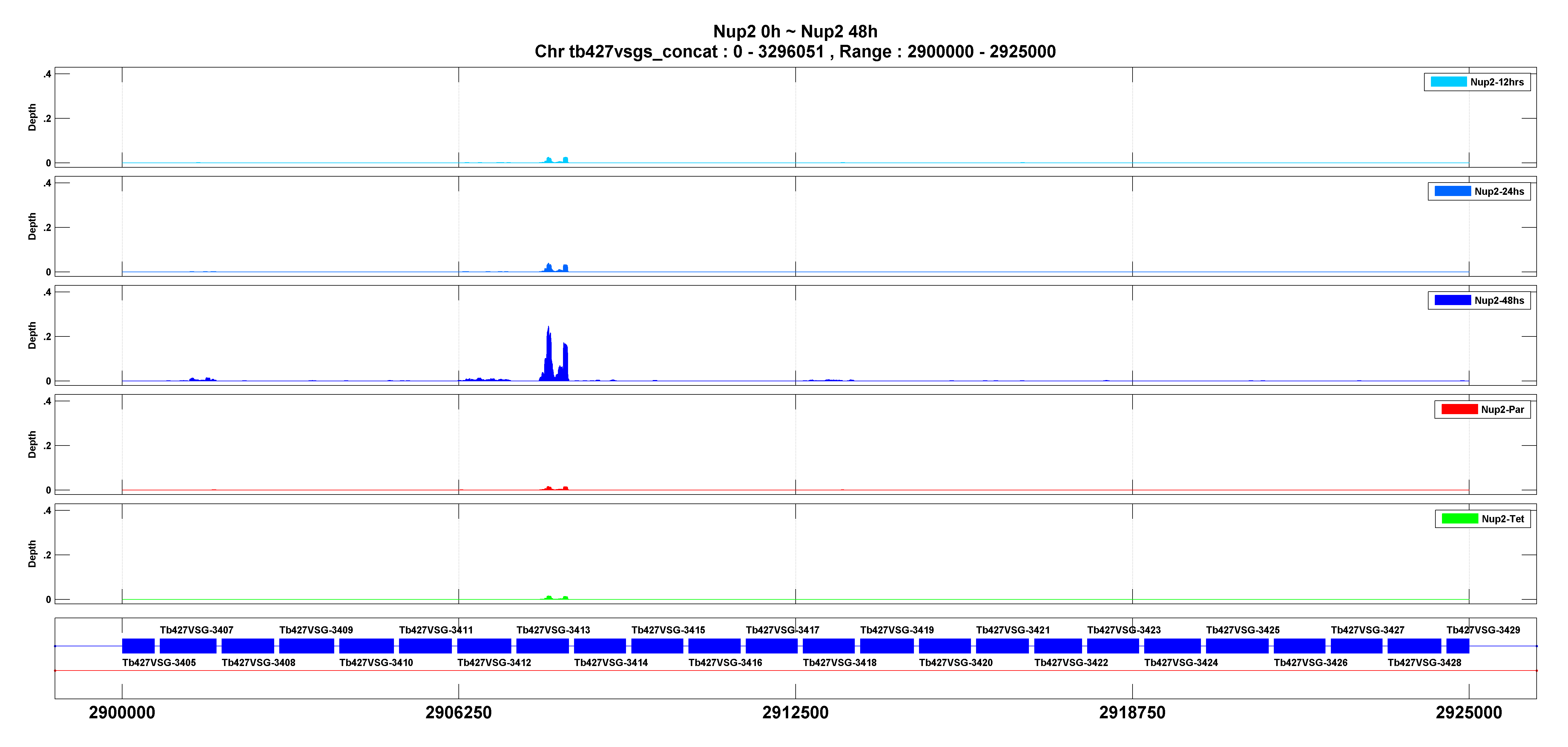

Supplement: SUPPLEMENTARY DATA [file supp_gkw751_nar-01100-x-2016-File026.zip › VSG transcriptome map/fig_tb427vsgs_concat_whole-seq_117.png]

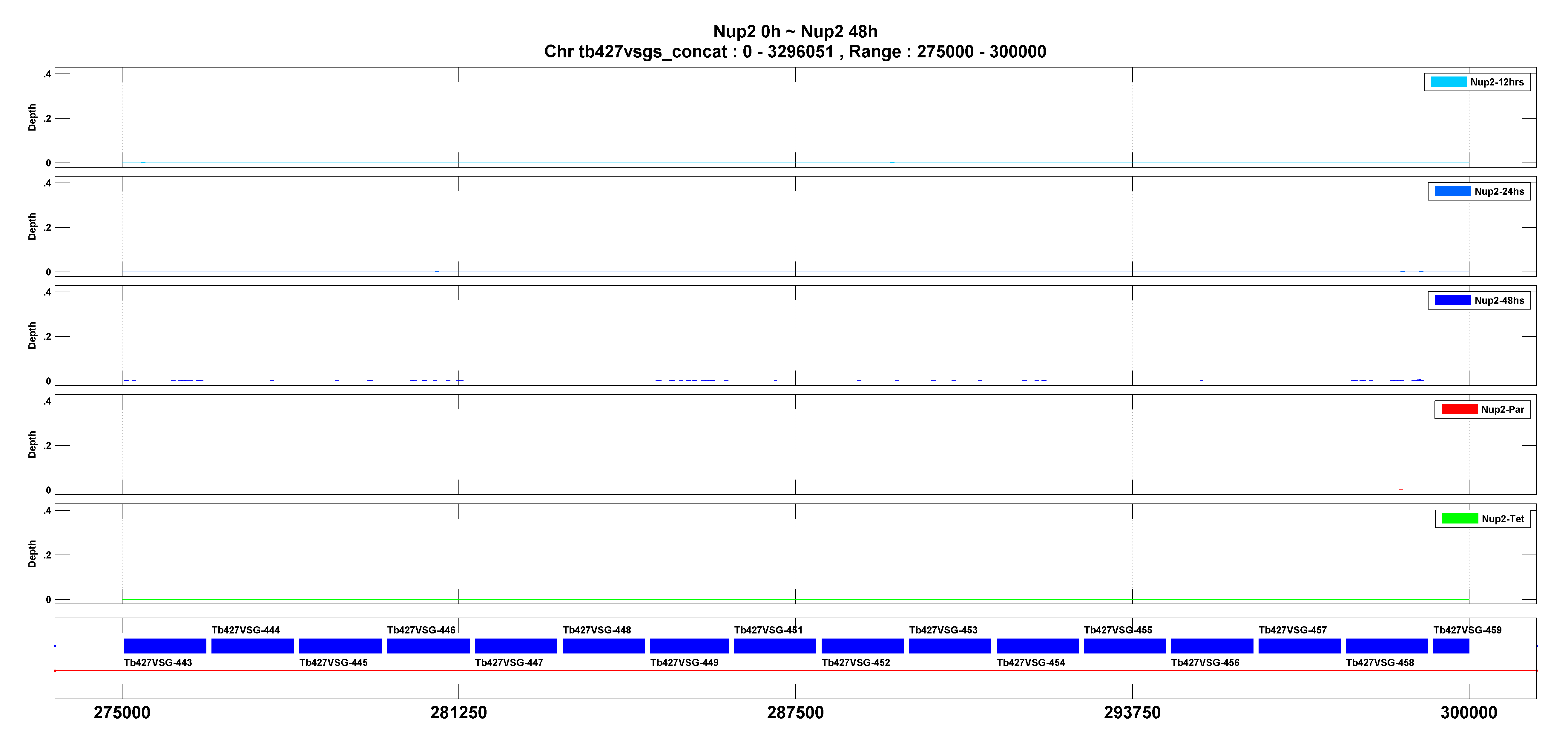

Supplement: SUPPLEMENTARY DATA [file supp_gkw751_nar-01100-x-2016-File026.zip › VSG transcriptome map/fig_tb427vsgs_concat_whole-seq_12.png]

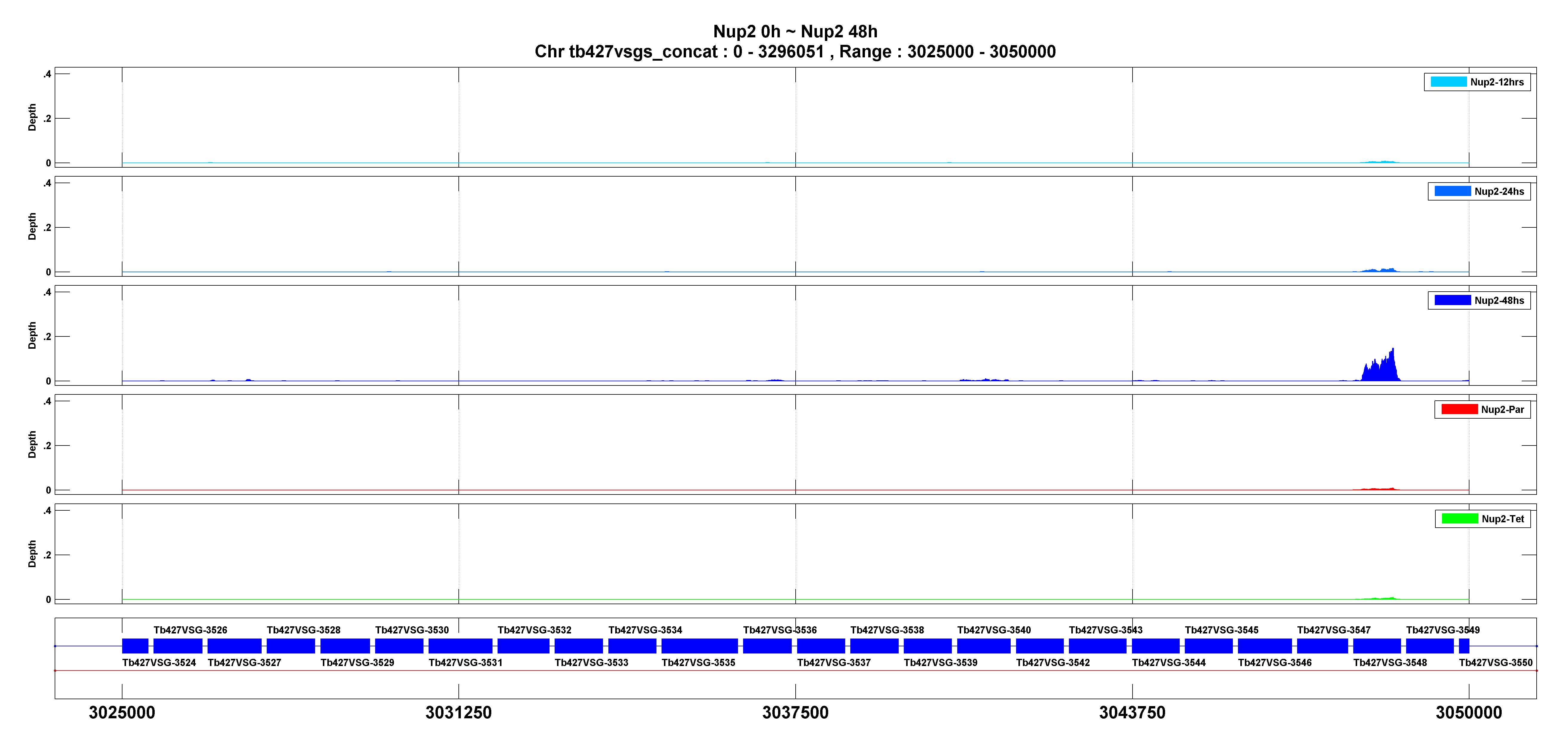

Supplement: SUPPLEMENTARY DATA [file supp_gkw751_nar-01100-x-2016-File026.zip › VSG transcriptome map/fig_tb427vsgs_concat_whole-seq_122.png]

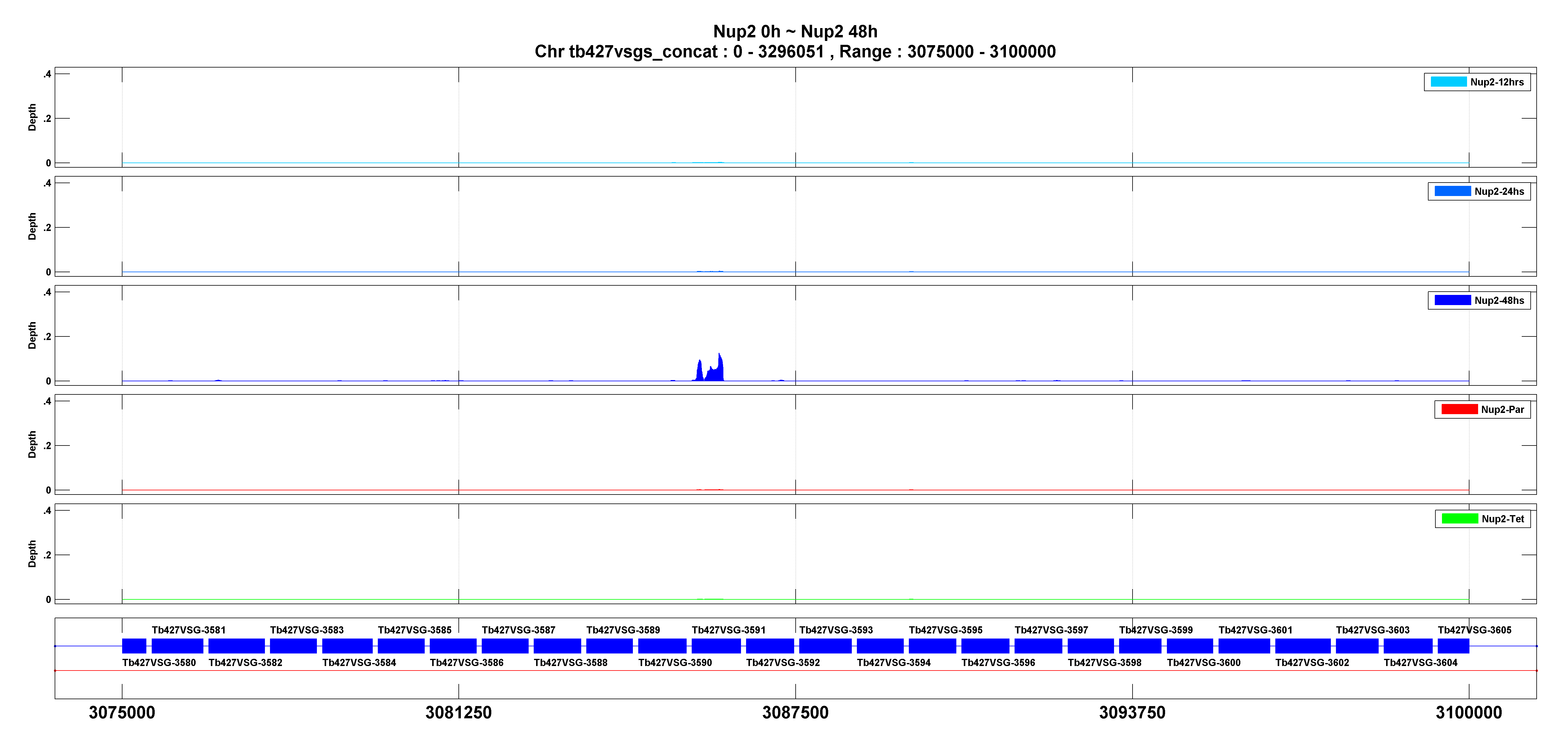

Supplement: SUPPLEMENTARY DATA [file supp_gkw751_nar-01100-x-2016-File026.zip › VSG transcriptome map/fig_tb427vsgs_concat_whole-seq_124.png]

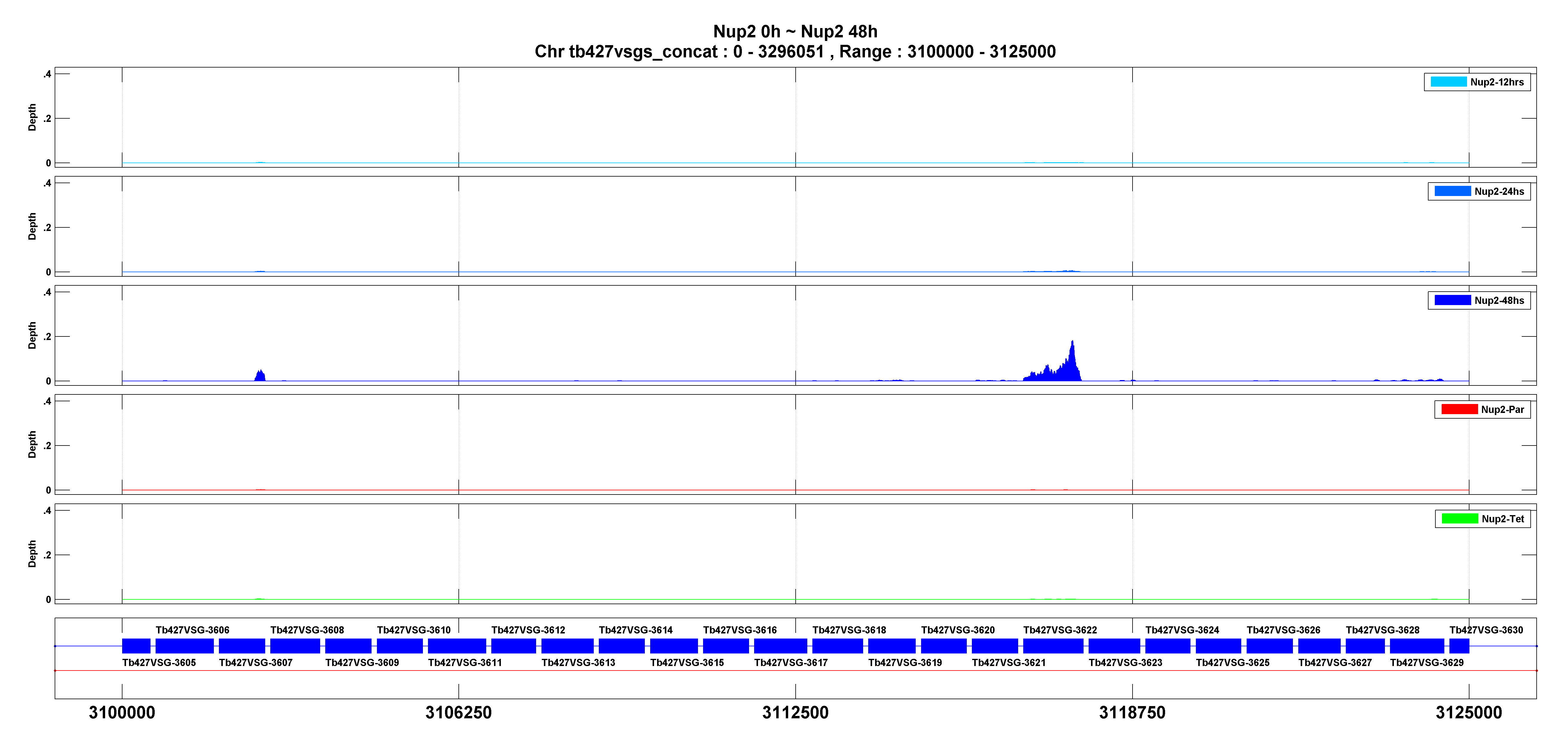

Supplement: SUPPLEMENTARY DATA [file supp_gkw751_nar-01100-x-2016-File026.zip › VSG transcriptome map/fig_tb427vsgs_concat_whole-seq_125.png]

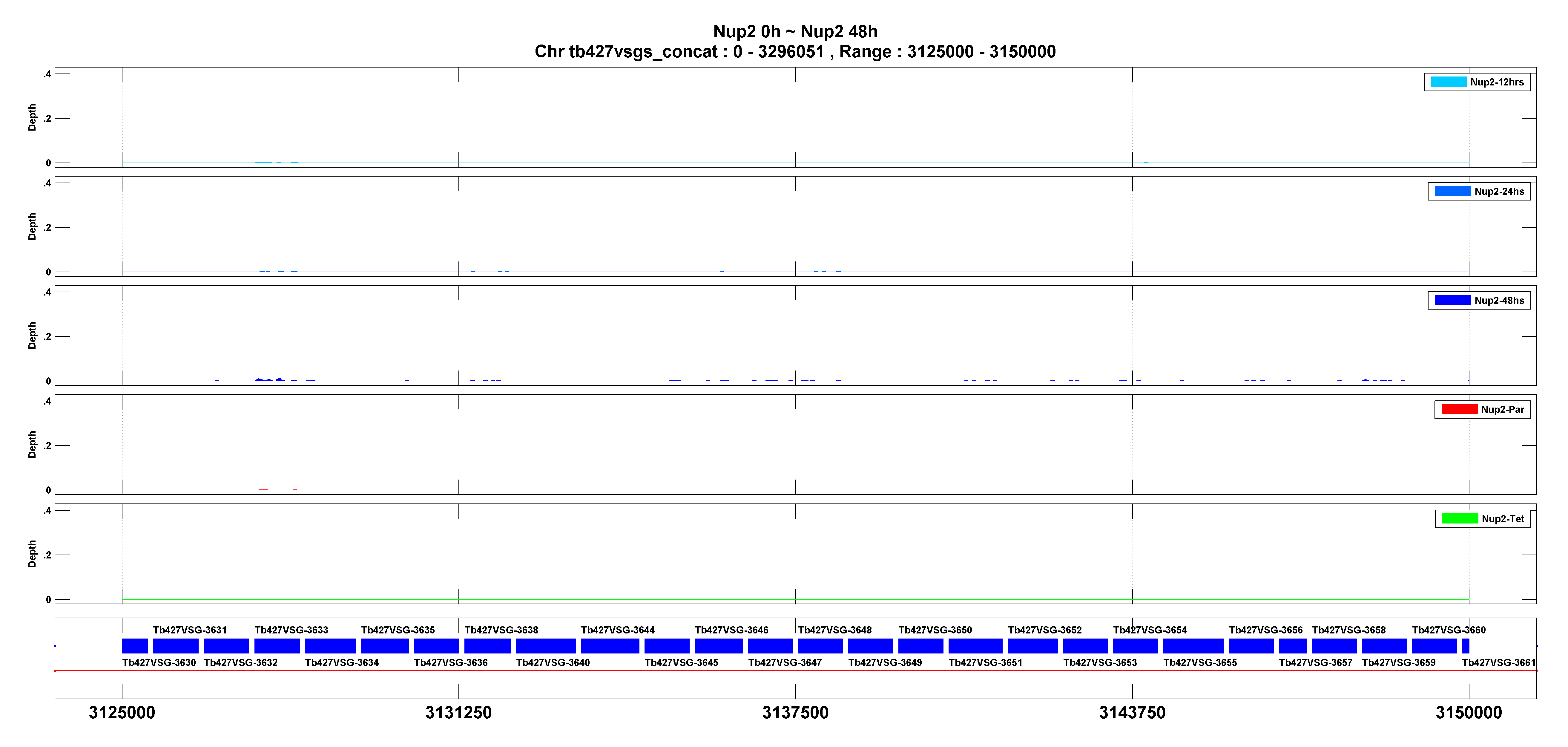

Supplement: SUPPLEMENTARY DATA [file supp_gkw751_nar-01100-x-2016-File026.zip › VSG transcriptome map/fig_tb427vsgs_concat_whole-seq_126.png]

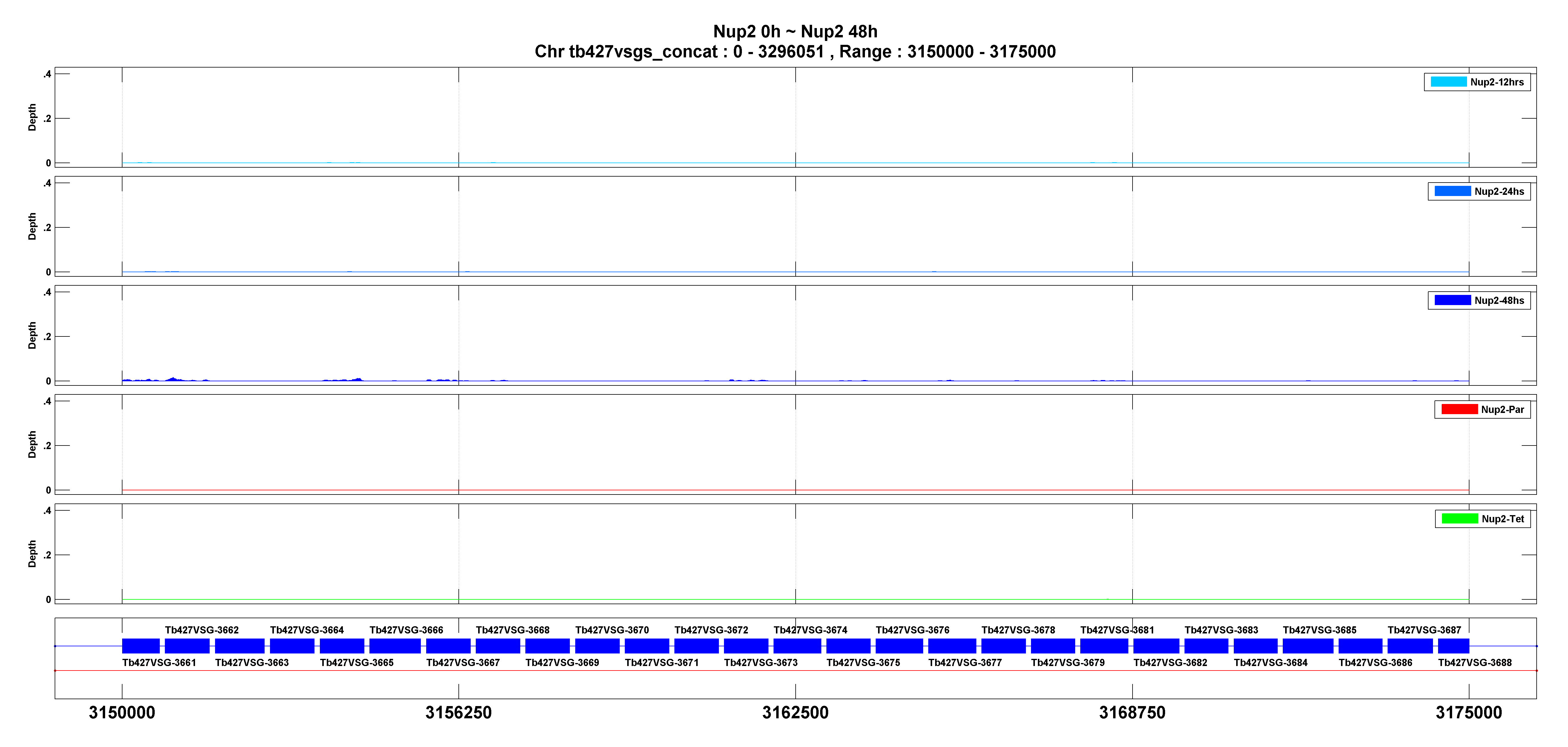

Supplement: SUPPLEMENTARY DATA [file supp_gkw751_nar-01100-x-2016-File026.zip › VSG transcriptome map/fig_tb427vsgs_concat_whole-seq_127.png]

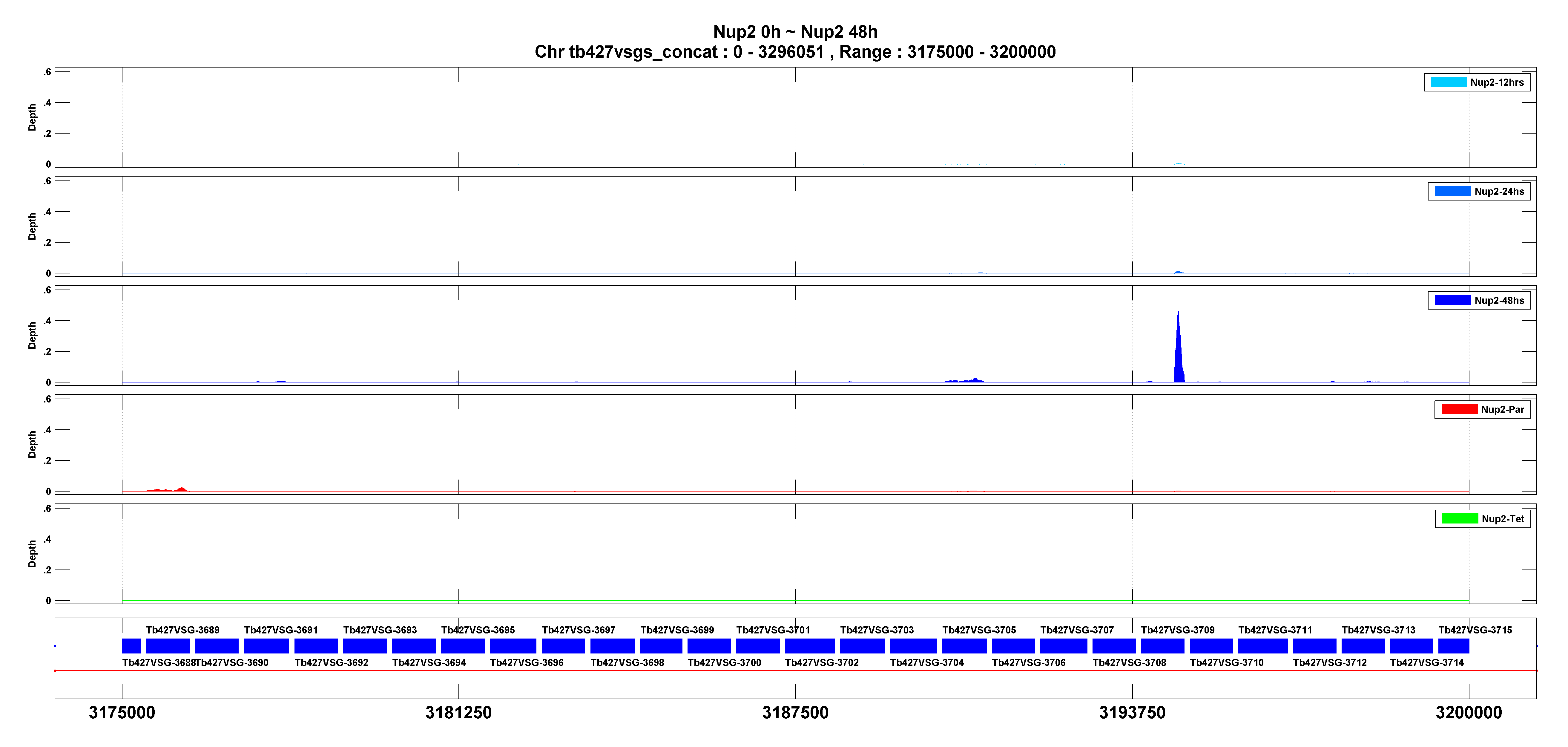

Supplement: SUPPLEMENTARY DATA [file supp_gkw751_nar-01100-x-2016-File026.zip › VSG transcriptome map/fig_tb427vsgs_concat_whole-seq_128.png]

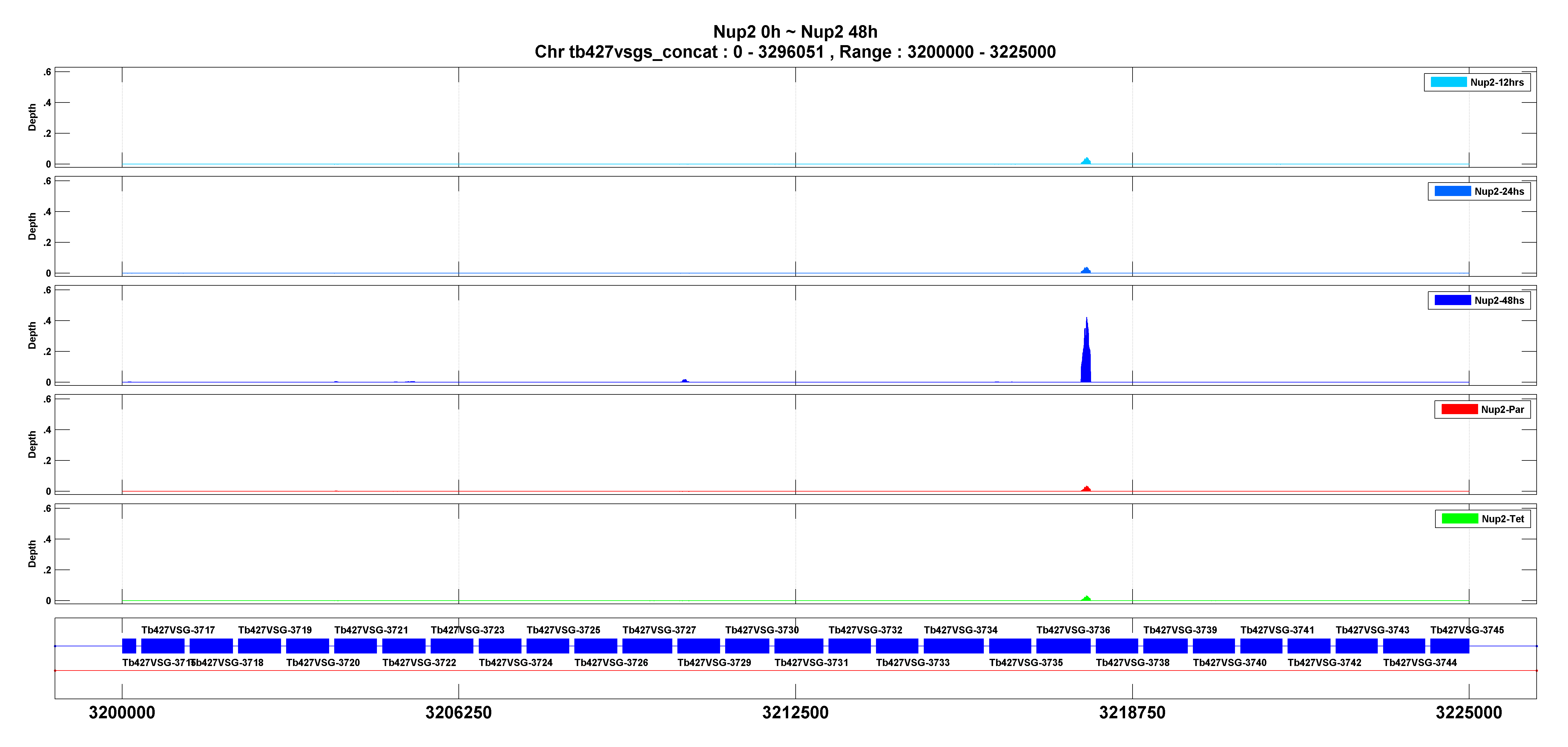

Supplement: SUPPLEMENTARY DATA [file supp_gkw751_nar-01100-x-2016-File026.zip › VSG transcriptome map/fig_tb427vsgs_concat_whole-seq_129.png]

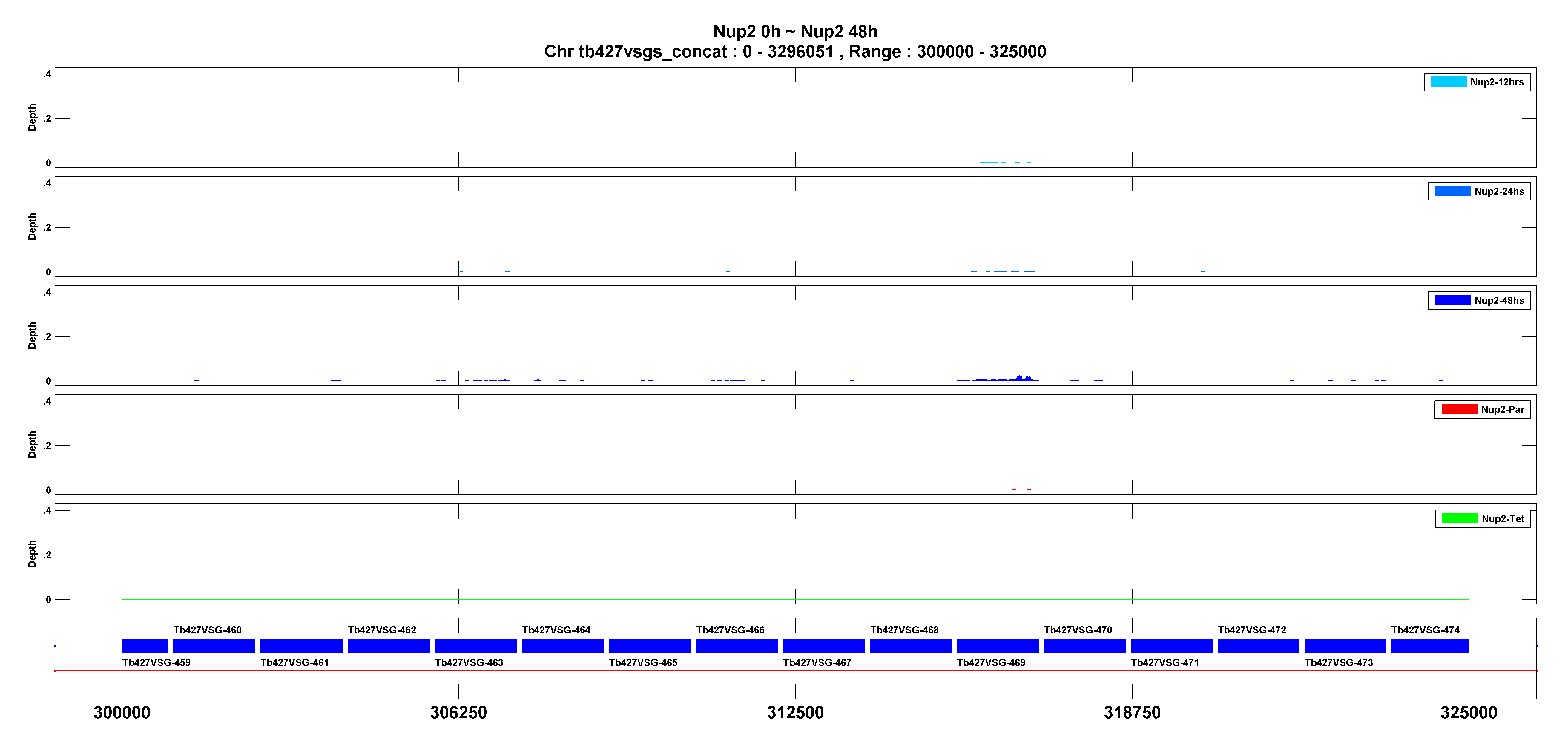

Supplement: SUPPLEMENTARY DATA [file supp_gkw751_nar-01100-x-2016-File026.zip › VSG transcriptome map/fig_tb427vsgs_concat_whole-seq_13.png]

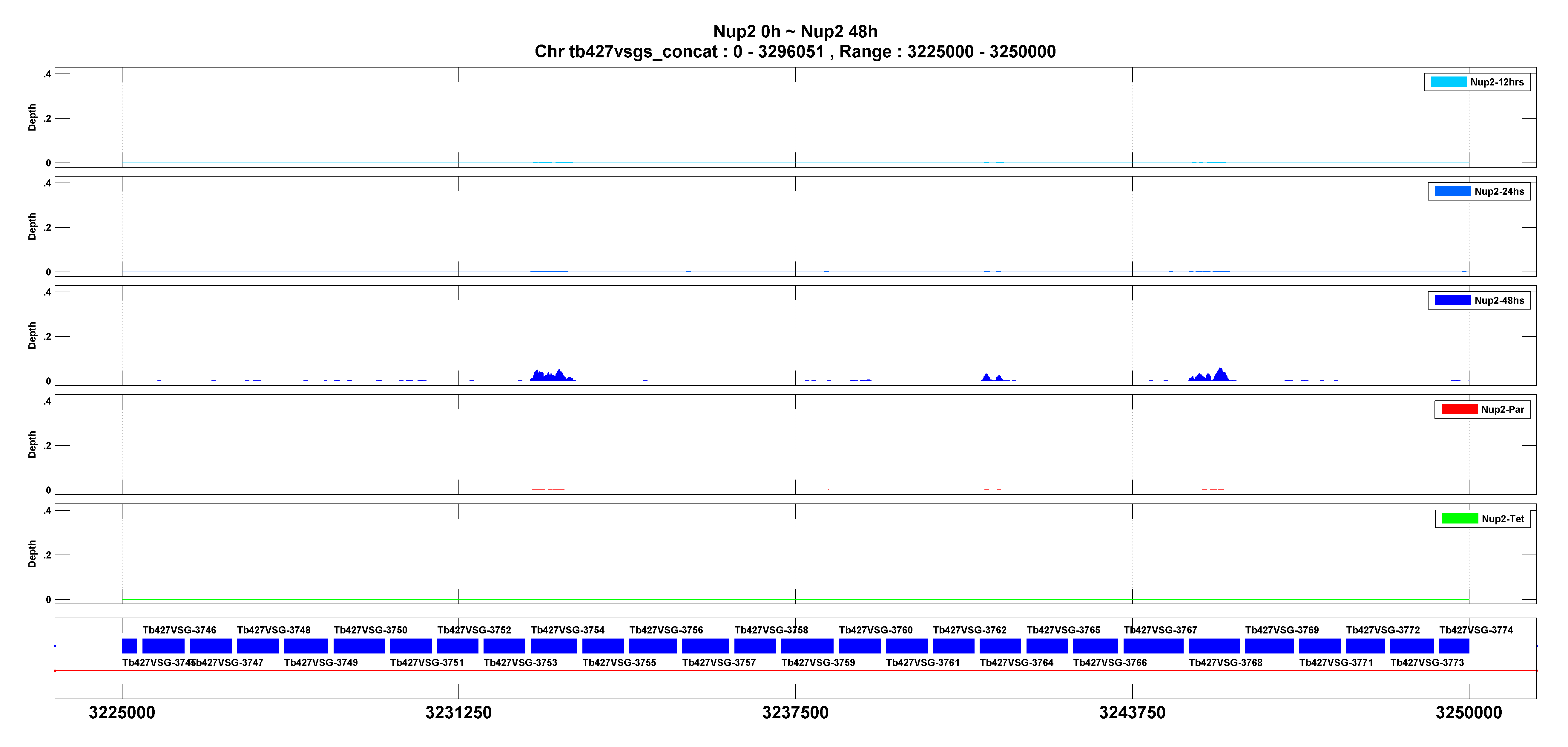

Supplement: SUPPLEMENTARY DATA [file supp_gkw751_nar-01100-x-2016-File026.zip › VSG transcriptome map/fig_tb427vsgs_concat_whole-seq_130.png]

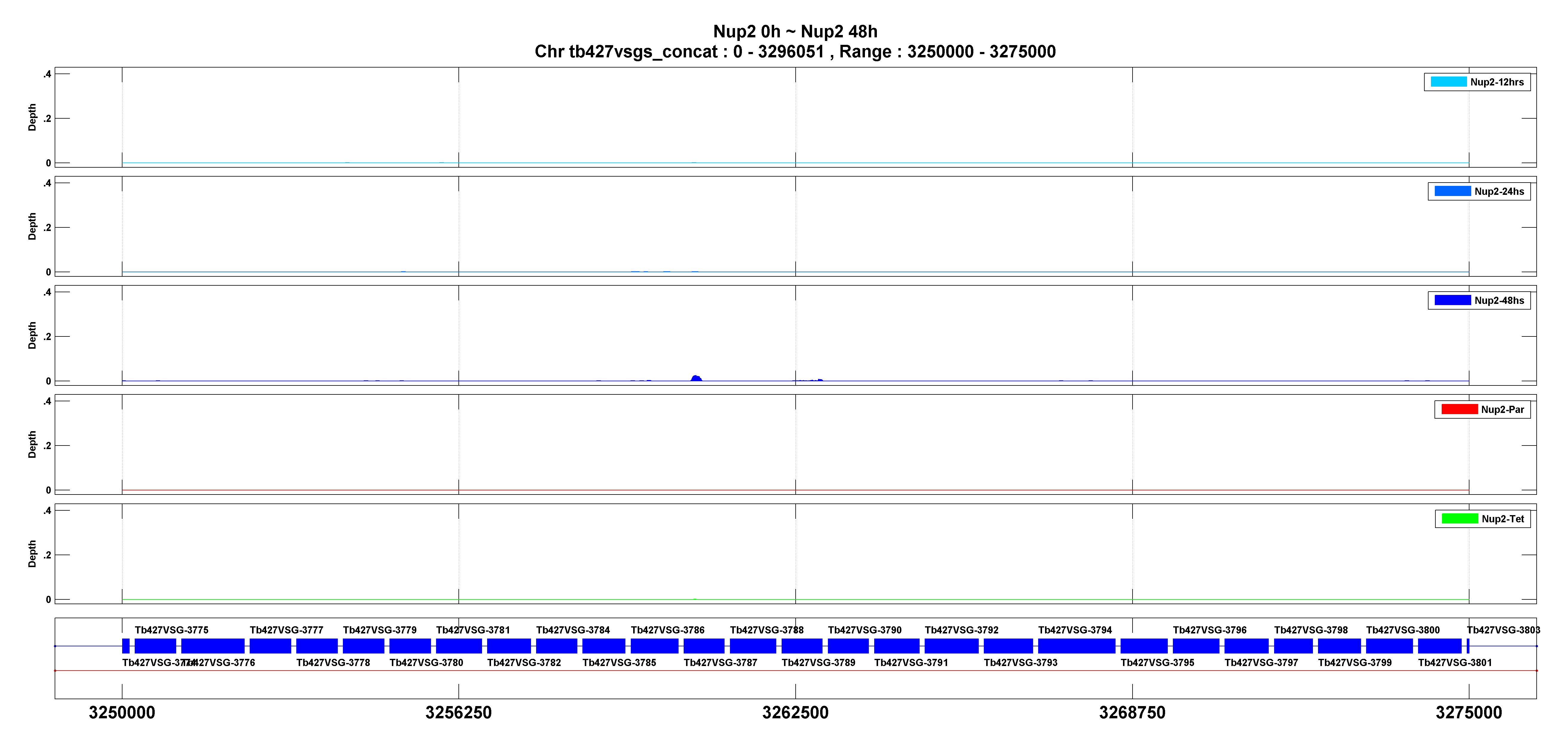

Supplement: SUPPLEMENTARY DATA [file supp_gkw751_nar-01100-x-2016-File026.zip › VSG transcriptome map/fig_tb427vsgs_concat_whole-seq_131.png]

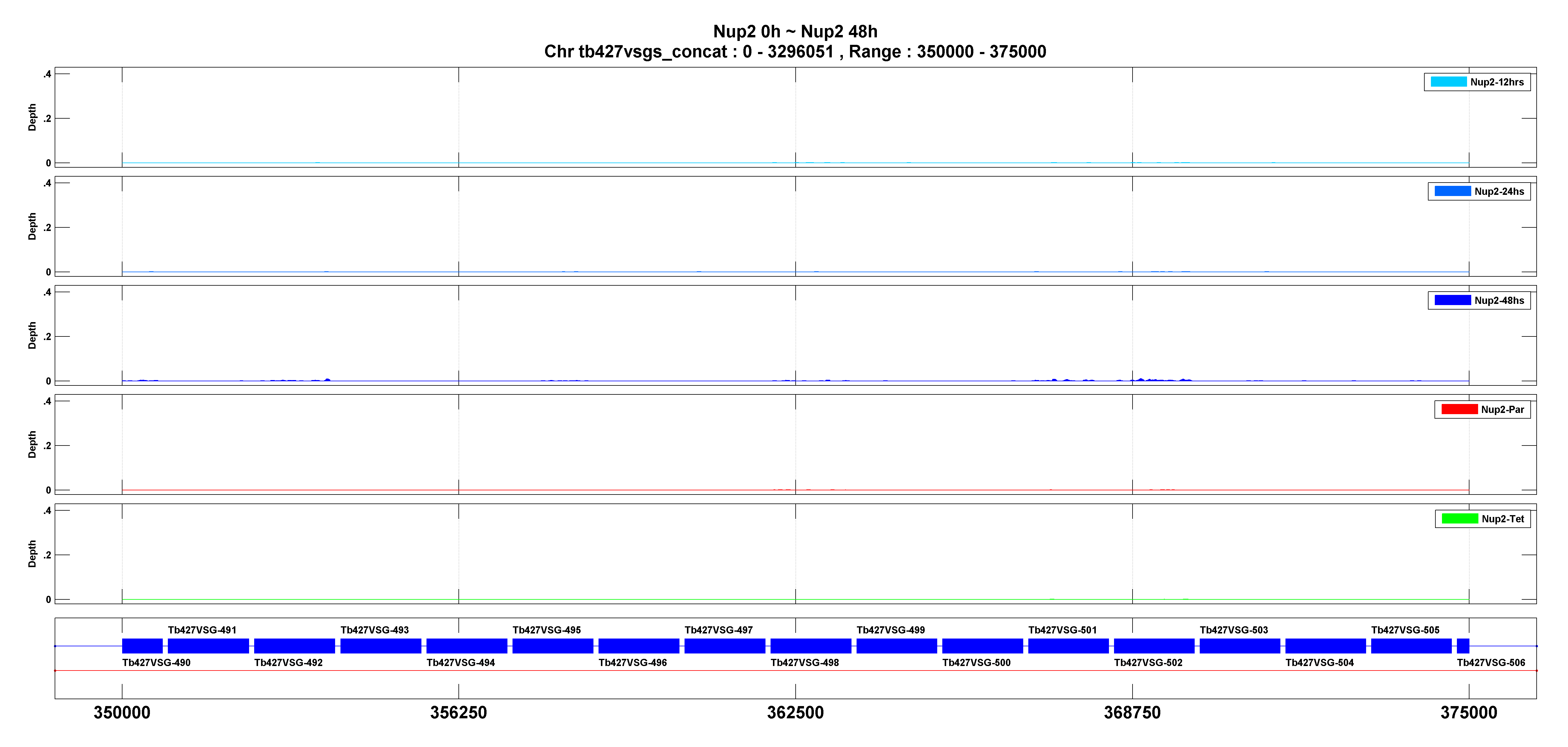

Supplement: SUPPLEMENTARY DATA [file supp_gkw751_nar-01100-x-2016-File026.zip › VSG transcriptome map/fig_tb427vsgs_concat_whole-seq_15.png]

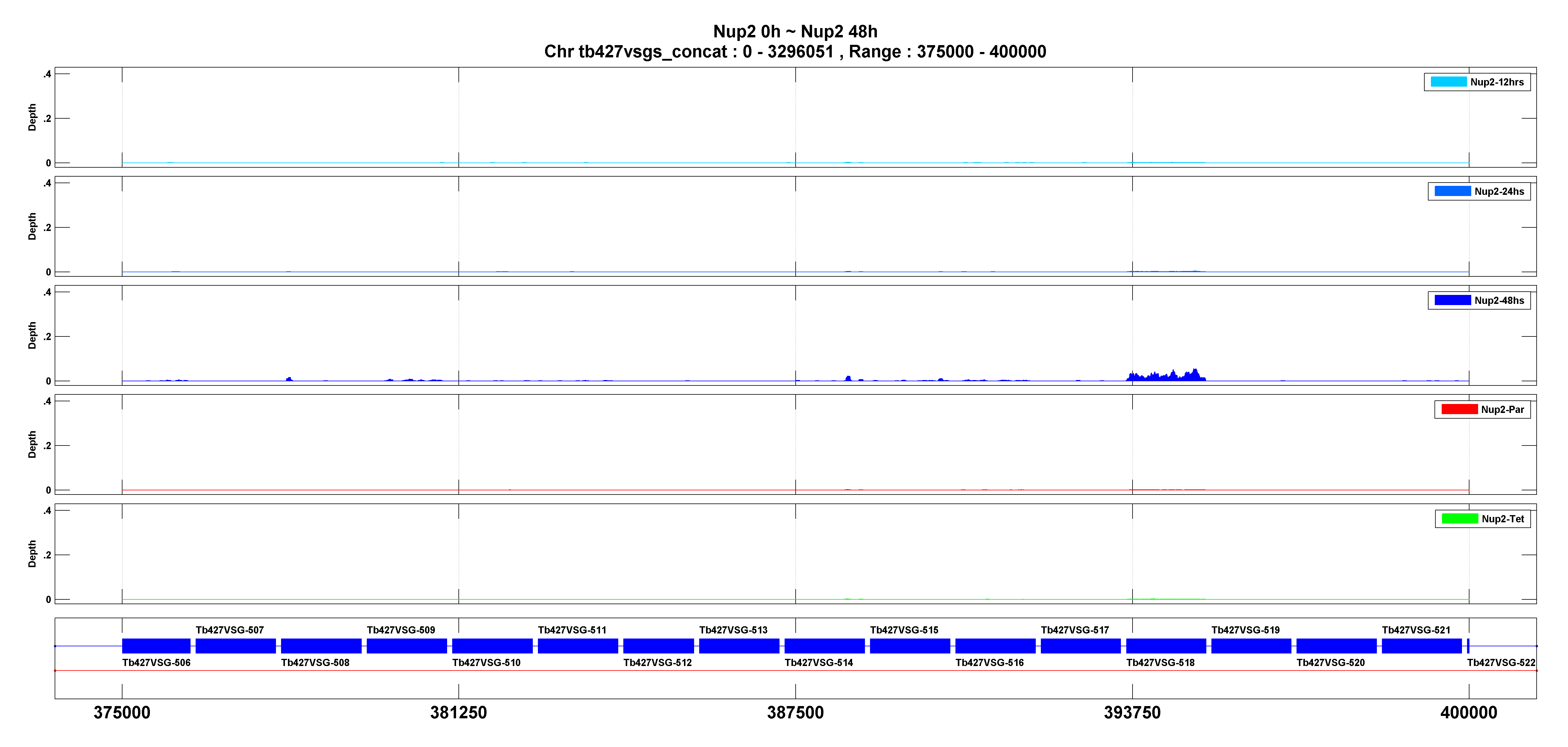

Supplement: SUPPLEMENTARY DATA [file supp_gkw751_nar-01100-x-2016-File026.zip › VSG transcriptome map/fig_tb427vsgs_concat_whole-seq_16.png]

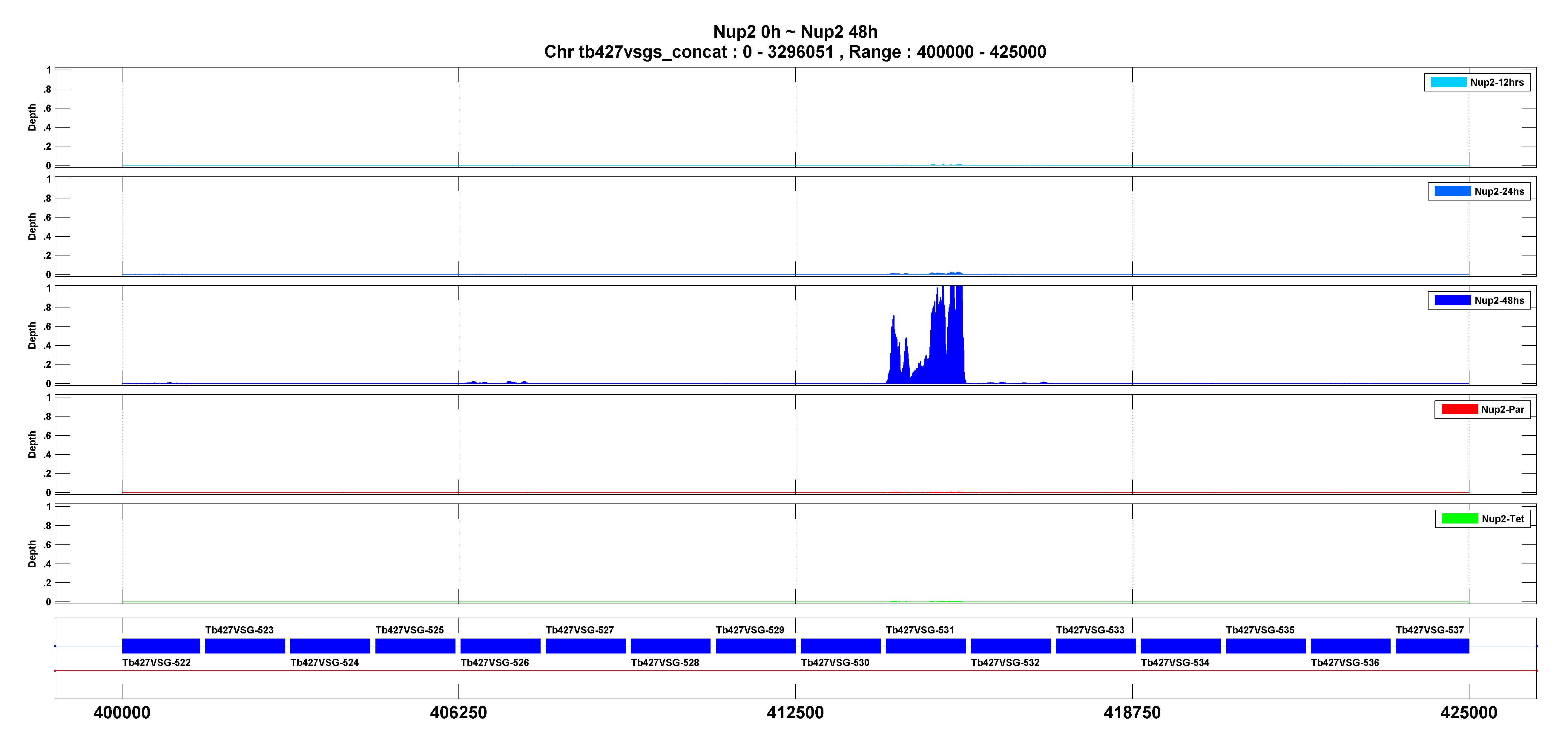

Supplement: SUPPLEMENTARY DATA [file supp_gkw751_nar-01100-x-2016-File026.zip › VSG transcriptome map/fig_tb427vsgs_concat_whole-seq_17.png]

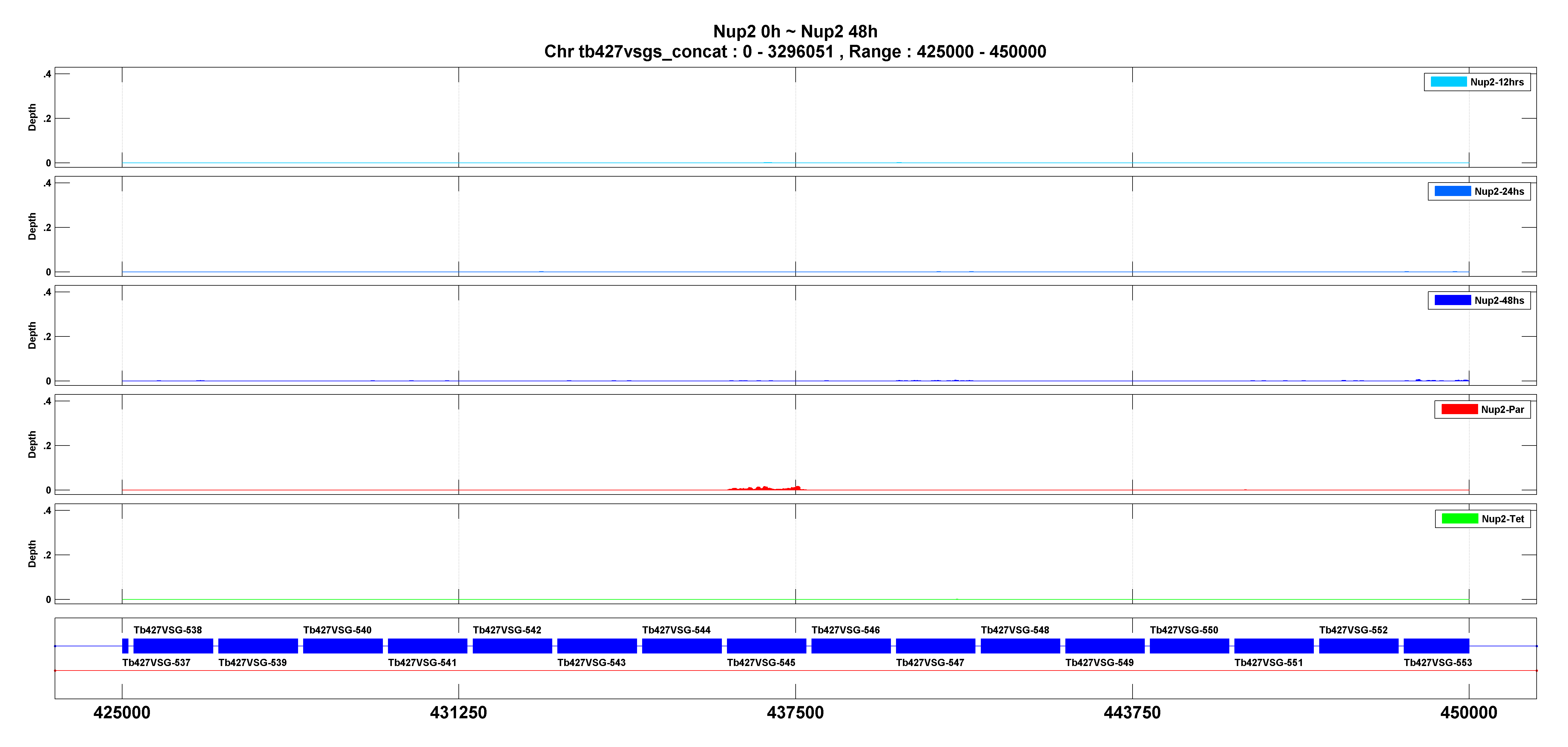

Supplement: SUPPLEMENTARY DATA [file supp_gkw751_nar-01100-x-2016-File026.zip › VSG transcriptome map/fig_tb427vsgs_concat_whole-seq_18.png]

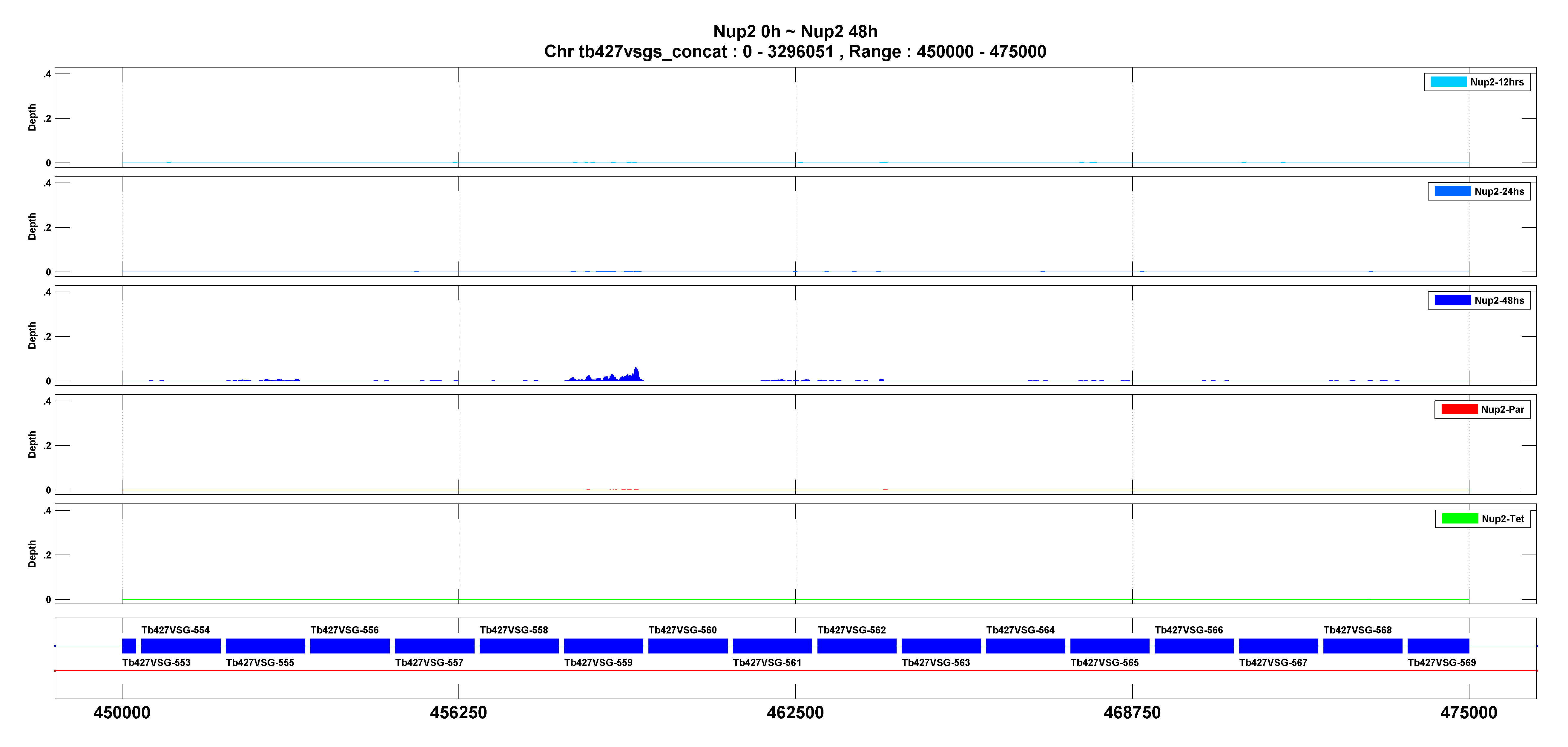

Supplement: SUPPLEMENTARY DATA [file supp_gkw751_nar-01100-x-2016-File026.zip › VSG transcriptome map/fig_tb427vsgs_concat_whole-seq_19.png]

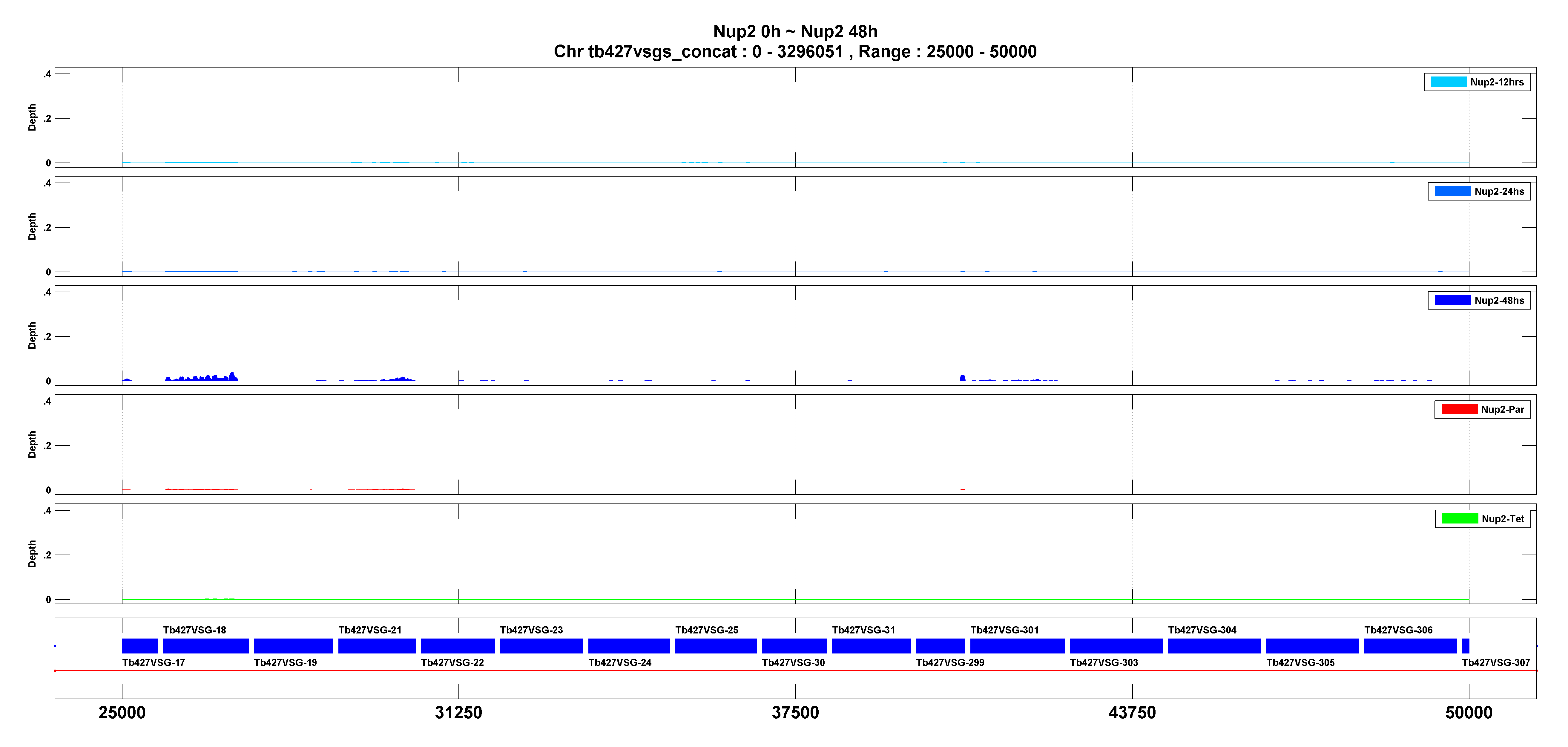

Supplement: SUPPLEMENTARY DATA [file supp_gkw751_nar-01100-x-2016-File026.zip › VSG transcriptome map/fig_tb427vsgs_concat_whole-seq_2.png]

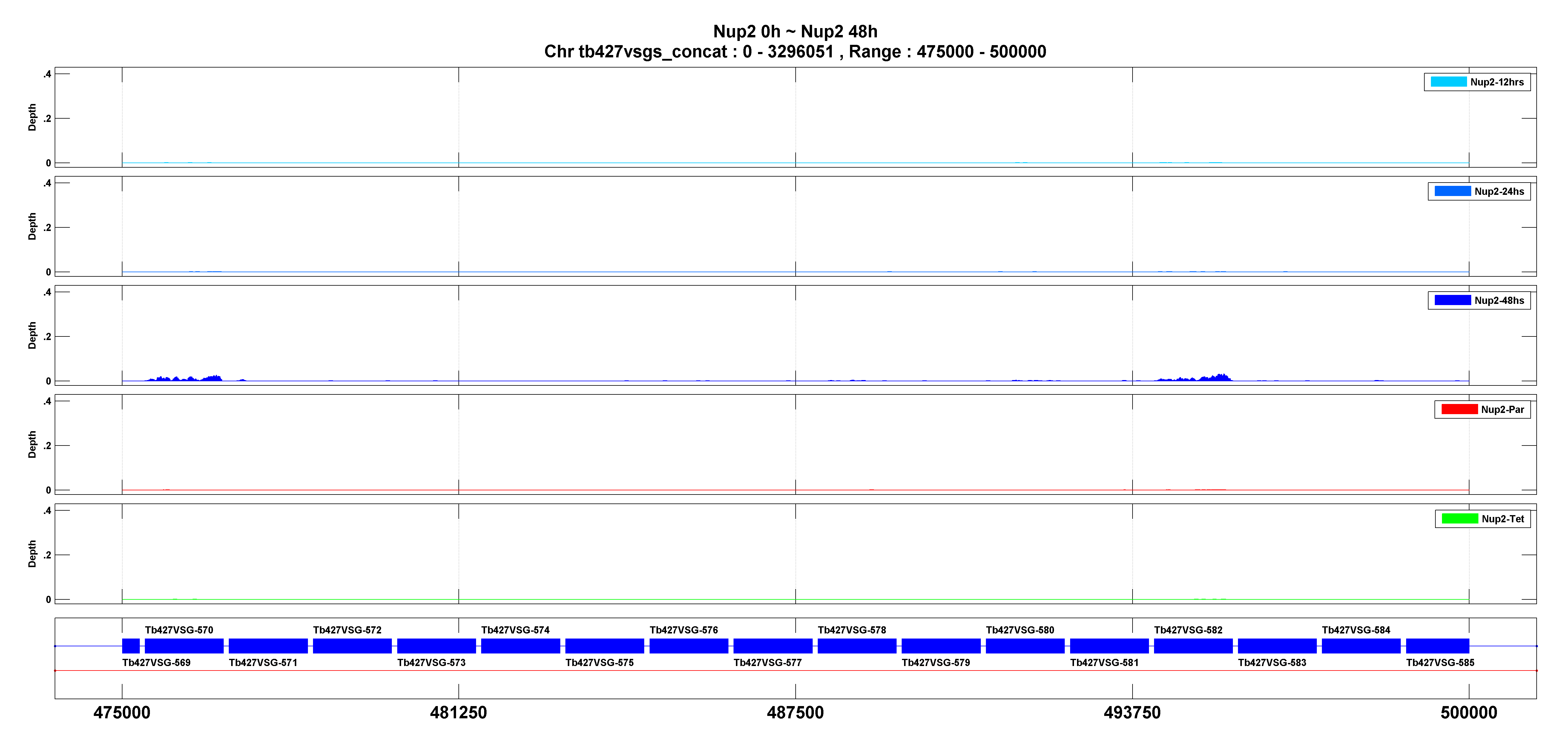

Supplement: SUPPLEMENTARY DATA [file supp_gkw751_nar-01100-x-2016-File026.zip › VSG transcriptome map/fig_tb427vsgs_concat_whole-seq_20.png]

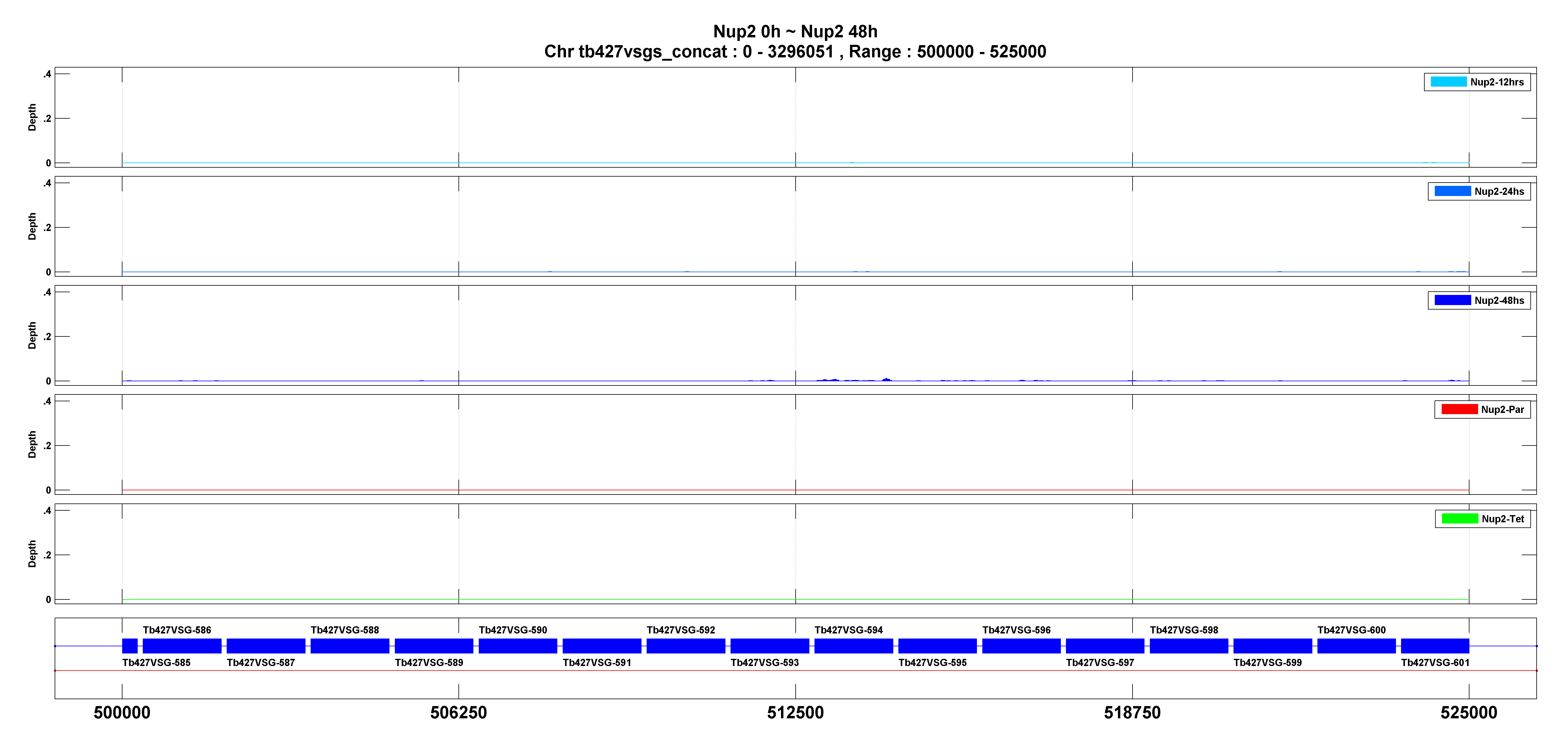

Supplement: SUPPLEMENTARY DATA [file supp_gkw751_nar-01100-x-2016-File026.zip › VSG transcriptome map/fig_tb427vsgs_concat_whole-seq_21.png]

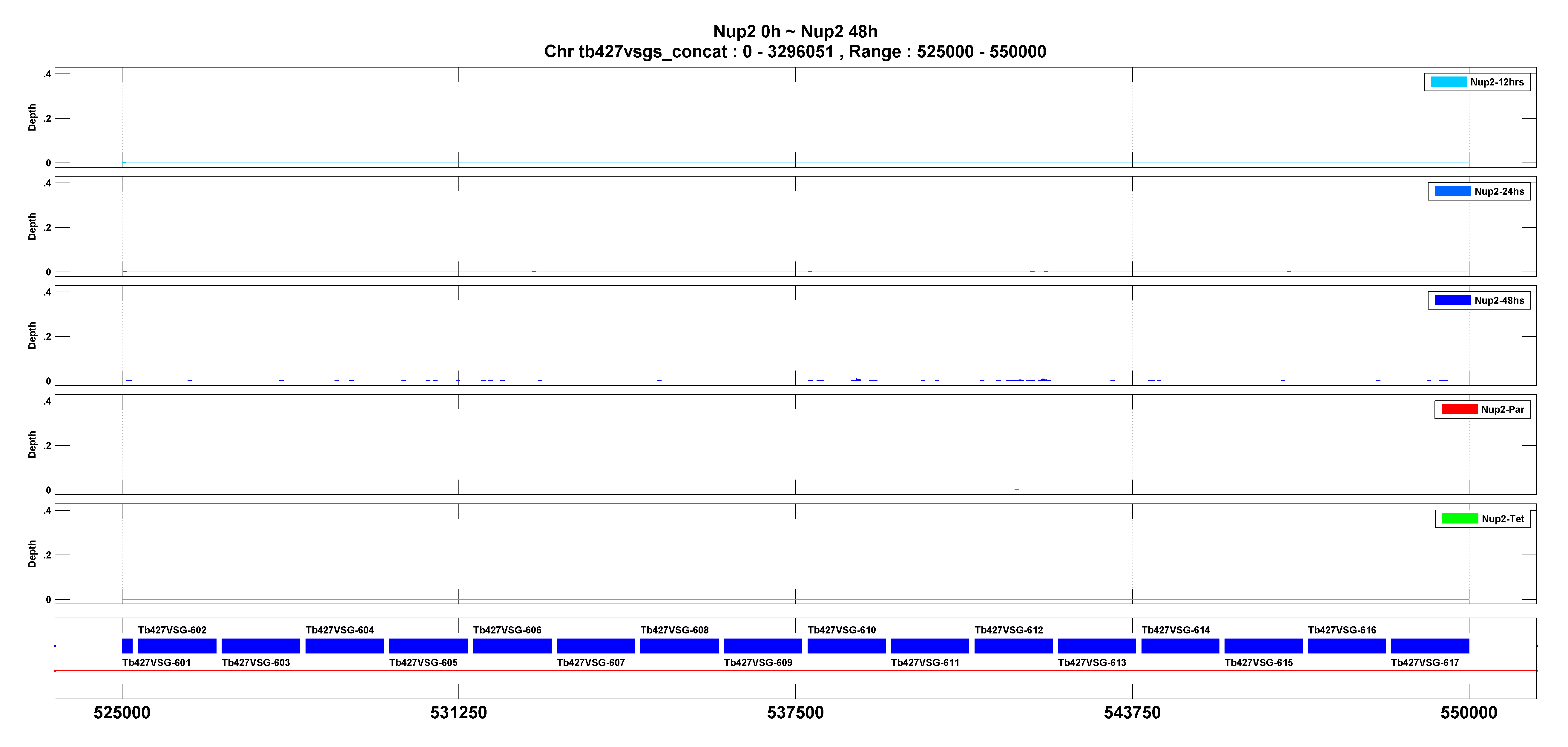

Supplement: SUPPLEMENTARY DATA [file supp_gkw751_nar-01100-x-2016-File026.zip › VSG transcriptome map/fig_tb427vsgs_concat_whole-seq_22.png]

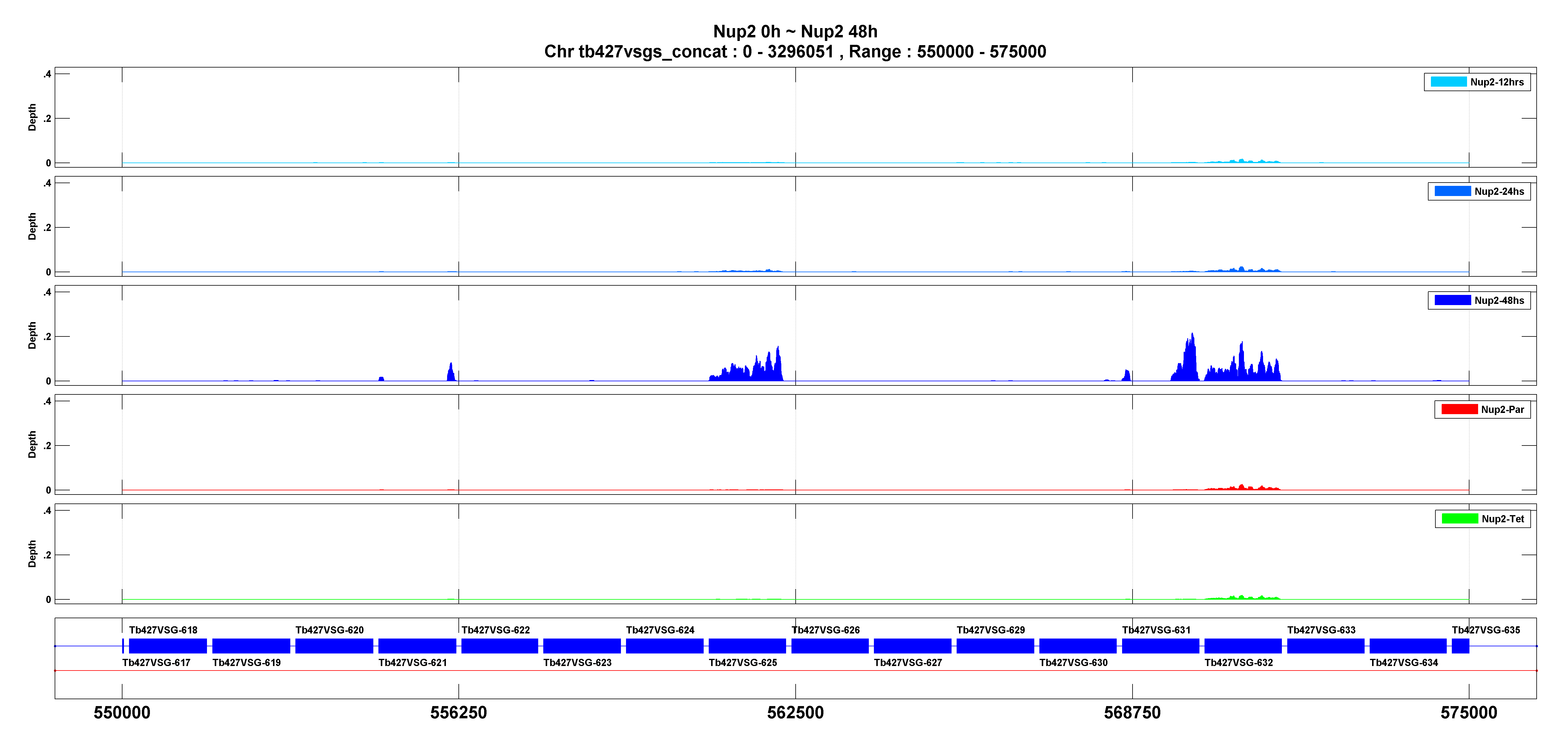

Supplement: SUPPLEMENTARY DATA [file supp_gkw751_nar-01100-x-2016-File026.zip › VSG transcriptome map/fig_tb427vsgs_concat_whole-seq_23.png]

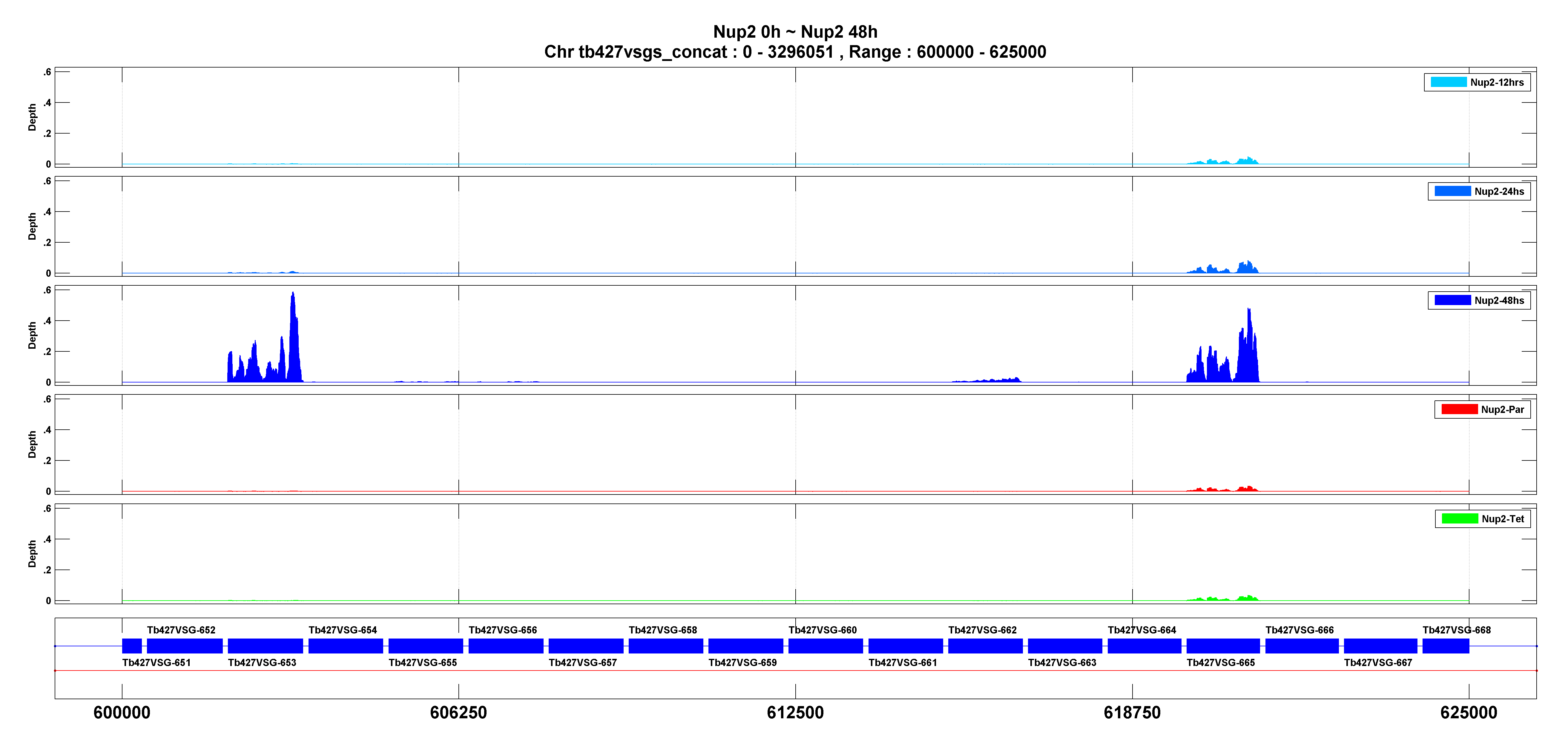

Supplement: SUPPLEMENTARY DATA [file supp_gkw751_nar-01100-x-2016-File026.zip › VSG transcriptome map/fig_tb427vsgs_concat_whole-seq_25.png]

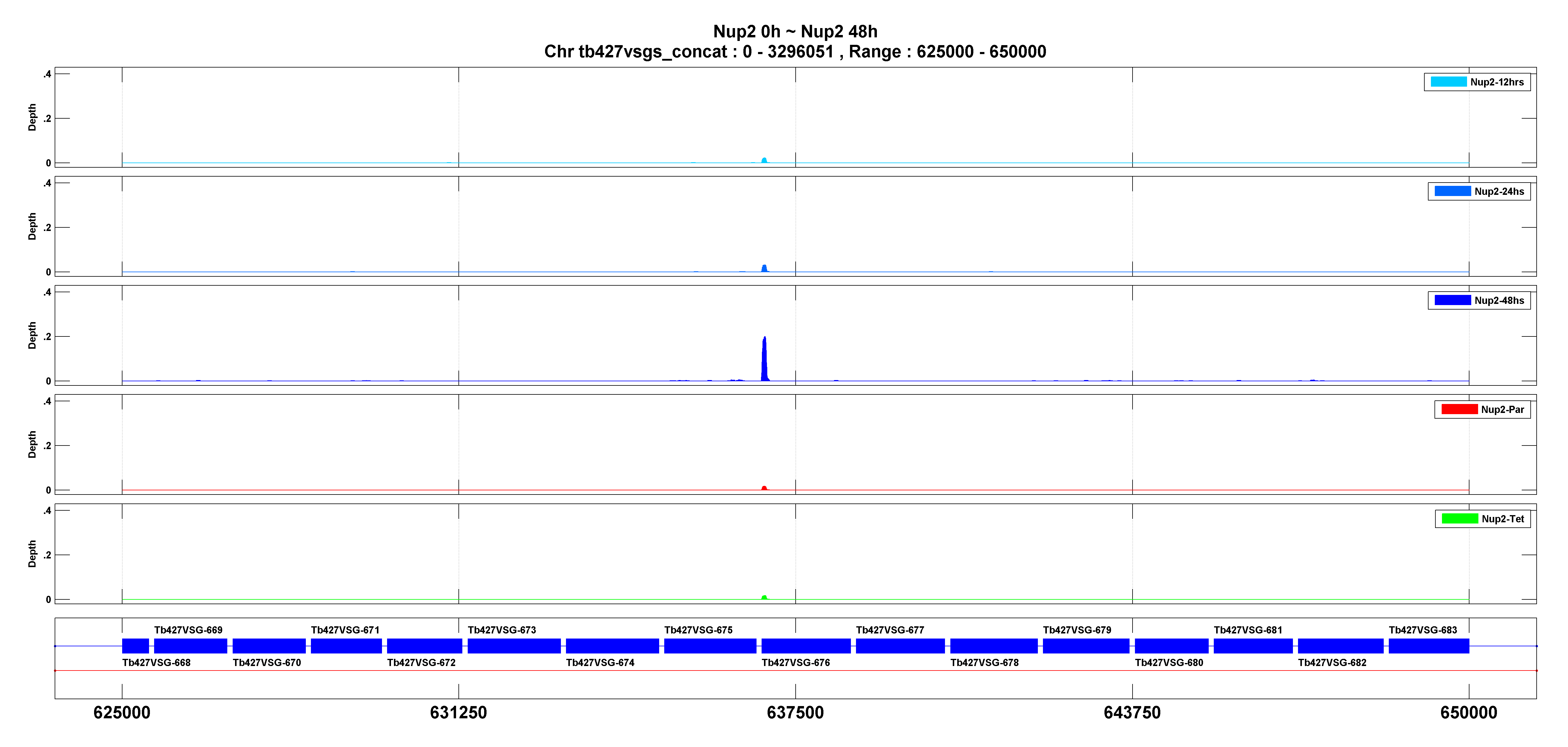

Supplement: SUPPLEMENTARY DATA [file supp_gkw751_nar-01100-x-2016-File026.zip › VSG transcriptome map/fig_tb427vsgs_concat_whole-seq_26.png]

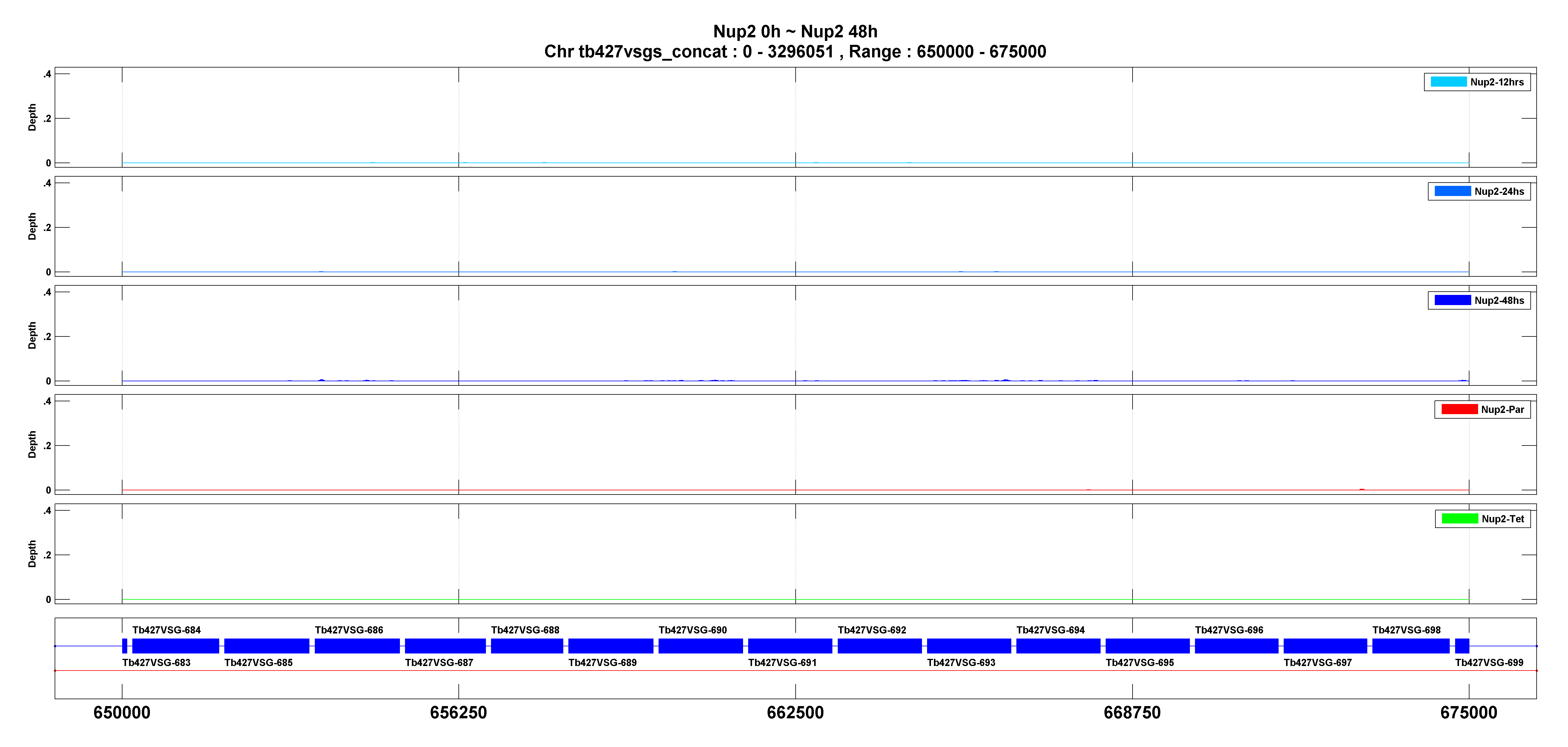

Supplement: SUPPLEMENTARY DATA [file supp_gkw751_nar-01100-x-2016-File026.zip › VSG transcriptome map/fig_tb427vsgs_concat_whole-seq_27.png]

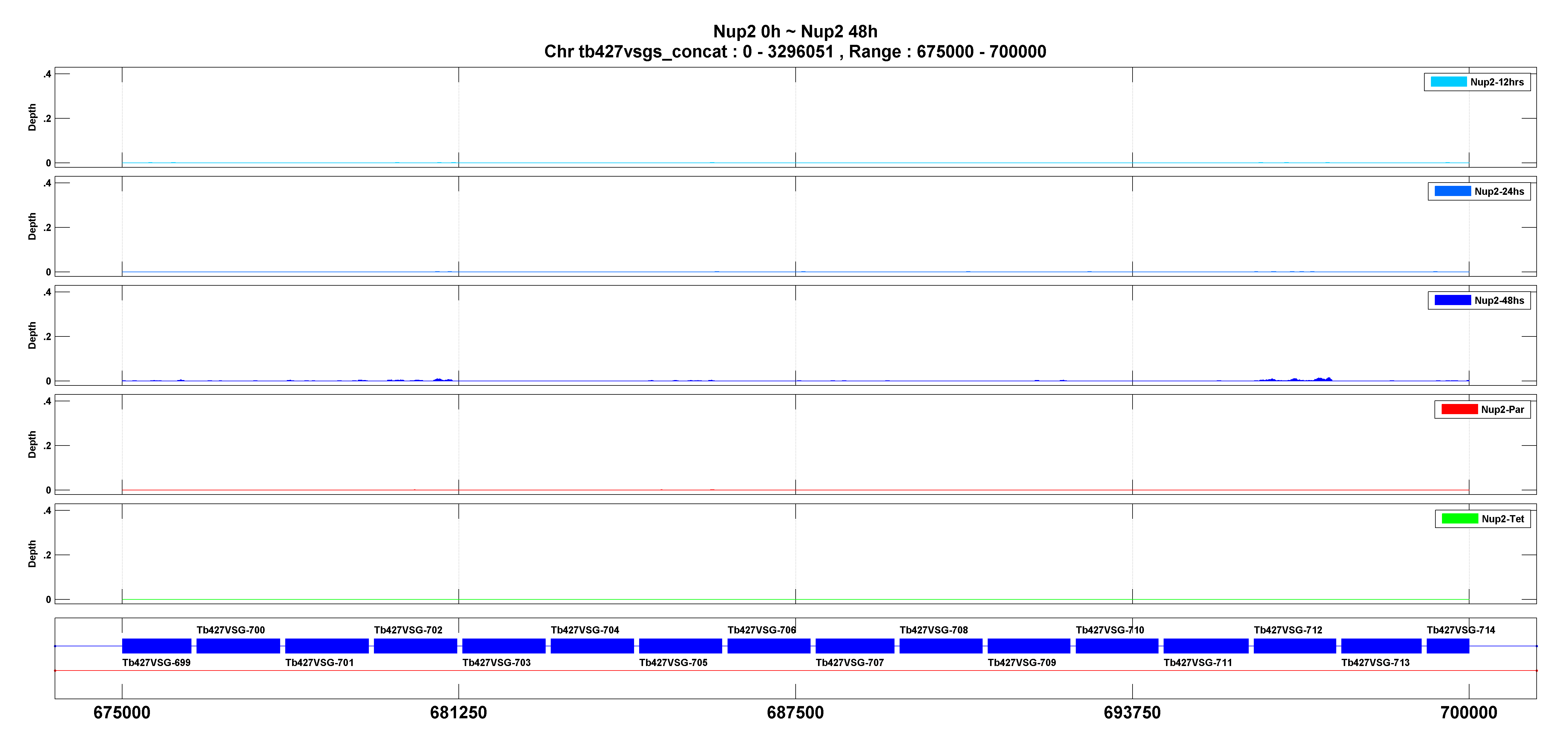

Supplement: SUPPLEMENTARY DATA [file supp_gkw751_nar-01100-x-2016-File026.zip › VSG transcriptome map/fig_tb427vsgs_concat_whole-seq_28.png]

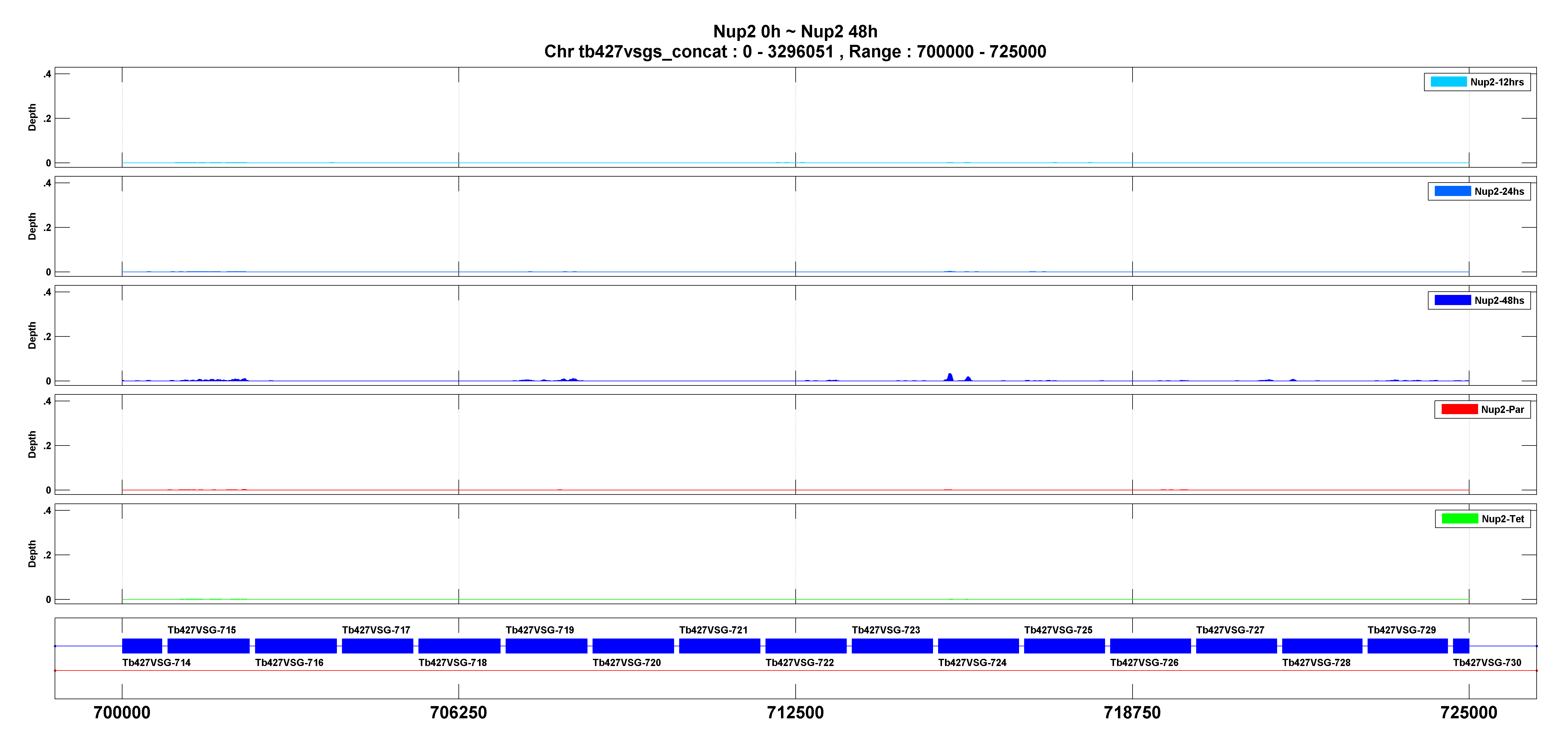

Supplement: SUPPLEMENTARY DATA [file supp_gkw751_nar-01100-x-2016-File026.zip › VSG transcriptome map/fig_tb427vsgs_concat_whole-seq_29.png]

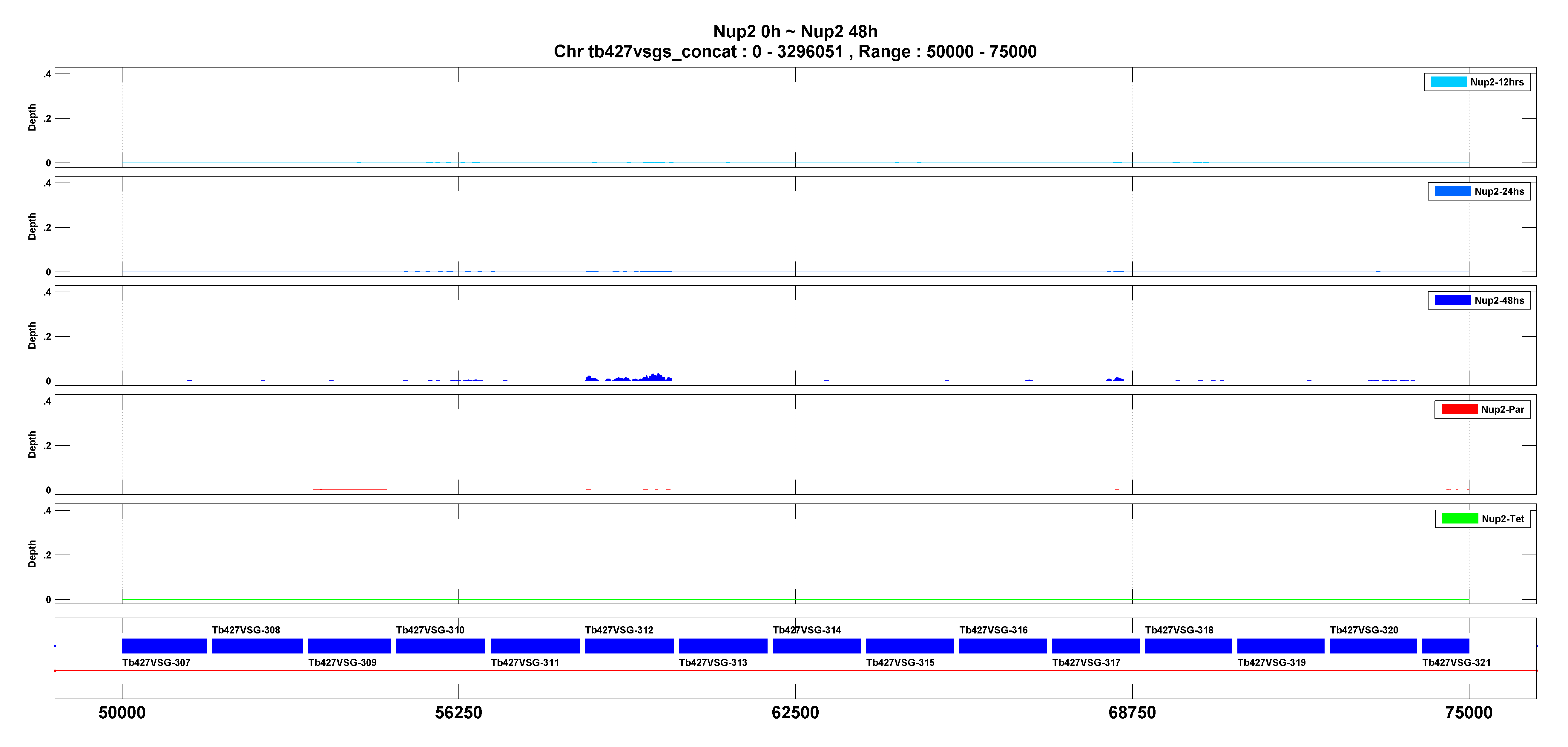

Supplement: SUPPLEMENTARY DATA [file supp_gkw751_nar-01100-x-2016-File026.zip › VSG transcriptome map/fig_tb427vsgs_concat_whole-seq_3.png]

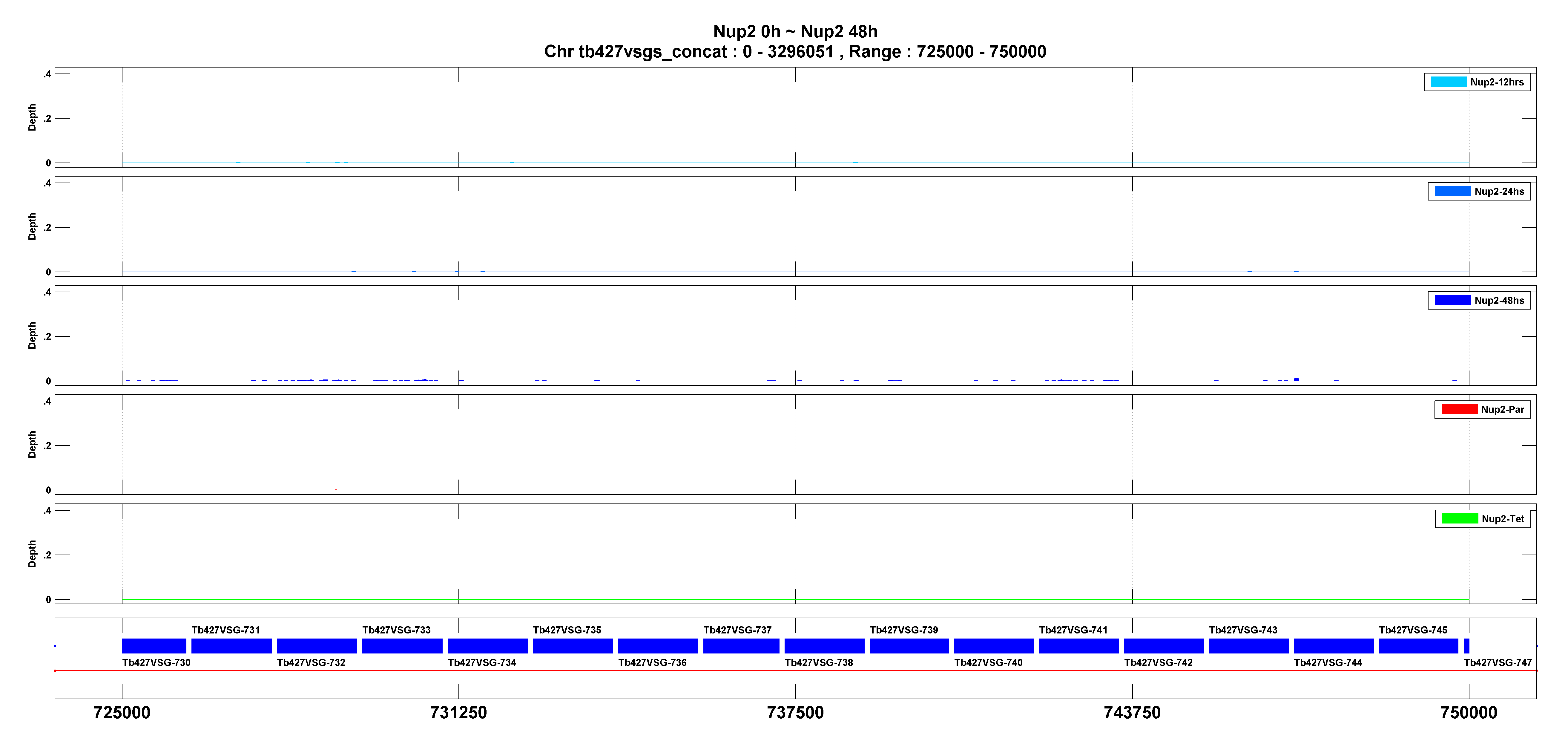

Supplement: SUPPLEMENTARY DATA [file supp_gkw751_nar-01100-x-2016-File026.zip › VSG transcriptome map/fig_tb427vsgs_concat_whole-seq_30.png]

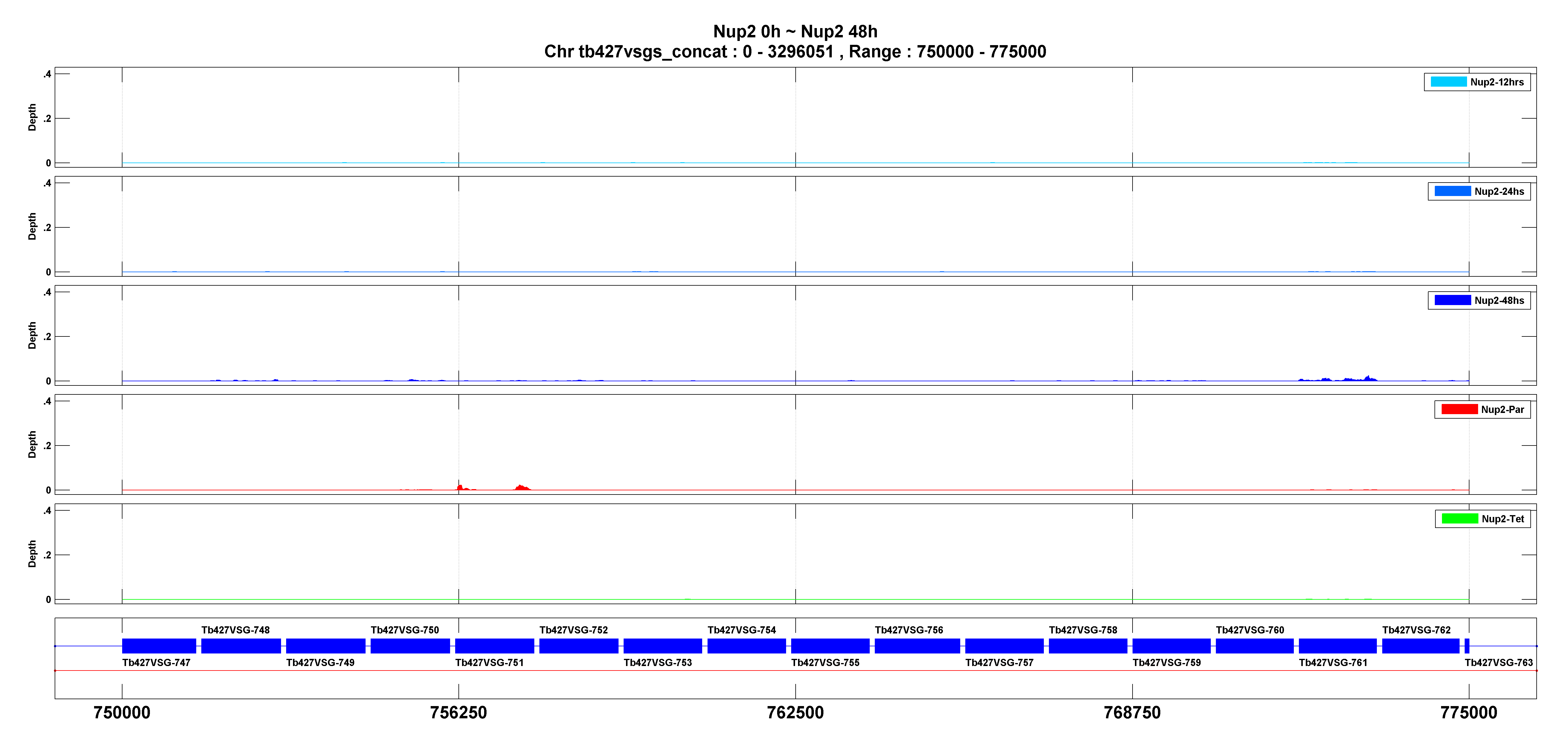

Supplement: SUPPLEMENTARY DATA [file supp_gkw751_nar-01100-x-2016-File026.zip › VSG transcriptome map/fig_tb427vsgs_concat_whole-seq_31.png]

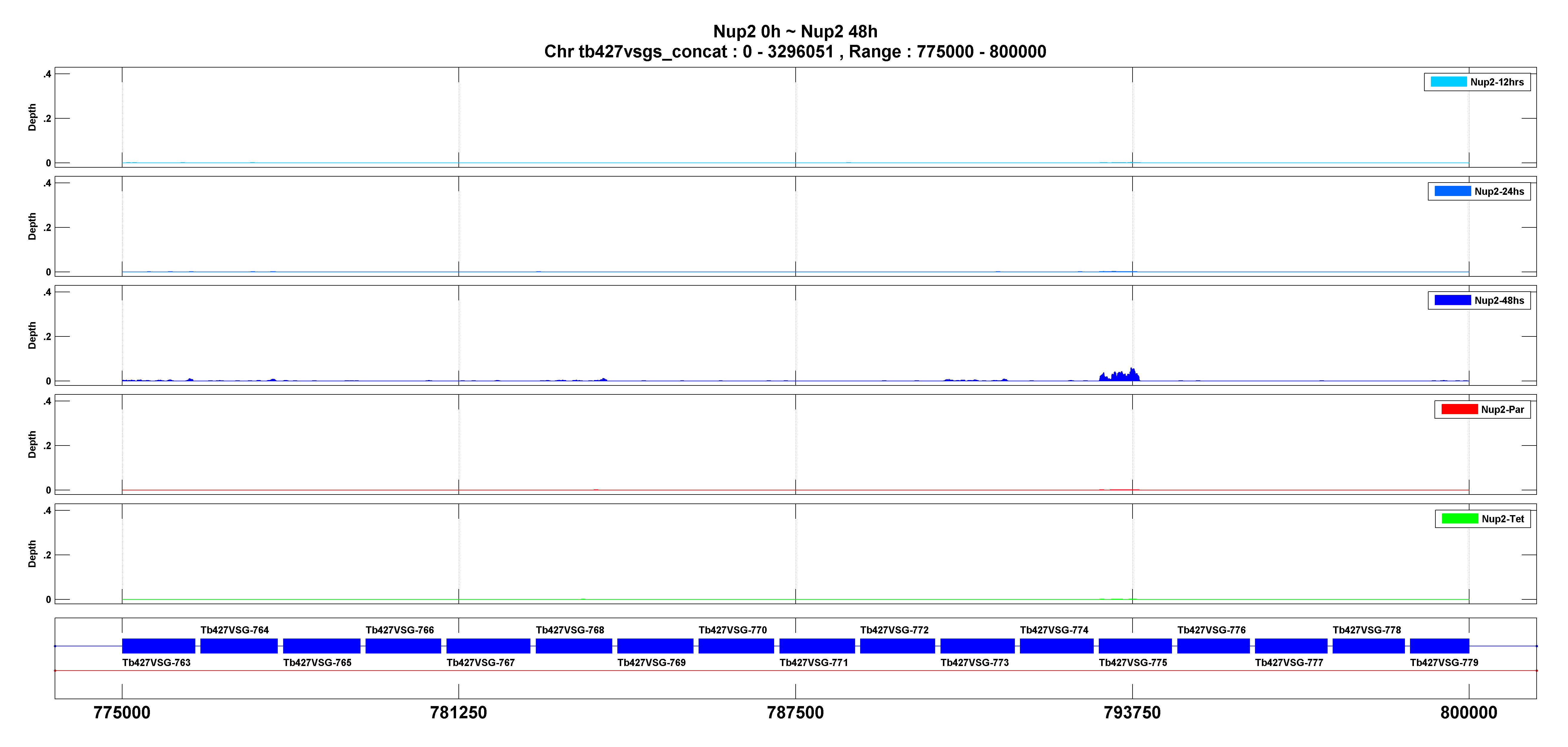

Supplement: SUPPLEMENTARY DATA [file supp_gkw751_nar-01100-x-2016-File026.zip › VSG transcriptome map/fig_tb427vsgs_concat_whole-seq_32.png]

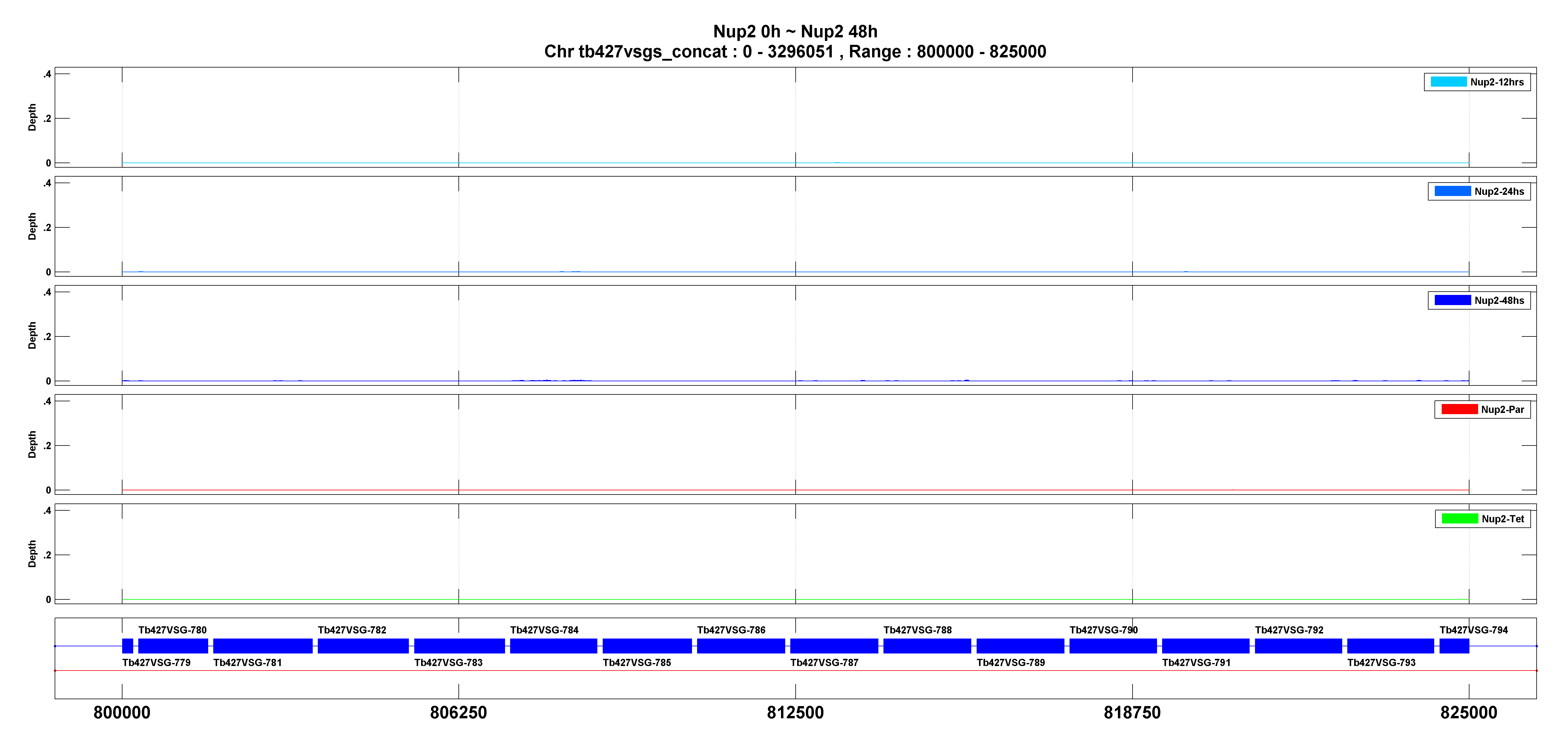

Supplement: SUPPLEMENTARY DATA [file supp_gkw751_nar-01100-x-2016-File026.zip › VSG transcriptome map/fig_tb427vsgs_concat_whole-seq_33.png]

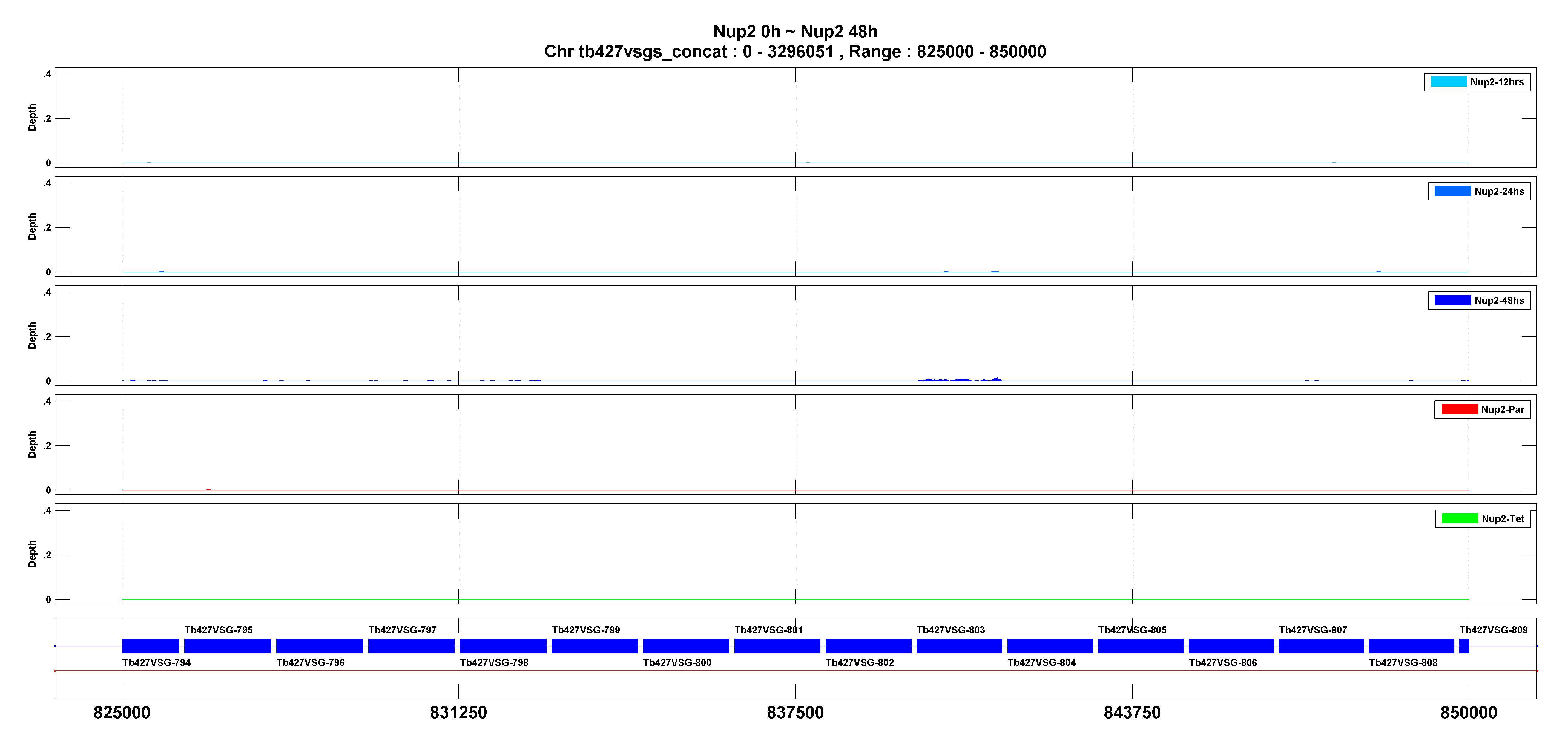

Supplement: SUPPLEMENTARY DATA [file supp_gkw751_nar-01100-x-2016-File026.zip › VSG transcriptome map/fig_tb427vsgs_concat_whole-seq_34.png]

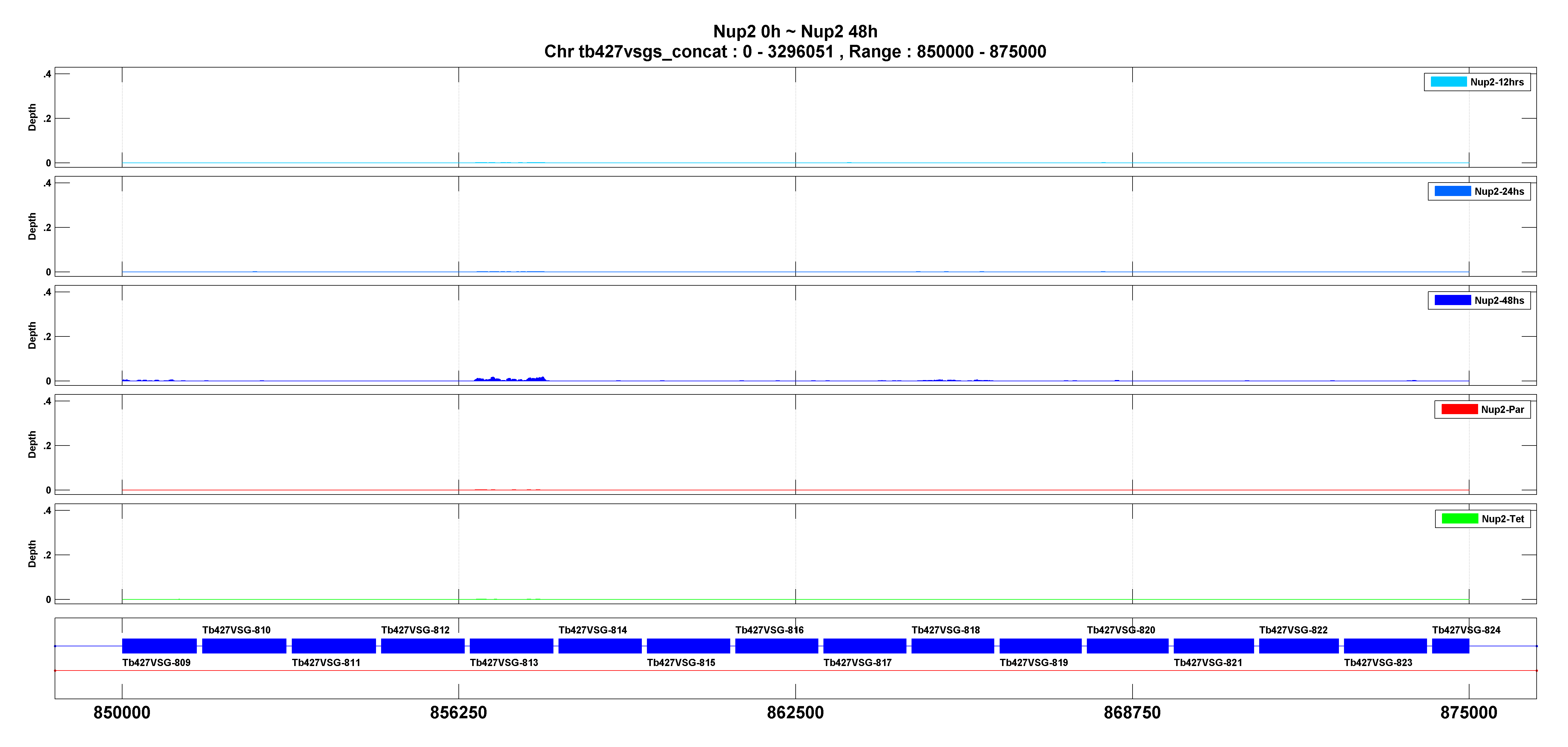

Supplement: SUPPLEMENTARY DATA [file supp_gkw751_nar-01100-x-2016-File026.zip › VSG transcriptome map/fig_tb427vsgs_concat_whole-seq_35.png]

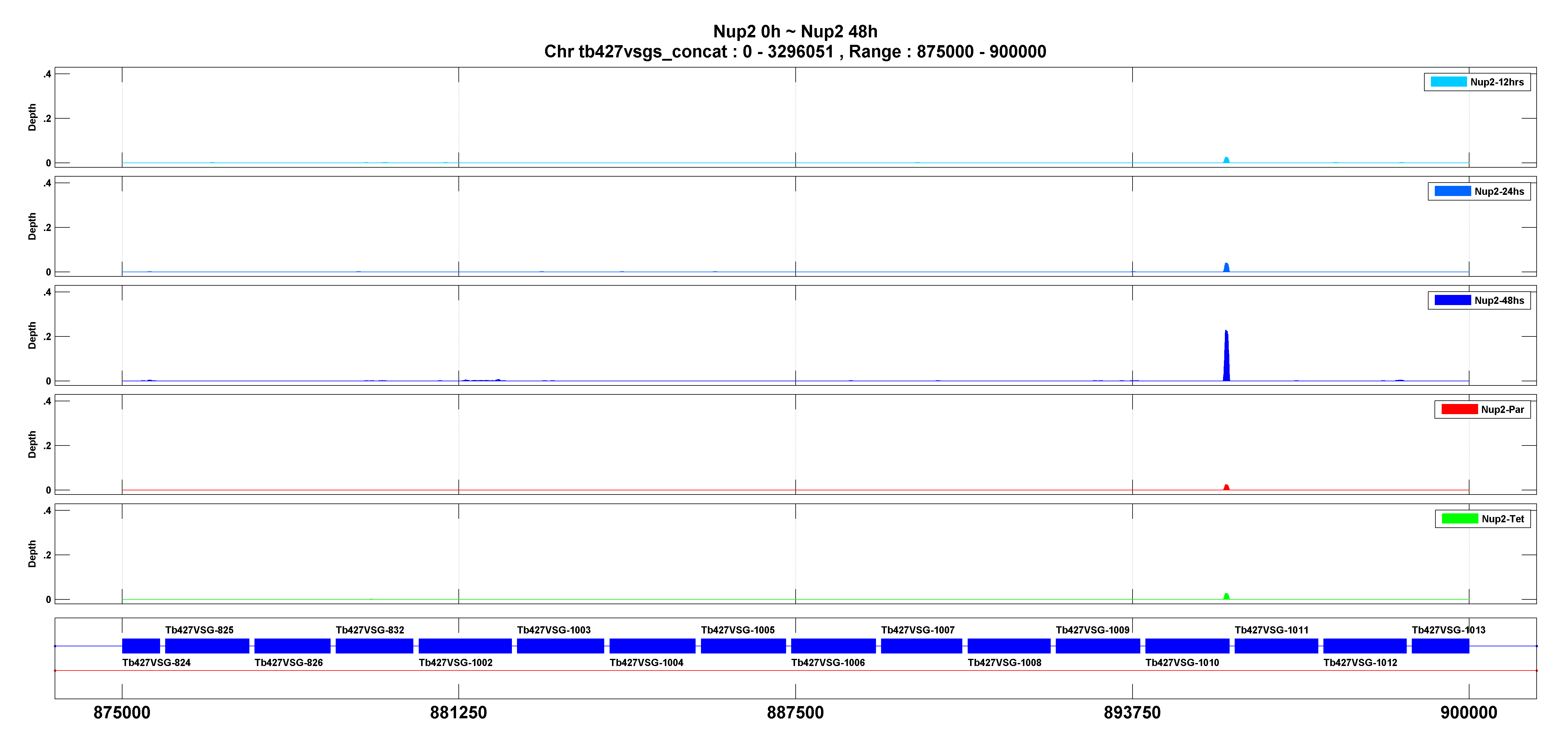

Supplement: SUPPLEMENTARY DATA [file supp_gkw751_nar-01100-x-2016-File026.zip › VSG transcriptome map/fig_tb427vsgs_concat_whole-seq_36.png]

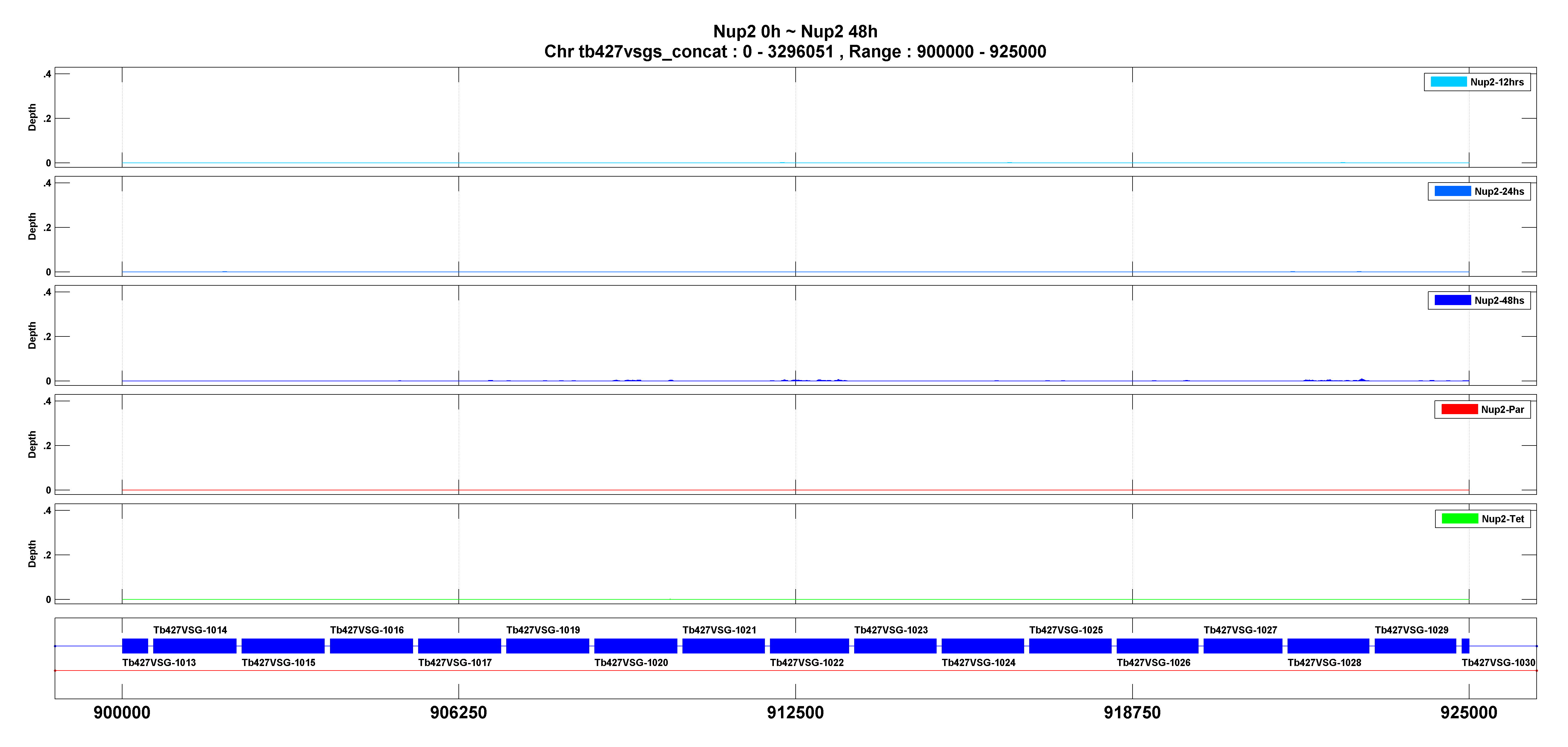

Supplement: SUPPLEMENTARY DATA [file supp_gkw751_nar-01100-x-2016-File026.zip › VSG transcriptome map/fig_tb427vsgs_concat_whole-seq_37.png]

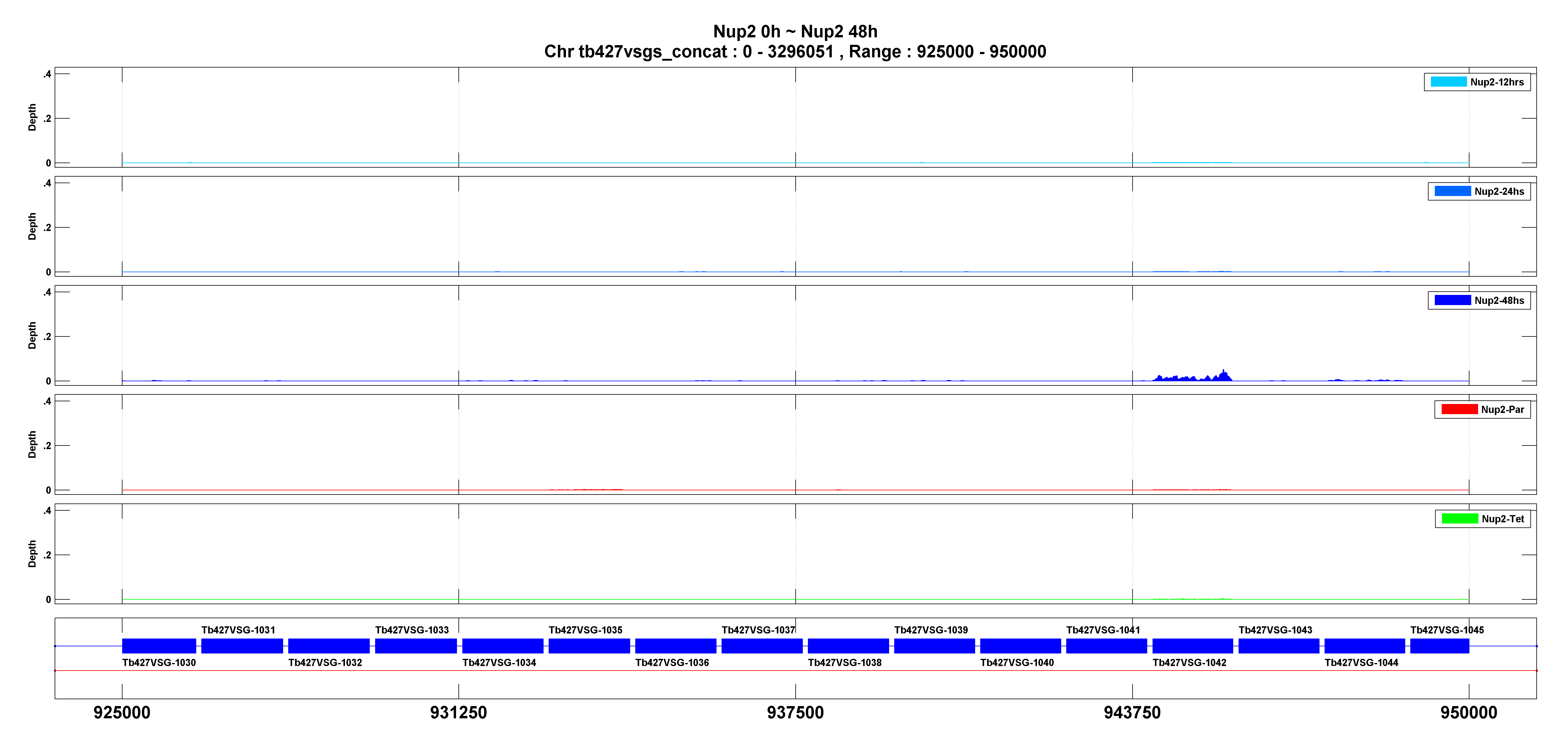

Supplement: SUPPLEMENTARY DATA [file supp_gkw751_nar-01100-x-2016-File026.zip › VSG transcriptome map/fig_tb427vsgs_concat_whole-seq_38.png]

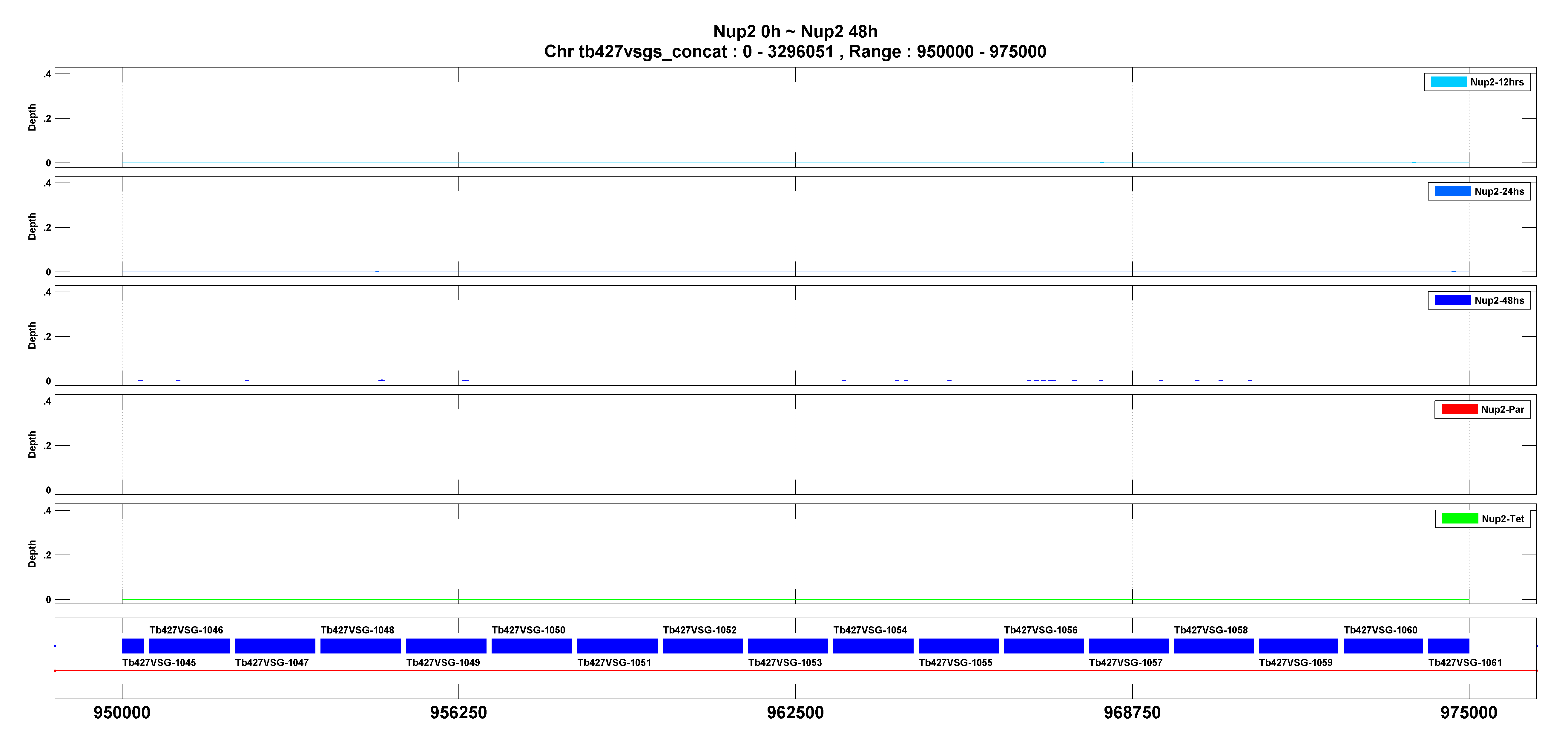

Supplement: SUPPLEMENTARY DATA [file supp_gkw751_nar-01100-x-2016-File026.zip › VSG transcriptome map/fig_tb427vsgs_concat_whole-seq_39.png]

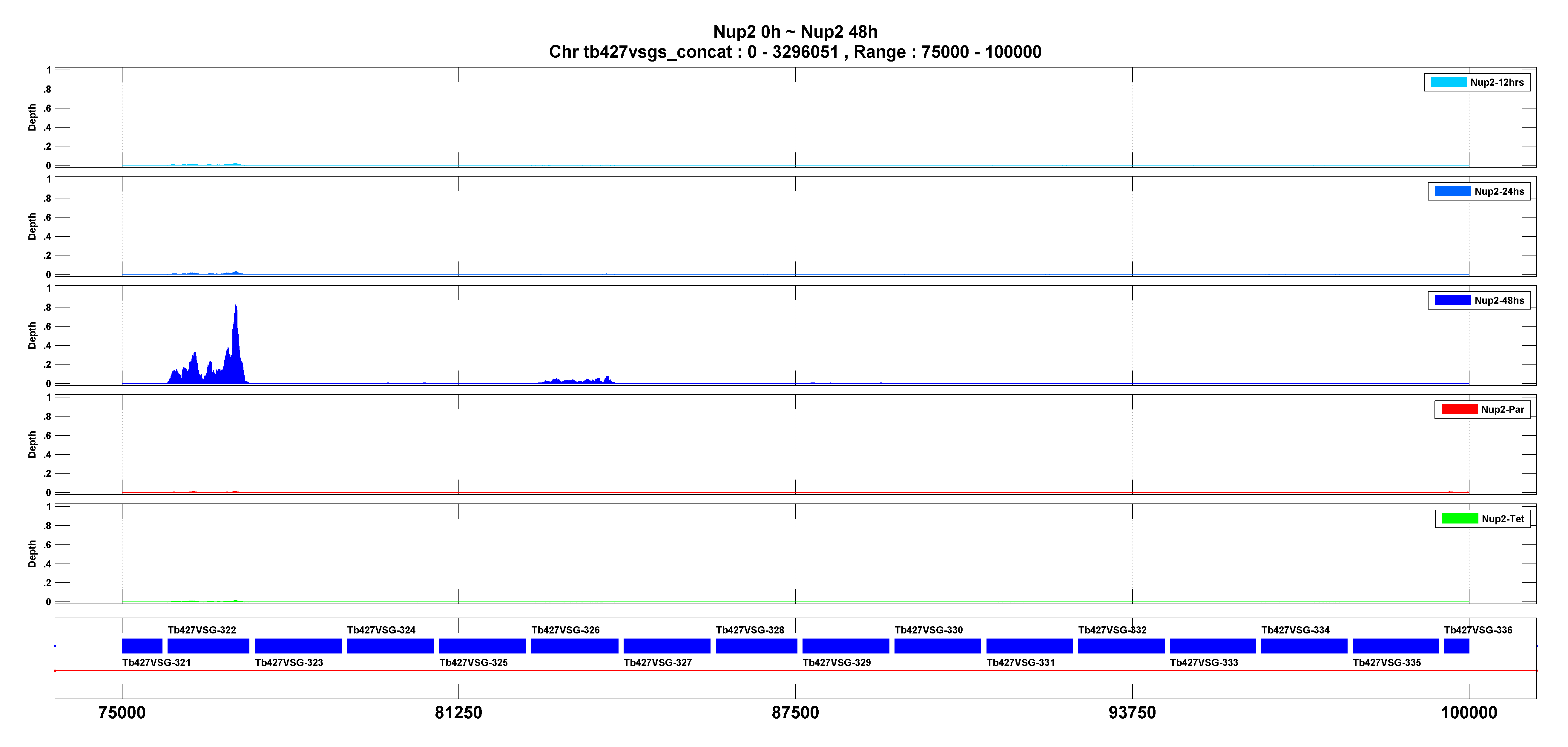

Supplement: SUPPLEMENTARY DATA [file supp_gkw751_nar-01100-x-2016-File026.zip › VSG transcriptome map/fig_tb427vsgs_concat_whole-seq_4.png]

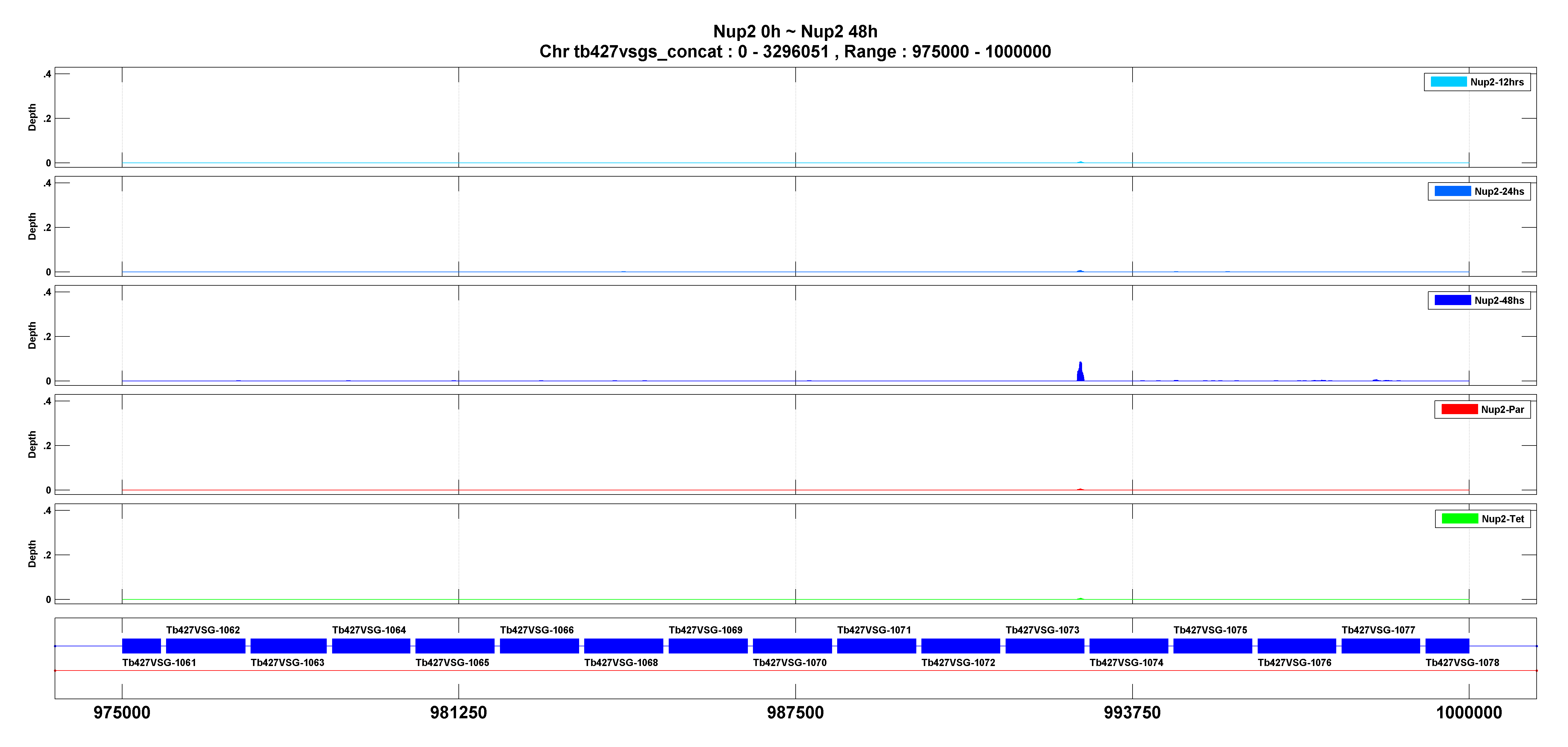

Supplement: SUPPLEMENTARY DATA [file supp_gkw751_nar-01100-x-2016-File026.zip › VSG transcriptome map/fig_tb427vsgs_concat_whole-seq_40.png]

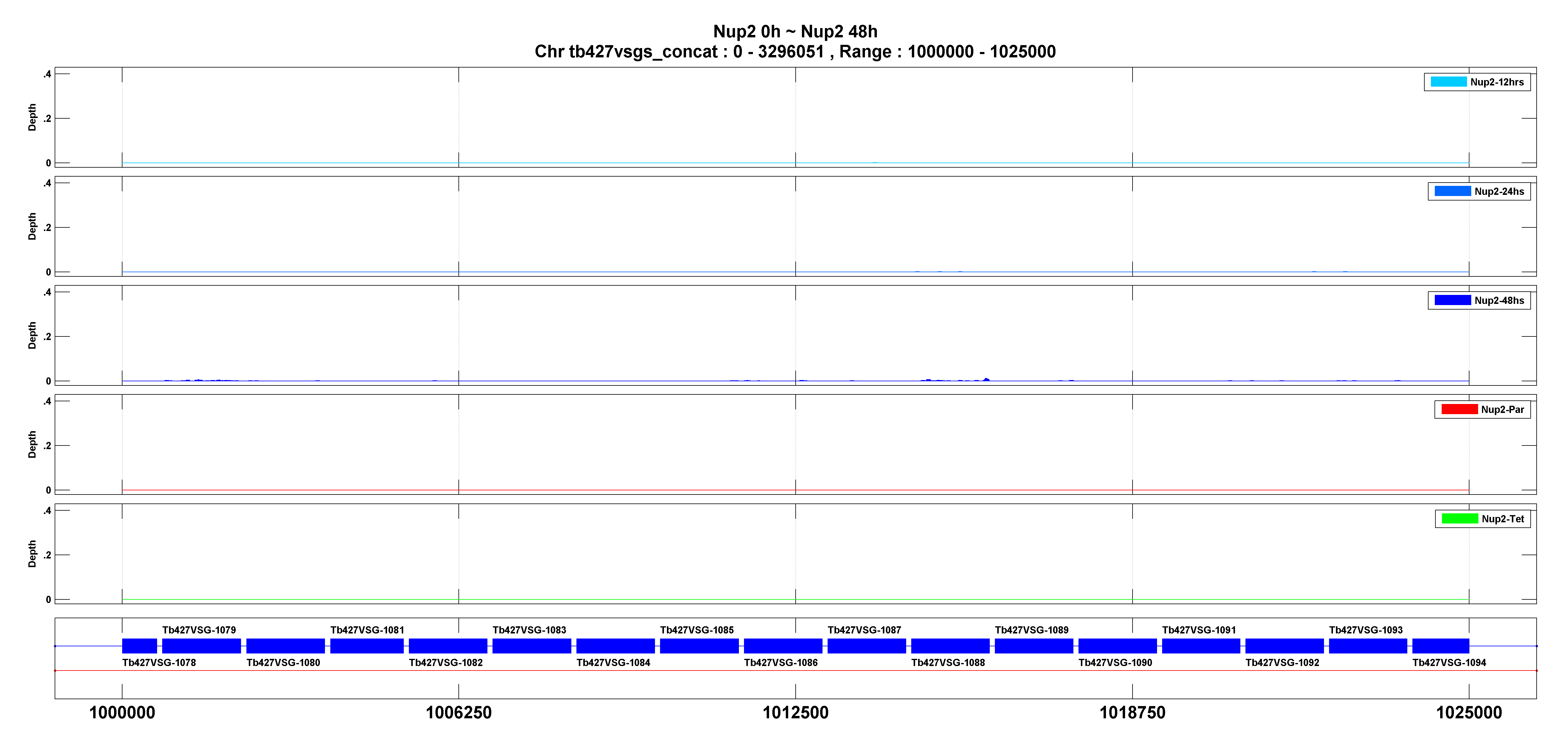

Supplement: SUPPLEMENTARY DATA [file supp_gkw751_nar-01100-x-2016-File026.zip › VSG transcriptome map/fig_tb427vsgs_concat_whole-seq_41.png]

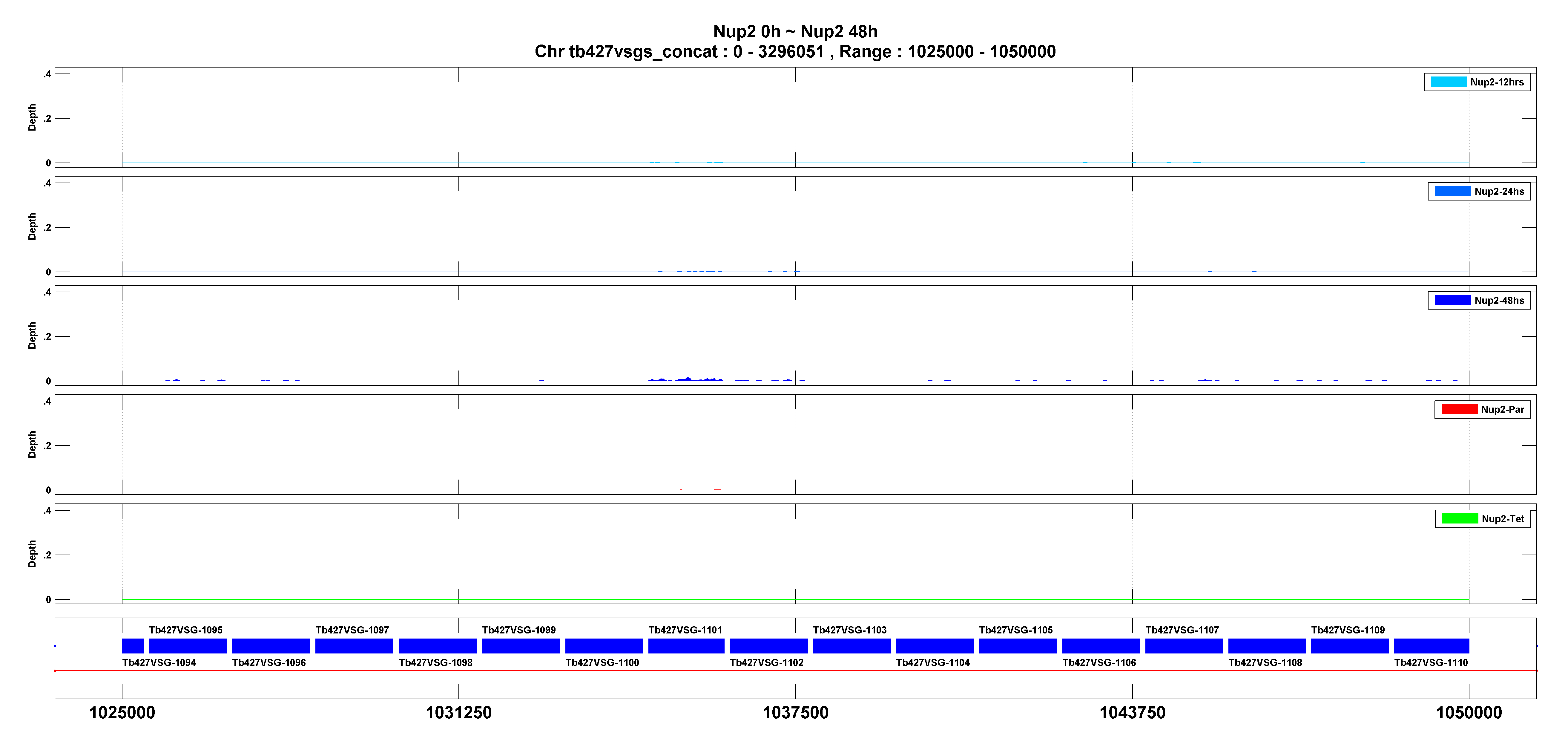

Supplement: SUPPLEMENTARY DATA [file supp_gkw751_nar-01100-x-2016-File026.zip › VSG transcriptome map/fig_tb427vsgs_concat_whole-seq_42.png]

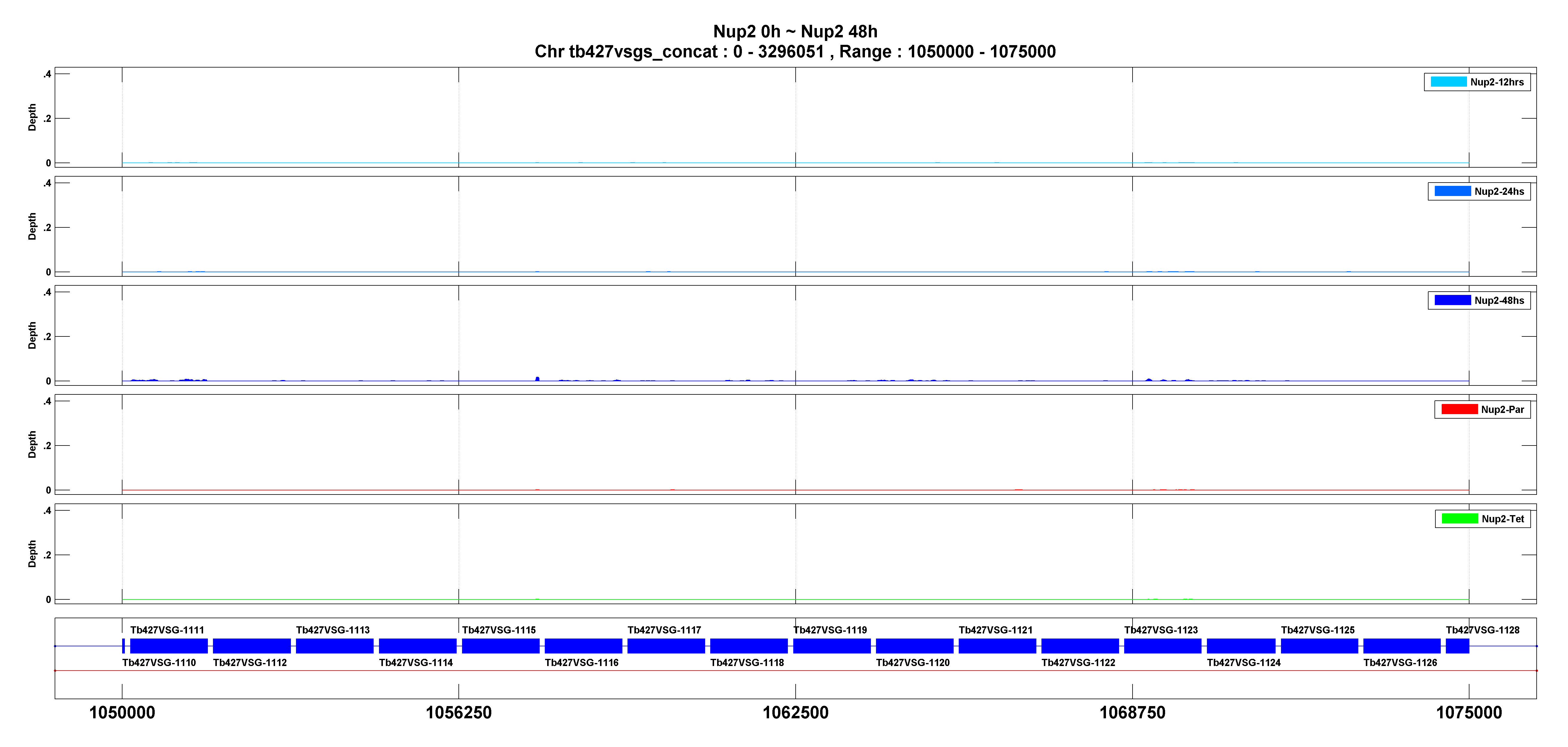

Supplement: SUPPLEMENTARY DATA [file supp_gkw751_nar-01100-x-2016-File026.zip › VSG transcriptome map/fig_tb427vsgs_concat_whole-seq_43.png]

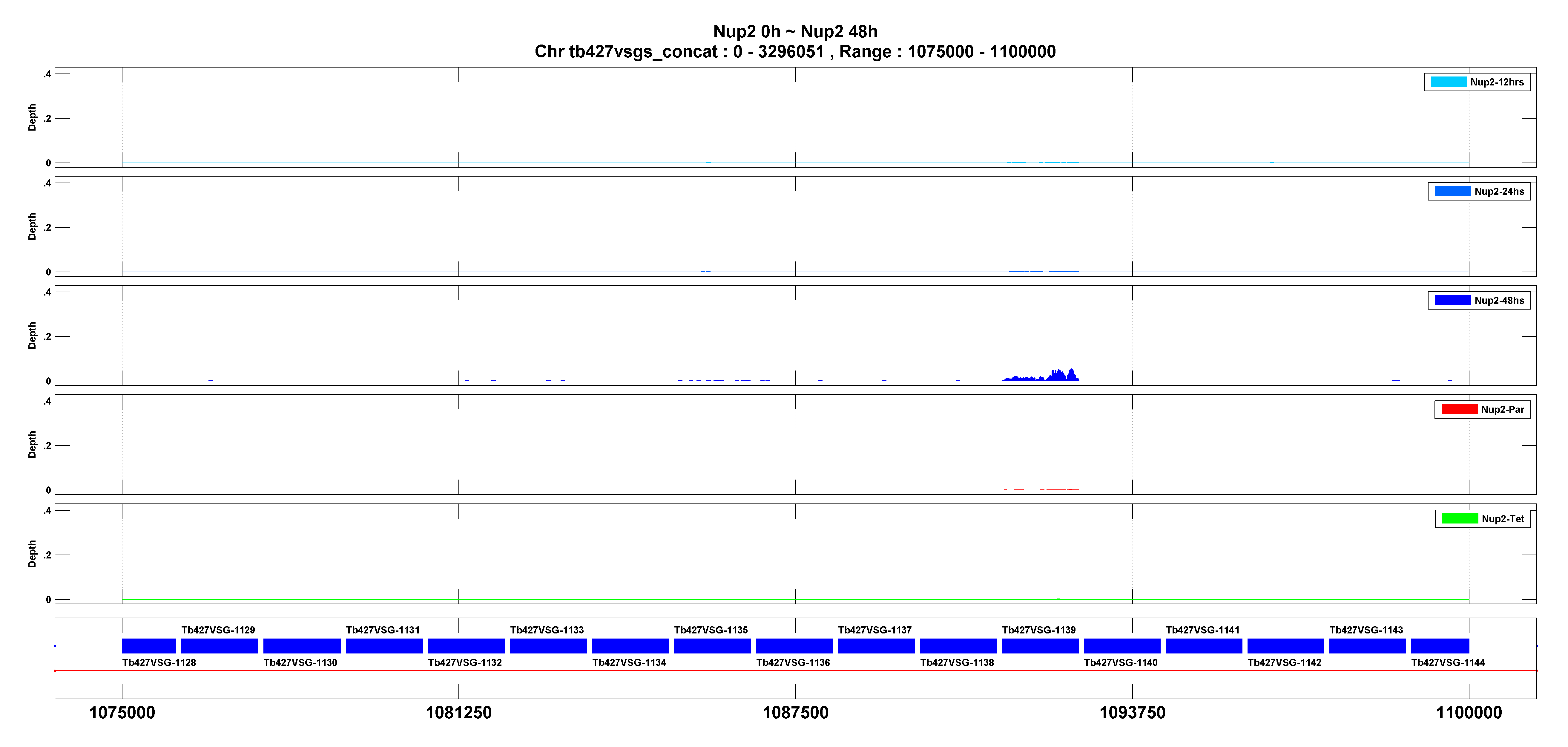

Supplement: SUPPLEMENTARY DATA [file supp_gkw751_nar-01100-x-2016-File026.zip › VSG transcriptome map/fig_tb427vsgs_concat_whole-seq_44.png]

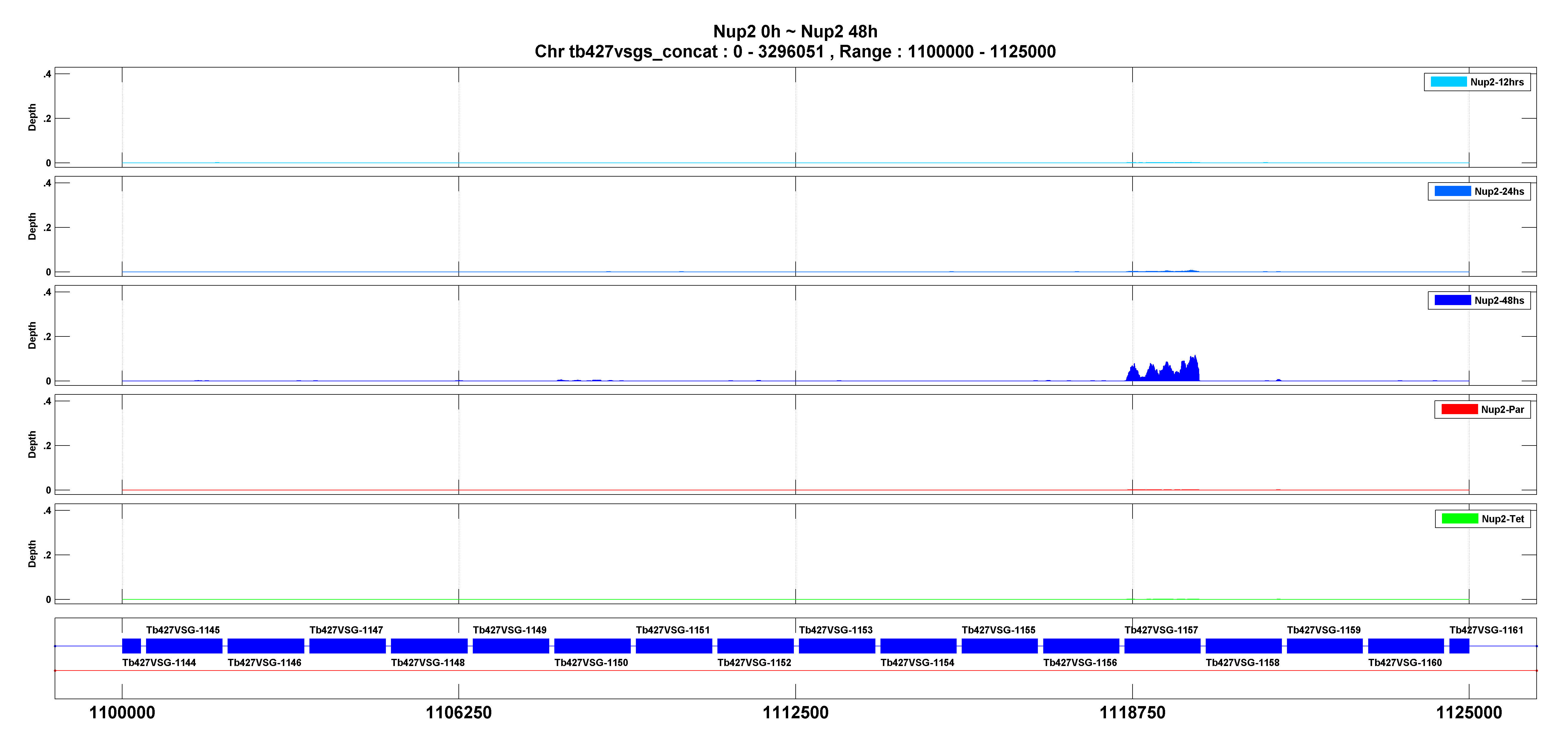

Supplement: SUPPLEMENTARY DATA [file supp_gkw751_nar-01100-x-2016-File026.zip › VSG transcriptome map/fig_tb427vsgs_concat_whole-seq_45.png]

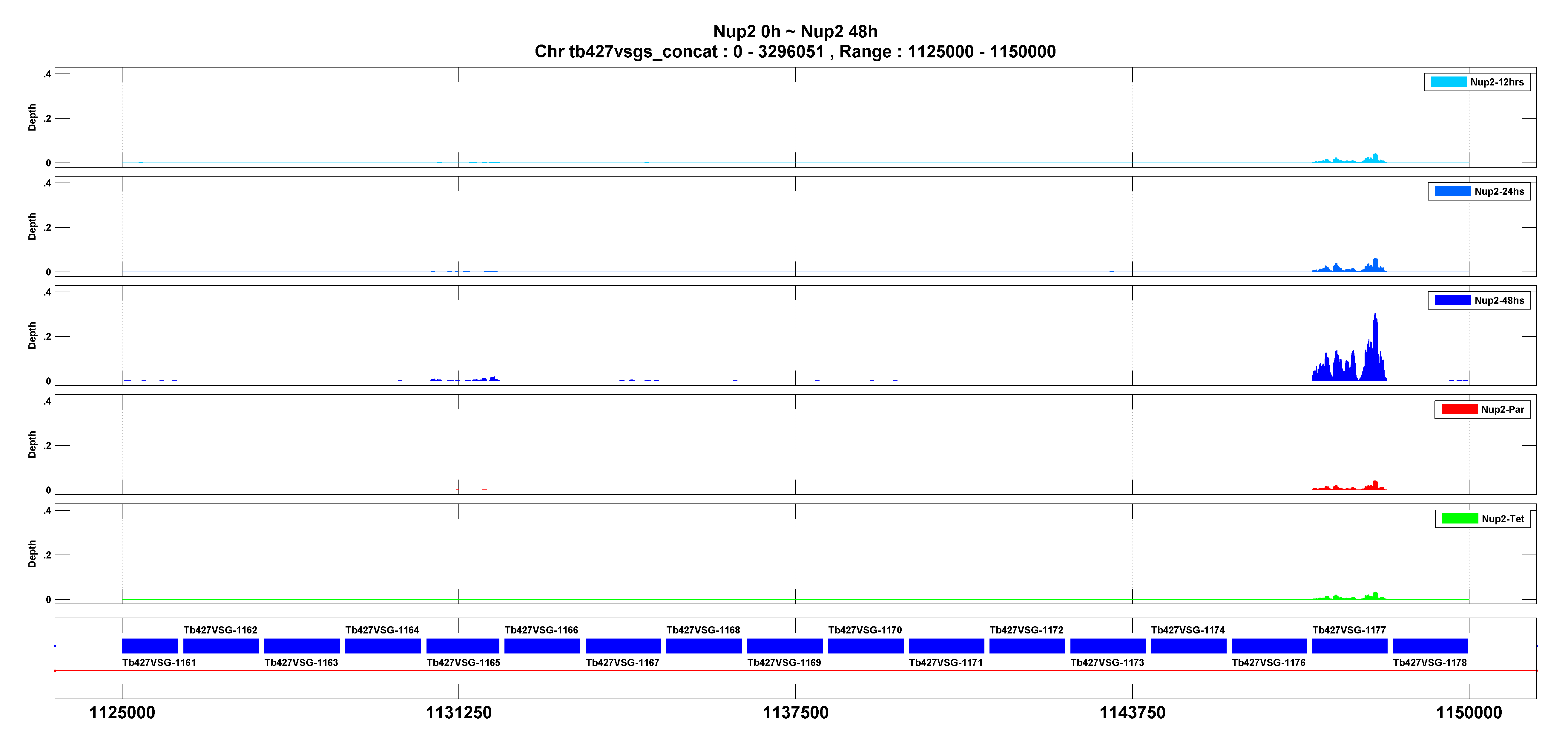

Supplement: SUPPLEMENTARY DATA [file supp_gkw751_nar-01100-x-2016-File026.zip › VSG transcriptome map/fig_tb427vsgs_concat_whole-seq_46.png]

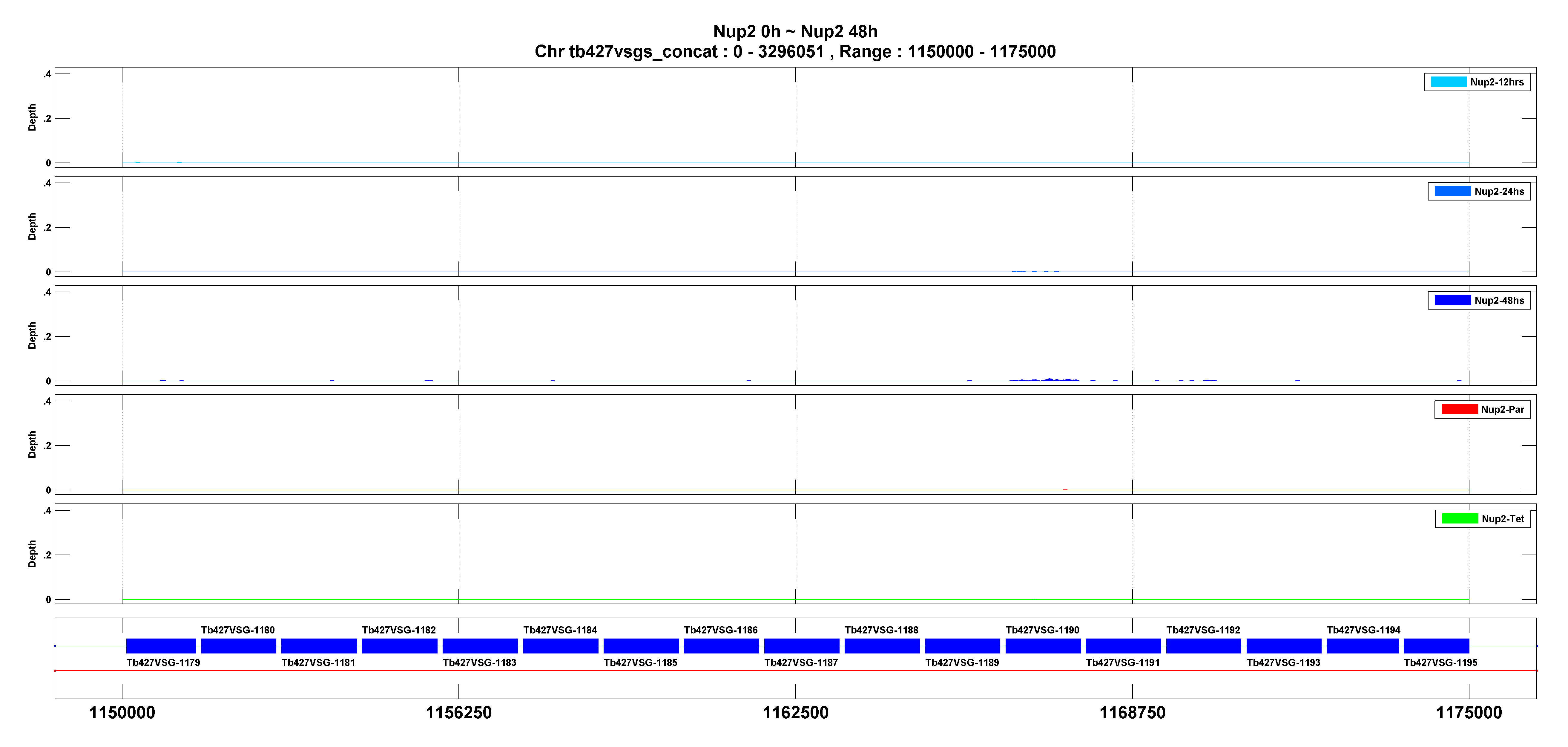

Supplement: SUPPLEMENTARY DATA [file supp_gkw751_nar-01100-x-2016-File026.zip › VSG transcriptome map/fig_tb427vsgs_concat_whole-seq_47.png]

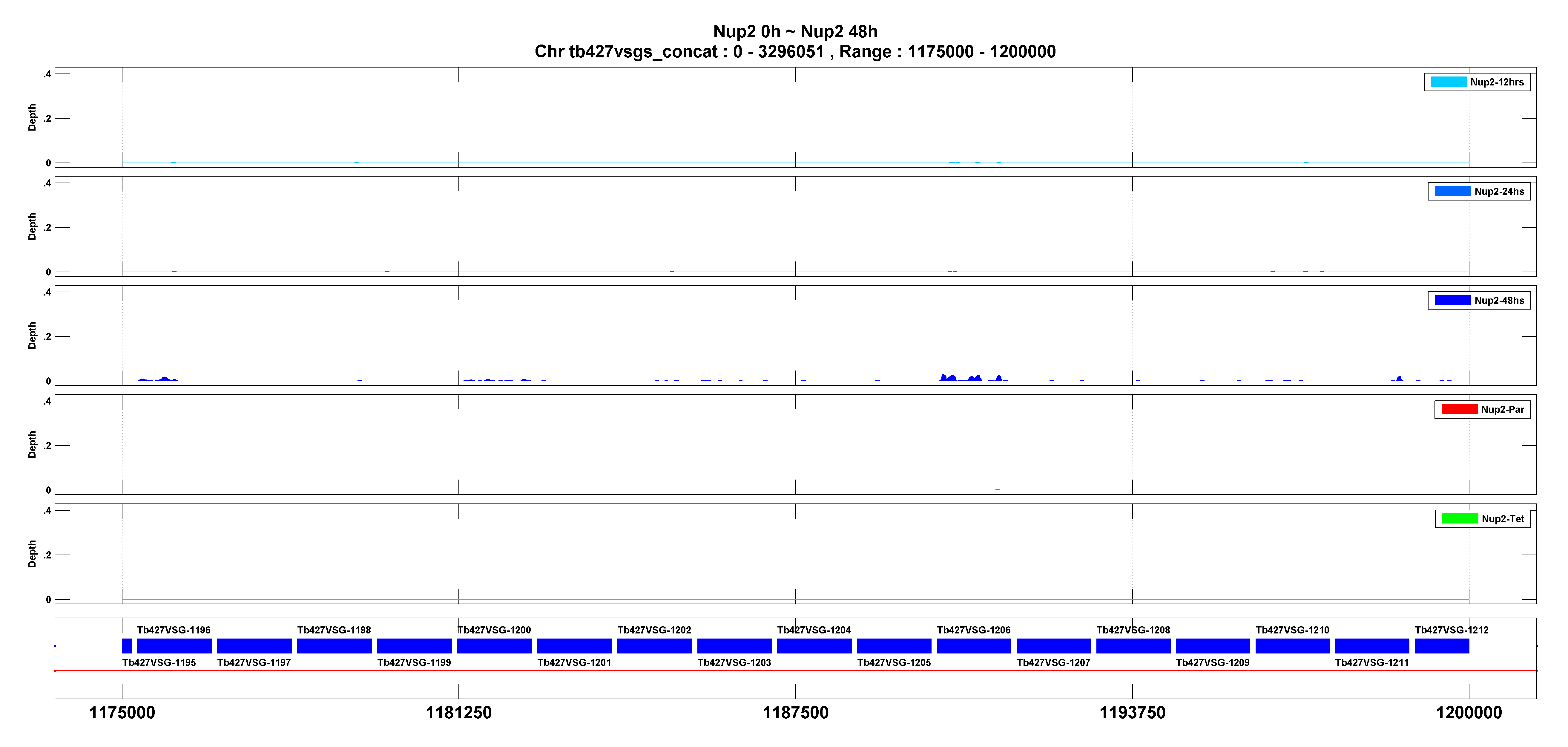

Supplement: SUPPLEMENTARY DATA [file supp_gkw751_nar-01100-x-2016-File026.zip › VSG transcriptome map/fig_tb427vsgs_concat_whole-seq_48.png]

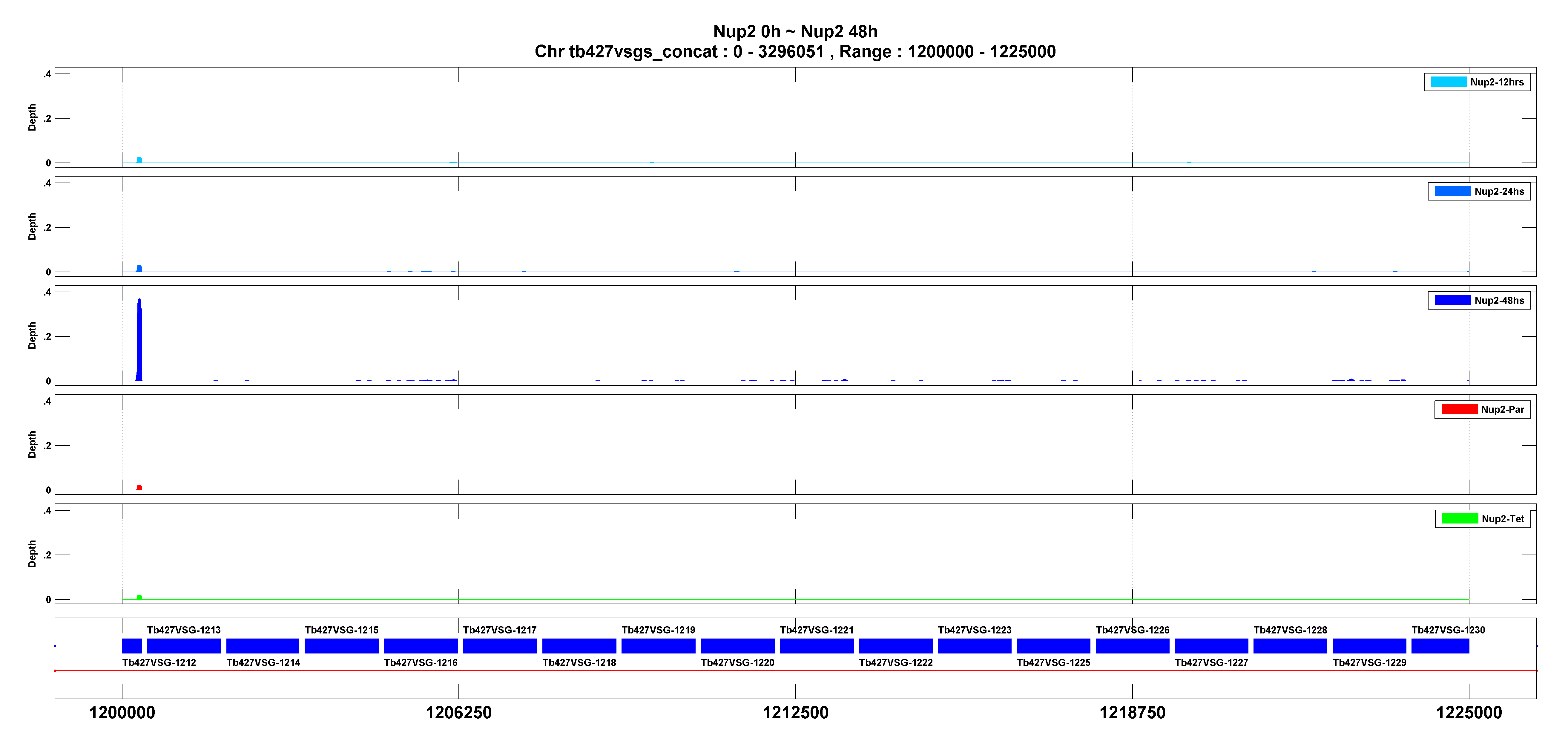

Supplement: SUPPLEMENTARY DATA [file supp_gkw751_nar-01100-x-2016-File026.zip › VSG transcriptome map/fig_tb427vsgs_concat_whole-seq_49.png]

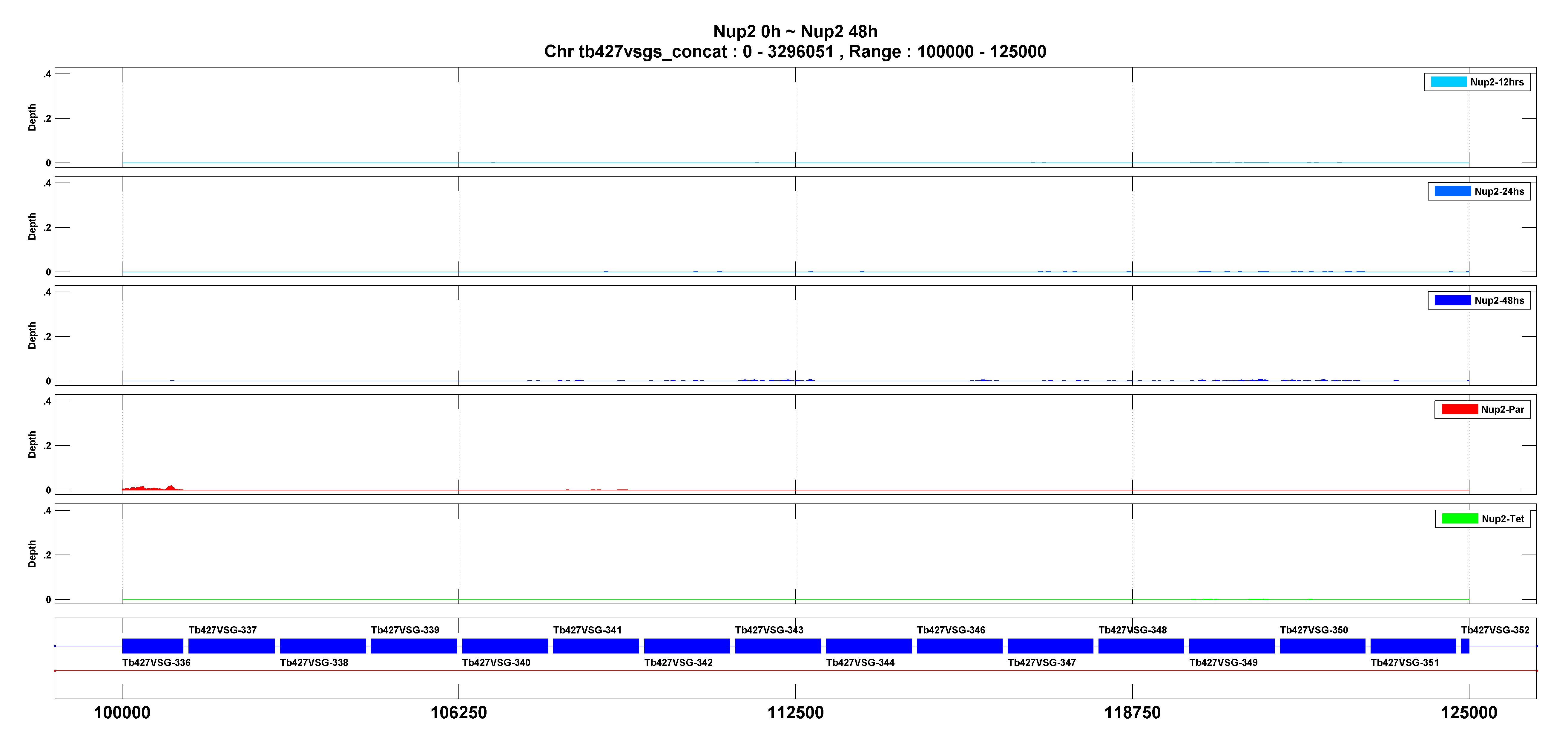

Supplement: SUPPLEMENTARY DATA [file supp_gkw751_nar-01100-x-2016-File026.zip › VSG transcriptome map/fig_tb427vsgs_concat_whole-seq_5.png]

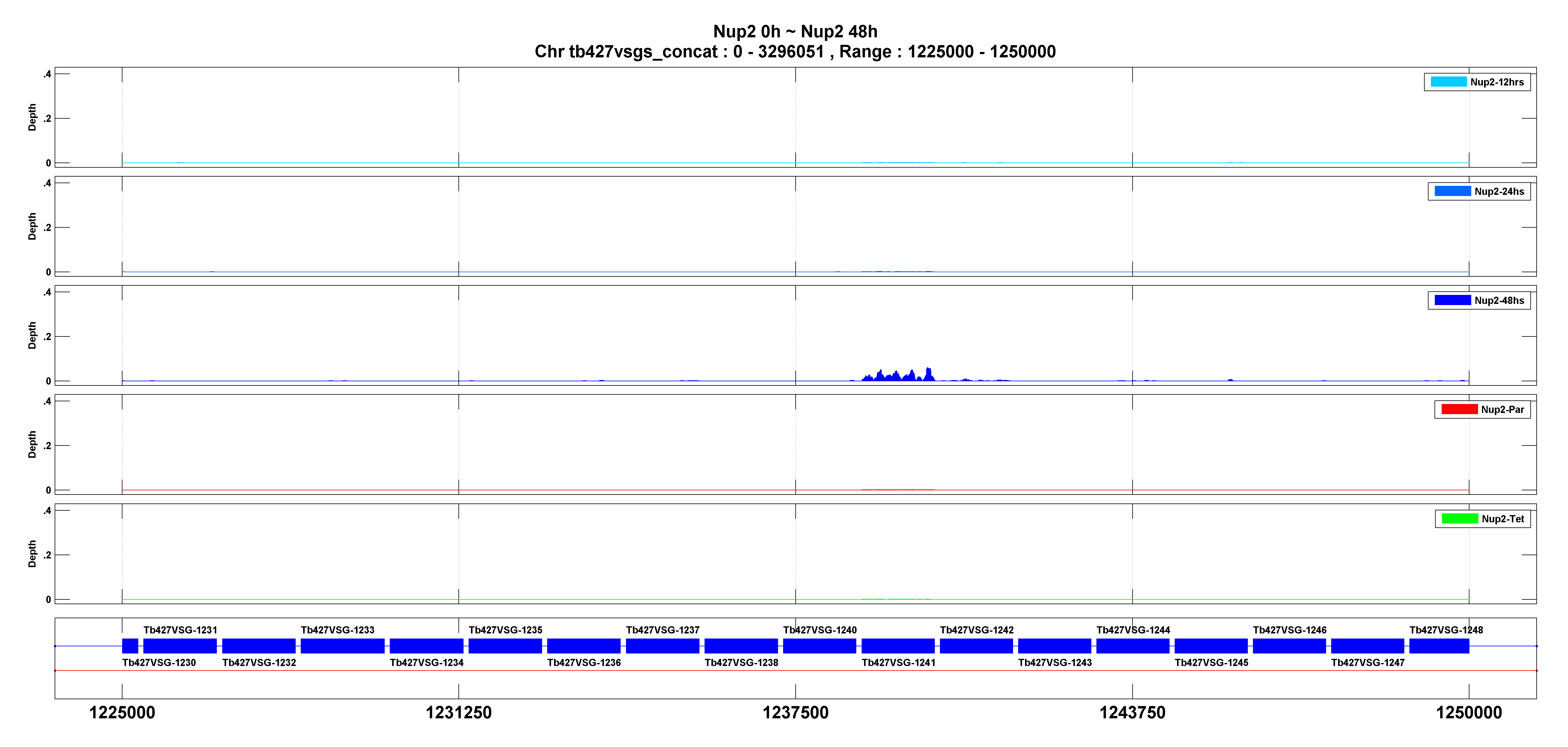

Supplement: SUPPLEMENTARY DATA [file supp_gkw751_nar-01100-x-2016-File026.zip › VSG transcriptome map/fig_tb427vsgs_concat_whole-seq_50.png]

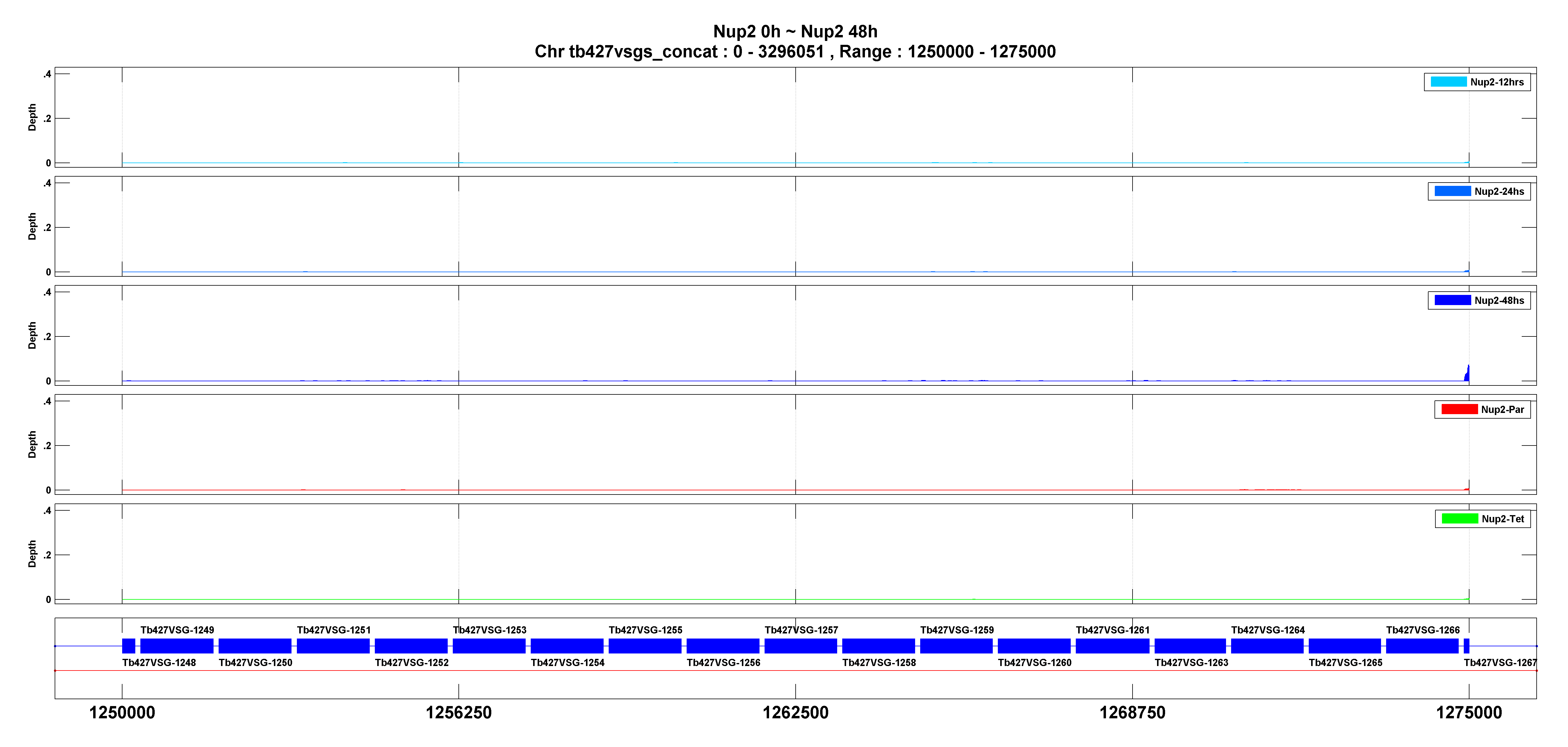

Supplement: SUPPLEMENTARY DATA [file supp_gkw751_nar-01100-x-2016-File026.zip › VSG transcriptome map/fig_tb427vsgs_concat_whole-seq_51.png]

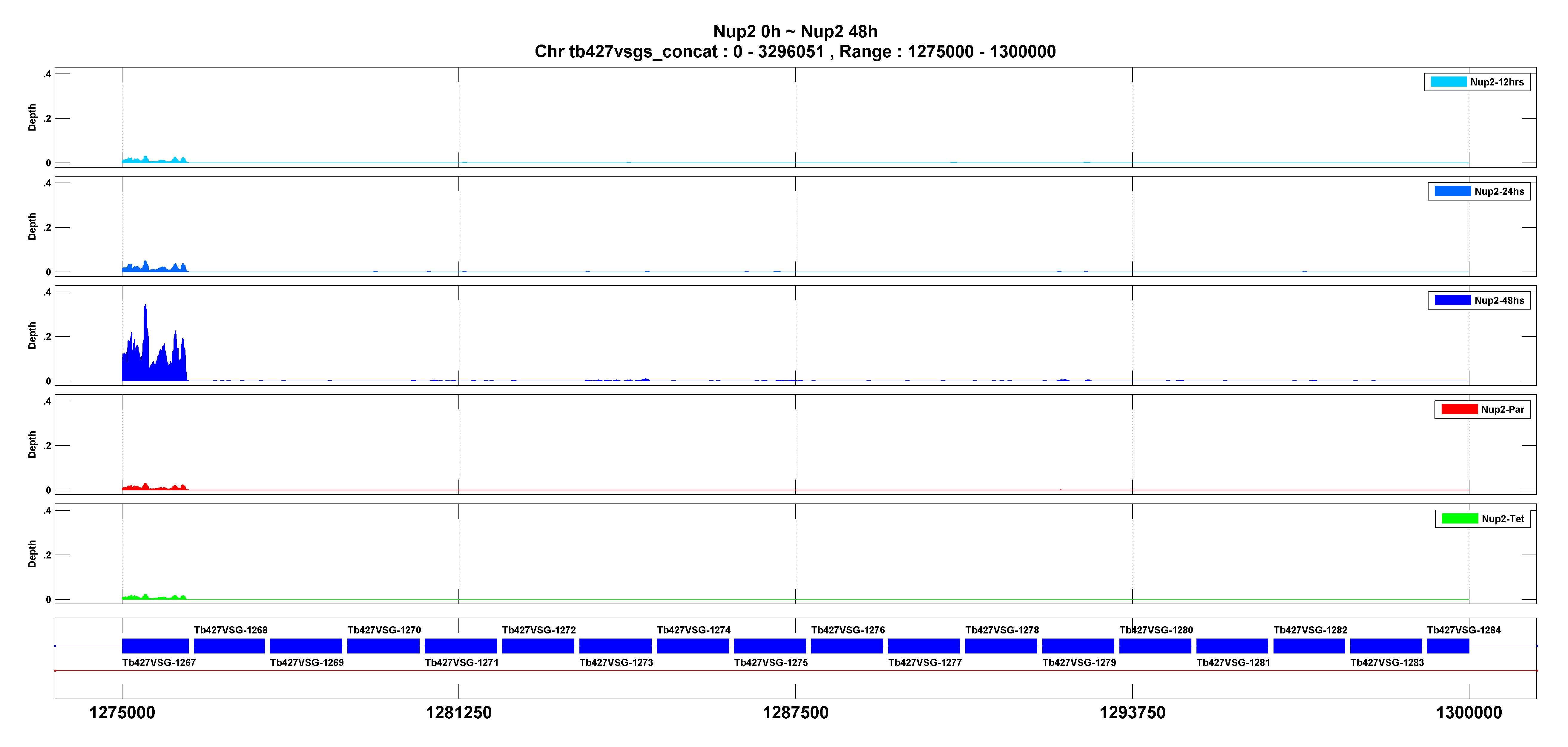

Supplement: SUPPLEMENTARY DATA [file supp_gkw751_nar-01100-x-2016-File026.zip › VSG transcriptome map/fig_tb427vsgs_concat_whole-seq_52.png]

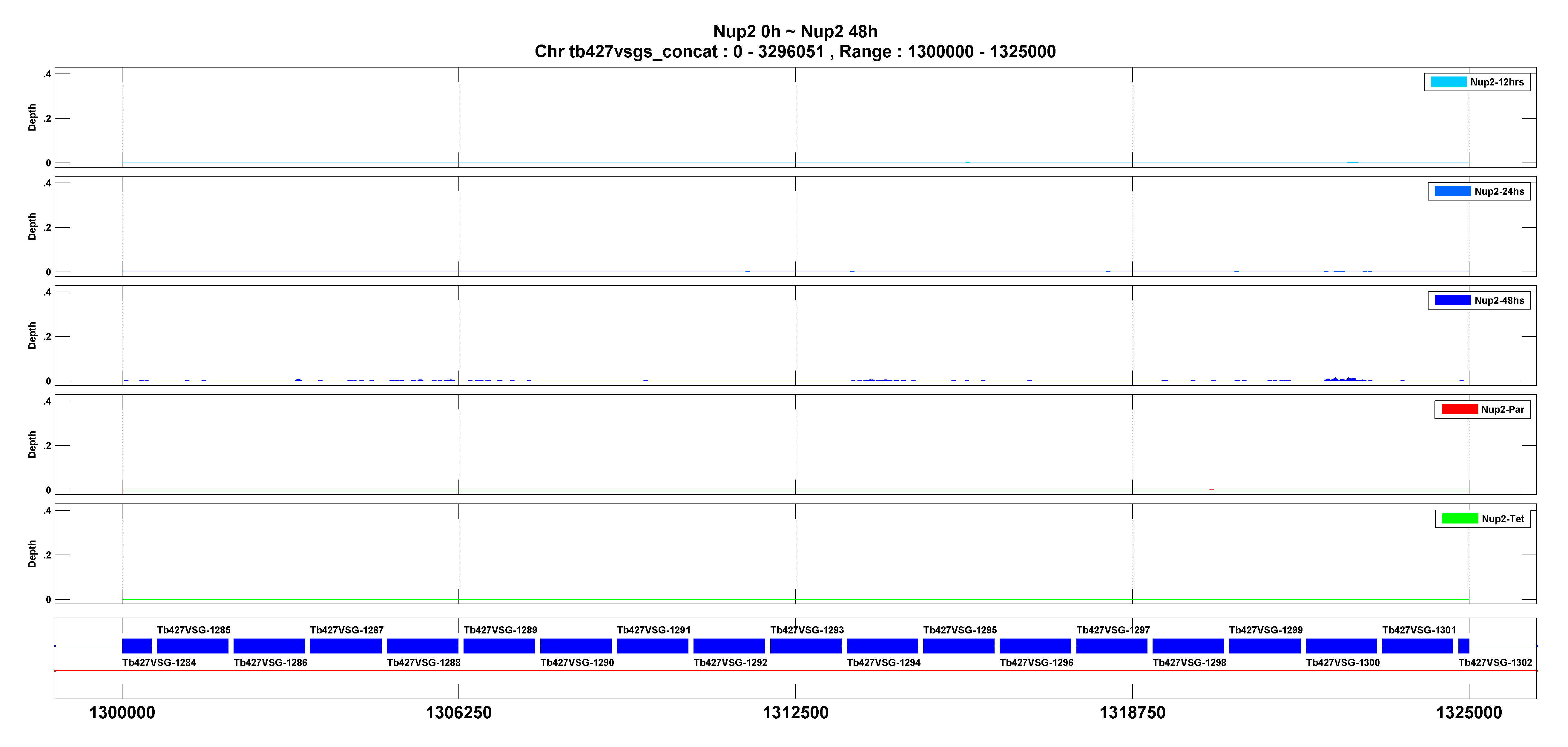

Supplement: SUPPLEMENTARY DATA [file supp_gkw751_nar-01100-x-2016-File026.zip › VSG transcriptome map/fig_tb427vsgs_concat_whole-seq_53.png]

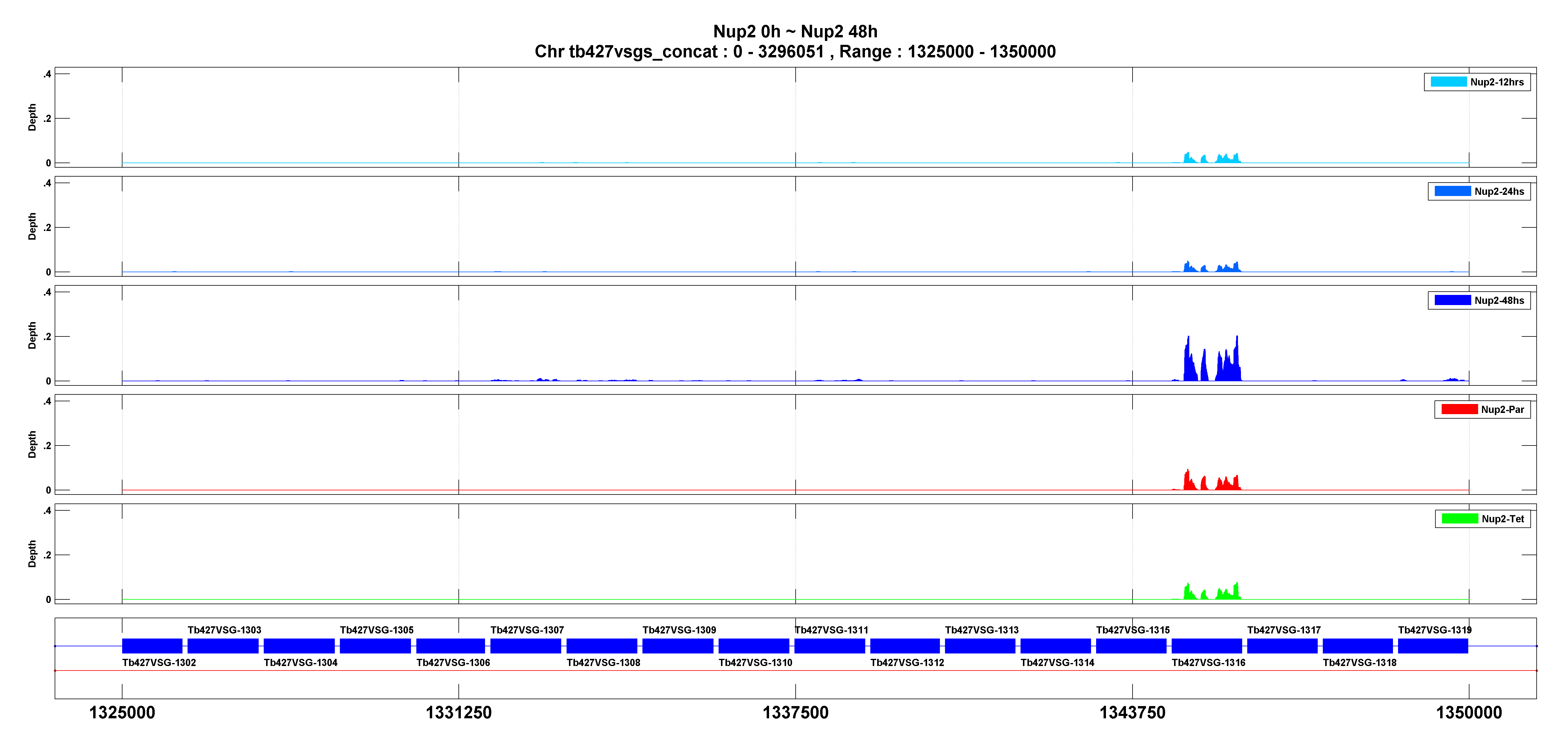

Supplement: SUPPLEMENTARY DATA [file supp_gkw751_nar-01100-x-2016-File026.zip › VSG transcriptome map/fig_tb427vsgs_concat_whole-seq_54.png]

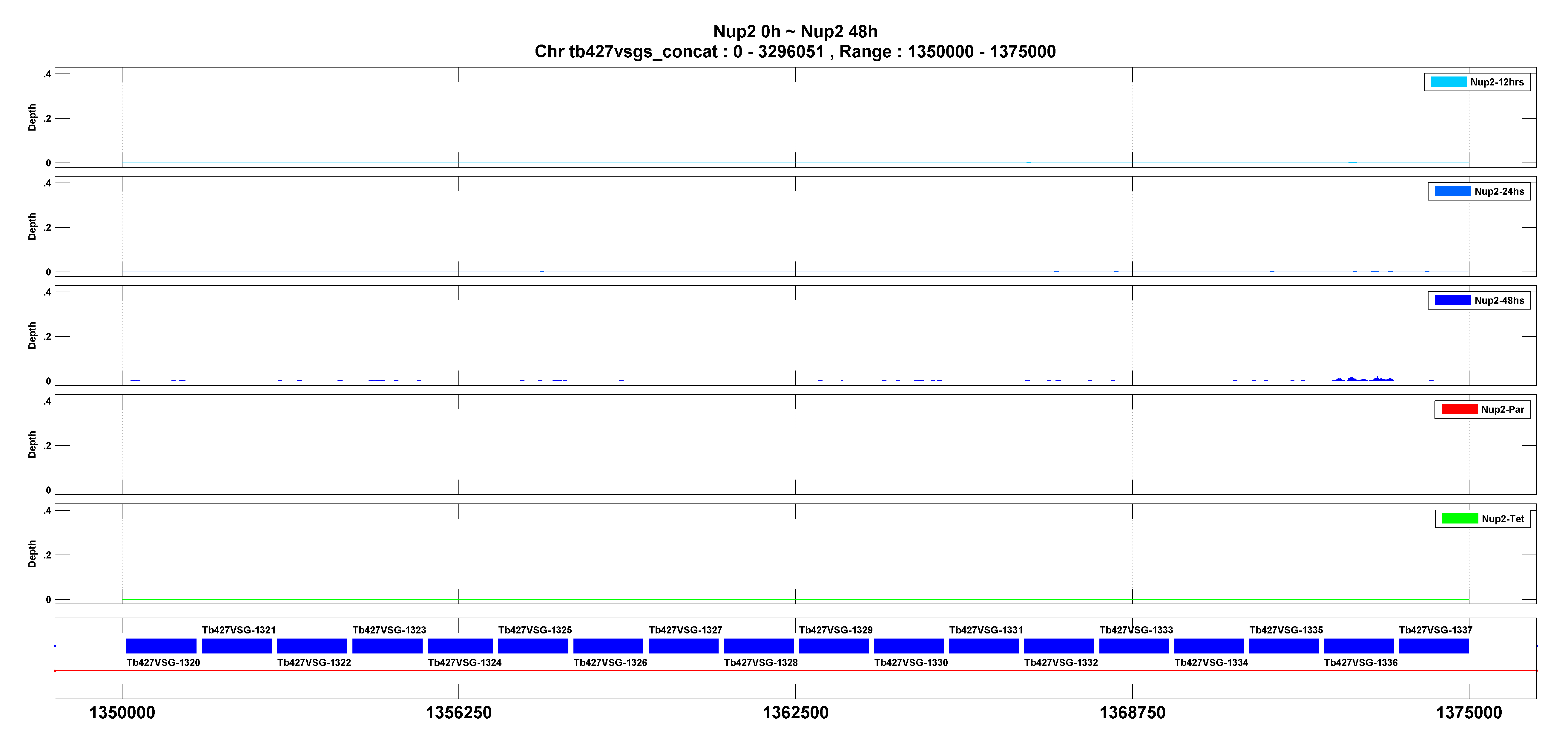

Supplement: SUPPLEMENTARY DATA [file supp_gkw751_nar-01100-x-2016-File026.zip › VSG transcriptome map/fig_tb427vsgs_concat_whole-seq_55.png]

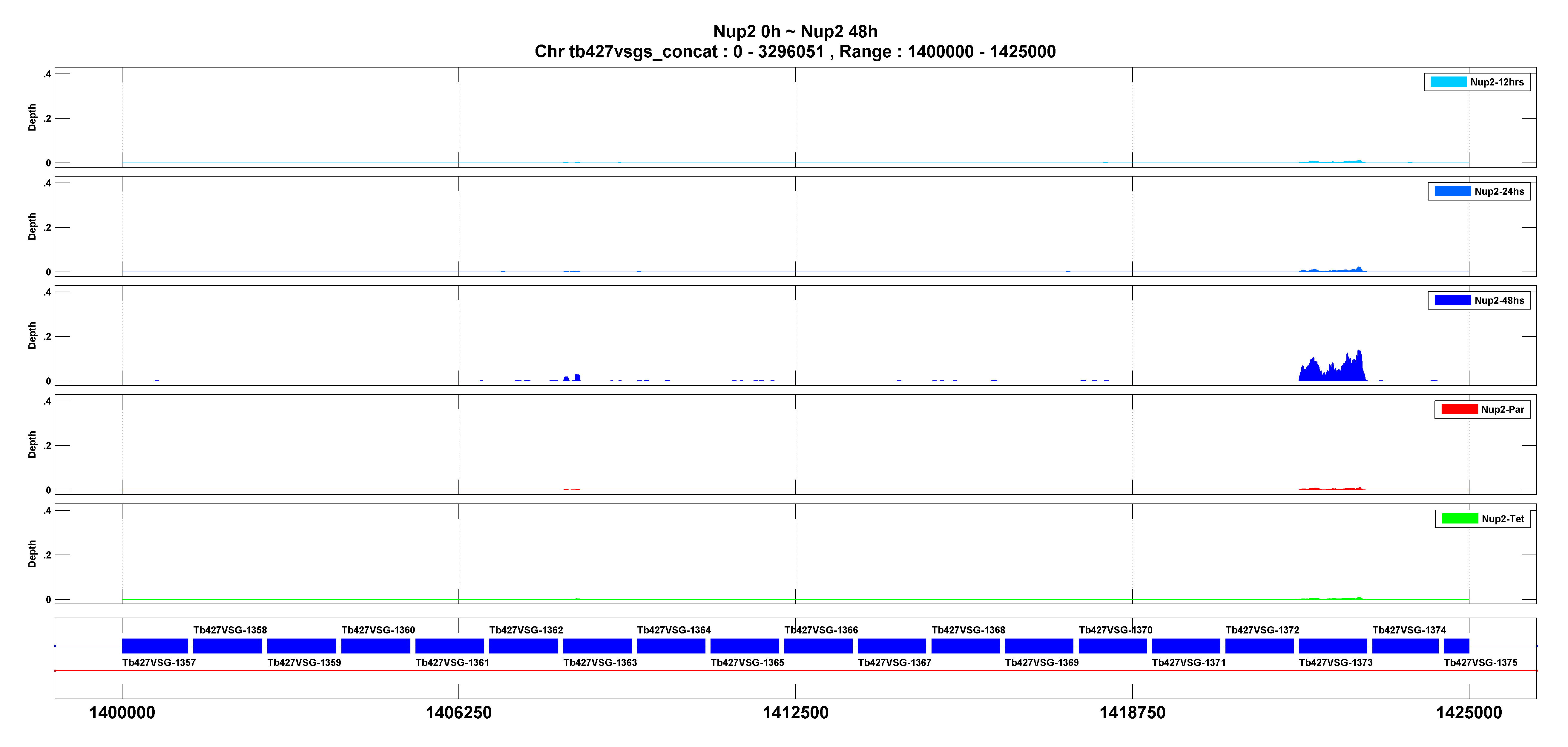

Supplement: SUPPLEMENTARY DATA [file supp_gkw751_nar-01100-x-2016-File026.zip › VSG transcriptome map/fig_tb427vsgs_concat_whole-seq_57.png]

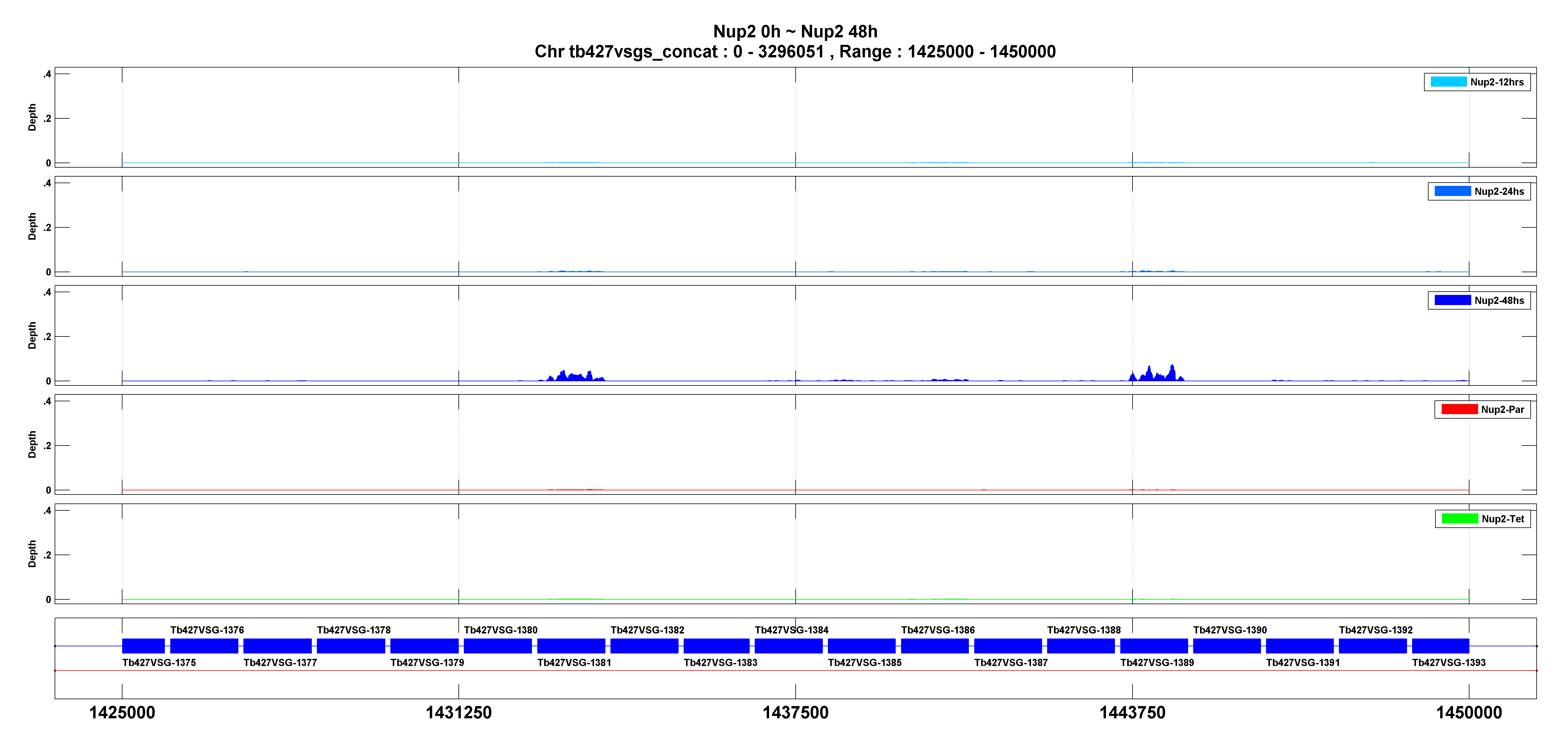

Supplement: SUPPLEMENTARY DATA [file supp_gkw751_nar-01100-x-2016-File026.zip › VSG transcriptome map/fig_tb427vsgs_concat_whole-seq_58.png]

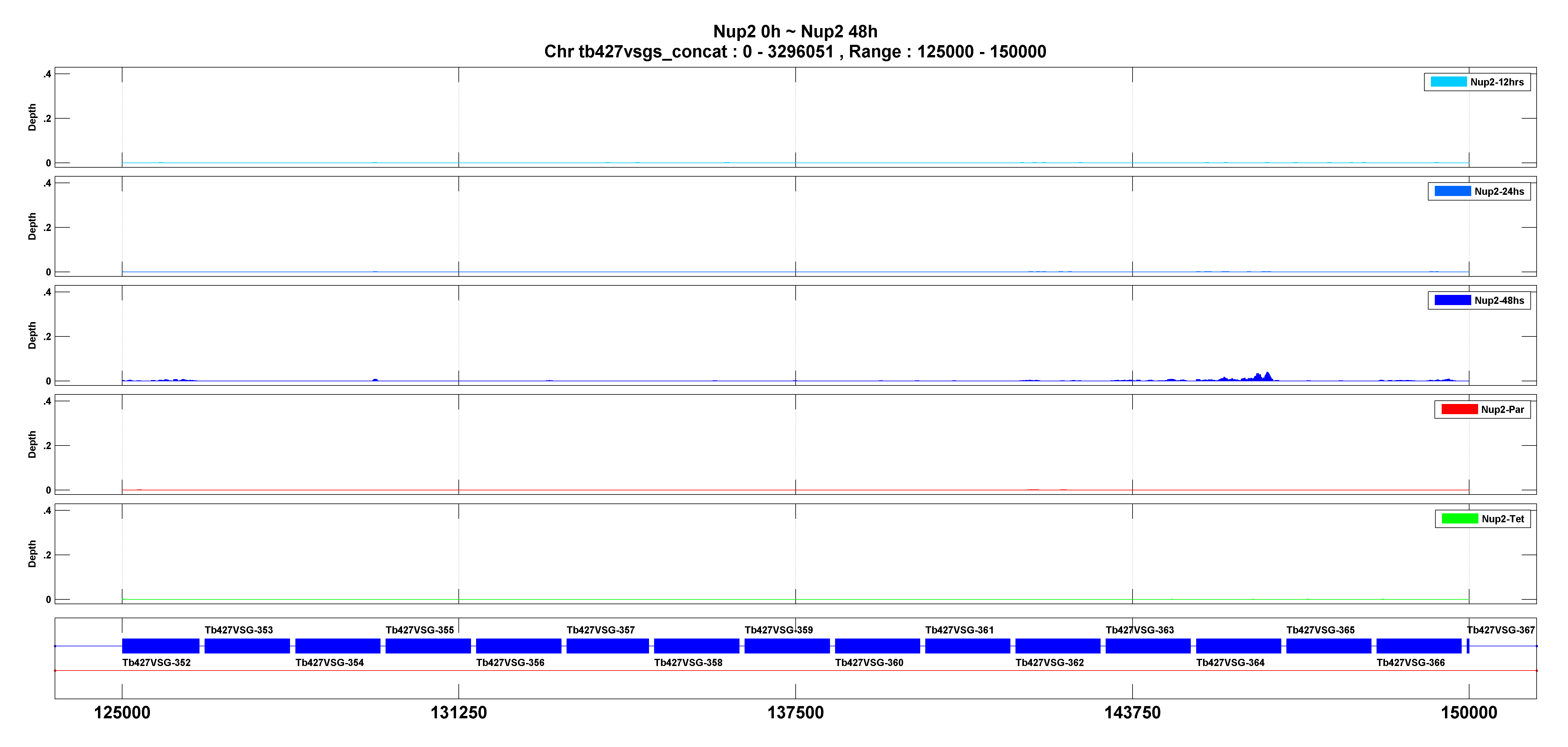

Supplement: SUPPLEMENTARY DATA [file supp_gkw751_nar-01100-x-2016-File026.zip › VSG transcriptome map/fig_tb427vsgs_concat_whole-seq_6.png]

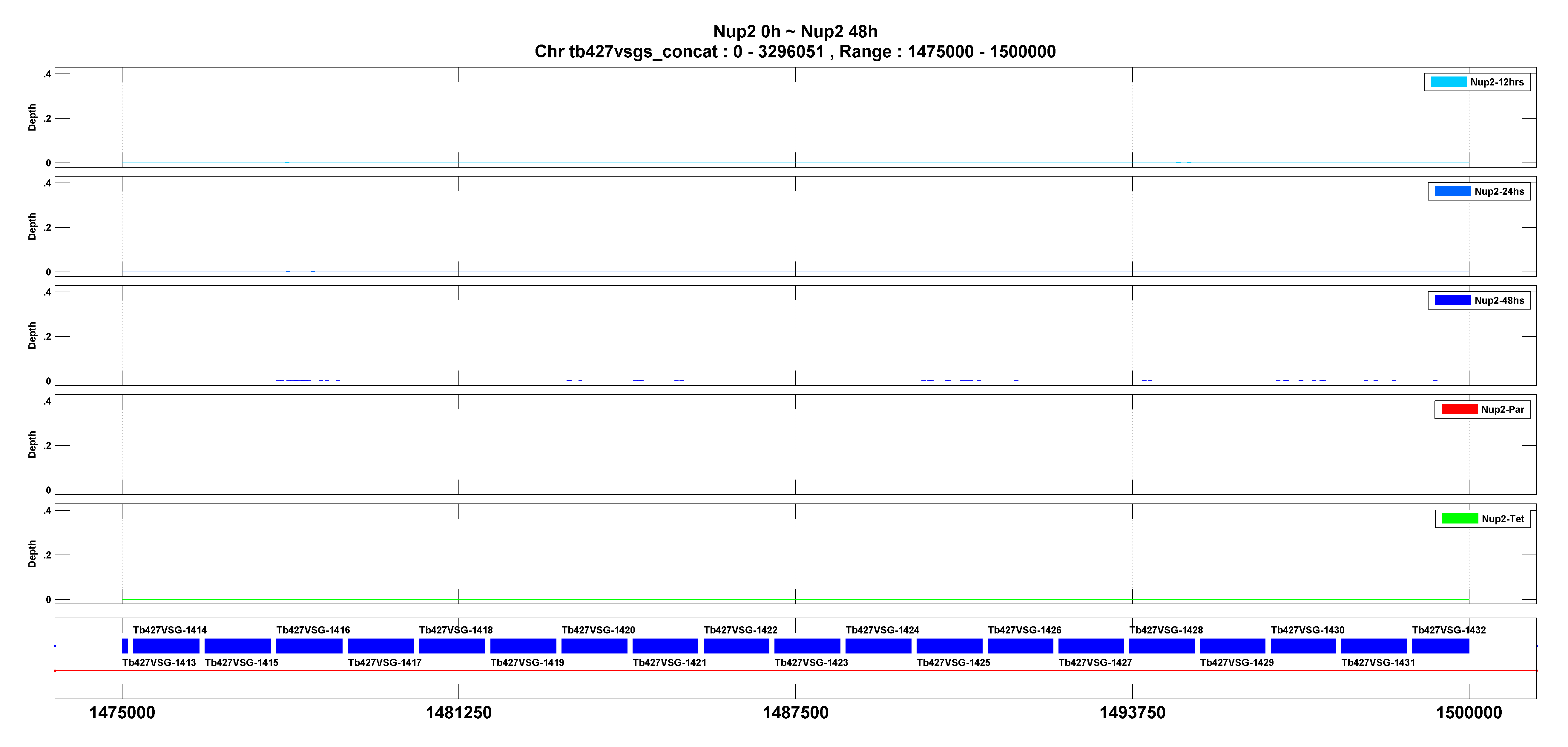

Supplement: SUPPLEMENTARY DATA [file supp_gkw751_nar-01100-x-2016-File026.zip › VSG transcriptome map/fig_tb427vsgs_concat_whole-seq_60.png]

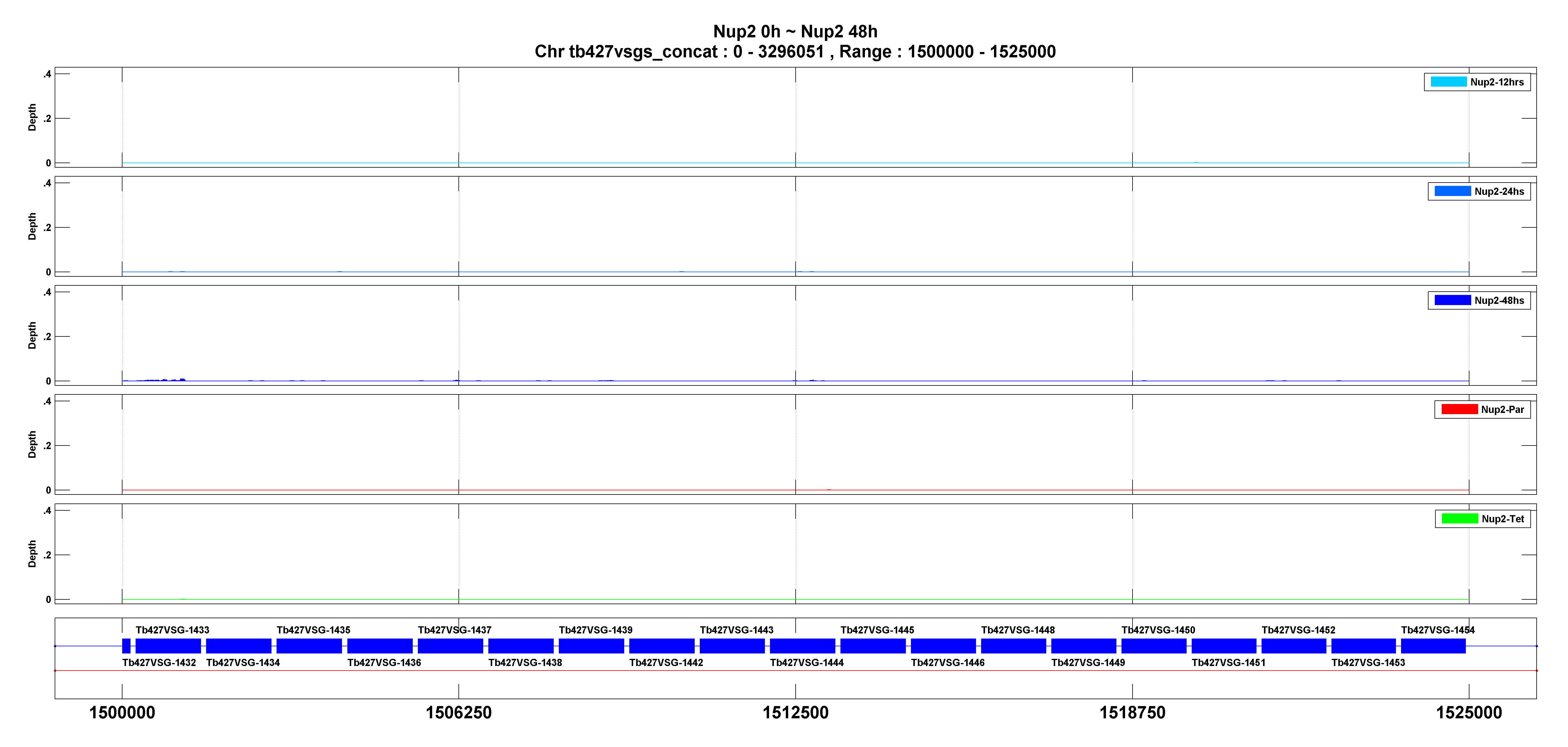

Supplement: SUPPLEMENTARY DATA [file supp_gkw751_nar-01100-x-2016-File026.zip › VSG transcriptome map/fig_tb427vsgs_concat_whole-seq_61.png]

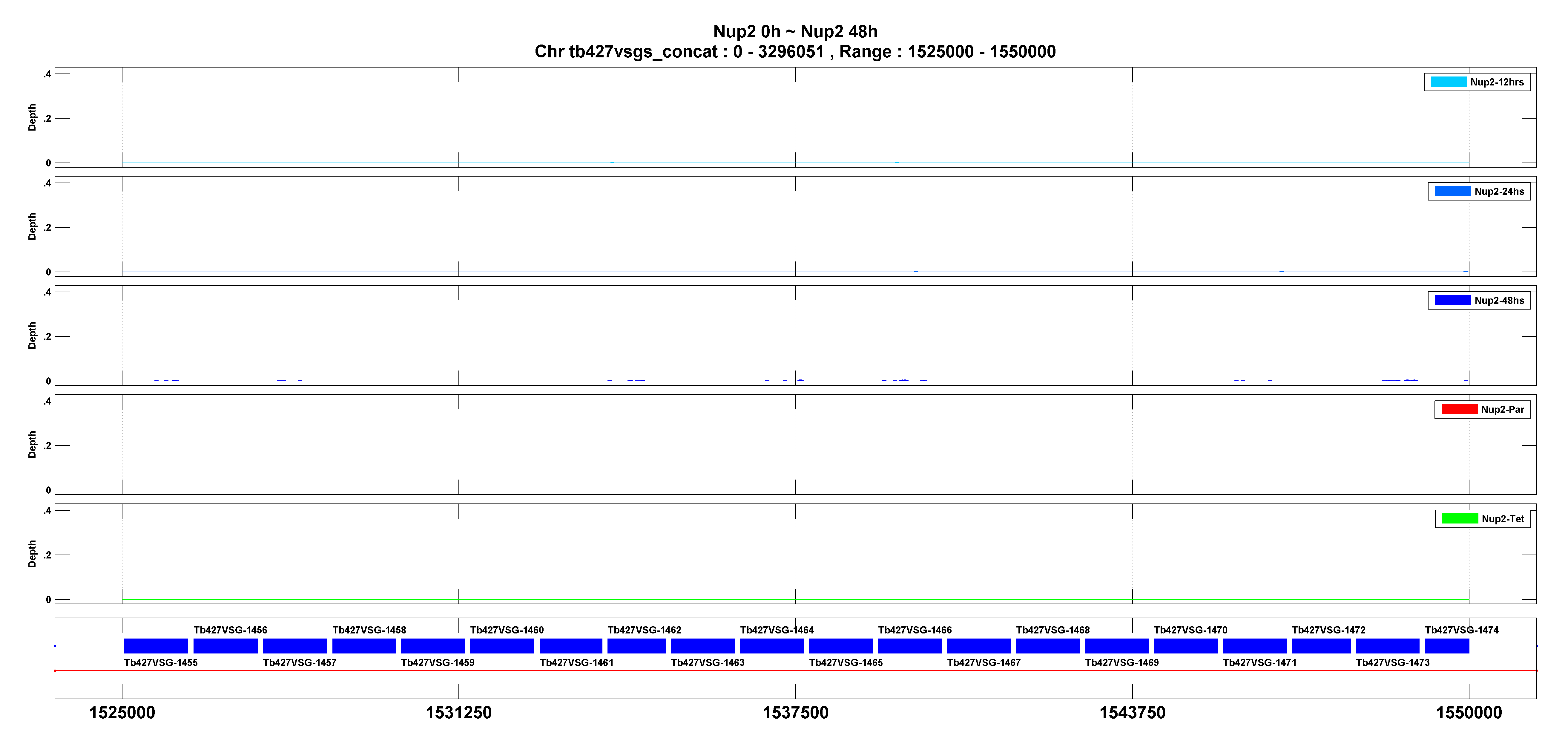

Supplement: SUPPLEMENTARY DATA [file supp_gkw751_nar-01100-x-2016-File026.zip › VSG transcriptome map/fig_tb427vsgs_concat_whole-seq_62.png]

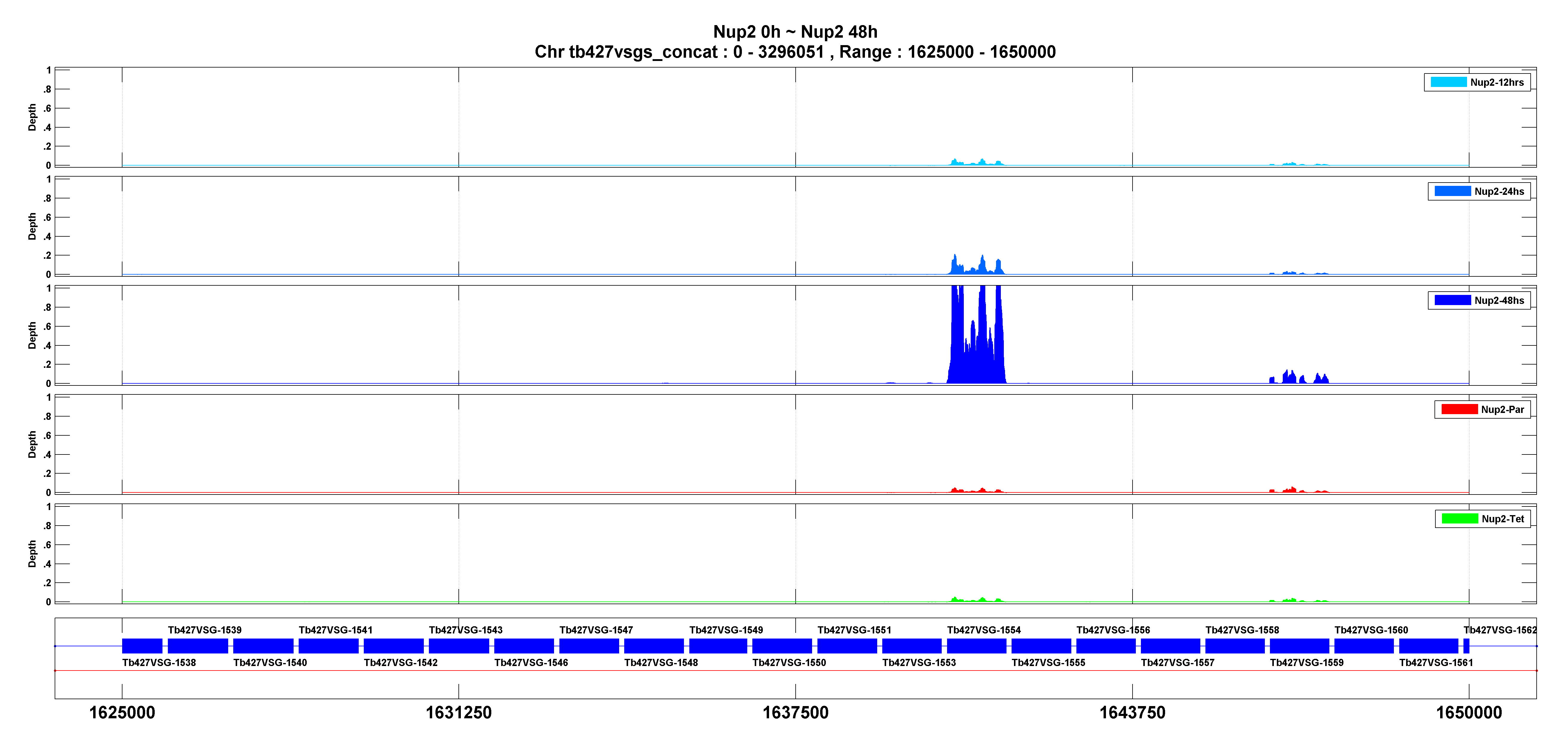

Supplement: SUPPLEMENTARY DATA [file supp_gkw751_nar-01100-x-2016-File026.zip › VSG transcriptome map/fig_tb427vsgs_concat_whole-seq_66.png]

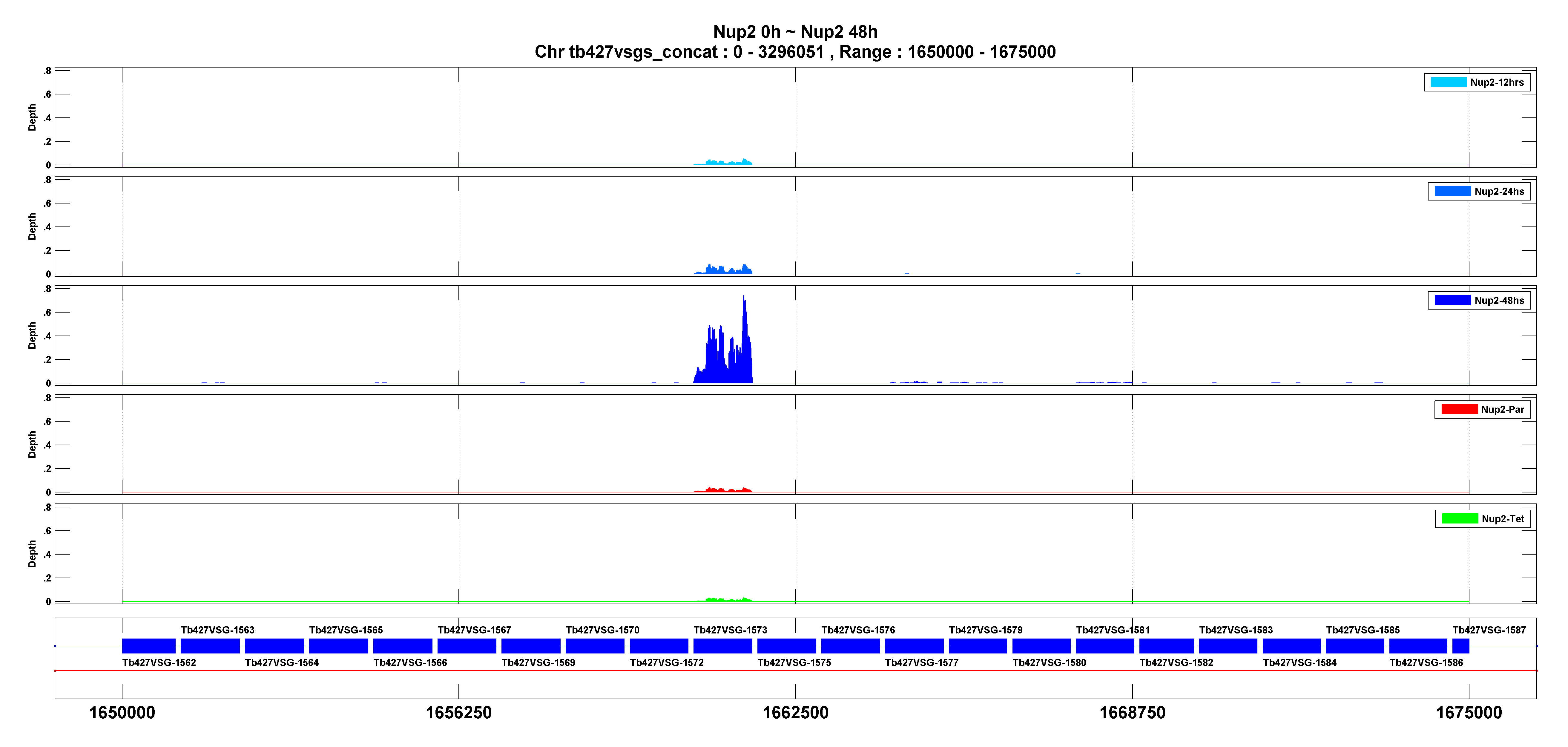

Supplement: SUPPLEMENTARY DATA [file supp_gkw751_nar-01100-x-2016-File026.zip › VSG transcriptome map/fig_tb427vsgs_concat_whole-seq_67.png]

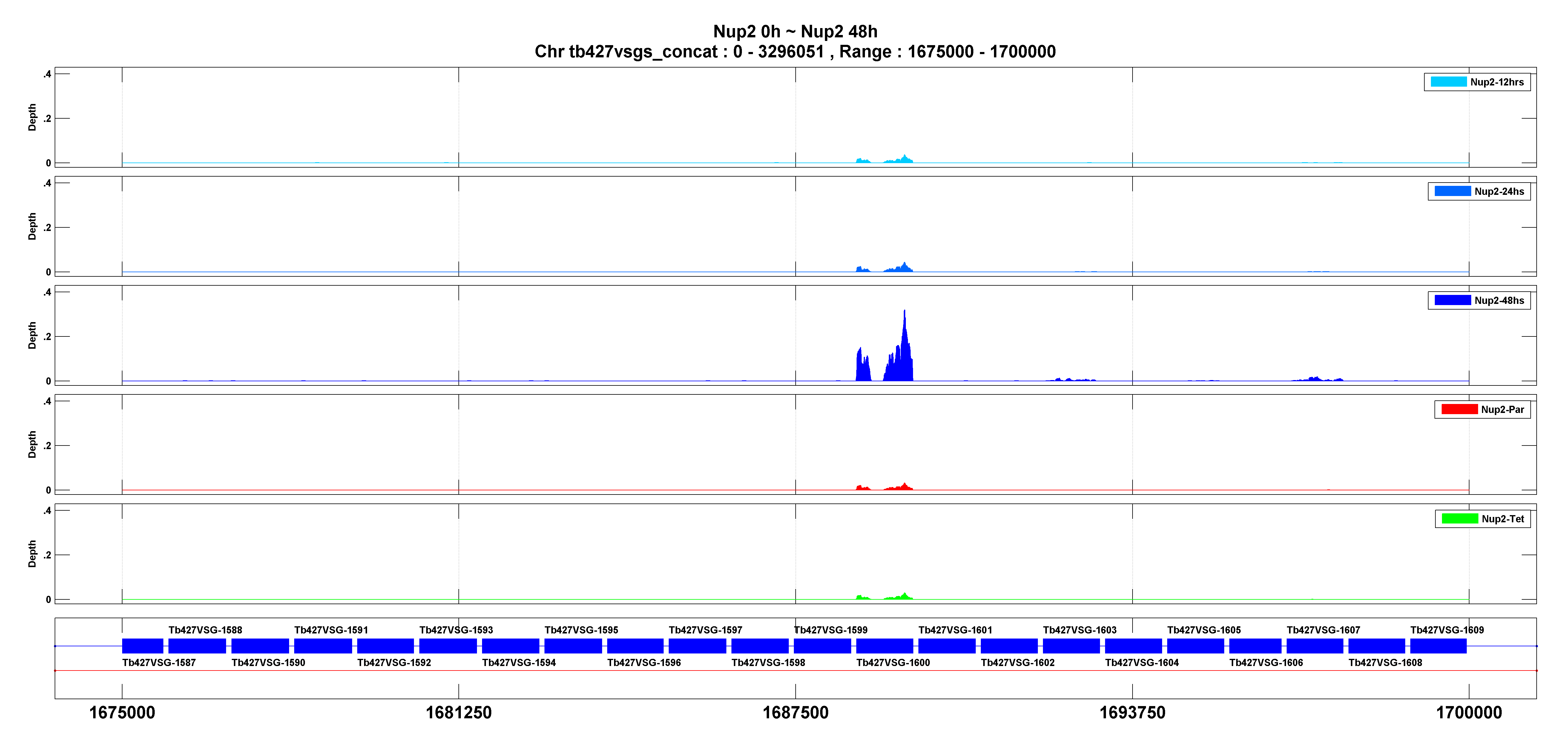

Supplement: SUPPLEMENTARY DATA [file supp_gkw751_nar-01100-x-2016-File026.zip › VSG transcriptome map/fig_tb427vsgs_concat_whole-seq_68.png]

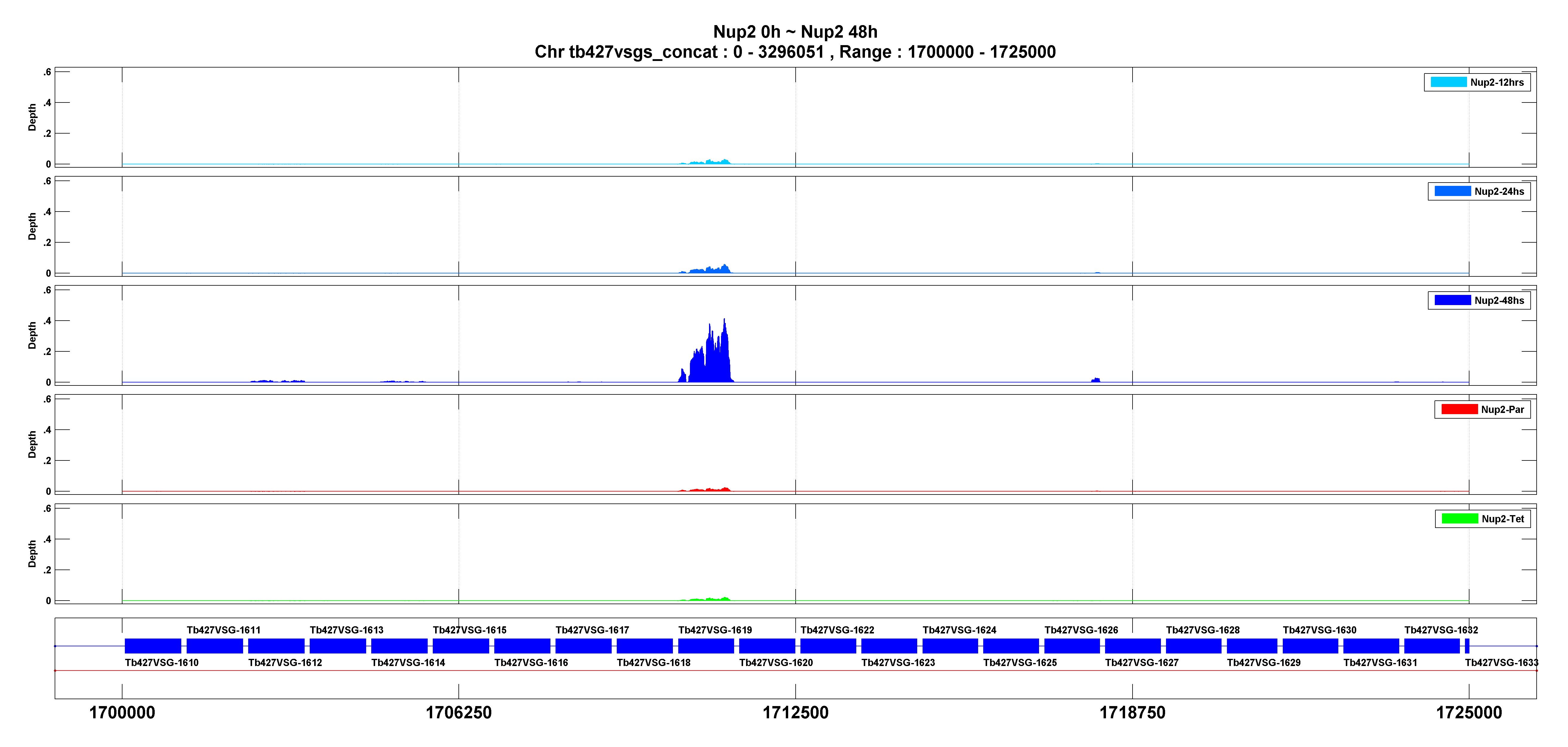

Supplement: SUPPLEMENTARY DATA [file supp_gkw751_nar-01100-x-2016-File026.zip › VSG transcriptome map/fig_tb427vsgs_concat_whole-seq_69.png]

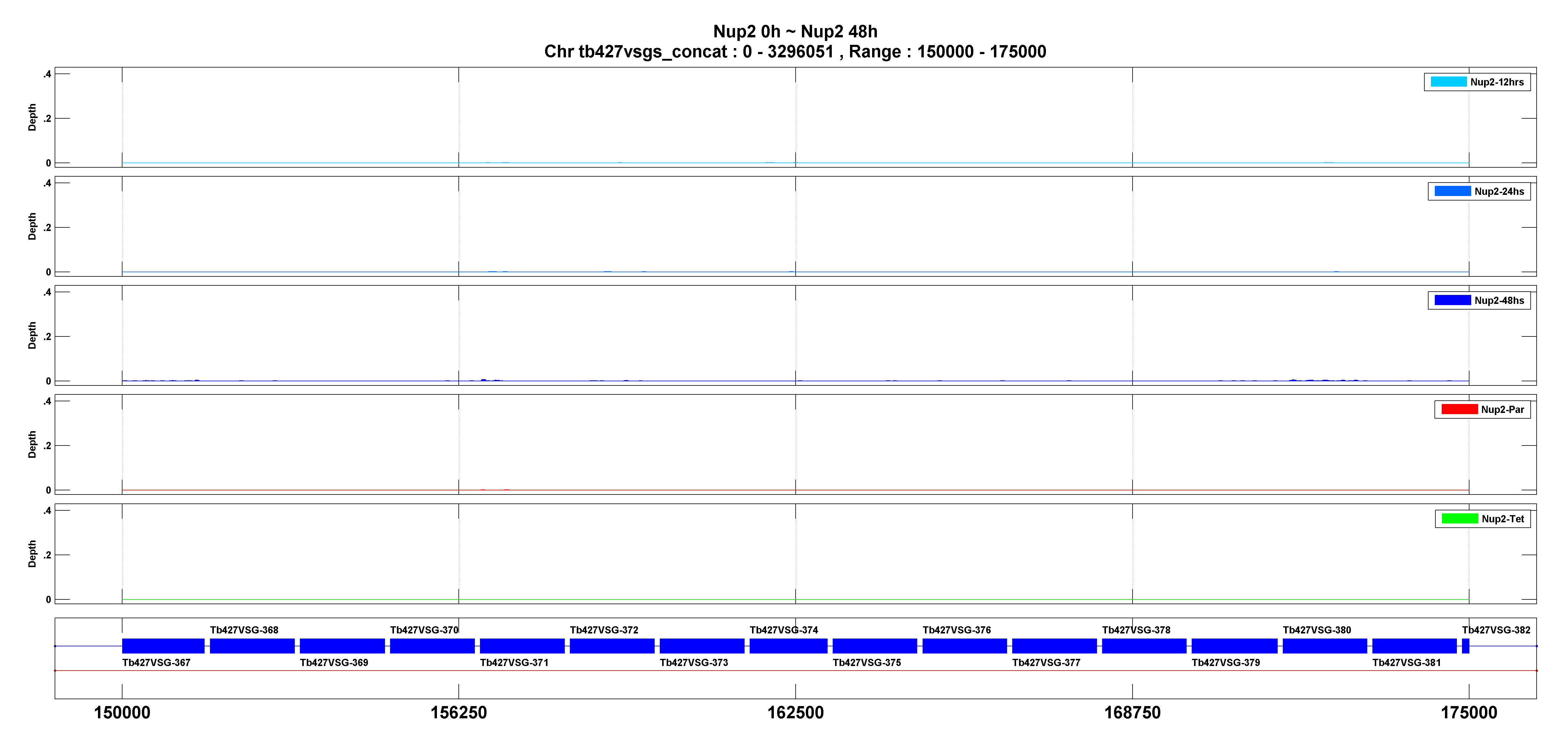

Supplement: SUPPLEMENTARY DATA [file supp_gkw751_nar-01100-x-2016-File026.zip › VSG transcriptome map/fig_tb427vsgs_concat_whole-seq_7.png]

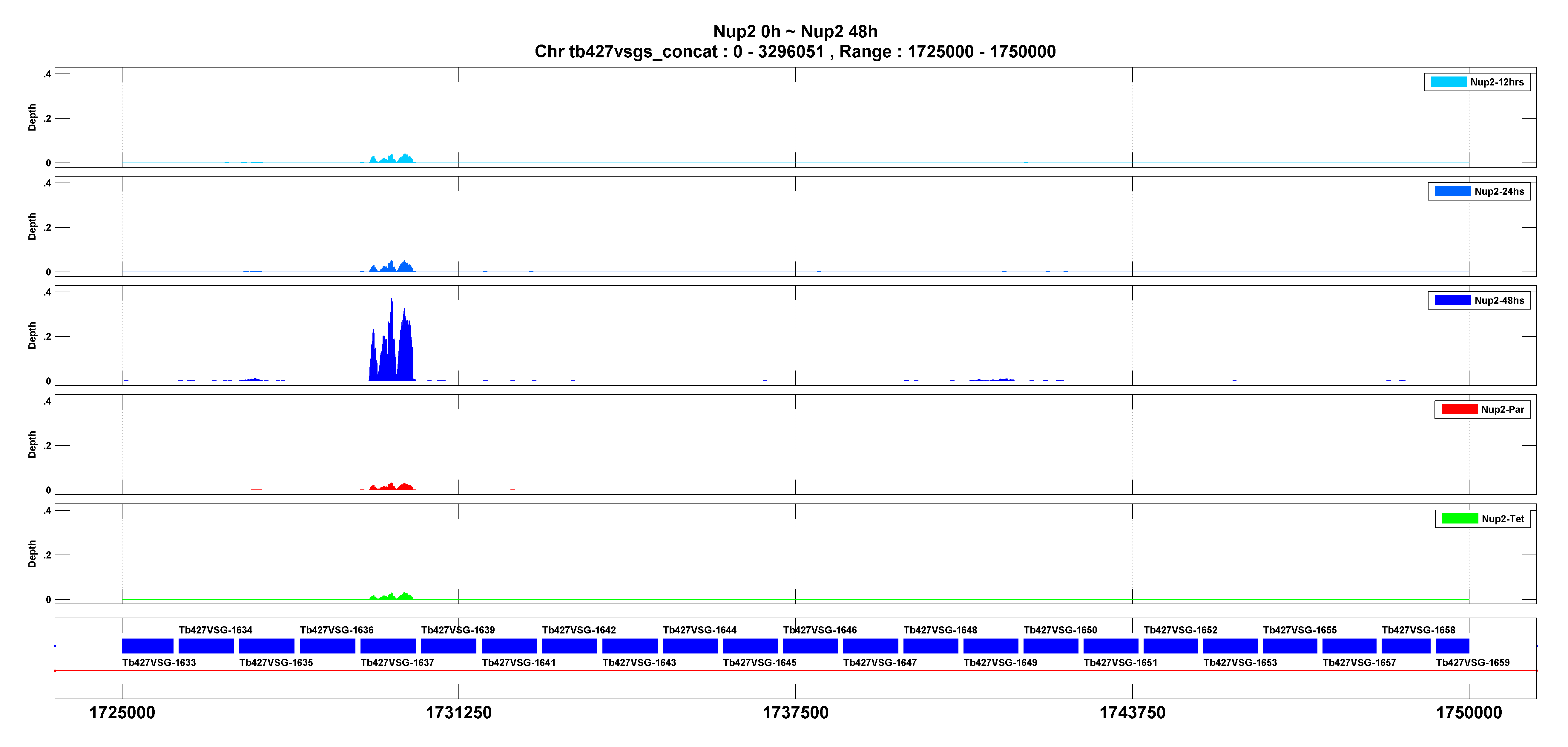

Supplement: SUPPLEMENTARY DATA [file supp_gkw751_nar-01100-x-2016-File026.zip › VSG transcriptome map/fig_tb427vsgs_concat_whole-seq_70.png]

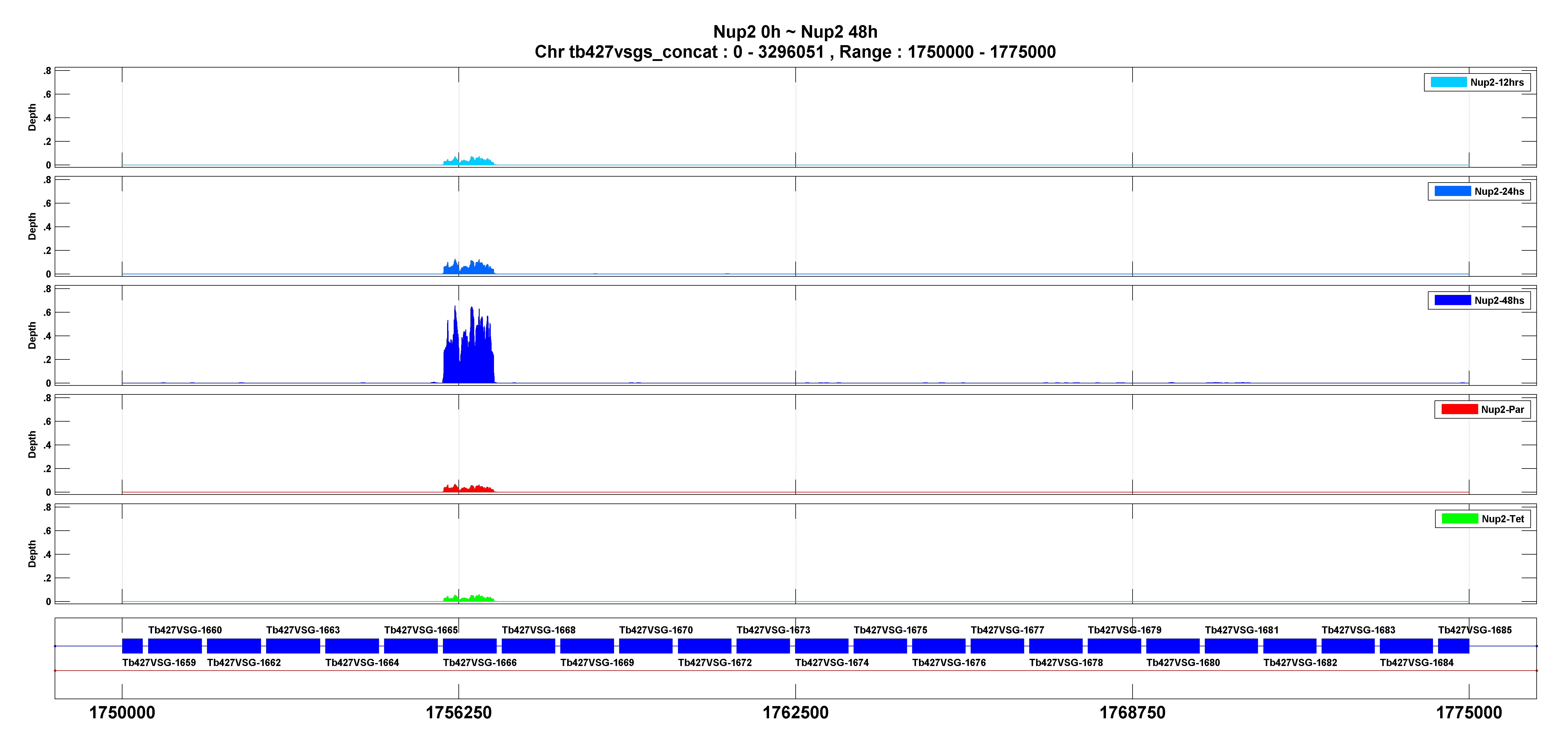

Supplement: SUPPLEMENTARY DATA [file supp_gkw751_nar-01100-x-2016-File026.zip › VSG transcriptome map/fig_tb427vsgs_concat_whole-seq_71.png]

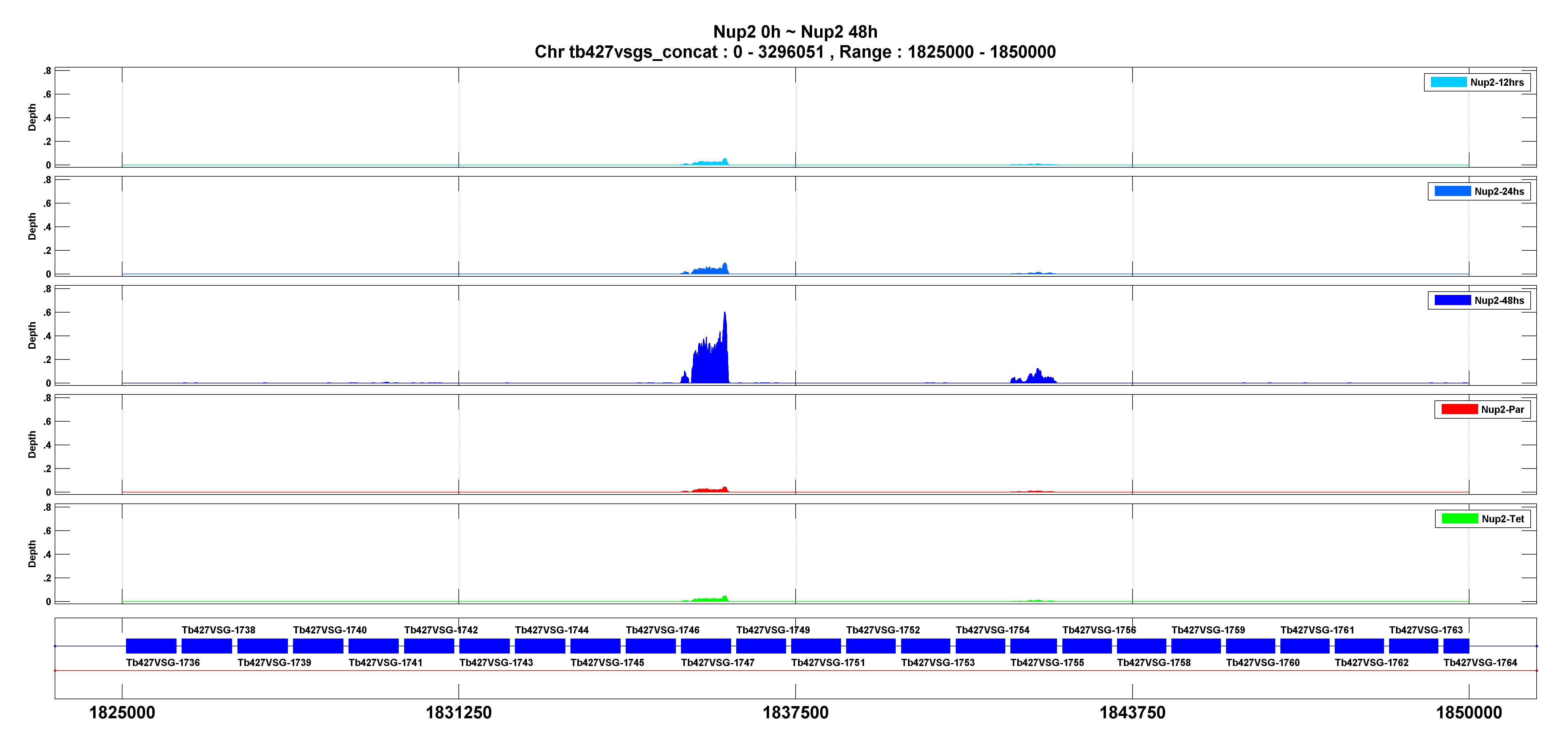

Supplement: SUPPLEMENTARY DATA [file supp_gkw751_nar-01100-x-2016-File026.zip › VSG transcriptome map/fig_tb427vsgs_concat_whole-seq_74.png]

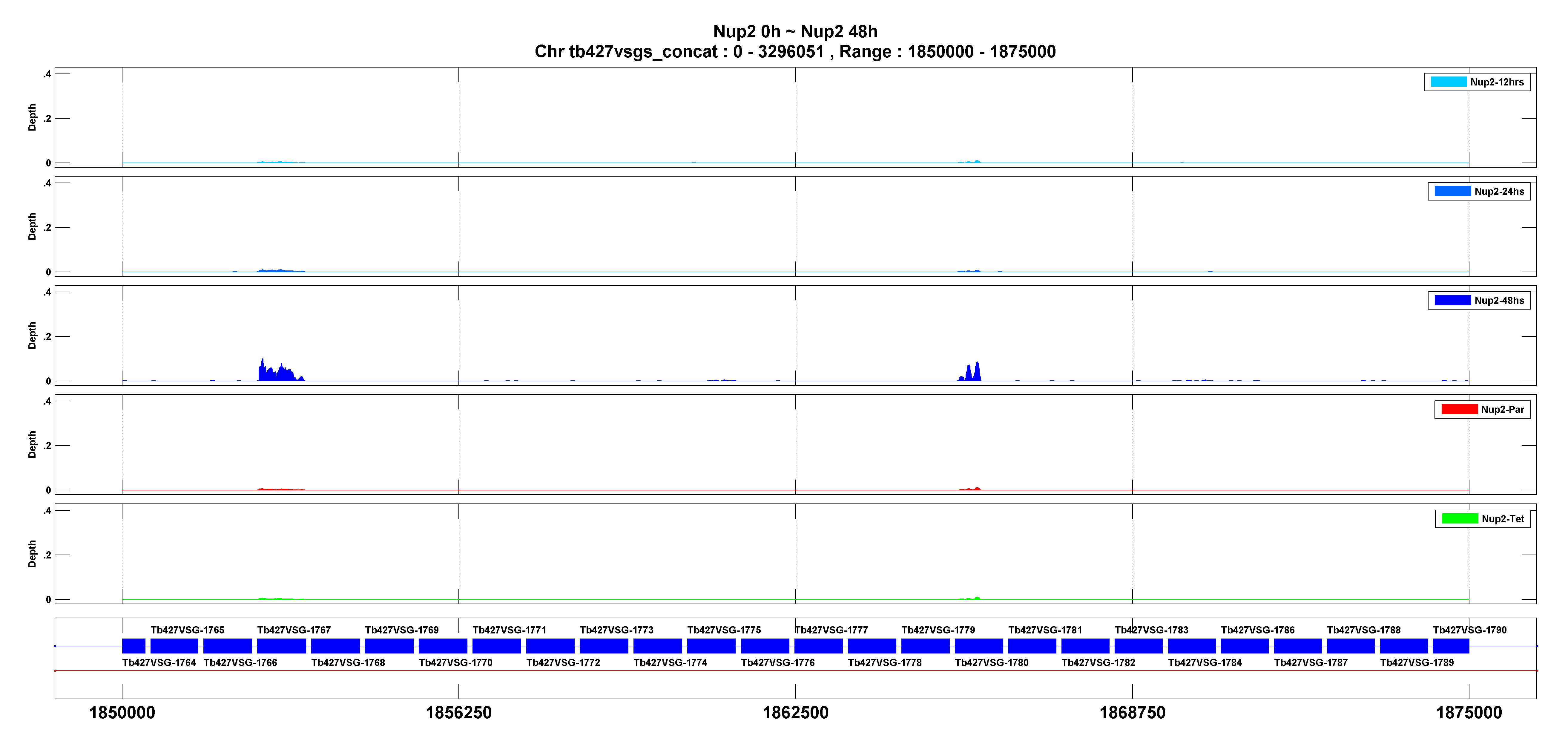

Supplement: SUPPLEMENTARY DATA [file supp_gkw751_nar-01100-x-2016-File026.zip › VSG transcriptome map/fig_tb427vsgs_concat_whole-seq_75.png]

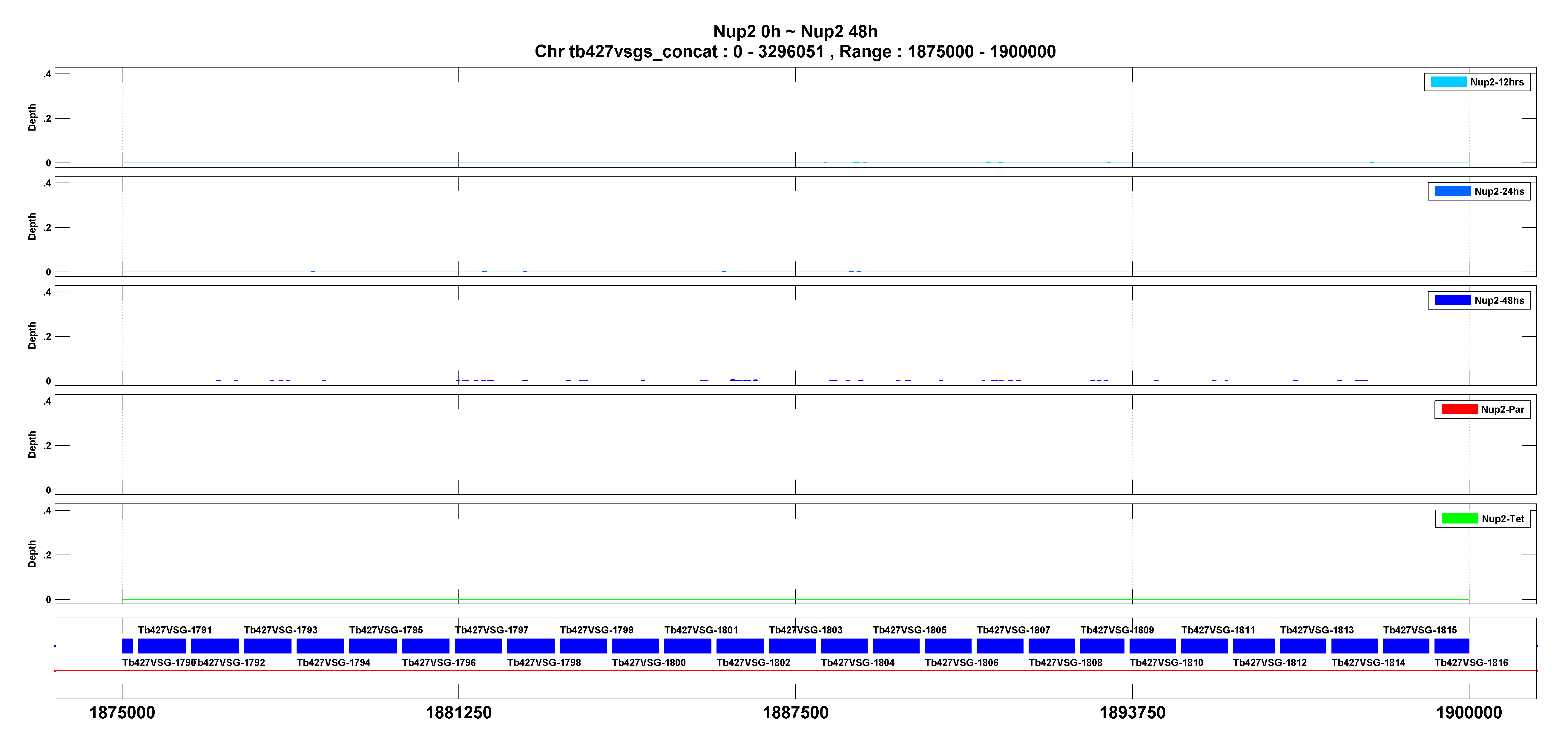

Supplement: SUPPLEMENTARY DATA [file supp_gkw751_nar-01100-x-2016-File026.zip › VSG transcriptome map/fig_tb427vsgs_concat_whole-seq_76.png]

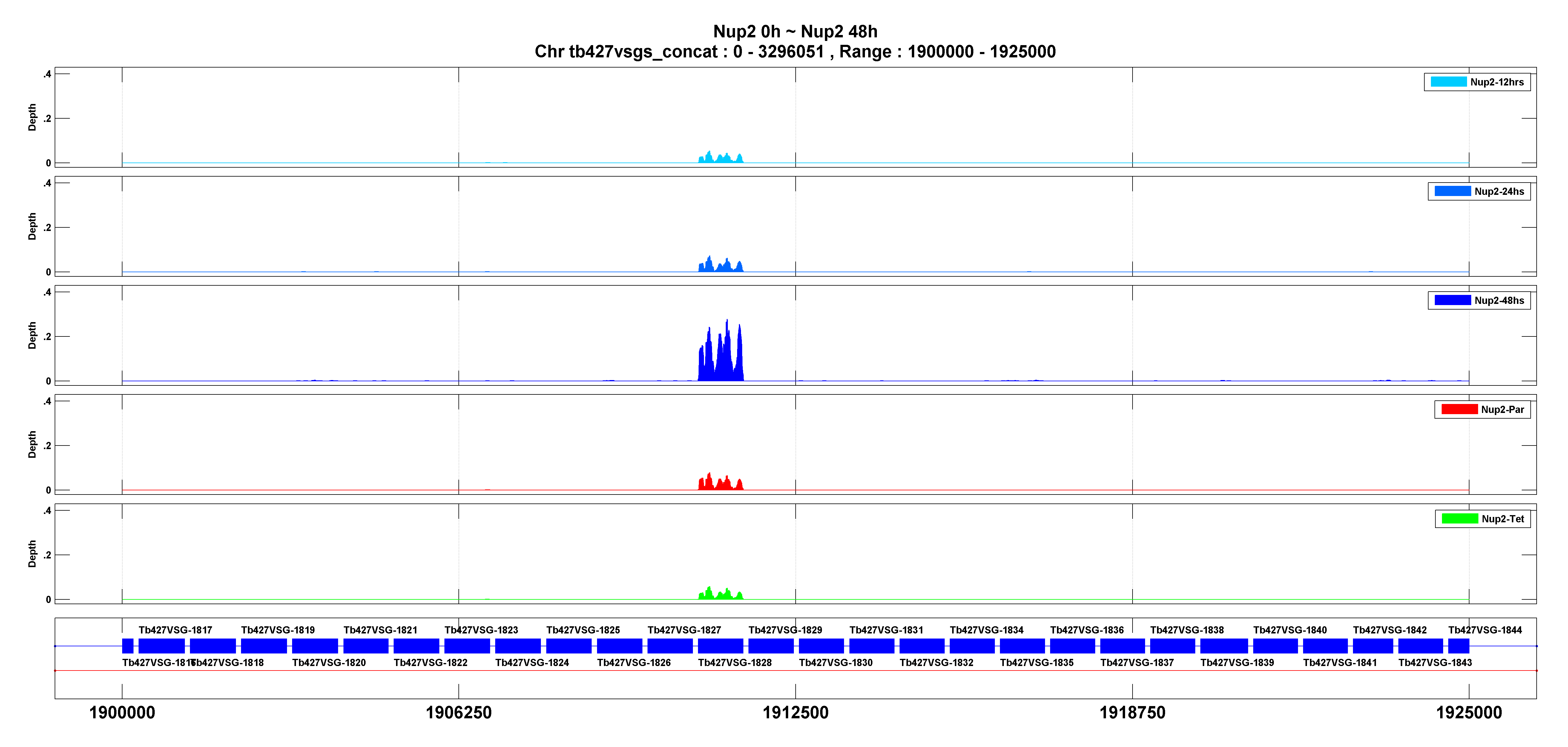

Supplement: SUPPLEMENTARY DATA [file supp_gkw751_nar-01100-x-2016-File026.zip › VSG transcriptome map/fig_tb427vsgs_concat_whole-seq_77.png]

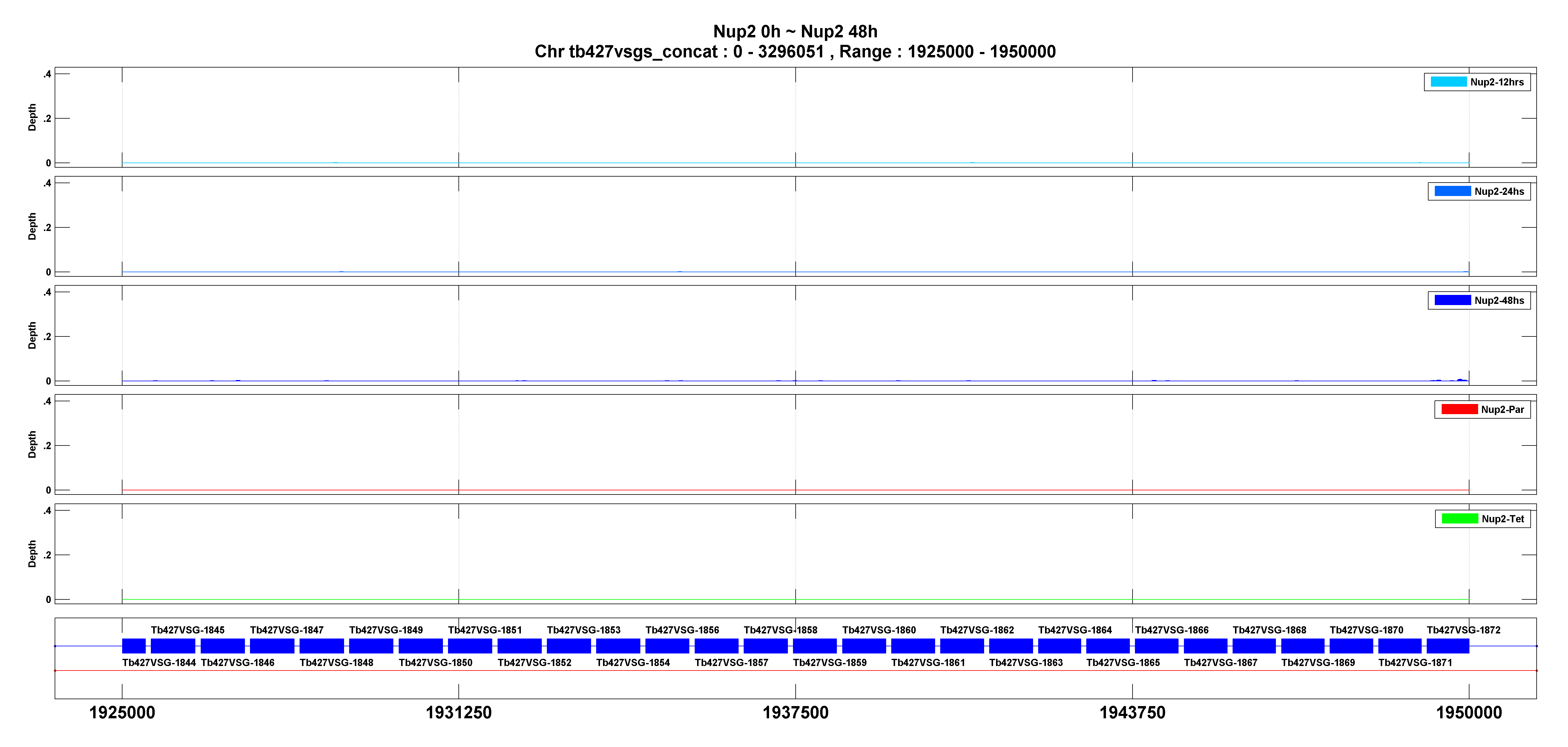

Supplement: SUPPLEMENTARY DATA [file supp_gkw751_nar-01100-x-2016-File026.zip › VSG transcriptome map/fig_tb427vsgs_concat_whole-seq_78.png]

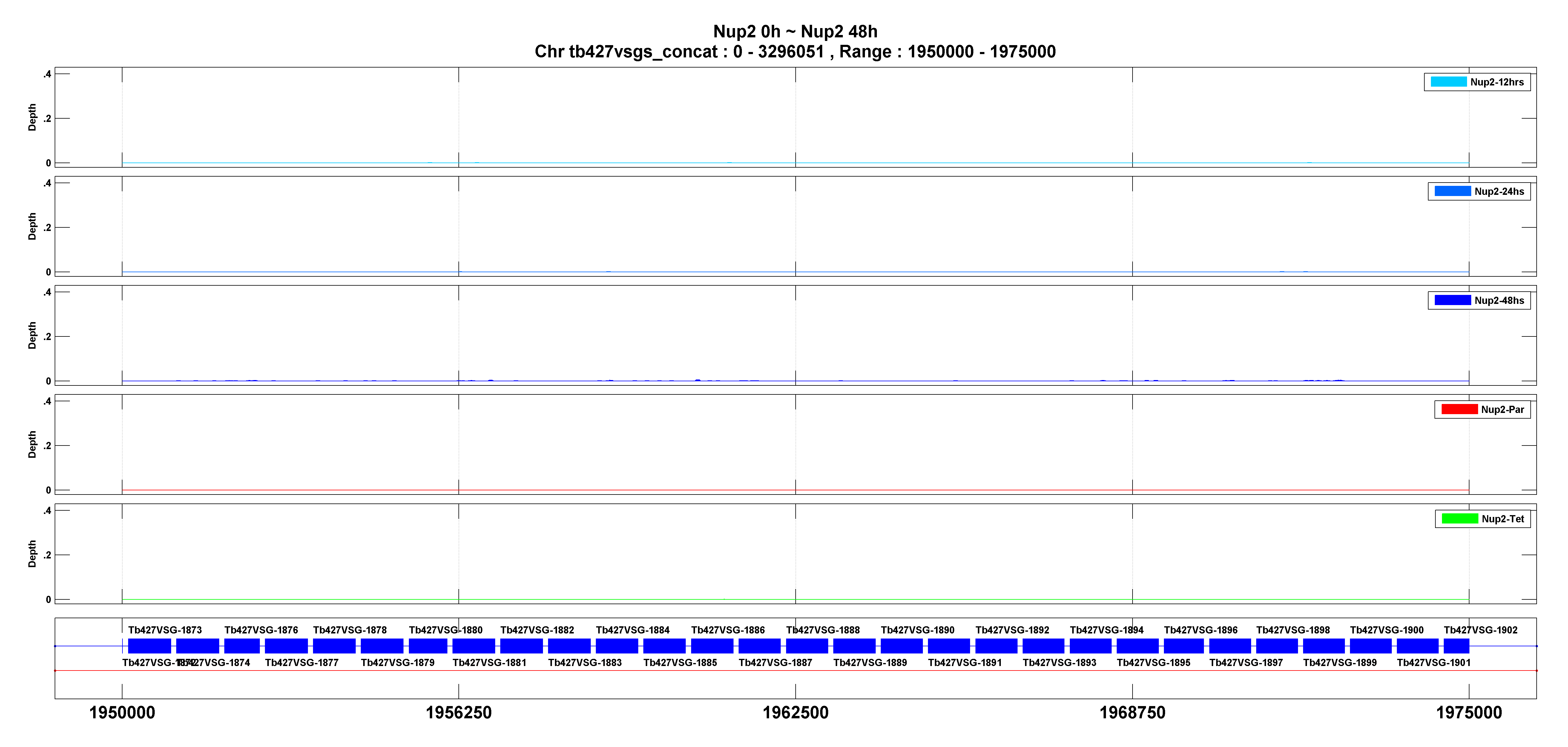

Supplement: SUPPLEMENTARY DATA [file supp_gkw751_nar-01100-x-2016-File026.zip › VSG transcriptome map/fig_tb427vsgs_concat_whole-seq_79.png]

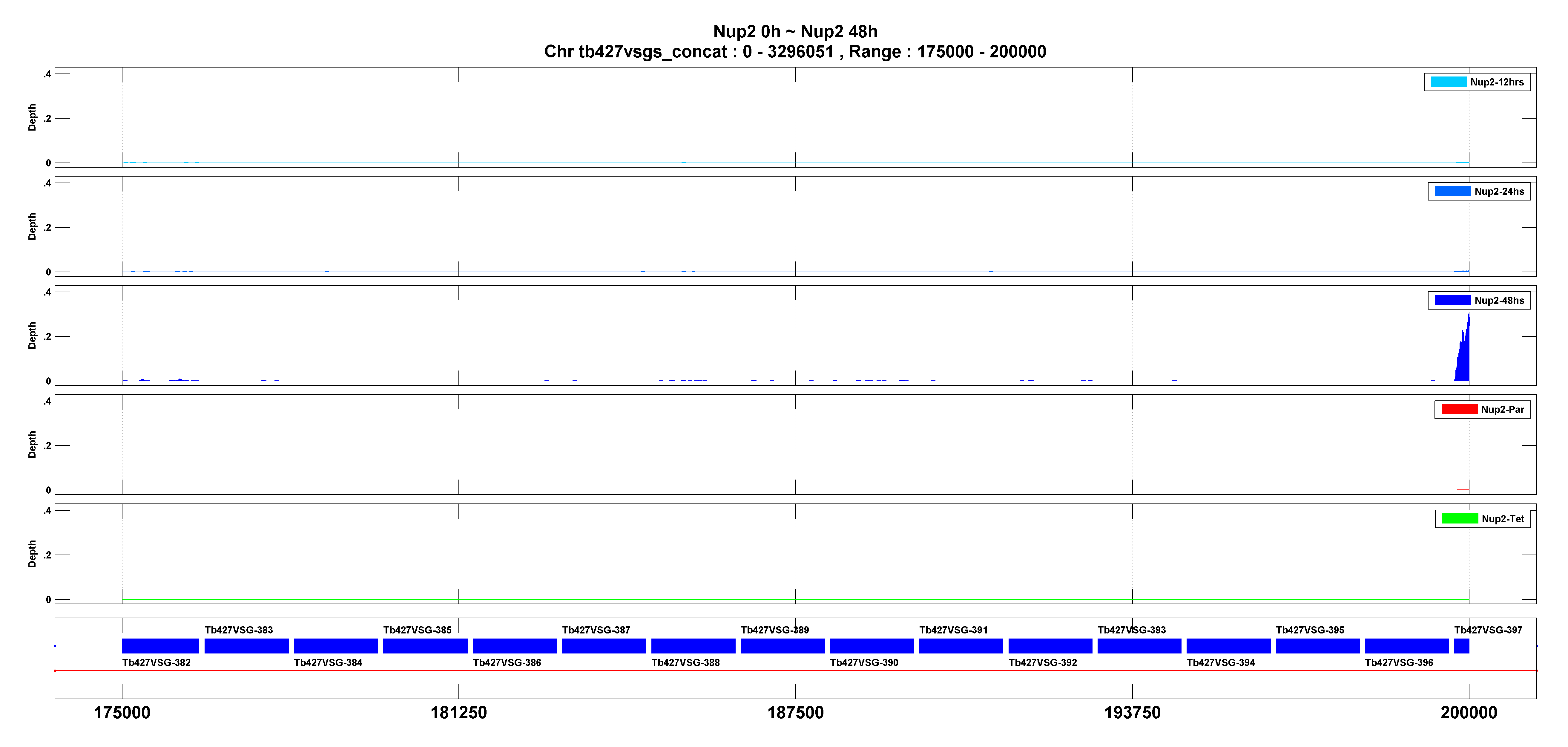

Supplement: SUPPLEMENTARY DATA [file supp_gkw751_nar-01100-x-2016-File026.zip › VSG transcriptome map/fig_tb427vsgs_concat_whole-seq_8.png]

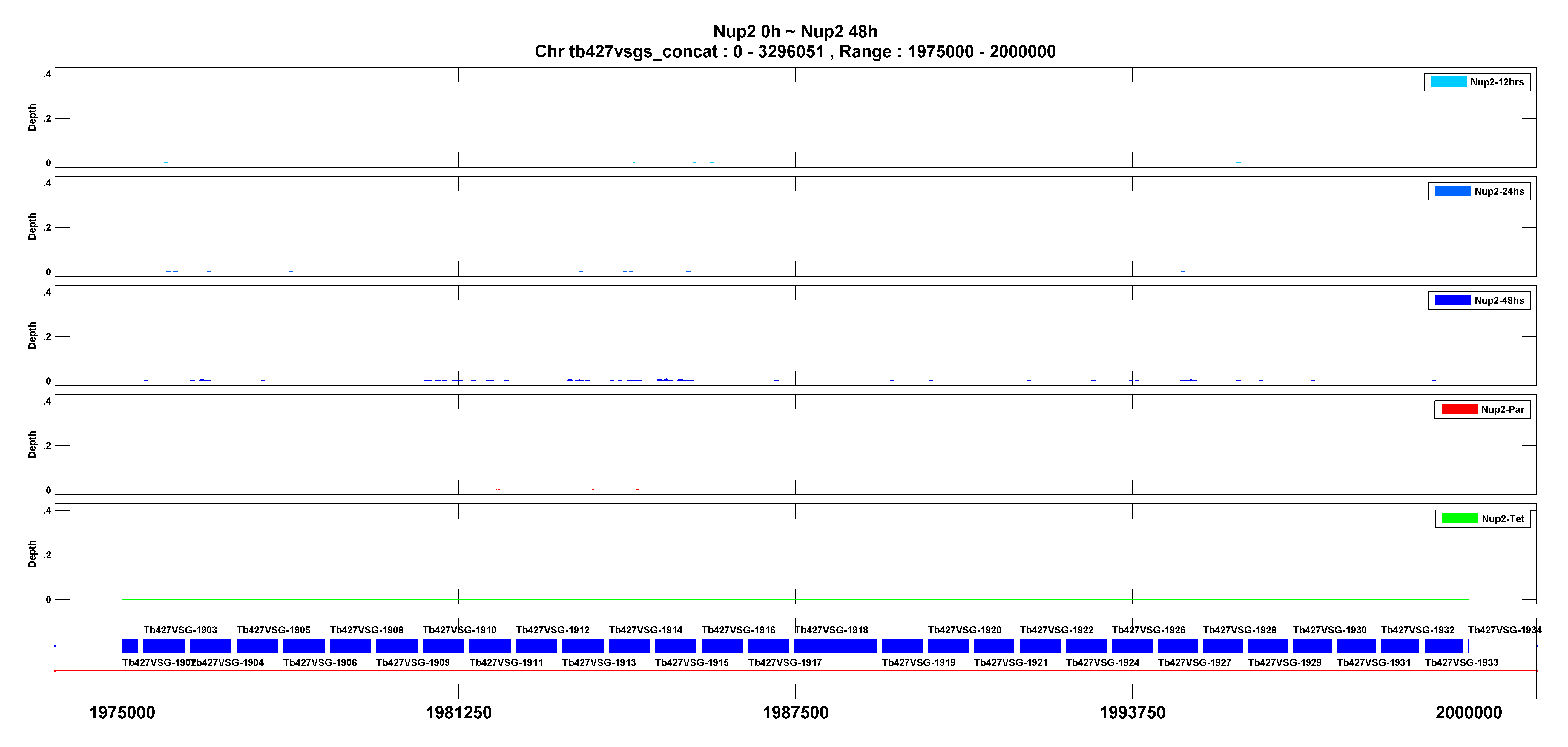

Supplement: SUPPLEMENTARY DATA [file supp_gkw751_nar-01100-x-2016-File026.zip › VSG transcriptome map/fig_tb427vsgs_concat_whole-seq_80.png]

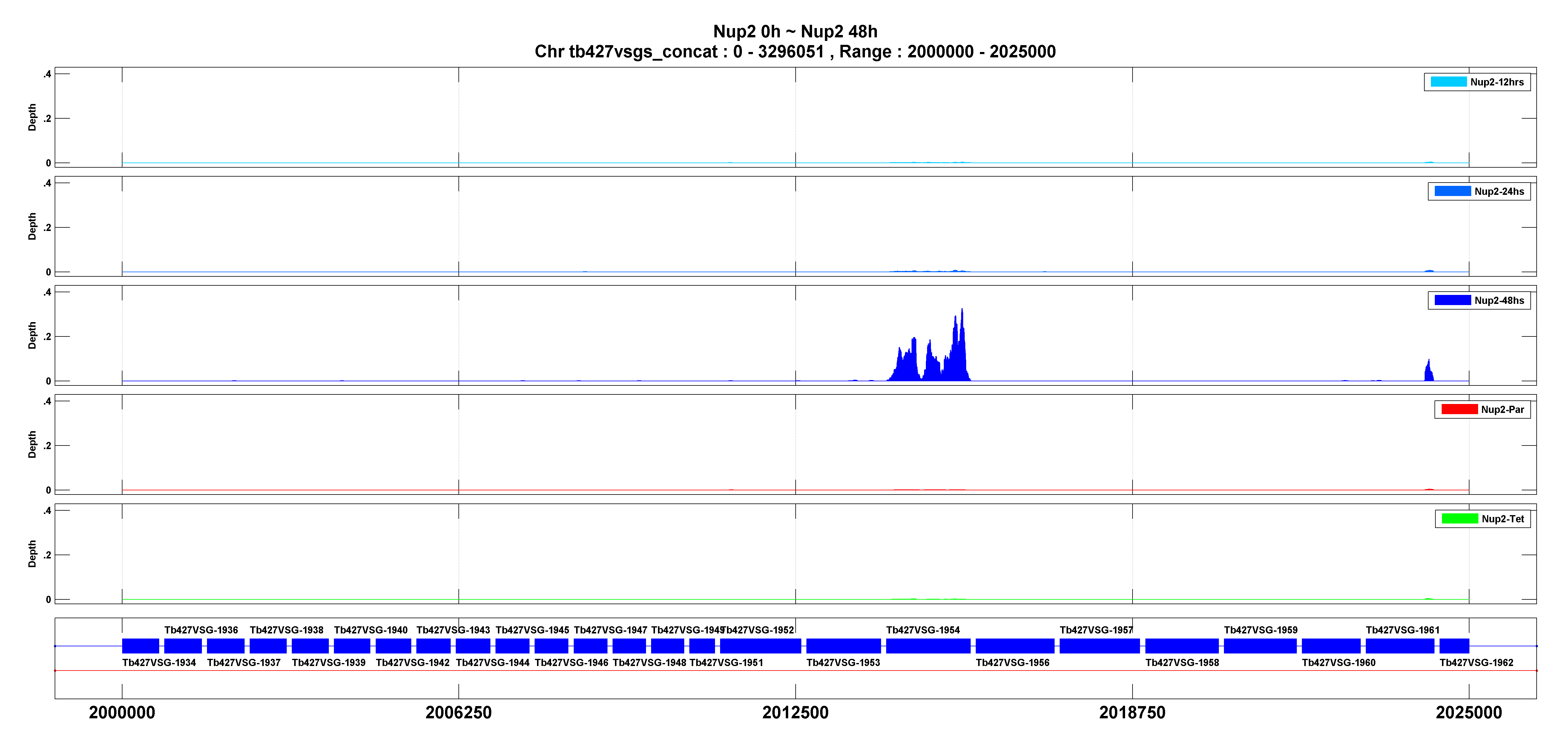

Supplement: SUPPLEMENTARY DATA [file supp_gkw751_nar-01100-x-2016-File026.zip › VSG transcriptome map/fig_tb427vsgs_concat_whole-seq_81.png]

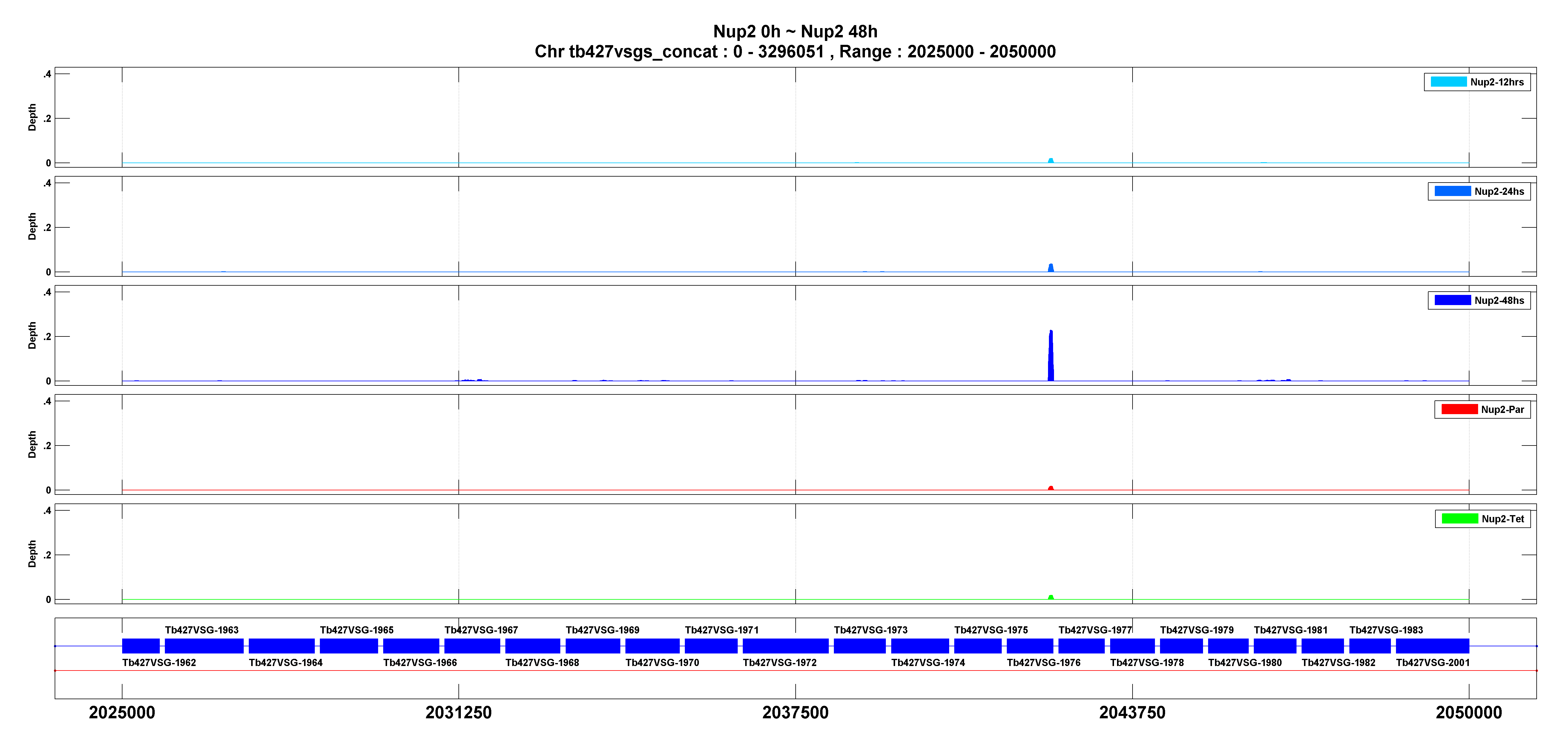

Supplement: SUPPLEMENTARY DATA [file supp_gkw751_nar-01100-x-2016-File026.zip › VSG transcriptome map/fig_tb427vsgs_concat_whole-seq_82.png]

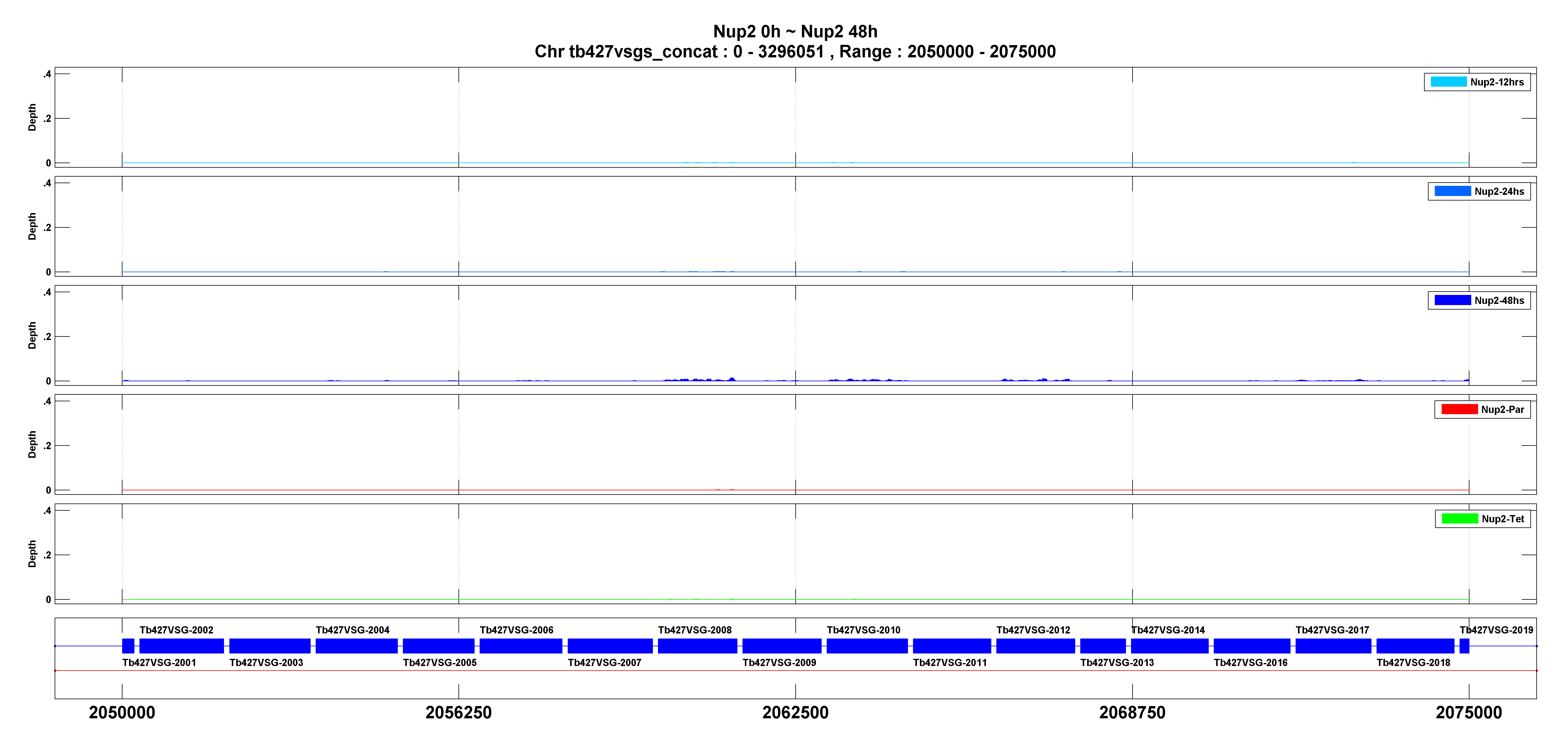

Supplement: SUPPLEMENTARY DATA [file supp_gkw751_nar-01100-x-2016-File026.zip › VSG transcriptome map/fig_tb427vsgs_concat_whole-seq_83.png]

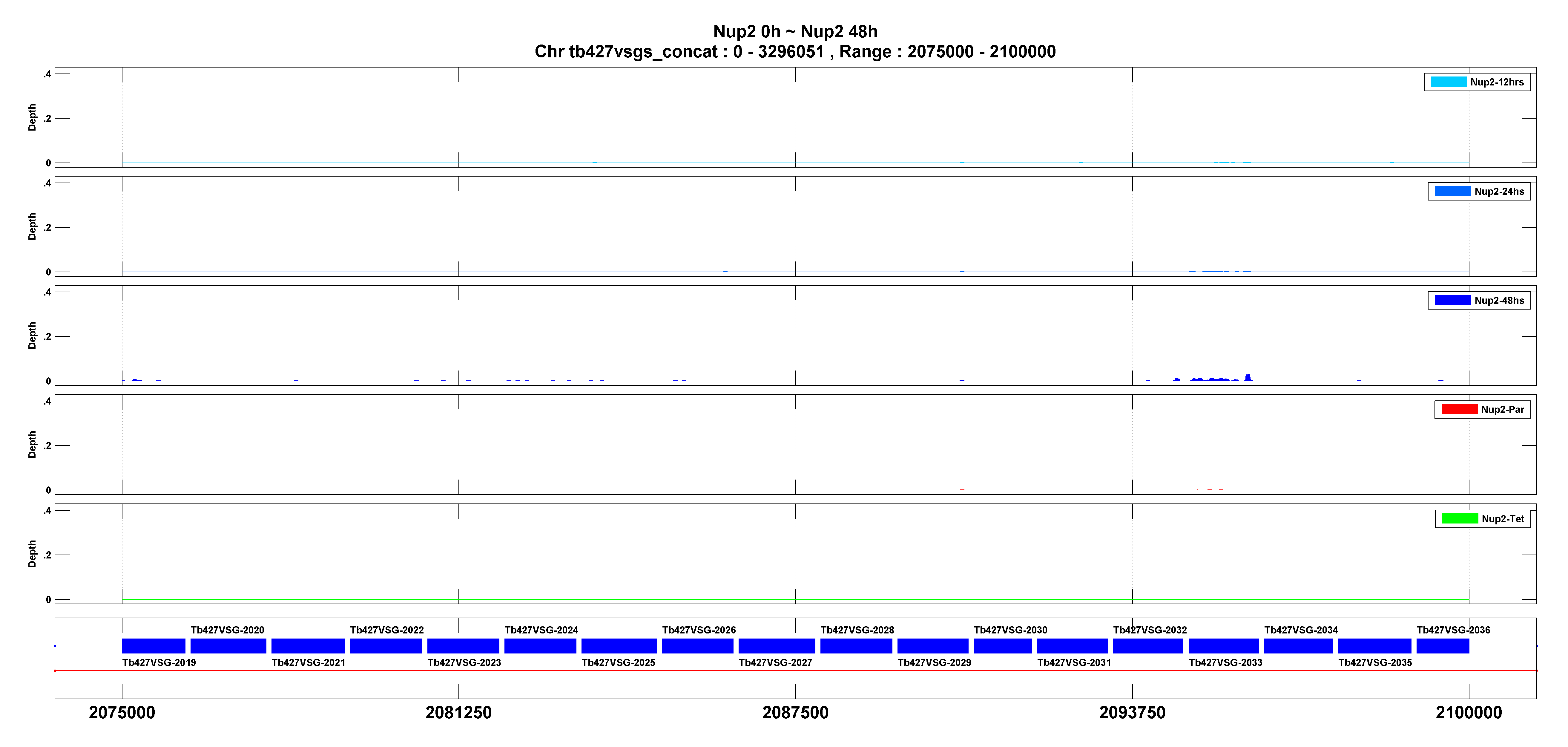

Supplement: SUPPLEMENTARY DATA [file supp_gkw751_nar-01100-x-2016-File026.zip › VSG transcriptome map/fig_tb427vsgs_concat_whole-seq_84.png]

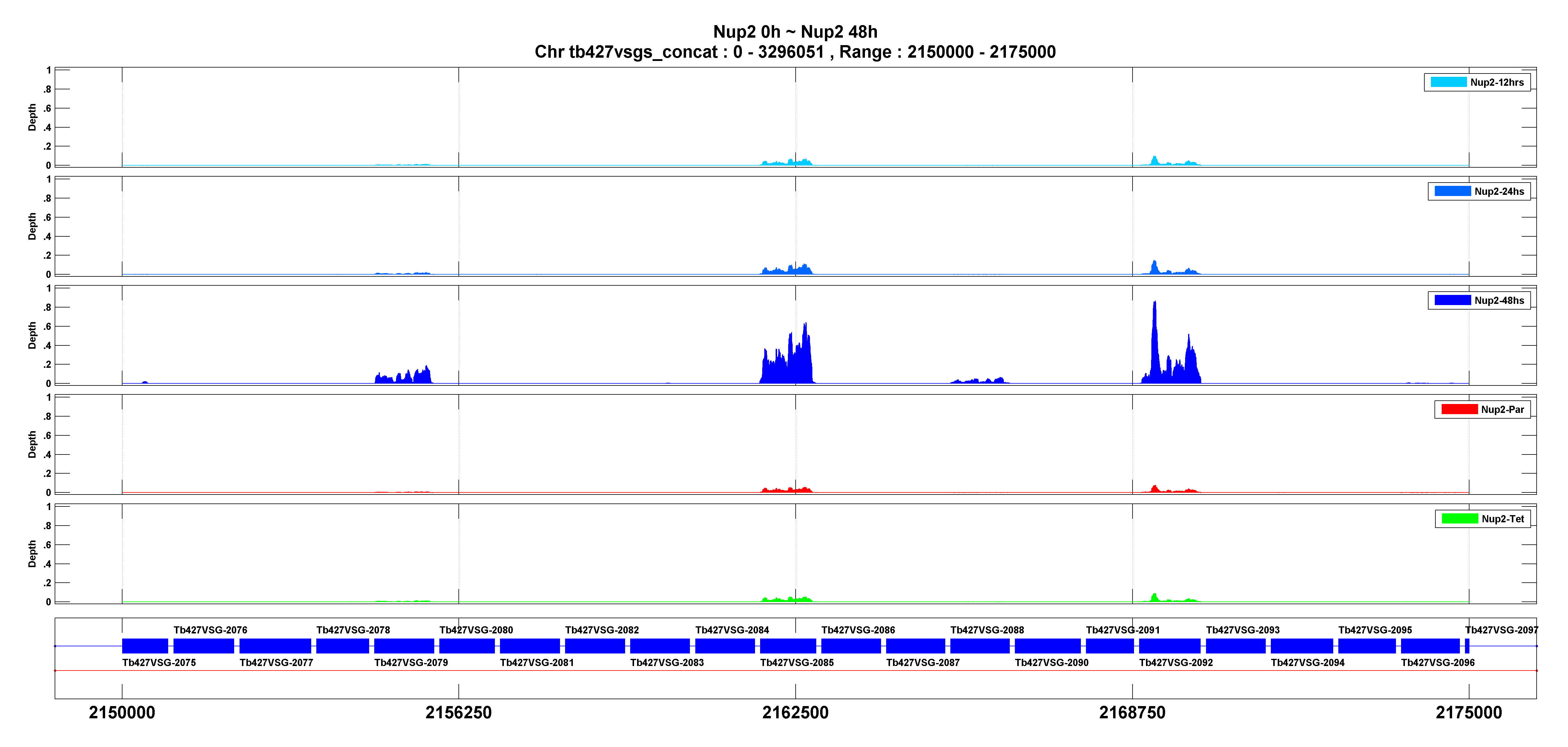

Supplement: SUPPLEMENTARY DATA [file supp_gkw751_nar-01100-x-2016-File026.zip › VSG transcriptome map/fig_tb427vsgs_concat_whole-seq_87.png]

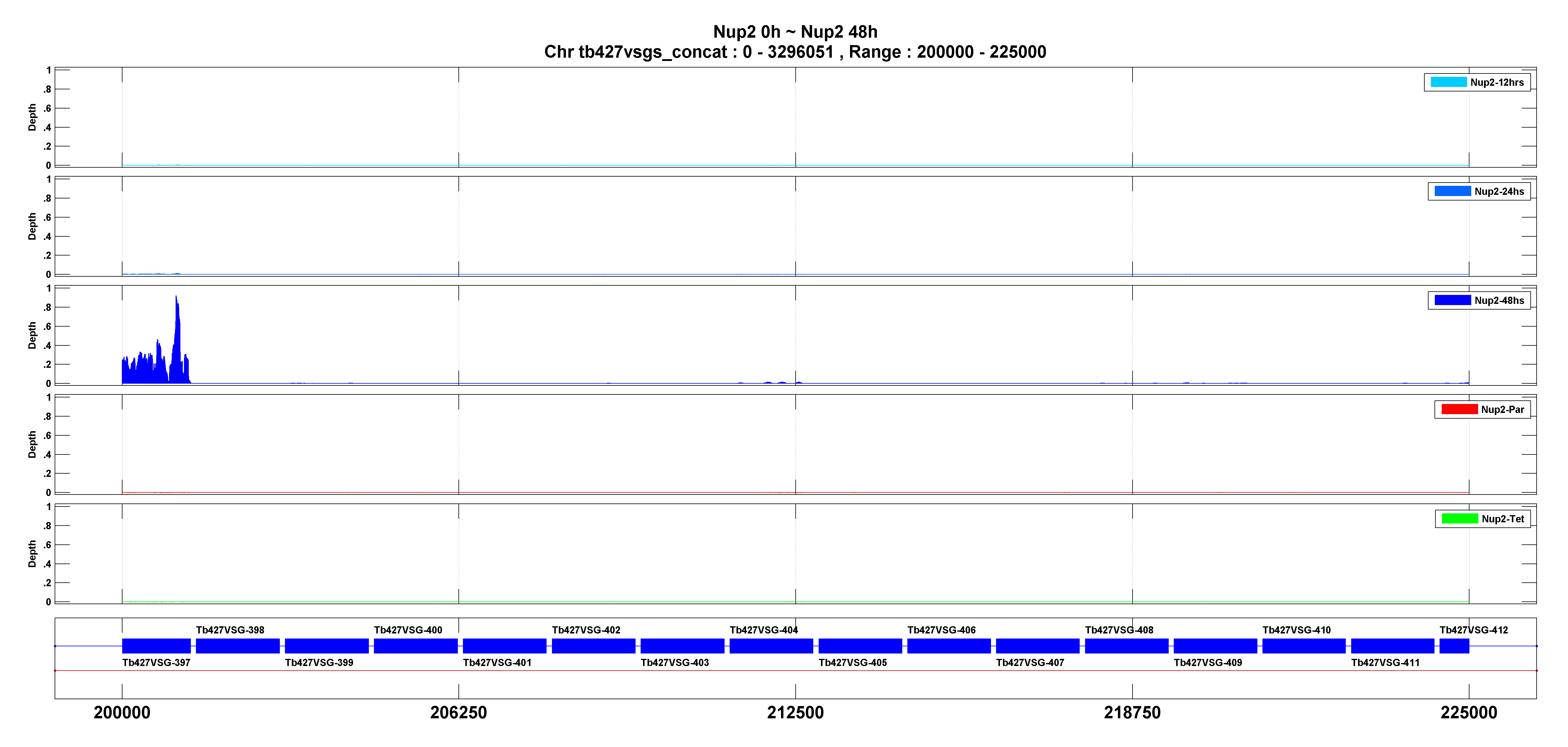

Supplement: SUPPLEMENTARY DATA [file supp_gkw751_nar-01100-x-2016-File026.zip › VSG transcriptome map/fig_tb427vsgs_concat_whole-seq_9.png]

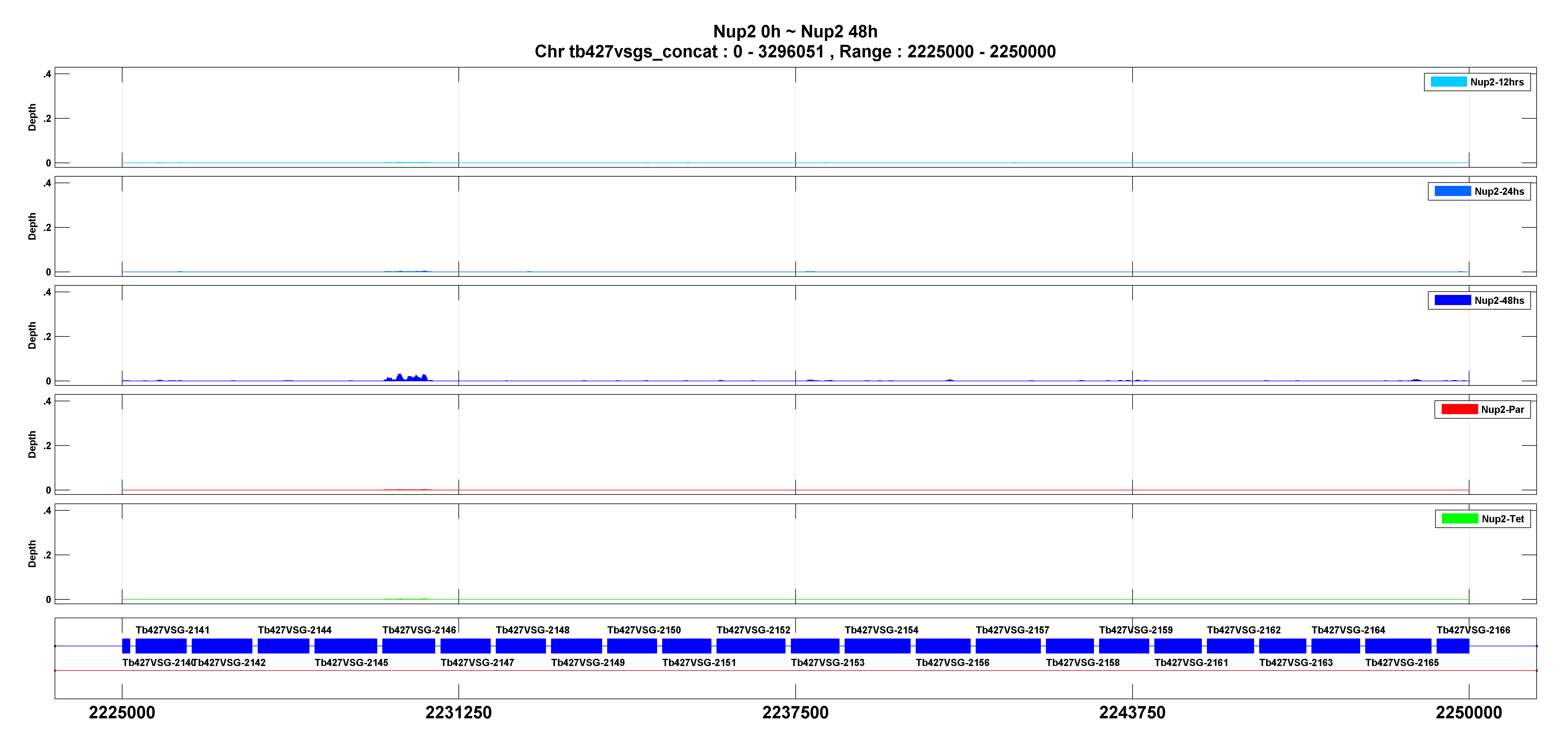

Supplement: SUPPLEMENTARY DATA [file supp_gkw751_nar-01100-x-2016-File026.zip › VSG transcriptome map/fig_tb427vsgs_concat_whole-seq_90.png]

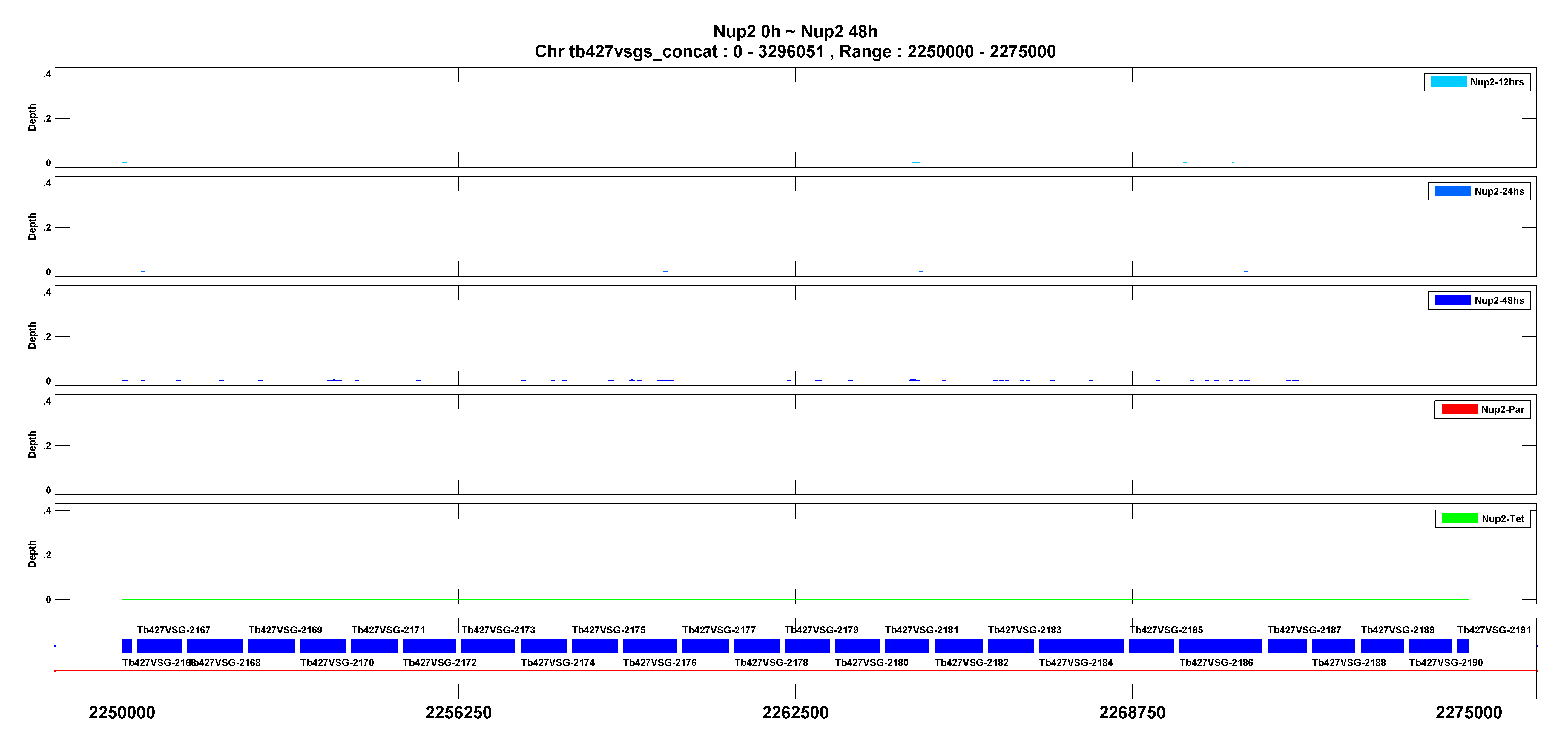

Supplement: SUPPLEMENTARY DATA [file supp_gkw751_nar-01100-x-2016-File026.zip › VSG transcriptome map/fig_tb427vsgs_concat_whole-seq_91.png]

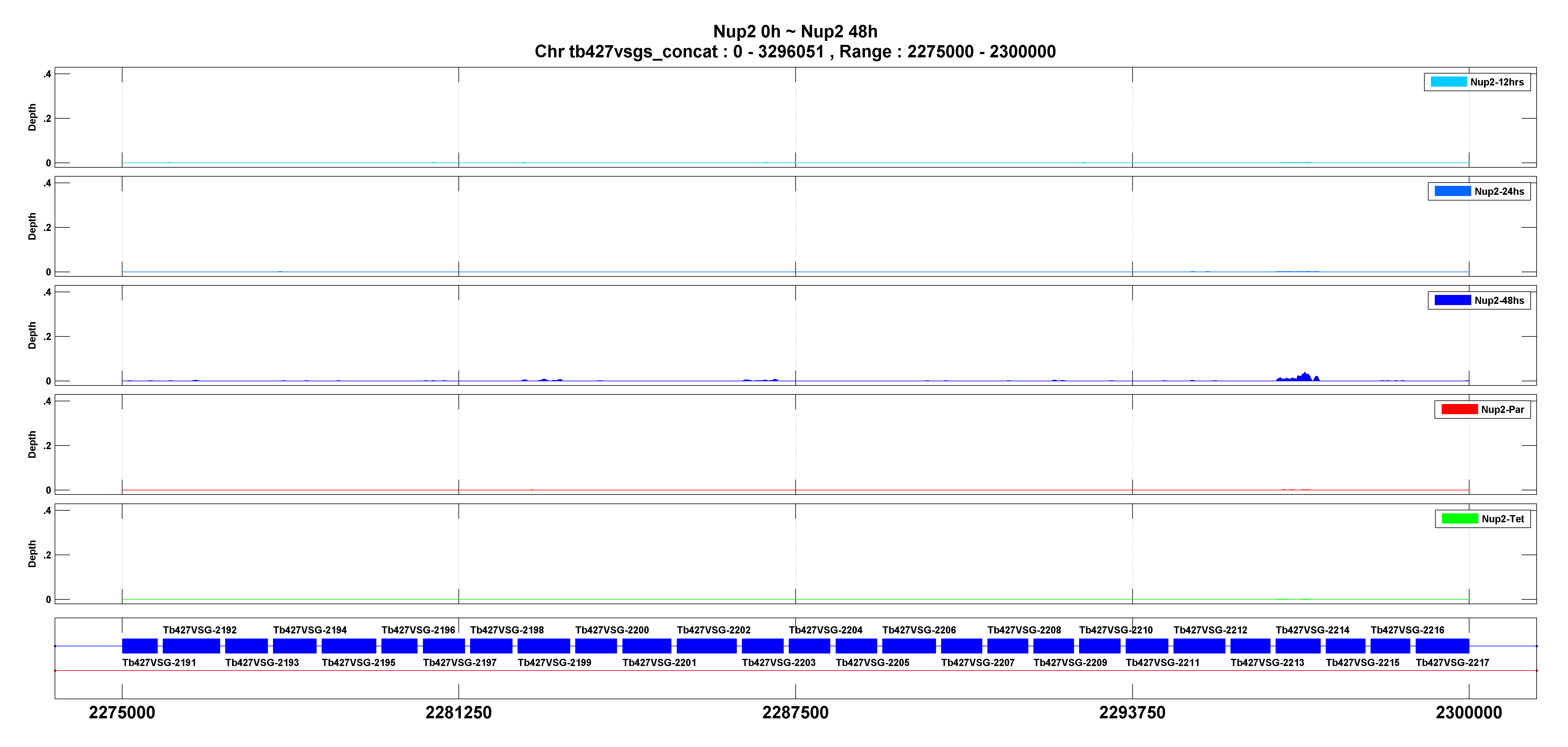

Supplement: SUPPLEMENTARY DATA [file supp_gkw751_nar-01100-x-2016-File026.zip › VSG transcriptome map/fig_tb427vsgs_concat_whole-seq_92.png]
