# Supplementary figures and images for: Co-dependence between trypanosome nuclear lamina components in nuclear stability and control of gene expression (part 2 of 2)
Source: Nucleic Acids Res. 2016 Sep 12;44(22):10554–70. doi: 10.1093/nar/gkw751 (PMC5159534; doi:10.1093/nar/gkw751)

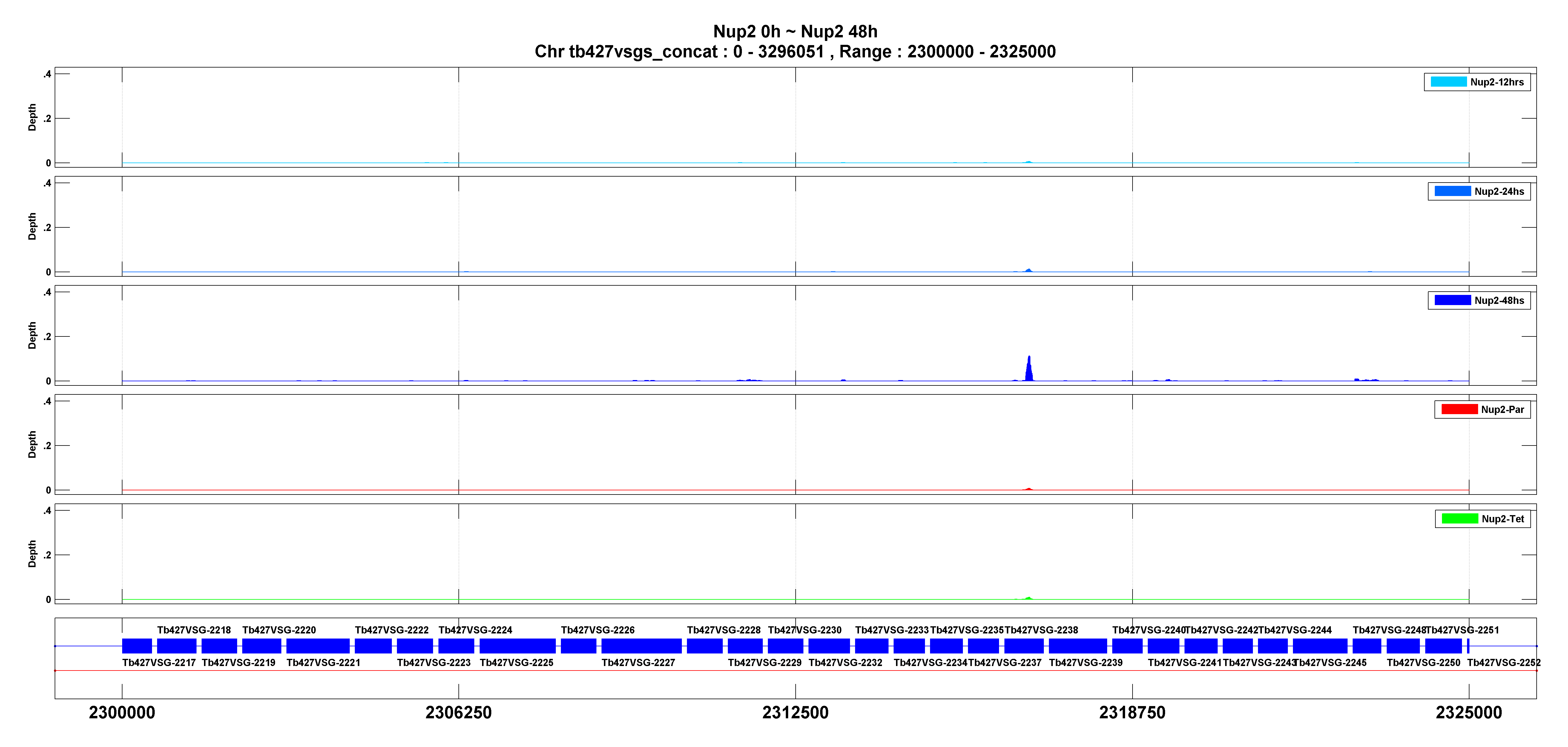

Supplement: SUPPLEMENTARY DATA [file supp_gkw751_nar-01100-x-2016-File026.zip › VSG transcriptome map/fig_tb427vsgs_concat_whole-seq_93.png]

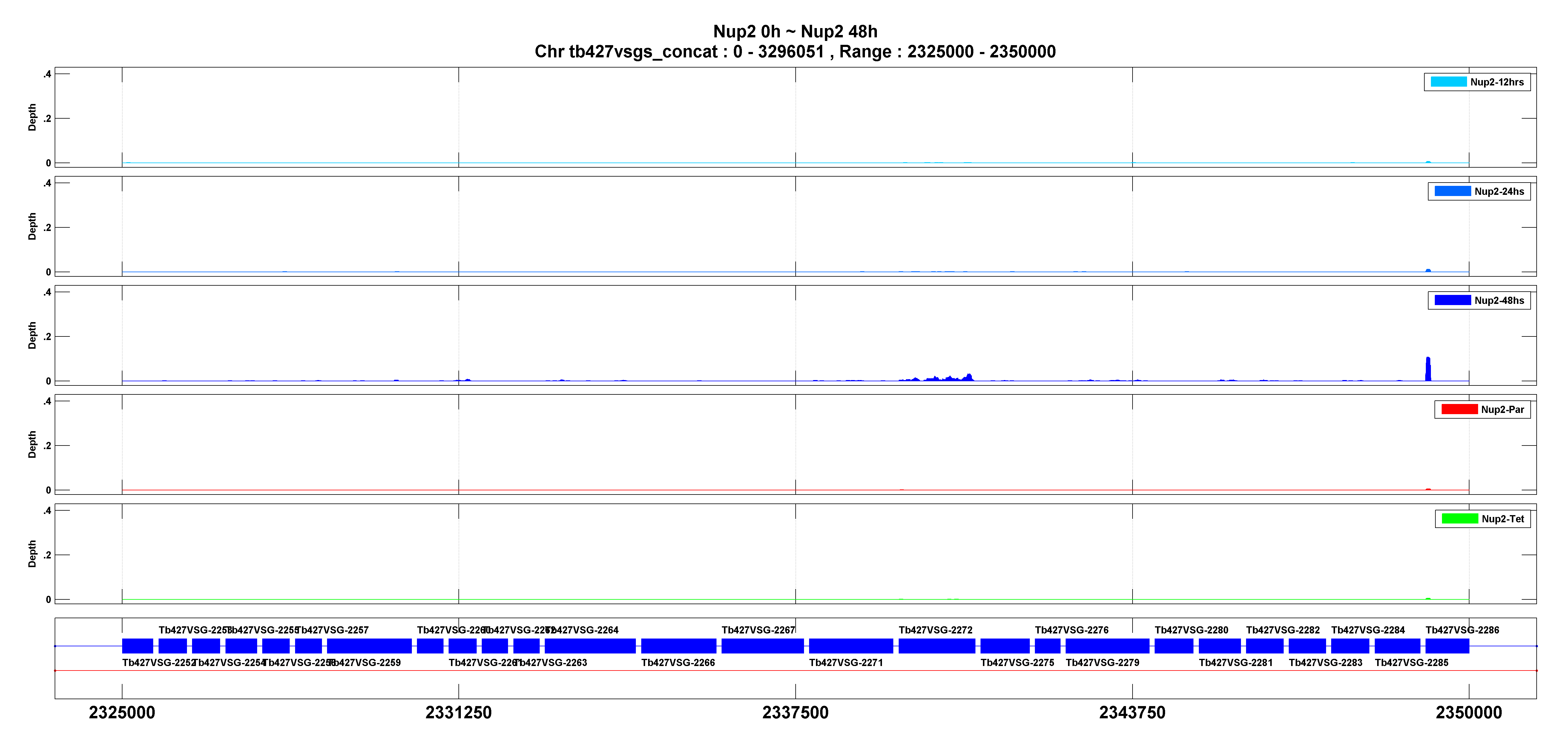

Supplement: SUPPLEMENTARY DATA [file supp_gkw751_nar-01100-x-2016-File026.zip › VSG transcriptome map/fig_tb427vsgs_concat_whole-seq_94.png]

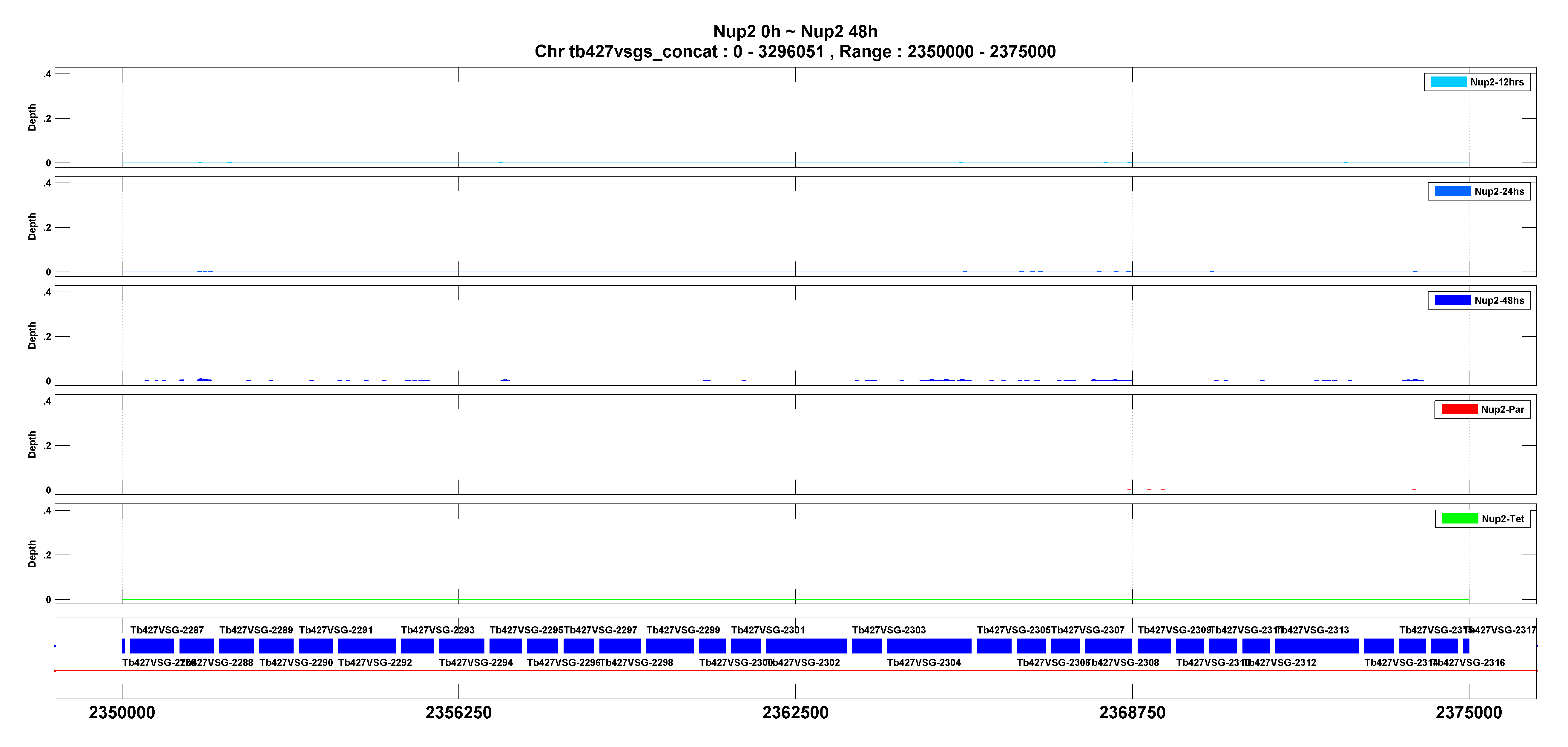

Supplement: SUPPLEMENTARY DATA [file supp_gkw751_nar-01100-x-2016-File026.zip › VSG transcriptome map/fig_tb427vsgs_concat_whole-seq_95.png]

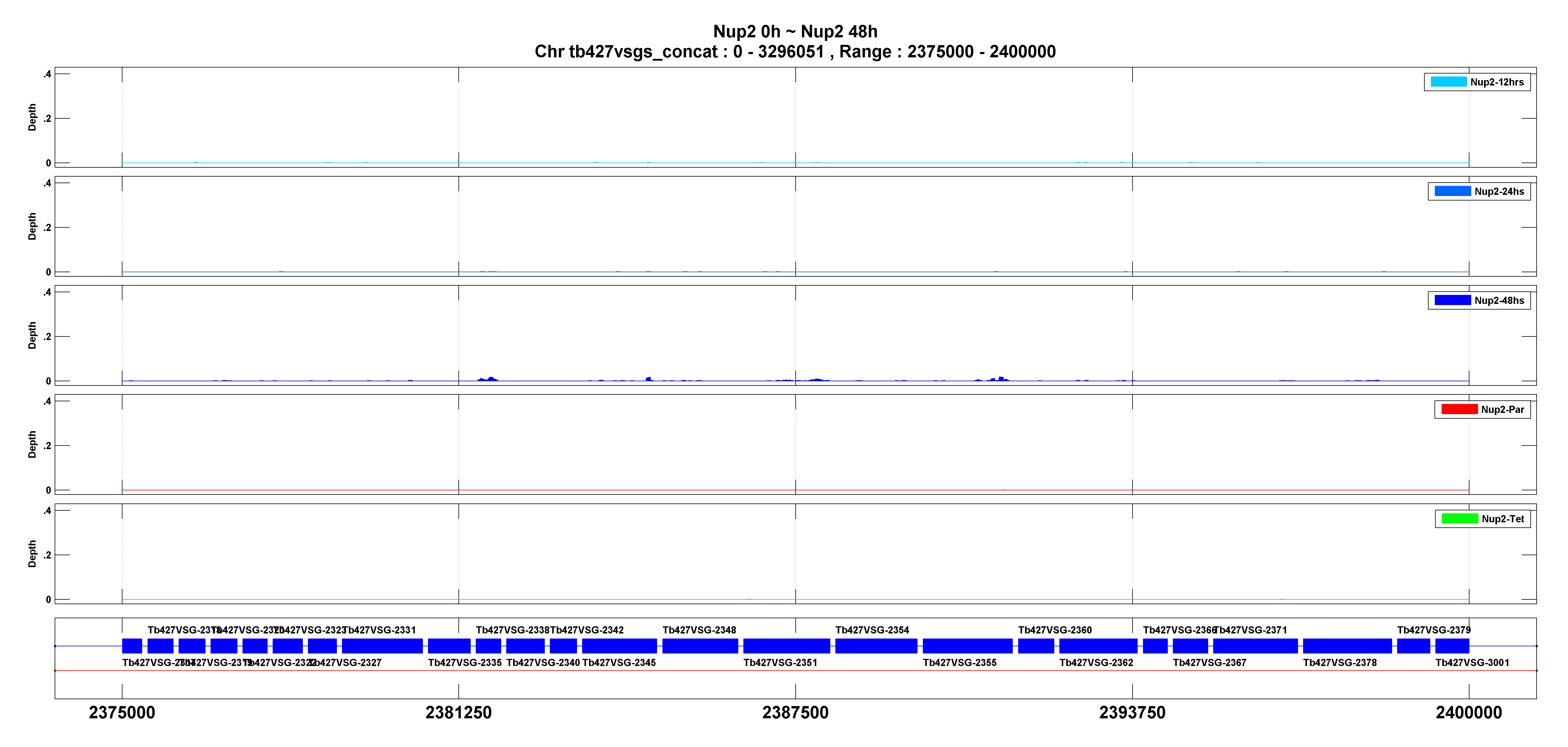

Supplement: SUPPLEMENTARY DATA [file supp_gkw751_nar-01100-x-2016-File026.zip › VSG transcriptome map/fig_tb427vsgs_concat_whole-seq_96.png]

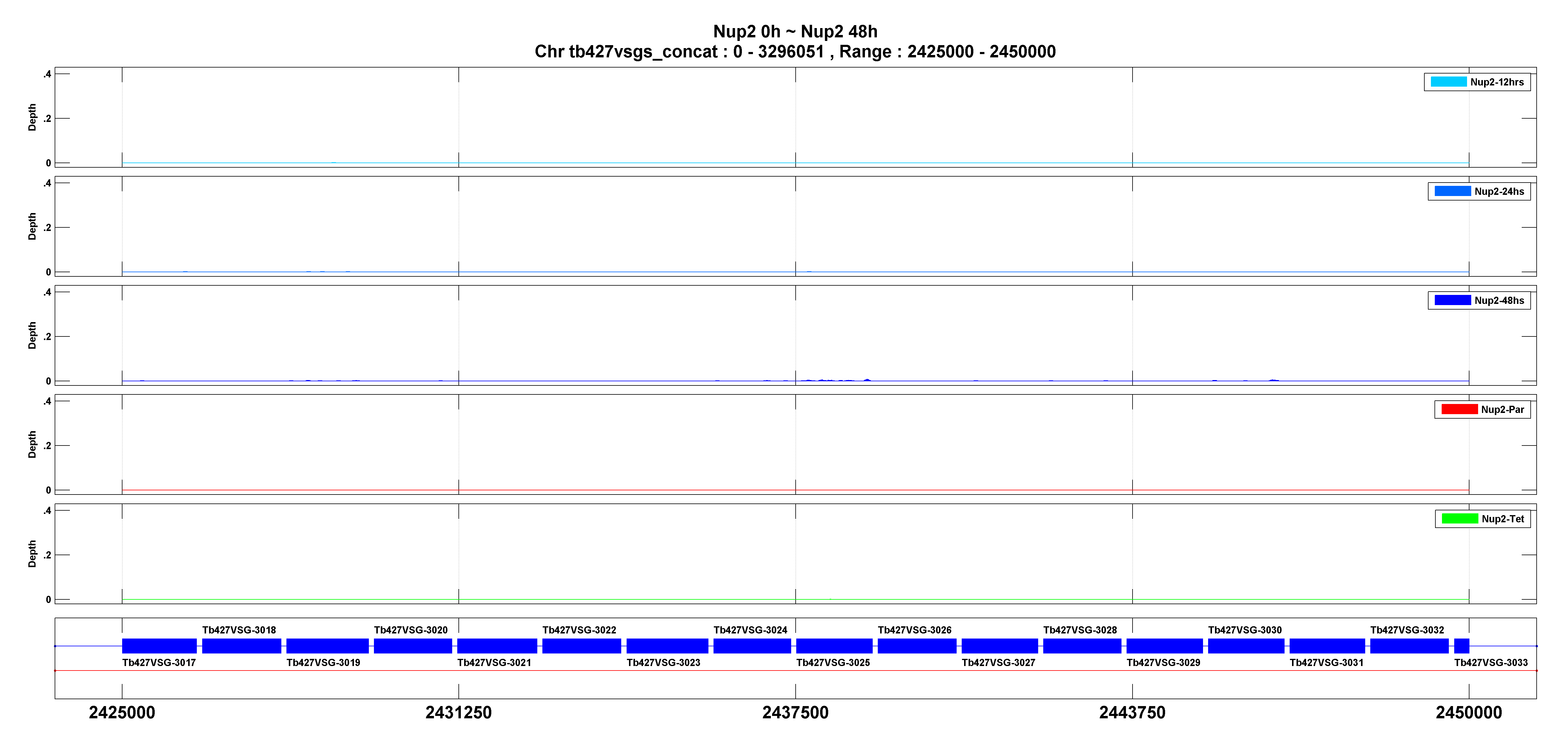

Supplement: SUPPLEMENTARY DATA [file supp_gkw751_nar-01100-x-2016-File026.zip › VSG transcriptome map/fig_tb427vsgs_concat_whole-seq_98.png]
